# Emotion regulation in children and adolescents: a systematic review and quality assessment of behavioral and physiological assessment methods and their theoretical foundations

**DOI:** 10.1186/s40359-026-04935-2

**Published:** 2026-07-01

**Authors:** Melanie Bunz, Rabea Derhardt, Felix Schreiber, Sabine Seehagen, Silvia Schneider

**Affiliations:** 1 Faculty of Psychology, Clinical Child and Adolescent Psychology, Mental Health Research and Treatment Center (FBZ), Ruhr University Bochum, Massenbergstraße 9-13, Bochum, 44787 Germany; 2 Department of Developmental Psychology, Faculty of Psychology, Ruhr University Bochum, Bochum, Germany; 3 German Center for Mental Health (DZPG), Partner Site Bochum/Marburg, BochumMarburg, Germany

**Keywords:** Emotion regulation methods, Children and adolescents, Behavioral observations, Physiological measurements, Theoretical embedding

## Abstract

**Background:**

Emotion regulation (ER) is crucial for understanding mental health and disorders. Valid and reliable ER measures are needed to develop models for mental health promotion and treatment. This systematic review provides a comprehensive overview of ER behavioral observation and physiological measures of children and adolescents, including their theoretical embedding and psychometric properties.

**Methods:**

Following PRISMA guidelines, we conducted a systematic literature search in PubMed, PsycINFO, Psyndex plus, and Web of Science without language restrictions. We included empirical studies assessing ER as an outcome variable in children and adolescents aged 0–15 years using at least one behavioral or physiological ER measure. Studies on secondary literature were excluded. Measurement types, theoretical grounding, and psychometric properties (reliability, validity, objectivity) were extracted and compared across age groups using descriptive statistics and chi-square tests.

**Results:**

A total of 516 studies met inclusion criteria. Only 5% reported validity evidence, 62% reliability, and 20% objectivity. Few studies referenced an ER definition or empirically tested an ER model. LAB-TAB and Delay of Gratification tasks were the most common behavioral observation measures, whereas RSA and EEG dominated physiological measures. Behavioral observation was more frequently used in younger children, whereas physiological measures were more common in older children (*p* < .001; *V* = .37). Most studies focused on the regulation of negative emotions (91%), and 90% relied on a single ER measure.

**Conclusion:**

ER measurement in children and adolescents is characterized by limited psychometric reporting and insufficient theoretical integration. This review provides an overview of available methods and their psychometric properties, facilitating the selection of appropriate measures for future research. Greater emphasis on validity, multimethod approaches, theoretical grounding, and the inclusion of positive emotions is needed to improve measurement standards and advance ER research.

**Supplementary Information:**

The online version contains supplementary material available at 10.1186/s40359-026-04935-2.

## Background

Emotions have essential functions for our survival: they prepare us for rapid motor actions [104], help us to deal with environmental changes [163], and inform and prepare us for social situations [47, 87]. Emotion regulation (ER) is defined as “the processes responsible for monitoring, evaluating, and modifying emotional reactions […] in order to accomplish one’s goals” ([233] pp. 27–28). In children, ER is important for lifetime health, being associated with psychological well-being [4], social competence [226] and mental health outcomes [122]. Low ER abilities in children are associated with later development of internalizing and externalizing disorders [94, 126, 216]. ER, as a transdiagnostic factor [96], plays a central role in understanding the development of mental health and mental disorders [122, 166]. Accordingly, valid assessment of ER in childhood is crucial for understanding and promoting mental health early in life. However, the conceptualization and measurement of ER remain inconsistent [2, 18, 60, 139, 172, 215, 222]. For example, the two-factor concept assumes that an emotion must first be elicited before regulation occurs [2, 48], whereas the one-factor concept views emotions and their regulation as inseparable components of a unified process [48]. These conceptual differences are reflected in heterogeneous measurement approaches, complicating the comparison and integration of findings across studies.

**Fig. 1:**
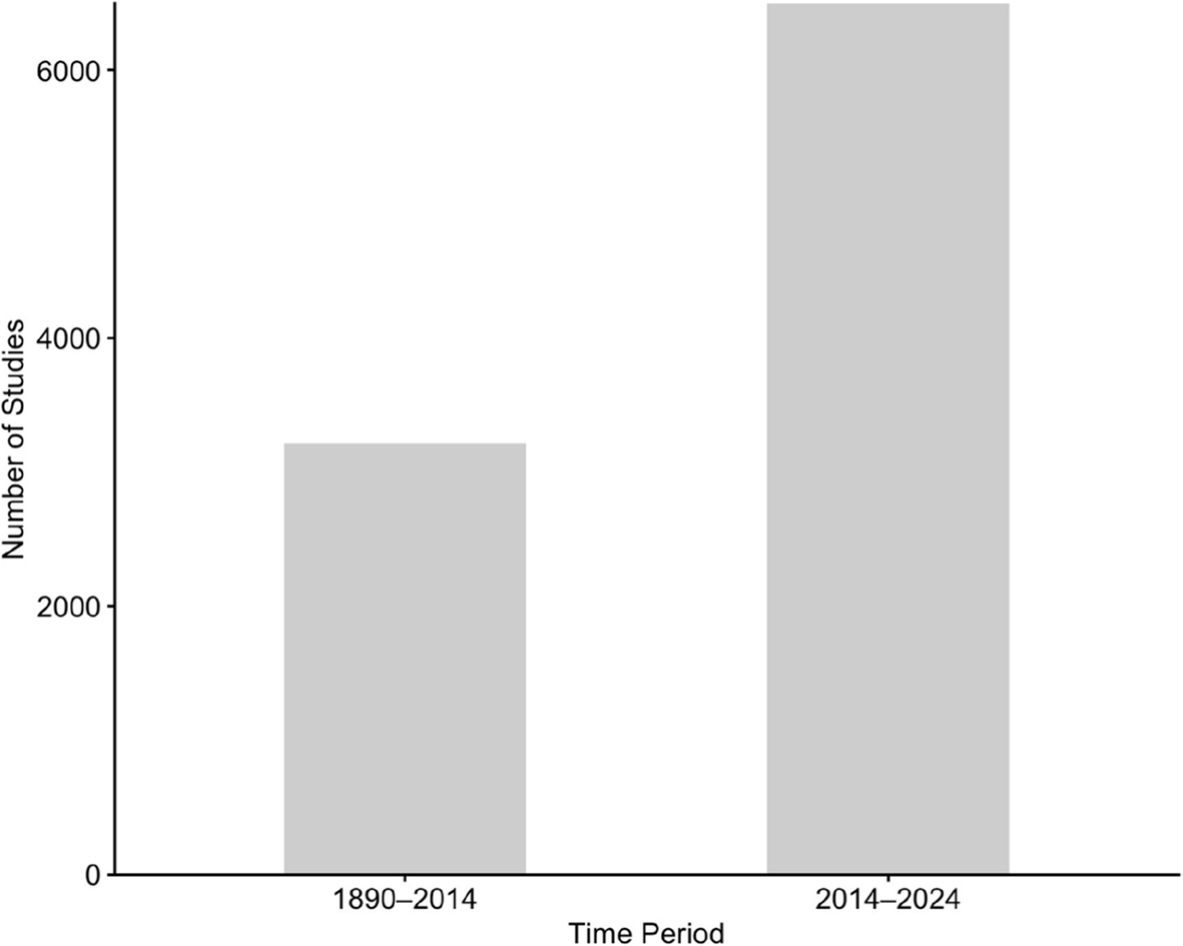
Number of PsycInfo records across two time periods The figure shows the number of records retrieved from PsycInfo for the periods 1890–2014 and 2014–2024, illustrating the increase in publications over time

Considering the existing evidence, several reviews and meta-analyses have examined ER in children and its links to psychopathology, social competence, and academic outcomes [4, 7, 61, 73, 78, 83, 85]. Prior reviews have summarized ER assessment methods in children and adolescents and provide a valuable overview and discussion [2, 60, 146, 260]. However, these reviews show several limitations: some lacked systematic analyses of primary studies [60, 260], focused on restricted age ranges [60], did not evaluate psychometric quality [2] or applied narrow eligibility criteria when selecting studies for inclusion [146]. In addition, to our knowledge, the most recent comprehensive review dates back to 2011, highlighting the need for an updated synthesis of the literature. For example, a search in PsycInfo for the terms “emotion regulation” OR “emotion management” in the preset age groups 0–17 years yields more than twice as many publications from the last ten years compared to the period from 1890 to 2014 (see Fig. 1).

Thus, in light of the growing body of literature, an up-to-date systematic review of behavioral and physiological ER measures in children and adolescents is required. In particular, their psychometric properties and theoretical foundations have not yet been comprehensively evaluated. This gap is particularly relevant given the variability in conceptualizations of ER and the previously limited systematic evaluation of existing measurement approaches. For adequate assessment of ER and its developmental trajectories in childhood, valid and age-appropriate measures as well as clear theoretical foundations are essential [135].

The present systematic review aims to address this gap. Specifically, we examined the number and types of behavioral and physiological ER measures used in children and adolescents. Furthermore, we evaluated their reported psychometric properties, including reliability, validity, and objectivity, and analyzed the theoretical definitions and models underlying these measures. Based on previous literature [2], we formulated the following hypotheses: (1) studies will differ in their underlying ER definitions and models, representing the one- and two-factors concept [48], (2) behavioral observation measures will be more frequently used in studies with younger children, whereas physiological measures will predominate in studies with older children, (3) single-method assessments of ER will be more common than multimethod approaches and (4) reliability of ER methods will be reported more often than validity.

## Methods

### Study design

This study is a systematic review of empirical research on behavioral observation and physiological measures of ER in children and adolescents (0–15 years). The review was conducted following the PRISMA 2020 guidelines [187] see checklists, Appendix, File 1 and File 2). The present review focuses exclusively on studies that assess ER using behavioral observations and physiological measurement methods. Although the literature search also identified studies employing other assessment approaches, such as questionnaires, interviews, narratives, EMA/apps, and computer-based tasks, these were not included in the present review but will be addressed in a separate, forthcoming review.

### Transparency and openness

All steps of the review were documented in accordance with open science practices. The review protocol was pre-registered at PROSPERO (ID: CRD42021266452, Link: https://www.crd.york.ac.uk/PROSPERO/view/CRD42021266452). All data, analysis code, and study materials are publicly available at Psych Archives. Modifications to the pre-registered eligibility criteria and search strategy were made due to an initially large number of hits (over 35,000). (Dataset Link: 10.23668/psycharchives.22185) (Code Link: 10.23668/psycharchives.22186).

### Eligibility criteria

Studies were included according to the PICOS criteria [177]. Eligible studies included participants from birth to 15 years and 11 months. Studies were required to use at least one ER measurement and report ER as an outcome variable. Both studies with and without group comparisons were eligible. No language restrictions were applied; studies in all languages were included. We initially pre-registered an age range of 0 to 18 years. However, this age range was reduced to 0 to 15 years and 11 months due to the large number of eligible records identified. Studies reporting on secondary literature (e.g. meta-analyses, reviews) were excluded.

### Search strategy

The search strategy was piloted in PubMed and adapted for each database (search strategy for PubMed, Appendix, File 3). The search was updated continuously until 13.09.2024. We used topic-specific databases PubMed (including MEDLINE; Ovid), PsycINFO (including PsycArticles; EBSCOhost), Psyndex plus (EBSCOhost) and Web of Science (Thomson Reuters, Clarivate Analytics) for adjoining disciplines. In contrast to the previously pre-registered search strategy, the Google Scholar database was excluded due to the large number of records retrieved, which exceeded the feasible scope of screening. Instead, Psyndex Plus was included as an additional database. No publication date limits were applied. For studies with unavailable full-texts, authors were contacted or articles purchased. Additionally, manual reference checks and citation tracking in the Social Science Citation Index were conducted.

### Measurement tools

#### Abstract screening

An individualized abstract screening tool, based on the eligibility criteria, was developed. After screening 2977 articles, the artificial intelligence “Rayyan” was used for further screening. Rayyan has been proved as a reliable tool for abstract sreening [38, 214, 246]. In this review, we found a substantial overall agreement (Cohen’s *κ* = 0.78; [160]).

#### Data extraction

For data extraction, an a priori-developed tool was piloted on the first five articles. Extracted data comprised study identification, study and measurement characteristics (study design, publication type), sample characteristics (age, gender, ethnicity, child/family characteristics), definition/model of ER, measurement of ER and psychometric properties of ER measurements (reliability, validity, objectivity).

#### Risk of bias ratings

For risk of bias ratings, study design-specific tools were used. The revised Cochrane Risk of Bias tool [227] was applied to randomized controlled trials, as it is specifically designed to evaluate internal validity in randomized designs. For non-randomized and observational quantitative studies, the Effective Public Health Practice Project (EPHPP) Quality Assessment Tool was used [232]. The EPHPP tool was selected because it enables standardized assessment across heterogeneous quantitative study designs and has demonstrated good reliability and applicability in public health and psychological research [9, 140, 232]. Within the EPHPP rating scheme, we excluded the “Study Design” section, as it was not applicable to non-RCT studies. Interrater agreement before reaching consensus for the global rating on the EPHPP scale was substantial (Cohen’s *κ* = 0.62; [160]) as well as for the RoB 2 Rating Scale (Cohen’s *κ* = 0.79). To facilitate comparison across studies, RoB 2 ratings were converted into a numerical format consistent with the EPHPP system (1 = low risk, 2 = some concerns, 3 = high risk). In the EPHPP tool, domains are rated as strong, moderate, or weak and a global rating is assigned based on the number of weak domains. RoB 2 classifies studies as low risk, some concerns, or high risk, depending on the highest risk level across domains.

### Procedures for screening, data extraction, and risk of bias assessment

Titles and abstracts were independently screened by two authors (MB, RD) and two advanced graduate students. Each record was screened by at least one of the two authors. Discrepancies (29%) were resolved through discussion and consensus. All potentially eligible articles were independently assessed in full text by three authors (MB, RD, FS) and two advanced graduate students. Each full-text review involved at least one of the authors. Reasons for exclusion were documented throughout the process. Disagreements were resolved by consensus. Risk of bias assessments were also conducted by the three authors (MB, RD, FS) and trained graduate students. Any discrepancies in ratings were discussed until consensus was reached.

### Outcomes and statistical analyses

The primary outcome was the reporting of psychometric properties in behavioral and physiological ER measures. Differences in reporting frequencies across psychometric properties were examined using Fisher’s exact tests. The proportion of studies reporting psychometric properties was estimated using a random-effects meta-analysis of logit-transformed proportions with inverse-variance weighting and Hartung–Knapp adjustment. Heterogeneity was assessed using Cochran’s Q and the I^2^ statistic. Subgroup analyses were conducted by age group and measurement type, with between-group differences evaluated using Q-tests. Sensitivity analyses were performed to assess the robustness of the meta-analytic estimates of the overall proportion of studies reporting psychometric properties. Specifically, the main meta-analysis was repeated within subgroups defined by risk-of-bias ratings (low, moderate, high). To examine whether studies reporting psychometric properties differed from those that did not, studies were dichotomized based on whether at least one psychometric property was reported. Group differences were analyzed using Wilcoxon rank-sum tests for continuous variables (e.g., sample size, gender, and ethnicity proportions), due to skewed data distributions, and chi-square tests (or Fisher’s exact tests, where appropriate) for categorical variables (e.g., measurement type, age group, child characteristics, language, study design, and country group).

**Fig. 2:**
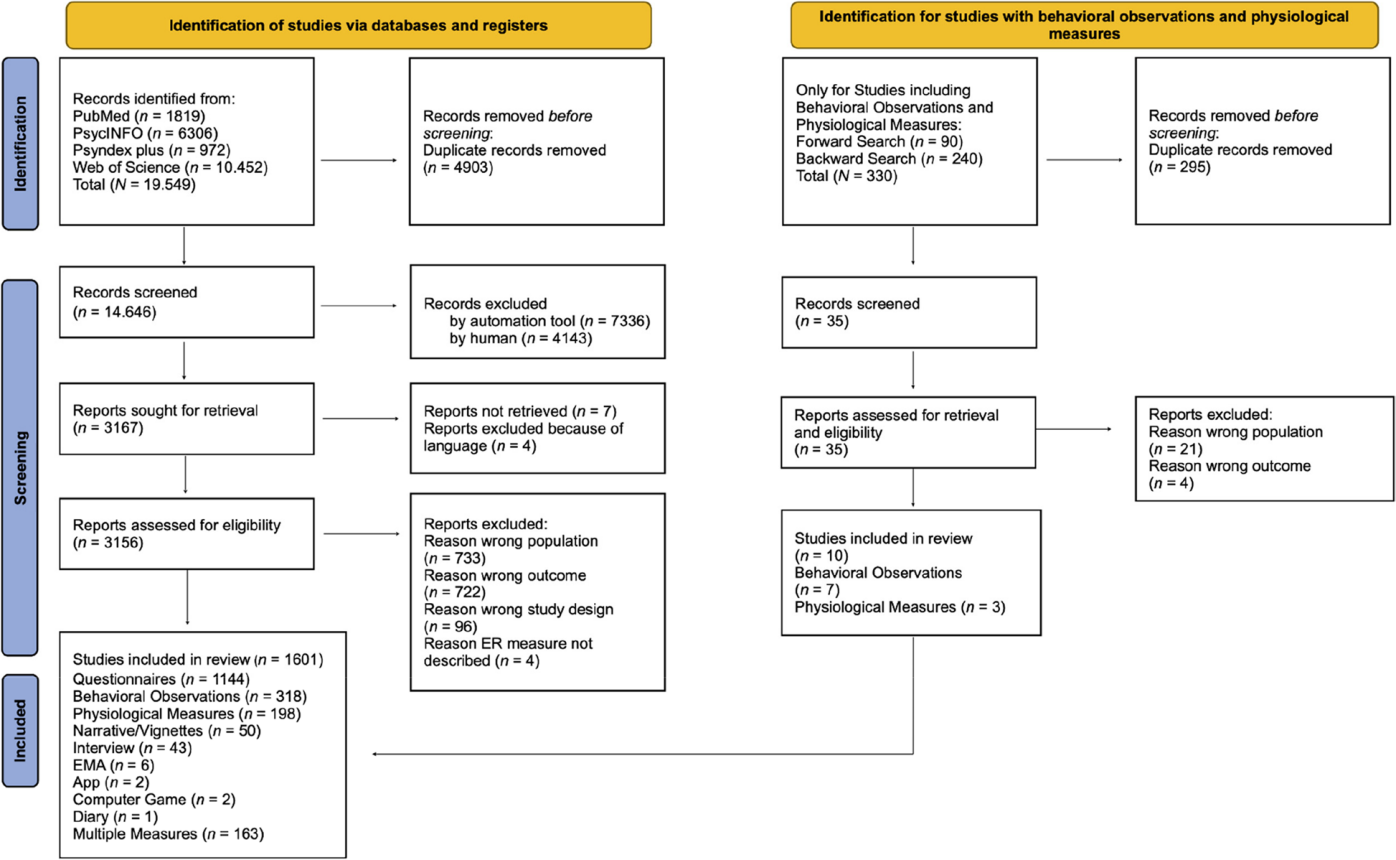
The flow diagram shows the literature search and study selection process The left panel shows identification and screening of all studies, while the right panel shows additional manual reference checks and citation tracking in the Social Sciences Citation Index for studies including behavioral observations and physiological measures

Data preparation and descriptive statistics were conducted in IBM SPSS Statistics (version 29). Meta-analyses were performed in R (version 4.4.1) using the meta package (Fig. 2).

## Results

### Study characteristics

In total, 1,601 studies were included. Most studies used questionnaires (*n* = 1,144), followed by behavioral observations (*n* = 318), and physiological measures (*n* = 198), with additional approaches including narratives or vignettes, interviews, ecological momentary assessment (EMA), apps, computer games, or diaries. A total of 163 studies (10%) employed more than one ER measurement method. This review focuses specifically on studies using behavioral observations and physiological measures (*n* = 516). Seven studies were excluded because researchers could not be contacted and articles could not be be purchased. Four articles were excluded due to the Japanese language, as no Japanese-speaking associate was available in our laboratory. Other exclusions were based on ineligible age groups, absence of an ER outcome measure, secondary data, or insufficient detail on ER measurement. Among the included studies, none were excluded for using the same sample, as each study employed different ER measures. This applied to four studies using physiological measurements and seven studies using behavioral observation measures. Most included studies were published in English (*n* = 311), with few in Spanish, German, French, or Italian (Appendix, Table 2). Most studies appeared in peer-reviewed journals (Appendix, Table 3). Descriptive designs without control groups were most common, followed by longitudinal and case–control studies; RCTs were least frequent (Appendix, Table 4). The earliest included study was from 1989, the most recent from 2024.

### Child and family characteristics

In total *N* = 104,152 children were examined (*n* = 61,318 in behavioral observation studies and *n* = 42,834 in physiological measurement studies). In studies on behavioral observations where gender was specified (*n* = 262), on average, 55% of the children were male. Studies included children aged from 0 to 180 months (Suppl. Mat., Table 1a-1e). The majority of the studies on behavioral observation were conducted predominantly with White children (*M* = 67%; *SD* = 25.72; with *n* = 136 studies providing this information) and only few studies (*n* = 21) provided information on socioeconomic status (SES) or family income. Regarding child health, most studies involved healthy children; only 49 studies (15%) included participants with psychological or physical disorders. Among psychological disorders, autism and externalizing disorders were most commonly studied (Table 1). For studies on physiological measures, on average 53% of the children were male (*n* = 176 studies provided information on gender), the majority of the examined children were White (*M* = 61%; *SD* = 25.88; *n* = 92) and only five studies reported SES or family income. Studies included children aged from 0 to 189 months (Suppl. Mat., Table 2a – 2e). In terms of mental health, most studies using physiological measures focused on healthy children. Of the 36 studies (18%) examining psychological or physical disorders, externalizing disorders were most frequently investigated (Table 1).

**Table 1:** Child and family characteristics according to study type

Characteristic	Behavioral Observation Studies			Physiological Measure Studies		
Gender Ratio (%)	*M*	*SD*	*n*	*M*	*SD*	*n*
Male	55.48	14.58	262	52.90	15.93	176
Female	44.51	14.58	262	47.09	15.93	176
Ethnicity (%)	*M*	*SD*	*n*	*M*	*SD*	*n*
White	66.69	25.72	136	61.09	25.88	92
Black/African American	22.93	24.30	87	22.36	23.34	75
Latino/Hispanic	22.15	26.53	65	11.24	10.72	51
Asian	9.15	17.03	41	9.75	15.63	51
Mixed Race/Others	8.54	8.19	63	11.11	9.54	65
Native American	3.6	5.03	11	3.16	2.45	11
Family Income/SES	*n*		%	*n*		%
Low income	20		6.30	4		2.00
Middle income	1		.30	1		.50
High income	0		0	0		0
Child Health	*n*		%	*n*		%
Externalizing Disorders	20		6.30	21		10.60
Autism	16		5.00	4		2.00
Physical Diseases	4		1.30	2		1.00
Stuttering	4		1.30	5		2.50
Internalizing Disorders	2		.60	3		1.50
Internet Addiction	1		.30	0		0
Tourette Syndrome	1		.30	0		0
Obesity	1		.30	1		.50

*M* Mean, *SD* Standard deviation, *n* Number of studies

### Risk of bias ratings

For studies on behavioral observation measures, both rating schemes (RoB and EPHPP) indicated a low risk of bias in 51% of studies, a moderate risk in 41%, and a high risk in 8%. For studies using physiological measurements, 49% showed low risk of bias, 47% moderate risk and 4% high risk according to both schemes. Across measurement types, most moderate ratings in the RoB 2 scheme were due to the domain "Selection of the reported results", with 43% of studies (*n* = 13) lacking pre-registration. Additionally, four studies (13%) received moderate ratings due to insufficient reporting of blinding and dropout analyses. Similarly, in the EPHPP scheme, high-risk ratings were most often due to insufficient reporting of blinding (*n* = 67; 15%) and dropout procedures (*n* = 93; 21%).

### Definitions of emotion regulation

In total, 47 different definitions of ER were identified within the included studies. Of these, the definition by [233] was the most frequently cited, followed by the definition by [117]. Both definitions describe ER as a process of emotion generation and response emphasizing that ER depends on personal and environmental factors, referred to as a functionalist view. Of the 47 definitions, 24 (51%) followed this functionalist view, 17 (36%) could be classified into the one-factor concept and none into the two-factor concept Appendix, Table 6). Other definitions highlighted various aspects of ER, such as its physiological components (e.g., emotional arousal; 26%; [53, 115, 133, 208], developmental perspectives (2%; [13, 234]) or conceptualized ER as self-regulation (11%; [1, 116]), temperament (2%; [110]) or as a factor of coping (2%; [103]). Some definitions emphasized the multifactorial nature of ER (17%; [105, 229, 234]), its role as both, a conscious and unconscious process (9%; [117, 127, 173]) and its homeostatic function, as ER skills facilitate a return to equilibrium [29]. Some definitions also described ER as goal-oriented (23%; [41, 51, 84, 113, 114, 118–120, 229, 233]). These variations and differing emphasis on the functions and components of ER underscore its inherent complexity and multifactorial nature. This variability supports the argument for employing a range of assessment tools when evaluating ER, as a single measure may fail to capture the full breadth of its dynamic processes [2].

### Models of emotion regulation

According to the models of ER, eight different models were identified within the included studies (Appendix, Table 7). The most frequently cited model across all studies was the Transactional Model [211], which conceptualizes ER in children as an interactive process between the individual and others. The second most cited was the Process Model [117], which distinguishes between antecedent-focused strategies (e.g., situation selection, modification, attentional deployment, cognitive change) and response-focused strategies (e.g., response modulation). In contrast to the definitions, the models primarily focus on the development of (ER) in children, considering factors such as parent emotion socialization [82, 178], caregiver-child interactions [108], and the intergenerational transmission of ER patterns [35]. Additionally, some models underscore the development of ER across multiple dimensions, such as physiological, emotional and attentional processes [258]. Notably, the Polyvagal Theory is distinct in its focus on the physiological underpinnings of ER, highlighting the role of parasympathetic control over cardiac activity [195, 196]. Overall, extremely few studies cited or tested ER models (87, 5%) or cited an ER definition (704, 44%).

### Behavioral observations and physiological measurements

#### Structure of tables

Types of measures for ER based on behavioral observations and physiological measurements are described in the Supplementary Material (Tables 1a–1e and 2a–2e). For each study, the minimum age at which the ER measurement was applied was used; if no minimum age was reported, the mean age was applied. Based on these values, measures were organized into broader age groups (neonates, infants, toddlers, preschool, school-aged children and teens) according to the American Academic of Pediatrics [6]. These broader categories were further divided into narrower age groups of 12 months categories. Within these 12 months categories the measures are organized according to the examined emotions (frustration/anger/disappointment, fear, jealousy, empathy, joy and non-specific emotion/mixed emotions). For the behavioral observations (Suppl. Mat., Table 1a – 1e), the task of how the emotion is triggered is described first, followed by the duration of this task, the coding manuals used to measure ER, information on reliability, validity and objectivity and the citations and number of studies using the emotion eliciting task. Specific coding manuals are not described for all tasks. The coding procedure can be found in the studies that use the emotion eliciting task. For the physiological measurements (Suppl. Mat., Table 2a – 2e), the measurement method (e.g. EEG, fMRI) is described first, followed by the measured physiological phenomenon, the emotion triggering tasks, information on reliability, validity and objectivity of the measured physiological phenomenon and the citations and number of studies measuring the physiological phenomenon.

#### Descriptive results according to behavioral observations studies

##### Types of measurements by age groups

Behavioral observation measurements on ER could be found for each age group. Most studies on behavioral observations were found for the age group of 13 to 24 months. The fewest studies could be found for the age group of neonates and 15-year-olds. Only two studies were found that assessed ER using the Neonatal Behavioral Assessment Scale [34] and the Arm Restraint Task [111] and only one study involving 15-year-olds was found, using the Conflict Discourse Task [15]. For infants, the most commonly used task was the Still-Face Paradigm [238], whereas for toddlers and preschoolers different versions of Delay of Gratification Tasks [176] were most commonly used. Most studies in school-aged children used the Disappointment Paradigm and in teens the Puzzle Task [129, 130].

Tasks used across broad age ranges in longitudinal designs included the Attractive Toy Behind Barrier Task (1–13 years; mainly with autistic children in older age groups; [11, 30, 32, 63], Attractive Toy in a Transparent Box Task (1–10 years; [12, 17, 27, 31]), the Disappointment Paradigm (2–9 years; [12, 74, 76, 99, 100, 182, 237], various Problem-Solving Tasks (1–9 years; [49, 142, 162, 183, 185]), emotional film clips (3–14 years; [66]), the Picture Distraction and Reappraisal Task (7–14 years; [128, 153]), the Children’s Play Therapy Instrument (4–10 years; [132–134]), the Triadic Interaction Paradigm, (2–7 years; [175]) and the non-opening Bottle Task (1,5–5 years; [255]). According to coding schemes, most of the studies used own coding schemes. The most frequently used coding manual was the Dysregulation Coding Scheme [142].

##### Type of measurements by elicited emotions

Measures were categorized as either non-specific/mixed emotional tasks or tasks targeting specific emotions such as frustration, anger, joy, or fear. Common frustration-inducing tasks included the Still-Face Paradigm, Delay of Gratification, Disappointment Paradigm, the Attractive Toy in a Transparent Box Task, the Attractive Toy Behind Barrier Task, and the High Chair Task, while the Spider Task was most frequently used for fear. The Preschool Self-Regulation Assessment was the most common non-specific emotional task.

Across all studies, 985 behavioral observation tasks were identified. Most elicited frustration (*n* = 532; 54%) or non-specific/mixed emotions (*n* = 339; 34%), with fear (*n* = 66; 7%), joy (*n* = 36; 4%), jealousy (*n* = 7; 1%), and empathy (*n* = 5; 1%) less common. Overall, 61% of tasks elicited negative emotions and only 4% positive emotions. Younger children were mostly tested with frustration-related tasks, whereas older children experienced more non-specific or mixed emotional tasks.

##### Psychometric properties of the measures

A total of 418 psychometric measures were reported for the ER tasks. Among these, 306 (73%) were reliability measures, 20 (5%) were validity measures, and 90 (22%) were objectivity measures (Appendix, Table 8).

##### Reliability

Overall, the included articles provided reliability information for almost all extracted ER tasks. Interrater reliability was the most commonly reported reliability index. Across all tasks interrater reliability ranged from 0.39 to 1.00, with most estimates falling within the good to excellent range (cf. [55]). Only one study assessed reliability beyond interrater agreement by additionally reporting Spearman Brown split-half reliability for the Emotional Oddball Task in an acceptable range [57, 58].

##### Validity

For validity measures of ER, the included articles on behavioral observations provided limited information. No validity data were reported for neonates, infants, or adolescents aged 12 to 15 years. The Delay of Gratification Task showed good construct, concurrent and predictive validity in toddlers, preschoolers and school-aged children [145, 186]. Several Lab-TAB tasks demonstrated moderate to strong convergent validity in toddlers and preschoolers [65, 81, 89, 106, 112, 145, 223, 259], and good construct and predictive validity in school-aged children [112, 136, 259]. Studies using the Preschool Self-Regulation Assessment and the Toy Sort Task, included evidence for construct and convergent validity in toddlers and preschoolers [223]. In school-aged children, the Impossible Building Tower Task showed good construct validity [205], while the Impossible Dexterity Task demonstrated support for concurrent and predictive validity [154]. The Puzzle Task showed mixed results, ranging from weak to good convergent and discriminant validity in older children and adolescents [16, 95, 142, 183].

##### Objectivity

Most of the studies ensured evaluation objectivity through blinded coding procedures. Coders were blind to hypotheses, group and intervention conditions [10, 11, 39, 56, 91, 93, 125, 137, 152, 158, 167, 171, 179, 186, 206, 225, 257]. Some studies also described implementation objectivity through assessors being blind to study hypotheses and purposes [40, 52, 67, 133, 179].

#### Descriptive results according to physiological measurement studies

##### Types of measurements by age groups

For all broader age groups physiological measurements were reported. The largest number of studies using physiological measures was conducted in 8- to 9-year-olds, while the fewest focused on neonates and 15-year-olds. Only one study assessed vagal tone during resting states in neonates [91]. In infants, electrocardiography (ECG) was the most frequently used method to examine heart rate, heart period, heart rate variability (HRV), and vagal tone. These measures provide insight into autonomic nervous system activity and are commonly interpreted as indicators of ER capacity [92]. Respiratory sinus arrhythmia (RSA), a specific component of HRV reflecting the rhythmic influence of breathing on heart rate, is widely interpreted as an index of parasympathetic nervous system activity and an indicator of the capacity for ER [123, 197]. In toddlers, RSA was the most frequently measured phenomenon. Preschool-aged children were studied using RSA as well as electroencephalography (EEG) measures, including event-related potentials (ERP), late positive potentials (LPP), and frontal EEG asymmetry, which reflect neural correlates of attentional and emotional processing [101, 131, 253]. In school-aged children and adolescents, studies primarily employed ECG (RSA), EEG (ERP, LPP, asymmetry), and functional magnetic resonance imaging ((f)MRI) to assess brain volume, whole-brain activity, and regions of interest (ROI) associated with emotional processing [71, 72, 79, 180, 193, 236, 248]. Overall, RSA and EEG measurements (ERP, LPP and asymmetry) were the most commonly investigated physiological phenomena across all age groups.

Longitudinal studies examined a variety of physiological measures across development. ECG measures of heart rate were assessed from 1 to 12 years [88, 158, 254, 256]. HRV was measured from 6 to 13 years years [62, 157], and vagal tone from 4 to 10 years [161]. RSA was assessed in children aged 3 to 14 years [99, 100, 107, 149, 149, 155, 231, 254]. EEG asymmetry measures were reported from 3 to 15 years [71, 99, 100], LPP measures from 5 to 15 years [75, 242, 248, 251], and ERP measures from 8 to 14 years [251]. Functional MRI ROI analyses covered 3 to 15 years [90, 180, 191, 192, 243, 244], and whole brain analyses from 7 to 14 years [243, 244].

##### Type of measurements by elicited emotions

A total of 856 physiological measurements were identified and categorized by emotion type. Most tasks elicited non-specific or mixed emotions (*n* = 587; 69%), followed by frustration (*n* = 234; 27%), fear (*n* = 20; 2%), empathy (*n* = 8; 1%), and joy (*n* = 7; 1%). Overall, 30% of tasks elicited negative emotions, compared with 2% eliciting positive emotions. Tasks for infants mainly targeted frustration, anger, or disappointment, while in children, non-specific or mixed emotions were most common; fear, empathy, or joy were rarely assessed. Many tasks used to elicit frustration, anger, or disappointment overlapped with those in behavioral observation studies.

##### Psychometric properties of the measures

A total of 33 psychometric measures for ER were reported in the included physiological measurement studies, considerably fewer than reported for behavioral observation studies. Of these, six (18%) assessed validity [36, 141, 148, 181, 188, 249], 13 (39%) reliability [31, 32, 147, 156, 174, 198, 202, 217, 230, 231, 241, 245], and 14 (42%) assessed objectivity (see Appendix, Table 8; [31, 32, 43, 99, 100, 138, 158, 198, 202, 217, 230].

##### Reliability

Excellent interrater reliability was reported for several EEG studies across infants, toddlers, preschoolers, and school-aged children, including ERP, asymmetry measures, and LPP [77, 156, 164, 230, 245]. Similarly, excellent interrater reliability was reported for several studies on RSA across broad age groups using different emotion-eliciting tasks [31, 147, 174, 198, 202, 217, 231, 241]. In addition, split-half reliability ranged from poor to good (cf. [58]) in an EEG study on ERP in preschool and school-aged children [245].

##### Validity

No direct validity measures were reported. However, several studies provided support for convergent validity by examining association between physiological measures and behavioral observation measures or questionnaire-based assessments of ER [90, 99, 100, 141, 148, 181, 204]. Support for convergent validity was also reported for heart rate and HRV measures in school-aged children, as the findings were consistent with previous research [36, 97, 98, 141]. No information on validity was provided in studies including adolescents.

##### Objectivity

Six studies reported objectivity through the use of blinded coders [31, 32, 99, 100, 138, 158, 230] and four studies reported implementation objectivity through blinded assessments [43, 198, 202, 217].

#### Inferential results by measurement type and age group

**Fig. 3:**
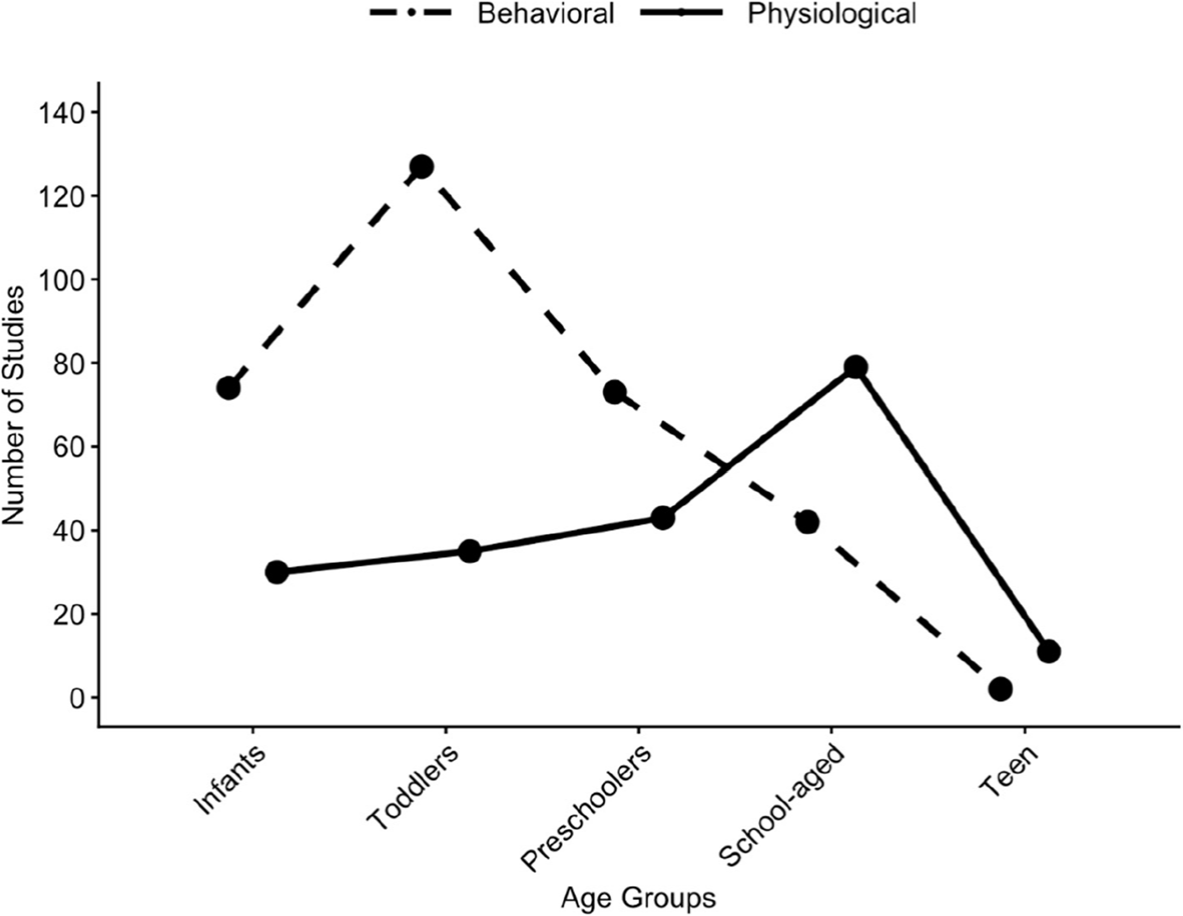
Distribution of studies by measurement type and age group This figure shows the number of studies categorized by measurement type across different age groups

A Chi-square test was conducted to compare the distribution of measurement types across age groups. Because one expected cell was below 5, Fisher's exact test was used. The results revealed a significant difference in the number of measurement types across age groups, (*p* < 0.001; *V* = 0.37). As shown in Fig. 3, studies focusing on younger age groups predominantly used behavioral observation measures, whereas studies involving older age groups favored physiological measurements.

#### Inferential results by psychometric properties

Of the 516 included studies, 314 reported at least one psychometric property. Across these, the proportion reporting reliability measure was 63.7% (95% CI [61.8, 65.6], *k* = 314), with no evidence of heterogeneity (I^2^ = 0.0%, Q(313) = 72.02, *p* = 1.000). Validity was reported in 23.7% of studies (95% CI [22.7, 24.7], *k* = 314), also with negligible heterogeneity (I^2^ = 0.0%, Q(313) = 30.27, *p* = 1.000). Objectivity was reported in 32.8% of studies (95% CI [31.2, 34.3], *k* = 314), again with no heterogeneity (I^2^ = 0.0%, Q(313) = 54.42, *p* = 1.000). Fisher’s exact tests indicated significant differences in reporting frequencies across psychometric properties, with reliability being reported more frequently than objectivity (*p* < 0.001; *V* = 0.24) and validity differing from objectivity (*p* = 0.035; *V* = 0.10), whereas no difference was observed between reliability and validity (*p* = 0.437; *V* = 0.05).

Reporting varied by measurement type. For reliability, studies on behavioral measures showed higher reporting rates (65.2%, 95% CI [63.4, 66.9], *k* = 292) compared to physiological measures (43.0%, 95% CI [32.4, 54.3], *k* = 22), and a significant subgroup effect (Q(1) = 16.76, *p* < 0.001, Cohen’s *h* = 0.45). For validity, no significant differences emerged between behavioral (23.2%, 95% CI [22.4, 24.0], *k* = 292, Cohen’s *h* = 0.18) and physiological measures (31.2%, 95% CI [21.2, 43.2], *k* = 22; Q(1) = 2.61, *p* = 0.106). For objectivity, physiological measures showed higher reporting (43.8%, 95% CI [33.5, 55.2], *k* = 22) compared to behavioral measures (32.0%, 95% CI [30.5, 33.5], *k* = 292), and a significant subgroup difference (Q(1) = 5.17, *p* = 0.023, Cohen’s *h* = 0.25).

Across age groups, the proportion of studies reporting reliability ranged from 56.4% to 75.0%, with significant subgroup differences (Q(4) = 14.40, *p* = 0.006, Cohen’s *h* = 0.40), with the highest proportion observed in the youngest children (Appendix, Table 9). For validity, the proportion of studies reporting this property ranged from 21.7% to 28.3% across age groups, with significant subgroup differences (Q(4) = 10.23, *p* = 0.037, Cohen’s *h* = 0.15). Validity was only assessed in studies including toddlers, preschoolers, and school-aged children (Appendix, Table 9). Objectivity was reported in 25.0% to 33.8% of the studies across age groups, with no significant subgroup differences (Q(4) = 1.39, *p* = 0.846, Cohen’s *h* = 0.19). Reporting was most frequent in studies including toddlers (Appendix Table 9).

Pooled effect estimates for specific reliability or validity indices were not calculated, as the available data were limited to the subset of studies reporting these psychometric indices (61% of all 516 studies). Because the reporting of such indices was not systematic across the literature, the resulting dataset may not have been fully representative of the broader evidence base. Consequently, any pooled estimates derived from this subset may have been biased and could potentially overestimate the true effects.

To examine whether studies reporting psychometric properties differed from those that did not, nonparametric and categorical analyses were conducted. Studies reporting psychometric properties had significantly larger sample sizes (*Mdn* = 94) than those that did not (*Mdn* = 73), *W* = 26,420, *p* = 0.002, *r* = 0.14. A small difference was also observed for the proportion of male participants, *W* = 19,980, *p* = 0.019, *r* = 0.11. Consistent with the meta-analytic subgroup analyses, reporting differed markedly by measurement type, χ^2^(1) = 330.31, *p* < 0.001, *V* = 0.80, and by age groups, *p* = 0.001, *V* = 0.39. No consistent differences were observed for ethnicity variables (all *ps* > 0.05). Reporting also differed by study design, *p* = 0.001 (Fisher’s exact test), with a small effect (*V* = 0.19), whereas no differences were found for child characteristics (*p* = 0.287, *V* = 0.22), language (*p* = 0.511, *V* = 0.09) or risk-of-bias ratings (*p* = 0.607, *V* = 0.05).

#### Sensitivity analyses by risk-of-bias ratings

Sensitivity analyses were conducted to examine the robustness of the findings across risk-of-bias ratings. These analyses were based on 288 studies, as 26 studies included both measurement types but only one study-level risk-of-bias rating. Across these studies, the proportion of studies reporting reliability was 64.5% (95% CI [62.6, 66.4], *k* = 288), with no evidence of heterogeneity (I^2^ = 0.0%, Q(287) = 62.60, *p* = 1.000). Results were consistent across risk-of-bias groups, with proportions of 70.8% for high risk (95% CI [65.2, 75.9], *k* = 17), 64.5% for low risk (95% CI [61.8, 67.1], *k* = 151), and 63.6% for moderate risk (95% CI [60.5, 66.6], *k* = 120). No significant between-group differences were observed, Q(2) = 5.81, *p* = 0.055, Cohen’s *h* = 0.15. The overall proportion of studies reporting validity was 23.8% (95% CI [22.8, 24.8], *k* = 288), again with no heterogeneity (I^2^ = 0.0%, Q(287) = 25.34, *p* = 1.000). Subgroup estimates were similar across risk levels: 24.8% for high risk (95% CI [21.2, 28.6], *k* = 17), 23.9% for low risk (95% CI [22.5, 25.3], *k* = 151), and 23.5% for moderate risk (95% CI [22.0, 24.9], *k* = 120). No significant subgroup differences were found, Q(2) = 0.52, *p* = 0.772, Cohen’s *h* = 0.03. The overall proportion of studies reporting objectivity was 31.9% (95% CI [30.4, 33.5], *k* = 288), with no heterogeneity (I^2^ = 0.0%, Q(287) = 45.76, *p* = 1.000). Estimates were comparable across risk groups: 26.9% for high risk (95% CI [22.5, 31.9], *k* = 17), 31.7% for low risk (95% CI [29.6, 33.8], *k* = 151), and 33.1% for moderate risk (95% CI [30.6, 35.6], *k* = 120). Between-group differences were not statistically significant, Q(2) = 5.36, *p* = 0.069, Cohen’s *h* = 0.13.

## Discussion

This review provides an in-depth overview of the qualitative and quantitative state of the existing behavioral observations and physiological measures of ER in children and adolescents. We systematically synthesized the existing evidence to summarize definitions and models of ER, as well as ER measures and their psychometric quality.

### Definitions and models of emotion regulation

Based on the theoretical embedding of the included studies, it can be concluded that the majority (51%) did not explicitly rely on a defined theoretical model or a formal definition of ER. This finding is consistent with a recent systematic review showing that most ER studies are not based on a specific theoretical framework [169]. The present review extends this observation to the literature on child ER.

Among the studies that cited an ER definition and/or tested an ER model, Thompson’s definition [233] was the most commonly used (39%), followed by the Transactional Model (59%; [211]). The prominence of Thompson’s definition is consistent with the broader literature, which identifies it as the most frequently referenced framework in both adult and child ER research [59, 60, 84]. Thompson’s definition conceptualizes ER as a goal-oriented process influenced by situational and individual contexts, incorporating an evaluative component of emotional reactions [233]. Similarly, the Transactional Model emphasizes the development of ER through dynamic interactions between the child and the environment [211]. These perspectives converge in viewing ER as a multifactorial, context-dependent, and functionally oriented process. Developmental models further specify how these processes unfold over time. In particular, ER is understood to emerge through interactional processes, gradually shifting from interpersonal (co-regulatory) to intrapersonal (self-regulatory) forms [143]. This developmental progression aligns with both the functionalist perspective emphasized by Thompson and the interactional focus of the Transactional Model, highlighting that goal-directed regulation is initially scaffolded within social interactions and becomes increasingly internalized across development.

The results partially support Hypothesis 1, showing that studies differ in their underlying ER definitions and models. However, the most frequently cited frameworks follow a functionalist perspective rather than the one- or two-factor concepts proposed by [48]. Additionally, a substantial proportion of studies do not rely on any explicit theoretical framework, underscoring the diversity of approaches and the absence of a single, unified model or definition of ER in the literature.

### Emotion regulation measurements

#### Type and number of measurements

In confirmation of the second hypothesis, it can be concluded that the type of ER measurement depends on the age of the children. Significantly more studies on behavioral observations were found in younger children than in older children, while significantly more studies on physiological measurements were conducted in older children than in younger children. This trend may partly reflect that behavioral observations are considered the gold standard for assessing ER in younger children, as they provide a direct and reliable way to capture emotional responses [219]. In contrast, physiological measures are more difficult to implement in younger children due to developmental variability in physiological systems, increased susceptibility to movement artifacts, and challenges with compliance during sensor placement and experimental procedures [69, 198, 262]. These factors can complicate both data acquisition and interpretation in infants and toddlers.

Regarding behavioral observations, the most commonly used tasks to induce negative emotions with good quality criteria were the Still-Face-Paradigm, the Lab-TAB tasks (Attractive Toy Behind Barrier, Attractive Toy in Transparent Box, High Chair and Spider Task), and variations of the Disappointment and Delay of Gratification Tasks. This finding is consistent with another systematic review, which describes these tasks as commonly used to elicit emotions in infants and preschoolers [210]. Notably, most behavioral observation studies used study-specific coding schemes rather than relying on established coding manuals. This limits comparability across studies and complicates the synthesis of findings, for example for meta-analyses and is at odds with the principles of cumulative science.

For physiological measurements, RSA and EEG are most frequently examined, along with some evidence supporting their validity. This finding aligns with previous research indicating, that RSA is a well-established indicator of parasympathetic activation associated with ER [21, 22]. It is also consistent with the adult and developmental literature, which indicates that EEG measures such as ERP, LPP and frontal EEG asymmetry are considered important neural correlates of emotional and ER processes [25, 131, 189]. However, this review did not find vagal tone to be commonly examined as an indicator of parasympathetic activation, despite its prominence in literature primarily based on adult samples [194]. Vagal tone is typically operationalised through HRV, often referred to as cardiac vagal tone [159]. HRV was frequently assessed in the reviewed studies; however, it was mainly used as a general physiological measure rather than being explicitly conceptualised or interpreted as an index of parasympathetic or vagal activity, as commonly done in adult research [159, 218]. This difference may reflect broader methodological and conceptual inconsistencies in the measurement and interpretation of HRV, including variability in the selection of HRV parameters, differences in theoretical conceptualisation, and inconsistencies in how parasympathetic activity is interpreted, as discussed in previous studies [8, 159, 218].

According to the number of used measurements*,* most studies employed a single ER measurement method (90%) rather than multiple methods. This finding is consistent with the third hypothesis, which stated that single-method assessments of ER are more common than multimethod approaches. Similar results were reported in a previous review by [2]. However, given the complex and multifaceted nature of ER, both the findings of the present review and prior literature emphasize the importance of using multiple measurement methods to comprehensively assess ER [3, 102].

#### Elicited emotions

Studies employing physiological measures frequently used tasks that elicited non-specific or mixed emotional responses. This approach may reflect the primary aim of many of these studies, which is to assess general autonomic reactivity or regulatory capacity rather than emotion-specific physiological responses. In particular, physiological research often focuses on capturing tonic (baseline) and phasic (task-related) changes in autonomic activity, as recommended in HRV research guidelines [159, 190], rather than isolating responses to discrete emotional states. From a theoretical perspective, this approach aligns with a broader conceptualization of ER, which encompasses not only deliberate, strategy-based processes but also more automatic and physiological forms of regulation [19, 144]. Within this broader framework, physiological measures may primarily capture implicit and less volitional regulatory processes, in contrast to the more explicit, strategy-based forms of ER typically assessed in behavioral observations.

In addition, across both types of measurements, a larger proportion of studies examined ER in response to negative emotions compared to positive emotions. This represents a critical limitation, as it neglects a key aspect of emotional experiences, which is included in the definition of ER as targeting both positive and negative emotions and involving the maintenance, enhancement, or reduction of emotional arousal [235]. The regulation of positive emotions remains comparatively understudied [109], despite its theoretical and clinical relevance. It complements the regulation of negative emotions, which has traditionally dominated psychopathology research [4]. To fully understand ER as a comprehensive mechanism, particularly in the context of mental disorders and mental health, it is essential to consider the entire emotional spectrum [50, 239].

#### Psychometric properties

The meta-analytic findings highlight substantial gaps in the reporting of psychometric properties across the literature. A considerable proportion of studies (39%) did not report any psychometric information, meaning that the meta-analytic results were necessarily based on a subset of studies. The analyses showed no evidence of substantial between-study heterogeneity (*I*^*2*^ = 0% across analyses), suggesting consistent reporting patterns among studies reporting psychometric properties. However, most studies contributed only a small number of psychometric indicators, resulting in wide study-level confidence intervals and limited power to detect between-study variability. At the same time, reporting patterns were similar across study subgroups, with most studies reporting only a limited set of psychometric indices and reliability being reported more consistently than validity. This finding supports the third hypothesis, which posited that reliability would be reported more frequently than validity. It is also consistent with research in other fields, such as health education, where reliability is more commonly assessed than validity in scales and questionnaires [20]. One possible explanation is that reliability is generally easier to assess than validity, particularly for observational or physiological measures, which are often more complex and resource-intensive [14, 150].

Differences between behavioral and physiological measures further suggest that methodological characteristics influence which psychometric properties are prioritized. Studies using behavioral observation reported significantly more reliability measures than studies using physiological methods, with the subgroup difference corresponding to a small-to-moderate effect. This likely reflects the reliance on subjective coding in behavioral observations, where interrater reliability is essential to ensure consistency [14, 150]. However, physiological measures also involve coding and subjective analytic decisions (e.g., artifact correction, segment selection), indicating that reliability assessment is equally relevant in these contexts. Notably, nearly all reported reliability indices were based on interrater reliability (92%) or internal consistency (8%), while no study assessed test–retest reliability. This represents a critical gap, as test–retest reliability is essential for determining the temporal stability of ER measures [250]. Evidence on reliability and validity, particularly discriminant validity and test–retest reliability, remains largely derived from adult populations and questionnaire-based measures [26, 28, 33, 224], with comparatively limited data available for behavioral observations and physiological measures in children.

Regarding validity, the most frequently reported forms were convergent (44%) and concurrent validity (28%), whereas discriminant validity was assessed in only one study (4%), highlighting a substantial gap. This is particularly concerning given the conceptual overlap of ER with related constructs such as temperament and coping [61, 247], making discriminant validity essential for clarifying construct boundaries [46, 165]. Reporting did not differ significantly between studies using behavioral observations and physiological measures. Objectivity was reported only occasionally across both measurement types, consistent with previous findings in the ER literature [5]. Although objectivity was reported somewhat more frequently in studies using physiological measures than behavioral observations, the associated effect size was small.

Age-related variation was observed for reliability and validity, but not for objectivity, suggesting developmental gaps in the assessment of psychometric properties, particularly in studies involving older children and adolescents. Although statistically significant, the subgroup differences corresponded to small effect sizes for validity and small-to-moderate effect sizes for reliability, indicating only modest differences across age groups.

Whether psychometric properties were reported appeared to vary systematically by measurement type and developmental stage, as indicated by moderate to large effect sizes, rather than reflecting unsystematic differences across studies or other study characteristics that showed only small effects. Sensitivity analyses further indicated that these patterns were stable across risk-of-bias ratings, suggesting that the reporting of psychometric properties was not systematically related to overall study quality.

Taken together, the strong emphasis on reliability, combined with limited reporting of validity and substantial non-reporting overall, highlights a systematic imbalance in current practices. This has important implications for the interpretability of findings, as reliability alone does not ensure that constructs are adequately captured. Strengthening reporting standards, particularly with regard to validity, will be essential to improve comparability across studies and enhance the cumulative value of research on ER across developmental stages.

### Strengths

A key strength of this review lies in its systematic approach, which overcomes the limitations of previous reviews by applying minimal restrictions on inclusion criteria (e.g. [2, 60, 146, 260]). Notably, this review imposes no language restrictions, making a significant contribution to overcoming the WEIRD bias. By including studies in all languages, the review enhances the diversity of ethnicities represented in the sample, offering a more comprehensive overview of the existing literature. Additionally, this is the first review to systematically evaluate and extract ER measurement methods for children and adolescents based on their psychometric properties. It also stands as the first study to explore the theoretical embedding of ER in the literature for children and adolescents, providing an overview of the most cited ER definitions and models in this field. Taken together, these findings on ER measurements, their psychometric properties, and their theoretical foundations represent a significant contribution to advancing the assessment of ER. Furthermore, they simplify the process of selecting ER measurements for future studies and encourage ongoing research into the psychometric properties of these measures.

### Limitations

However, this review has several limitations that should be considered when interpreting the findings. First, the restricted age range, which was necessary for feasibility, limits the generalizability of our conclusions regarding ER measurements in adolescents, as the focus is primarily on younger age groups. Second, the review excluded questionnaire-based methods to enhance readability, meaning that our analysis of psychometric properties is confined to behavioral observation and physiological measurement methods, which may omit relevant insights from self-report instruments. Third, the available data on psychometric properties were limited to a subset of studies, as a considerable proportion of studies did not report any psychometric information. Consequently, the analyses of psychometric properties are based on a selective subset of the literature, which may not be fully representative of all studies. Our analyses indicate systematic differences between studies that report psychometric properties and those that do not, particularly by measurement type and age groups, suggesting that reporting reflects study characteristics rather than consistent measurement standards. Fourth, the characteristics of the underlying study populations introduce further constraints. Consistent with the WEIRD problem in psychological science [213], most studies provided limited information on ethnicity or household income (Table 1). When reported, samples were predominantly White, and few studies included participants from low-income families (Table 1). Because ER strategies vary across cultural and socioeconomic contexts, the lack of diverse samples limits the generalizability and applicability of the findings [64, 68, 200, 201]. Fifth, the clinical scope of the included studies is narrow. Most investigations focused on healthy children, with only a small proportion examining psychological disorders (Table 1). Internalizing disorders, which often co-occur with externalizing disorders, were underrepresented despite their prevalence and early onset, particularly in the case of anxiety disorders [24, 151]. Consequently, the current evidence provides a limited view of the psychometric quality and applicability of ER measurement methods in clinical populations.

Taken together, these limitations indicate that while the review provides a systematic overview of ER measurement methods and their psychometric properties, the generalizability of the findings to broader, more diverse, or clinical populations remains constrained.

### Implications for future research

This review highlights several gaps in the literature on ER measurements, suggesting directions for future research. One important consideration is the need to more carefully specify the underlying definitions and theoretical models of ER being examined. Clearer conceptual grounding may help guiding the selection, application, and interpretation of measurement methods. In this context, further efforts toward greater consistency in measurement approaches may be beneficial. For example, more systematic alignment in the choice of behavioral observation tasks, physiological indicators, and reporting practices supports comparability across studies. This review represents a first step toward a more systematic assessment of ER using behavioral and physiological measures, providing an overview of available methods and their psychometric properties and thereby facilitating the selection of measures for future studies. In particular, more consistent reporting of psychometric properties, such as reliability, including test–retest estimates, and different forms of validity, especially discriminant validity, strengthens the interpretability of findings. The currently limited reporting of discriminant validity and test–retest reliability restricts conclusions about the distinctiveness and stability of ER constructs. This is particularly relevant in developmental research, where ongoing developmental changes influence both the expression and measurement of ER, and where more robust psychometric data supports the development of predictive models for mental health outcomes. The meta-analytic findings further suggest that the reporting of psychometric properties is associated with study characteristics rather than consistent reporting standards. Future research should therefore adopt more standardized measurement and reporting practices, including the routine reporting of reliability and validity indices. In addition, most behavioral observation and physiological measurement studies have predominantly focused on negative emotions, with comparatively less attention given to the regulation of positive emotions. Expanding research in this area is important, as positive emotions are a central aspect of ER, linked to mental health and well-being, and are relevant in social relationships [50, 80]. Beyond the characteristics of individual measures, combining multiple ER assessment methods provides a more comprehensive perspective on regulatory processes. Likewise, most studies focus on healthy children or externalizing disorders, leaving evidence on internalizing and other clinically relevant conditions comparatively sparse. Addressing these gaps enhances the applicability of ER measurement methods and deepens our understanding of emotional development and psychopathology.

Continued attention to psychometric quality, conceptual clarity, and multimethod approaches will contribute to a more cumulative and comparable evidence base, encompassing both negative and positive emotional processes.

## Supplementary Information

Supplementary Material 1.

## Data Availability

The study was pre-registered at PROSPERO [https://www.crd.york.ac.uk/PROSPERO/view/CRD42021266452] All data code, and codebooks associated with this study are publicly available via PsychArchives at: (Dataset Link: 10.23668/psycharchives.22185) (Code Link: 10.23668/psycharchives.22186).

## Appendix

### File 1

**Table:** PRISMA 2020 Checklist

**Section and Topic **	**Item #**	**Checklist item **	**Location where item is reported **
**TITLE **			
Title	1	Identify the report as a systematic review.	p. 1
**ABSTRACT **			
Abstract	2	See the PRISMA 2020 for Abstracts checklist.	Appendix, File 3
**INTRODUCTION **			
Rationale	3	Describe the rationale for the review in the context of existing knowledge.	p. 6
Objectives	4	Provide an explicit statement of the objective(s) or question(s) the review addresses.	p. 7
**METHODS **			
Eligibility criteria	5	Specify the inclusion and exclusion criteria for the review and how studies were grouped for the syntheses.	pp. 8-9
Information sources	6	Specify all databases, registers, websites, organisations, reference lists and other sources searched or consulted to identify studies. Specify the date when each source was last searched or consulted.	p. 8
Search strategy	7	Present the full search strategies for all databases, registers and websites, including any filters and limits used.	p. 9; Appendix, File 3
Selection process	8	Specify the methods used to decide whether a study met the inclusion criteria of the review, including how many reviewers screened each record and each report retrieved, whether they worked independently, and if applicable, details of automation tools used in the process.	pp. 9-11, Fig. 2
Data collection process	9	Specify the methods used to collect data from reports, including how many reviewers collected data from each report, whether they worked independently, any processes for obtaining or confirming data from study investigators, and if applicable, details of automation tools used in the process.	pp. 9-11
Data items	10a	List and define all outcomes for which data were sought. Specify whether all results that were compatible with each outcome domain in each study were sought (e.g. for all measures, time points, analyses), and if not, the methods used to decide which results to collect.	pp. 9-11
	10b	List and define all other variables for which data were sought (e.g. participant and intervention characteristics, funding sources). Describe any assumptions made about any missing or unclear information.	pp. 9-11
Study risk of bias assessment	11	Specify the methods used to assess risk of bias in the included studies, including details of the tool(s) used, how many reviewers assessed each study and whether they worked independently, and if applicable, details of automation tools used in the process.	pp. 9-11
Effect measures	12	Specify for each outcome the effect measure(s) (e.g. risk ratio, mean difference) used in the synthesis or presentation of results.	pp. 9-11
Synthesis methods	13a	Describe the processes used to decide which studies were eligible for each synthesis (e.g. tabulating the study intervention characteristics and comparing against the planned groups for each synthesis (item #5)).	pp. 8-9
	13b	Describe any methods required to prepare the data for presentation or synthesis, such as handling of missing summary statistics, or data conversions.	pp. 8-9; p. 13
	13c	Describe any methods used to tabulate or visually display results of individual studies and syntheses.	p. 18
	13d	Describe any methods used to synthesize results and provide a rationale for the choice(s). If meta-analysis was performed, describe the model(s), method(s) to identify the presence and extent of statistical heterogeneity, and software package(s) used.	pp. 8-9; p. 18
	13e	Describe any methods used to explore possible causes of heterogeneity among study results (e.g. subgroup analysis, meta-regression).	-
	13f	Describe any sensitivity analyses conducted to assess robustness of the synthesized results.	p. 10
Reporting bias assessment	14	Describe any methods used to assess risk of bias due to missing results in a synthesis (arising from reporting biases).	-
Certainty assessment	15	Describe any methods used to assess certainty (or confidence) in the body of evidence for an outcome.	-
**RESULTS **			
Study selection	16a	Describe the results of the search and selection process, from the number of records identified in the search to the number of studies included in the review, ideally using a flow diagram.	Fig. 2
	16b	Cite studies that might appear to meet the inclusion criteria, but which were excluded, and explain why they were excluded.	p. 13
Study characteristics	17	Cite each included study and present its characteristics.	Table 1, Appendix: Table 1-4, Suppl. Material Table 1+ 2
Risk of bias in studies	18	Present assessments of risk of bias for each included study.	Risk of Bias ratings in shared data
Results of individual studies	19	For all outcomes, present, for each study: (a) summary statistics for each group (where appropriate) and (b) an effect estimate and its precision (e.g. confidence/credible interval), ideally using structured tables or plots.	Table 1, Appedix: Table 1-4, p. 13
Results of syntheses	20a	For each synthesis, briefly summarise the characteristics and risk of bias among contributing studies.	pp. 13-16
	20b	Present results of all statistical syntheses conducted. If meta-analysis was done, present for each the summary estimate and its precision (e.g. confidence/credible interval) and measures of statistical heterogeneity. If comparing groups, describe the direction of the effect.	pp- 18-26
	20c	Present results of all investigations of possible causes of heterogeneity among study results.	pp. 15-16, pp. 26-27
	20d	Present results of all sensitivity analyses conducted to assess the robustness of the synthesized results.	-
Reporting biases	21	Present assessments of risk of bias due to missing results (arising from reporting biases) for each synthesis assessed.	-
Certainty of evidence	22	Present assessments of certainty (or confidence) in the body of evidence for each outcome assessed.	-
**DISCUSSION **			
Discussion	23a	Provide a general interpretation of the results in the context of other evidence.	pp. 27-32
	23b	Discuss any limitations of the evidence included in the review.	pp. 33-34
	23c	Discuss any limitations of the review processes used.	pp. 33-34
	23d	Discuss implications of the results for practice, policy, and future research.	pp. 34-35
**OTHER INFORMATION**			
Registration and protocol	24a	Provide registration information for the review, including register name and registration number, or state that the review was not registered.	p. 8
	24b	Indicate where the review protocol can be accessed, or state that a protocol was not prepared.	p. 8
	24c	Describe and explain any amendments to information provided at registration or in the protocol.	pp. 8-9
Support	25	Describe sources of financial or non-financial support for the review, and the role of the funders or sponsors in the review.	Title page
Competing interests	26	Declare any competing interests of review authors.	Title page
Availability of data, code and other materials	27	Report which of the following are publicly available and where they can be found: template data collection forms; data extracted from included studies; data used for all analyses; analytic code; any other materials used in the review.	p. 8

### File 2

**Table:** PRISMA 2020 for abstracts checklist

Section and Topic	Item #	Checklist item	Reported (Yes/No)
**TITLE **			
Title	1	Identify the report as a systematic review.	yes
**BACKGROUND **			
Objectives	2	Provide an explicit statement of the main objective(s) or question(s) the review addresses.	yes
**METHODS **			
Eligibility criteria	3	Specify the inclusion and exclusion criteria for the review.	yes
Information sources	4	Specify the information sources (e.g. databases, registers) used to identify studies and the date when each was last searched.	yes
Risk of bias	5	Specify the methods used to assess risk of bias in the included studies.	yes
Synthesis of results	6	Specify the methods used to present and synthesise results.	yes
**RESULTS **			
Included studies	7	Give the total number of included studies and participants and summarise relevant characteristics of studies.	yes
Synthesis of results	8	Present results for main outcomes, preferably indicating the number of included studies and participants for each. If meta-analysis was done, report the summary estimate and confidence/credible interval. If comparing groups, indicate the direction of the effect (i.e. which group is favoured).	yes
**DISCUSSION **			
Limitations of evidence	9	Provide a brief summary of the limitations of the evidence included in the review (e.g. study risk of bias, inconsistency and imprecision).	yes
Interpretation	10	Provide a general interpretation of the results and important implications.	yes
**OTHER **			
Funding	11	Specify the primary source of funding for the review.	yes
Registration	12	Provide the register name and registration number.	yes

### File 3

Search Terms:

“emotion* regulat*” OR “emotion* management” OR “affect* regulat*”

If there is no age selection by the searching machine, we add:

AND“child*” OR “infan*” OR “neonatal” OR “newborn” OR “bab*” OR “toddler” OR“preschooler” OR “pre-school*” OR “schoolchildren” OR “pupils” OR “youth” OR“young” OR “teen” OR “kid*” OR “juvenile” OR “adolescen*”

**Table 2:** Number of studies according to their language

Language	Behavioral Observation Studies		Physiological Measurement Studies	
	*n*	%	*n*	%
English	311	97.8	197	99.5
Spanish	3	0.9	0	0
German	2	0.6	1	0.5
French	1	0.3	0	0
Italian	1	0.3	0	0

**Table 3:** Number of studies according their publication type

Publication Type	Behavioral Observation Studies		Physiological Measurement Studies	
	*n*	%	*n*	%
Peer reviewed	308	96.9	196	99
Non peer reviewed	4	1.3	2	1
Dissertation	6	1.9	0

**Table 4:** Number of studies according to their study designs

Study Design	Behavioral Observation Studies		Physiological Measurement Studies	
	*n*	*%*	*n*	*%*
Descriptive (no group comparisons)	120	37.7	97	49
Longitudinal (e.g. cohort)	112	35.2	43	21.7
Case-Control	65	20.4	47	23.7
RCT	19	6.0	11	5.6

**Table 5:** Risk of bias according to study type

Risk of Bias	Behavioral Observation Studies		Physiological Measurement Studies	
	*n*	*%*	*n*	*%*
low	163	51.3	97	49
moderate	131	41.2	93	47
high	24	7.5	8	4

**Table 6:** Definitions of ER, number of studies citing the definition and underlying concepts

Definition of ER	Underlying Concept	Number of Studies citing Definition
“Emotion regulation consists of the extrinsic and intrinsic processes responsible for monitoring, evaluating, and modifying emotional reactions, especially their intensive and temporal features, to accomplish one’s goals.” [233]	Functionalist view	274
“Emotion regulation refers to the processes by which individuals influence which emotions they have, when they have them, and how they experience and express these emotions. Emotion regulatory processes may be automatic or controlled, conscious or unconscious, and may have their effects at one or more points in the emotion generative process.” [117, 118]	Functionalist view; Physiological perspectives ER as unconscious and conscious process	114
“Emotion regulation refers to processes that serve to manage emotional arousal and support adaptive social and non-social responses” [45]	Functionalist view; Physiological perspectives	79
“Behaviors, skills, and strategies, that serves to modulate, inhibit, and enhance emotional experiences to accomplish one’s goals” [41]	Functionalist view	31
“Emotion regulation refers to modifying the intensity, duration,and valence of an emotion.“ [60, 117]	One Factor Concept	20
“Emotion regulation may be conceptualized as involving the (a) awareness and understanding of emotions, (b) acceptance of emotions, (c) ability to control impulsive behaviors and behave in accordance with desired goals when experienced negative emotions, and (d) ability to situationally appropriate emotion regulation strategies flexibly to modulate emotional responses as desired in order to meet individual goals and situational demands.” [114]	Functionalist view	13
“Emotion regulation encompasses a broad set of behaviors that permit a child to dampen, intensify or maintain the magnitude and duration of an emotional response.” (Gross 2007)	Functionalist view	12
“Processes used to manage and change if, when, and how (e.g., how intensely) one experiences emotions and emotion-related motivational and physiological states, as well as how emotions are expressed behaviorally” (Eisenberg et al., 2007)	Multifactorial perspective	12
“From my perspective, emotion regulation requires the activation of a goal to up- or down-regulate either the magnitude or duration of the emotional response” (Gross et al., 2011)	One Factor Concept	12
“Emotion regulation has been defined as the capacity to manage and control emotional states in order to adapt to different contexts” [73]	One Factor Concept; Functionalist view	11
“Emotion regulation involves children's ability to selectively maintain and alter emotional experiences and expressions” (Shields. & Cicchetti, 1998)	One Factor Concept	10
"We define emotion regulation as the intra- and extra organismic factors by which emotional arousal is redirected, controlled, modulated, and modified to enable an individual to function adaptively in emotionally arousing situations." [53]	One Factor Concept, Functionalist view; Physiological perspectives	9
“Emotion regulation is the modification of any process in the system that generates emotion or its manifestation in behavior. The processes that modify emotions come from the same set of processes as those that are involved in emotion in the first place.” [48]	One Factor Concept	9
“The capacity to modulate one’s emotional arousal such that an optimal level of engagement with one’s environment is fostered” (Cicchetti et al., 1995)	One Factor Concept; Functionalist view; Physiological perspectives	8
"The ability to control own emotion, regulating when and how it appears. It implies monitoring, evaluating and modifying emotional reactions to achieve the proposed objectives and goals" [118]	One Factor Concept, Functionalist view	8
“Self-regulation, which is broadly defined as the ability to regulate our own arousal, emotion, and behavior” (Shonkoff 2000)	Self-Regulation	7
“Emotion regulation refers to those behaviors that serve to modulate arousal” [208]	Physiological perspectives	6
"Two biologically based components of temperament: reactivity and self-regulation…self-regulation is any process that serves to modulate reactivity in order to keep the arousal within an individually optimal range." (Rothbart 1989)	Physiological perspectives; ER as component of temperament Self-Regulation	6
"Emotion regulation is the process via which an emotional state is modified either in that is it decreased or attenuated or that it is increased or strengthened” [60]	One Factor Concept	5
“Emotion regulation is thus a conceptual rubric for a diffuse network of allied processes, from within and outside the child that contribute to emotional self-management. Consequently, the nature of developmental change in emotion regulation is also multifaceted” [234]	Multifactorial developmental perspective; Functionalist view	5
“We define emotion-related self-regulation as the process of initiating, avoiding, inhibiting, maintaining, or modulating the occurrence, form, intensity, or duration of internal feeling states, emotion-related physiological, attentional processes, motivational states, and/or the behavioral concomitants of emotion in the service of accomplishing affect-related biological or social adaptation or achieving individual goals.” [84]	Self-regulation; Multifactorial perspective	5
“Emotion regulation is a complex process involving one’s physiological, experiential, and behavioral expressions of an emotion and the ability to modulate the speed and intensity of escalation and deescalation of that emotion” [36, 261]	Multifactorial perspective; Functionalist view	5
“Emotion regulation is the ability to manage one's subjective experience of emotion, especially its intensity and duration, and to manage strategically one's expression of emotion in communicative contexts" [212]	Functionalist view	4
"Emotion regulation is subject to a variety of definitions and interpretations, it is often conceived as being the processes or strategies that are used to manage emotional arousal so that successful inter-personal functioning is possible.” [45]	Functionalist view; Physiological perspectives	4
“Ability to (a) inhibit inappropriate behavior related to strong negative or positive affect, (b) self-soothe any physiological arousal that the strong affect has induced, (c) refocus attention, and (d) organize for coordinated action in the service of an external goal.” [113]	Functionalist view	4
"We define the process of emotion regulation as the manipulation in self or others of either emotion antecedents or one or more of the components of an emotional response" [122]	One Factor Concept	3
Focus on ER as “automatic (largely unconscious) regulatory processes such as those involved in overlearned habits or culturally transmitted norms” [173]	ER as unconscious process	3
"Self-regulation is defined as processes functioning to modulate this reactivity, including behavioral patterns of approach and avoidance, attentional orientation and selection“ [207]	Self-Regulation	2
“Emotion Regulation (ER) is the ability to modify arousal and emotional reactivity to achieve goals and maintain adaptive behaviors” [23]	One Factor Concept; Physiological perspectives; Functionalist view	2
“We therefore conceptualize ER as a complex system that involves dynamic regulation of behavioral (e.g.,direct displays of emotions) and biological (e.g., HPA axis) responses to internal and external stimuli” [120]	Multifactorial perspective; Physiological perspectives	2
"We propose that emotion regulation is one dimension of a multileveled self-regulatory system that governs children's behavior" [42]	Self-Regulation	1
"The notion that emotions can be regulated as an adaptive means, a strategy, to achieve one's goals within a given context" [51]	One Factor Concept; Functionalist view	1
ER “involves varied knowledge and skills, including (a) knowing that one’s emotions can be controlled and (b)awareness of possible strategies and associated advantages/limitations”	ER as conscious process	1
“ER as a subset of coping, since the stressors and their regulation relate to the emotional experience” [103] (translated by the first author)	ER as a concept of coping	1
“Emotion regulation is not a single behavior, but a varied collection of processes and strategies’’ [105]	Multifactorial perspective	1
"ER refers to the behaviors, skills, and strategies that modulate affective arousal in a way that facilitates adaptive functioning” [41]	Functionalist view; One Factor Concept; Physiological perspectives	1
Emotion regulation refers to children's ability to appropriately regulate their emotions (e.g., fear, anxiety, joy) as well as the behaviors influenced by such emotional reactions.”	Two Factor Concept	1
“ER is defined as a set of processes that serve to modulate, maintain, or enhance the intensity and valence of emotional experiences” [199]	One Factor Concept	1
“ER comprises the adequate management of emotional activation to achieve effective social functioning. It involves initiating, maintaining, modulating, or changing the occurrence, intensity, or duration of internal feeling states and emotion-related physiological reactions” [203]	Functionalist view Physiological perspective	1
“The concept of infant emotion regulation refers to the child´s ability or willingness to calm down or settle and adjust to physiological or environmental conditions or stimulation” [228]	Functionalist view Physiological perspective	1
"Emotion regulation involves the use of behavioral and cognitive strategies to change the duration and intensity of an emotion” [240]	One Factor Concept	1
"Emotion regulation involves the process of regulating emotional arousal and emotional expressions flexibly according to environmental demands” [252]	Functionalist view	1
“Emotion regulation is a process that integrates physiological, cognitive, and behavioral components" [70]	Multifactorial perspective	1
"Taken together, these various positions on defining ER overlap considerably and ultimately agree that the concept is complex, involves multiple systems (genetic, neurophysiological, behavioral, psychological), becomes more integrated with development, and must be considered with regard to the individual’s goals and the social and cultural context within which ER develops" [229]	Multifactorial perspective; Functionalist view	1
"Implicit emotion regulation may be defined as any process that operates without the need for conscious supervision or explicit intentions, and which is aimed at modifying the quality, intensity, or duration of an emotional response. Implicit emotion regulation can thus be instigated even when people do not realize that they are engaging in any form of emotion regulation and when people have no conscious intention of regulating their emotions" [127]	ER as unconscious process	1
"Emotion regulation is any process that influences the onset, offset, magnitude, duration, intensity, or quality of one or more aspects of emotion response" [184]	One Factor Concept	1
“Emotional regulation skills comprise the extent to which individuals react to emotion-inducing events and are then able to return to a state of equilibrium” [29]	Concept of homeostasis	1
“Emotion regulation is a set of complex skills which are necessary for effective adaptation and successful social interactions in everyday life.” [170]	Functionalist view	1
“Emotion regulation is the ability to modulate the valence and intensity of emotions in service of a goal.” [168]	One Factor Concept; Functionalist view	1

**Table 7:** Models of ER, number of studies citing the model, brief description of model concept

Model of ER	Concept	Number of Studies citing Model
The Transactional Model [211]	Child’s regulation is product of interaction between self and other regulation	51
Process Model of Emotion Rgulation [117]	Describes ER as a process involving five stages: situation selection, situation modification, attentional deployment, cognitive change, response modulation	13
Tripartite Model of Emotion Regulation [178]	This model describes how children learn ER within the family considering three socialization components: observation, emotion-related parenting practice and emotional family climate	11
Mutual Regulation Model [108]	Describes ER as a process that involves reciprocal emotional and behavioral influence between interaction partners	3
Self-regulation intergenerational transmission model [35]	Framework that integrates prenatal, social/contextual, and neurobiological mechanisms to explain the intergenerational transmission of self-regulation	3
Heuristic model of the socialization of emotion [82]	This model describes the influence of parents' emotion socialization behaviors on children's social and emotional development (e.g. ER)	4
Polyvagal Theory in the context of emotion regulation [195, 196]	Framework for understanding the physiological basis of emotion regulation, emphasizing the dynamic parasympathetic control of cardiac activity; facilitating social engagement, focused attention, defensive responses and self-soothing	1
Developmental hierarchical-integrative model [258]	This model explains self-regulation development in early childhood as a multi-dimensional construct involving physiological, emotional and attentional processes; lower-level regulatory functions develop first, supporting the maturation of higher-order functions	1

**Table 8:** Number psychometric property measures by measurement type

Psychometric Property	Behavioral Observation Studies		Physiological Measurement Studies		Total	
	*n*	*%*	*n*	*%*	*n*	*%*
Reliability	306	59.30	13	2.52	319	61.82
Validity	20	3.87	6	1.16	26	5.03
Objectivity	92	17.83	14	2.71	106	20.16

Percentages are based on all included studies (*n* = 516)

**Table 9:** Number psychometric property measures by age groups

Broad Age Groups	Reliability		Validity		Objectivity	
	*n*	*%*	*n*	*%*	*n*	*%*
Infants	75	14.53	0	0	19	3.68
Toddler	126	24.42	10	1.94	46	8.91
Preschooler	78	15.12	6	1.16	25	4.84
School-aged children	38	7.36	10	1.37	16	3.10
Teens	2	0.39	0	0	0	0

Percentages are based on all included studies (*n* = 516)
