# Supplementary material for: Emotion regulation in children and adolescents: a systematic review and quality assessment of behavioral and physiological assessment methods and their theoretical foundations
Source: BMC Psychol. 2026 Jul 1;14:945. doi: 10.1186/s40359-026-04935-2 (PMC13321600; doi:10.1186/s40359-026-04935-2)
Supplement: Supplementary file 1 — Supplementary Material 1. [file 40359_2026_4935_MOESM1_ESM.docx]

**Supplemental Material**

Description of Supplemental Material: The supplemental material consists of Tables 1 and 2. Table 1 summarizes behavioral observation measures, and Table 2 summarizes physiological measures. Each table is divided into sections (a) through (e), representing broader age groups (a: neonates and infants, b: toddlers, c: preschoolers, d: school-aged children, e: adolescents). These broader age groups are further subdivided into narrower 12-month categories. Within each 12-month category, measures are organized according to the type of emotion examined (frustration/anger/disappointment, fear, jealousy, empathy, joy, and non-specific/mixed emotions). For each measure, information on psychometric properties and references to studies using the measure is provided.

**Table 1a**

*Behavioral observation methods of emotion regulation for neonates and infants (0 to 12 months old)*

| **Age Range (months)** | | **Task** | **Duration (sec/min/h)** | **Coding Manual** | **Reliability** | **Validity** | **Objectivity** | **Studies using this task** | **Number/ Percentage of studies using task** |
| --- | --- | --- | --- | --- | --- | --- | --- | --- | --- |
| **Neonates** | | | | | | | | | |
|  | | Observations with Neonatal Behavior Assessment Scale | 30 sec |  | Cronbach α = .66 |  | Evaluation objectivity through blinded coders | (Halligan et al., 2013) | 1 (0.3%) |
|  |  |  |  |  |  |  |  |  |  |
|  |  | Arm Restraint Task (Lab-TAB) | 30 sec |  | Cohen's κ = .75 - .76 |  |  | (Liu, C. et al., 2022) | 1 (0.3%) |
| **Infant** | | | | | | | | | |
|  | **Frustration/Anger/Disappointment Eliciting Tasks** | | | | | | | | |
| **0 to 12 months** | | Still-Face-Paradigm | 2 - 6 min | Strange Situation reunion behavior scales (Ainsworth, 1978); Self-Regulatory Behavior Scoring System (Gianino & Tronick, 2013); Coding System for Regulatory Patterns (Fuertes, 2009); Post-Still-Face Reengagement Behavior Codes (Kogan & Carter, 1996); Infant Regulatory Scoring System (IRSS; (Tronick, 1990); AFFEX system (Izard et al., 1995); Infant Self- Regulation Scheme (ISRS (Millman, 2007); Infant Reengagement Scale (Conradt & Ablow, 2010); Infant Caregiver Engagement Phases (ICEP (Reck, 2009)) | Cohen's κ = .53 - .98; ICC = .73 - .96; PPMCC = .80 - .98 |  | Implementation and evaluation objectivity through blinded assessors and coders | (Atkinson et al., 2021; August et al., 2017; Bagne, 2021; Bolten et al., 2013; Braungart-Rieker et al., 2001; Braungart-Rieker et al., 1998; Conradt & Ablow, 2010; Ding et al., 2024; Ekas et al., 2013; Feldman, 2015; Fuertes et al., 2021; Ginnell et al., 2022; Heron-Delaney et al., 2016; Kogan & Carter, 1996; Maclean et al., 2009; Manian & Bornstein, 2009; Shapiro et al., 1998; Sullivan et al., 2015; Yaari et al., 2018) | 18 (5.6%) |
|  |  |  |  |  |  |  |  |  |  |
|  |  | Arm-Restraint Procedure (Lab-TAB) | 30 sec - 2 min. | Strategies of Emotional Regulation Scale (Faure et al., 2013); Lab-TAB manual (Goldsmith, 1999) | Cohen's κ = .47 - .99; ICC = .93 -.95; Cronbach α = .77 |  | Evaluation objectivity through blinded coders | (Buss & Goldsmith, 1998; Calkins et al., 2002; Dollar et al., 2020; Eiden et al., 2009; Faure et al., 2013; Feldman, 2015; Granat et al., 2017; Gratz et al., 2014; Habersaat et al., 2013; Halligan et al., 2013; Perry, Calkins & Bell, 2016; Stifter & Spinrad, 2002; Ursache et al., 2013; Wu & Feng, 2020; Wu & Gazelle, 2021) | 14 (4.4%) |
|  |  |  |  |  |  |  |  |  |  |
|  |  | Attractive Toy behind Barrier (Lab-TAB) | 30 sec - 2 min | Strategies of Emotional Regulation Scale (Faure et al., 2013); Lab-TAB manual (Goldsmith, 1999) | Cohen's κ = .47 - .99; ICC .71 - .95; Cronbach α = .77 |  | Evaluation objectivity through blinded coders | (Blandon et al., 2010; Bozicevic et al., 2021; Bozicevic et al., 2016; Buss & Goldsmith, 1998; Calkins et al., 2002; Gratz et al., 2014; Habersaat et al., 2013; Halligan et al., 2013; Tang et al., 2024; Ursache et al., 2013; Wu et al., 2021) | 10 (3.1%) |
|  |  |  |  |  |  |  |  |  |  |
|  |  | Toy Removal (Lab-TAB) | 30 sec – 3.5 min |  | Cohen's κ = .58 - .95; ICC = .85 - .99; Cronbach α = .77; Pearson r = .63 - .98; |  | Evaluation objectivity through blinded coders | (Anzman-Frasca et al., 2018; Granat et al., 2017; Halligan et al., 2013; Kim et al., 2014; Stępień-Nycz et al., 2015; Wu & Gazelle, 2021) | 6 (1.8%) |
|  |  |  |  |  |  |  |  |  |  |
|  |  | Delay of Gratification (with a cookie, chocolate) | 1 min - 10 min |  | Cohen's κ = .64 - .90; Cronbach α = .98; Pearson r = .63 - .98 |  | Evaluation objectivity through blinded coders | (Bridges et al., 1997; Chang et al., 2012; Chavez Arana et al., 2020; Feng et al., 2011; Stępień-Nycz et al., 2015) | 5 (1.5%) |
|  |  |  |  |  |  |  |  |  |  |
|  |  | Toy Clean-Up | 2 - 4 min |  | Cohen's κ = .69 - 1.0 |  | Implementation objectivity through standardazied/blinded observations procedures | (Feldman, 2015; Stifter. et al., 1999; Zimmermann & Stansbury, 2004) | 3 (0.9%) |
|  |  |  |  |  |  |  |  |  |  |
|  |  | Disappointment Task | 1 min |  | Cohen's κ = .69 - 1.0 |  |  | (Cole et al., 1994; Lapierre, 2016) | 2 (0.6%) |
|  |  |  |  |  |  |  |  |  |  |
|  |  | Attractive Toy in a Transparent Box | 3 min |  | Cohen's κ = .78 - .90 | Predictive validity: higher IQ predicted more constructive ER strategies (β = .71, p < .001) and poorer language skills predicted more avoidance strategies (β = −0.41, p = .03) (Wu et al., 2022) |  | (Kuiper et al., 2023; Witt et al., 2014) | 2 (0.6%) |
|  |  |  |  |  |  |  |  |  |  |
|  |  |  |  |  |  |  |  |  |  |
|  |  | The Parent-Ignore-Toddler-Situation (PITS) | 4.5 min |  | Cohen’s k = .73 - .98 |  |  | (Ekas et al., 2013) | 1 (0.3%) |
|  |  |  |  |  |  |  |  |  |  |
|  |  | Toy hidden in different ways |  | Infant Regulatory Scoring System (Weinberg & Tronick, 1994) | Pearson r = .76 - .90 |  |  | (Evrard et al., 2011) | 1 (0.3%) |
|  |  | **Fear Eliciting Task** | | | | | | | |
|  |  | Masks (Lab-TAB) | 10 sec - 3 min | Strategies of Emotional Regulation Scale (Faure et al., 2013) | Cohen's κ = .47 - .99; ICC = .86 |  | Evaluation objectivity through blinded coders | (Abraham et al., 2018; Halligan et al., 2013; Ursache et al., 2013; Witt et al., 2014; Wu & Gazelle, 2021; Wu et al., 2021) | 6 (1.8%) |
|  |  |  |  |  |  |  |  |  |  |
|  |  | Jumping Spider (Lab-TAB) | 10 - 20 sec. | Lab-TAB manual (Goldsmith, 1999) | Cohen's κ = .57 - .94; ICC = .81 - .99 |  | Evaluation objectivity through blinded coders | (Buss & Goldsmith, 1998; Gratz et al., 2014; Tsotsi et al., 2020) | 3 (0.9%) |
|  |  |  |  |  |  |  |  |  |  |
|  |  | Unpredictable Mechanical Toy (Lab-TAB) | 30 sec |  | Cohen's κ = .57 - .94 |  | Evaluation objectivity through blinded coders | (Buss & Goldsmith, 1998) | 1 (0.3%) |
|  |  |  |  |  |  |  |  |  |  |
|  |  | Observations During Vaccination Appointments | 3 min | LAB-TAB (Goldsmith & Rothbart, 1996) | Cohen's κ = .63 - .96 |  | Evaluation objectivity through blinded coders | (Gennis et al., 2023) | 1 (0.3%) |
|  |  | **Empathy Eliciting Task** | | | | | | | |
|  |  | The Emotional Resonance Task | 6 min |  |  |  |  | (Geangu et al., 2011) | 1 (0.3%) |
|  |  | **Joy Eliciting Task** | | | | | | | |
|  |  | Puppets Task (Lab-TAB) |  | Strategies of Emotional Regulation Scale (Faure et al., 2013) | Cohen's κ = .47 - .83 |  | Evaluation objectivity through blinded coders | (Habersaat et al., 2013; Hart & Behrens, 2013) | 2 (0.6%) |
|  |  |  |  |  |  |  |  |  |  |
|  |  | Umbrella Task (Lab-TAB) |  | Strategies of Emotional Regulation Scale (Faure et al., 2013) | Cohen's κ = .47 - .83 |  |  | (Habersaat et al., 2013) | 1 (0.3%) |
|  |  |  |  |  |  |  |  |  |  |
|  |  | Popping Bubbles (Lab-TAB) | 2 min |  | ICC = .89 |  | Evaluation objectivity through blinded coders | (Abraham et al., 2018) | 1 (0.3%) |
|  |  |  |  |  |  |  |  |  |  |
|  |  | Peek-a-boo (LAB-TAB) |  |  | Cohen's κ = .82 - .95 |  |  | (Granat et al., 2017) | 1 (0.3%) |
|  |  |  |  |  |  |  |  |  |  |
|  |  | Big Simon (Lab-TAB) |  |  | Cohen's κ = .81 |  | Evaluation objectivity through blinded coders | (Hart & Behrens, 2013) | 1 (0.3%) |
|  |  | **Non Specific Emotion Eliciting Tasks** | | | | | | | |
|  |  | Parent-Child Interaction; Nursing Child Assessment Teaching Scale (NCATS) | 3 - 90 min | Dysregulation Coding System (Lin & Crnic, 2012); Infant Caregiver and Engagement Phases (ICEP; (Weinberg & Tronick, 1999); Monadic Phase Manual (Tronick et al., 1980) | Cohen's κ =.60 - .89; ICC = .79 - .87; Cronbach α = .59 - .85;  82,9 % agreement between coders in 15% of the videos |  | Implementation objectivity through standardized observations procedures | (Black et al., 1996; Feldman, 2015; Feldman & Greenbaum, 1997; Lin et al., 2021; Miller et al., 2000; Neault, 2015; Riva Crugnola et al., 2019; Zimmermann & Stansbury, 2004) | 8 (2.5%) |
|  |  |  |  |  |  |  |  |  |  |
|  |  | Strange Situation Procedure; stranger-infant interaction episode | 50 sec - 8 min | Infant Regulatory Scoring System (IRSS; (Weinberg & Tronick, 1996) Infant and Caregiver Engagement Phases (ICEP; (Weinberg & Tronick, 1999) | Cohen’s k = 56 - 1.0; Pearson r = .71 - .89 |  | Evaluation objectivity through blinded coders | (Diener et al., 2002; Mangelsdorf et al., 1995; Riva Crugnola et al., 2011; Soussignan et al., 2009; Speranza, 2002) | 5 (1.5%) |
|  |  |  |  |  |  |  |  |  |  |
|  |  | Shape Sorter Task | 10 min |  | Cohen's κ = .77 - .82 |  | Evaluation objectivity through blinded coders | (Martins et al., 2016; Martins et al., 2012) | 2 (0.6%) |
|  |  |  |  |  |  |  |  |  |  |
|  |  | Behavior Response Paradigm (BRP) | 2 - 3 min |  | Cohen's κ = .70 - .94 |  |  | (Feldman, 2015) | 1 (0.3%) |
|  |  |  |  |  |  |  |  |  |  |
|  |  | Mobile task | 6 min | Self-Regulatory Behavior Scoring System (Gianino & Tronick, 2013) | Cohen's κ = .87 |  | Evaluation objectivity through blinded coders | (Shapiro et al., 1998) | 1 (0.3%) |
|  |  |  |  |  |  |  |  |  |  |
|  |  | Mother Separation–Reunion Episode |  |  | Cohen's κ = .70 - .94 |  |  | (Feldman, 2015) | 1 (0.3%) |
|  |  |  |  |  |  |  |  |  |  |
|  |  | Developmental Testing | 2.5 - 4 h | Bayley Scales of Infant Development (BSID-II, (Bayley, 1969) | Cronbach α = .82 |  | Evaluation objectivity through blinded coders | (Jones et al., 2017) | 1 (0.3%) |
|  |  |  |  |  |  |  |  |  |  |
|  |  | Daily Routines: separation from parents, diaper changes, conflicts between children |  |  | Cohen's κ = 0.79 - 1.0 |  |  | (Konishi et al., 2018) | 1 (0.3%) |
|  |  |  |  |  |  |  |  |  |  |
|  |  |  |  |  |  |  |  |  |  |
|  |  | Emotion Challenge Condition, Experimenter-Soothe Condition and Mother-Reunion Condition | 9 min |  |  |  |  | (Little & Carter, 2005) | 1 (0.3%) |
|  |  |  |  |  |  |  |  |  |  |
|  |  | Structured Exploratory and Problem-Solving Tasks | 10 - 20 min |  | Cohen's κ = .72 - .78; ICC .82 - .99 |  |  | (Carter et al., 1999) | 1 (0.3%) |

**Table 1b**

*Behavioral observation methods of emotion regulation for toddlers (13 to 36 months old)*

| **Age Range (months)** | **Task** | **Duration (sec/min/h)** | **Coding Manual** | **Reliability** | **Validity** | **Objectivity** | **Studies using this task** | **Number/ Percentage of studies using task** |
| --- | --- | --- | --- | --- | --- | --- | --- | --- |
| **Toddler** | | | | | | | | |
| **13 to 24 months** | **Frustration/Anger/Disappointment Eliciting Tasks** | | | | | | | |
|  | Delay of Gratification (cookie, snack, crayon or gift); Preschool Self-Regulation Assessment; PSRA, Party paradigm | 1 - 9 min |  | Cohen's κ = .58 - .98; ICC=.76–.96;  Pearson r = .63 - .98 | Support for construct and concurrent validity; Lab-TAB Tasks: Moderate to strong convergent validity of the negative emotion elicitation compared to other child observer ratings (*r* = .25 – .76; *r* = .27 – .41) | Evaluation objectivity through blinded coders | (Bendezú et al., 2018; Benga et al., 2018; Bridges et al., 1997; Calkins & Johnson, 1998; Calkins et al., 1998; Chang et al., 2012; Cole et al., 2013; Feldman, 2015; Feng et al., 2011; Gilliom et al., 2002; Green et al., 2023; Grolnick et al., 1996; Housman et al., 2023; Morales et al., 2005; Nuske et al., 2018; Ratcliff et al., 2021; Roben et al., 2013; Schoppmann et al., 2019, 2022; Stępień-Nycz et al., 2015; Supplee et al., 2011; Susa et al., 2014) | 22 (6.9%) |
|  |  |  |  |  |  |  |  |  |
|  | Attractive Toy in a Transparent Box | 30 sec - 10 min | Leiter International Performance Scale, Revised (Roid & Miller, 1997); Children’s Emotion Regulation Scale (Boyd-Soisson, 2002); Observational Coding System (OCS) | Cohen's κ = .40 - 1.0; ICC = .69 - .93; Cronbach α = .64 - .88;  Pearson r = .84 - .96 | Lab-TAB Tasks: Moderate to strong convergent validity of the negative emotion elicitation compared to other child observer ratings (r = .25 – .76; r = .27 – .41); Convergent: high correlation of ER and emotional reactivity (r = .91) | Evaluation objectivity through blinded coders | (Aviles et al., 2022; Calkins & Johnson, 1998; Calkins et al., 1998; Cibralic et al., 2024; Day. et al., 2022; Gallegos et al., 2017; Graziano et al., 2011; Graziano & Hart, 2016; Graziano, Keane & Calkins, 2010; Graziano et al., 2013; Hazen et al., 2010; Heeman et al., 2024; Hill et al., 2006; Khoury et al., 2016; Malin et al., 2014; Nuske et al., 2018; Qu et al., 2016; Villani et al., 2018; Williford et al., 2007; Witt et al., 2014) | 20 (6.2%) |
|  |  |  |  |  |  |  |  |  |
|  | High Chair (Lab-TAB) | 2 - 5 min | Observational Coding System (OCS) | Cohen's κ = .75 - 1.0; Cronbach α = .88; Pearson r = .70 - .96 |  |  | (Calkins et al., 1999; Calkins & Johnson, 1998; Calkins et al., 1998; Graziano, Calkins & Keane, 2010; Graziano et al., 2011; Graziano, Keane & Calkins, 2010; Graziano et al., 2013; Hill et al., 2006; Perry, Calkins et al., 2018; Smith. et al., 2006; Williford et al., 2007) | 11 (3.4%) |
|  |  |  |  |  |  |  |  |  |
|  | Attractive Toy behind Barrier | 30 sec – 4.5 min | Lab-TAB Manual (Goldsmith, 1999) | Cohen's κ = .75 - .98; ICC .71 - .95;  Pearson r = .70 |  | Evaluation objectivity through blinded coders | (Bocknek et al., 2020; Bocknek et al., 2018; Bozicevic et al., 2021; Bozicevic et al., 2016; Buss & Goldsmith, 1998; Calkins & Johnson, 1998; Frick et al., 2018; Gratz et al., 2014; Smith. et al., 2006; Wu & Gazelle, 2021; Wu et al., 2021) | 10 (3.1%) |
|  |  |  |  |  |  |  |  |  |
|  | Toy Removal Task | 2 – 3.5 min |  | Cohen's κ = .78 - .99; ICC = .85 - .99; Pearson r = .63 - .98 |  | Evaluation objectivity through blinded coders | (Feldman et al., 2011; Kerr et al., 2021; Kim et al., 2014; Roque et al., 2013; Stępień-Nycz et al., 2015; Ursache et al., 2013; Wu et al., 2021; Yan et al., 2021) | 8 (2.5%) |
|  |  |  |  |  |  |  |  |  |
|  | Still-Face-Paradigm | 1.5 - 6 min | Infant Self- Regulation Scheme (ISRS; (Millman, 2007); Child and Caregiver Mutual Regulation (CCMR) scoring system (Weinberg et al., 2008); Child and Caregiver Mutual Regulation (CCMR scoring system, (Weinberg et al., 2003) | Cohen's κ = .71 - .98; ICC > .80 |  | Evaluation objectivity through blinded coders | (Atkinson et al., 2021; Ding et al., 2024; Ekas et al., 2013; Feldman et al., 2011; Gago Galvagno, Elgier & Azzollini, 2022; Gago Galvagno et al., 2019; Gago Galvagno, Grandis et al., 2022; Gago-Galvagno, Miller et al., 2022; Gilliom et al., 2002) | 9 (2.8%) |
|  |  |  |  |  |  |  |  |  |
|  | Arm Restraint Task (Lab-TAB) | 30 sec - 2 min | Computerized coding software (Observational Coding System); Lab-TAB Manual (Goldsmith, 1999) | Cohen's κ = .40 - .94 |  | Evaluation objectivity through blinded coders | (Buss & Goldsmith, 1998; Day. et al., 2022; Gratz et al., 2014; Mirabile et al., 2009; Wu et al., 2021) | 5 (1.5%) |
|  |  |  |  |  |  |  |  |  |
|  | Disappointment Paradigm; Box Empty task (Lab Tab) | 1 - 8 min |  | Cohen's κ = .73 – .91; ICC = .79 - .87 | Lab-TAB Tasks: Moderate to strong convergent validity of the negative emotion elicitation compared to other child observer ratings (r = .25 – .76, r = .27 – .41) | Evaluation objectivity through blinded coders | (Nuske et al., 2018; Penela et al., 2015; Tan et al., 2013) | 3 (0.9%) |
|  |  |  |  |  |  |  |  |  |
|  | Clean-up Task | 3 min |  | Cohen's κ = .64 - 1.0 |  |  | (Gilliom et al., 2002; Kiel et al., 2020; Volling et al., 2002) | 3 (0.9%) |
|  |  |  |  |  |  |  |  |  |
|  | No Toy Task | 3 - 5 min |  | ICC=.76 – .96; Pearson r = .74 – .92 |  | Evaluation objectivity through blinded coders | (Supplee et al., 2009; Supplee et al., 2011) | 2 (0.6%) |
|  |  |  |  |  |  |  |  |  |
|  | Don’t Touch Task (Toys, Cookies) | 2 - 8 min | The Parent Child Interaction Coding Scheme (PARCHISY; (Deater-Deckard, 1997) | Cohen's κ = .83 - .99; ICC = .82 |  |  | (Hughes et al., 2015; Scherhag & Burgard, 2023) | 2 (0.6%) |
|  |  |  |  |  |  |  |  |  |
|  | Not Sharing Task (Lab-TAB) | 90 min |  | ICC = .82 - .96 |  |  | (Hassan et al., 2023) | 1 (0.3%) |
|  |  |  |  |  |  |  |  |  |
|  | End of the Line Tsk (Lab-TAB) | 30 sec - 2 min | Lab-TAB (Goldsmith, 1999) | Cohen's κ = .40 – .67 |  | Evaluation objectivity through blinded coders | (Day. et al., 2022) | 1 (0.3%) |
|  |  |  |  |  |  |  |  |  |
|  | Inter Adult Anger | 15 min |  | Cohen's κ = .74 - .88, ICC = .75 - 1.0 |  |  | (Maughan & Cicchetti, 2002) | 1 (0.3%) |
|  |  |  |  |  |  |  |  |  |
|  | Non-Opening Bottle | 30 - 60 sec. |  |  |  |  | (Wiefferink et al., 2012) | 1 (0.3%) |
|  |  |  |  |  |  |  |  |  |
|  | Toy Wrap (Preschool Self-Regulation Assessment; PSRA) |  |  |  | Support for construct and concurrent validity |  | (Housman et al., 2023) | 1 (0.3%) |
|  |  |  |  |  |  |  |  |  |
|  | Parent-Ignore-Toddler-Situation (PITS) | 4.5 min |  | Cohen's κ = .73 - .98 |  |  | (Ekas et al., 2013) | 1 (0.3%) |
|  |  |  |  |  |  |  |  |  |
|  | Toy hidden in different ways |  | Infant Regulatory Scoring System (Weinberg & Tronick, 1994) | Pearson r = .76 - .90 |  |  | (Evrard et al., 2011) | 1 (0.3%) |
|  | **Fear Eliciting Tasks** | | | | | | | |
|  | Spider (Lab-TAB) | 10 sec - 2 min | Lab-TAB Manual (Goldsmith & Rothbart, 1996) | Cohen's κ = .40 – 1.0; ICC = .70 - .95; Pearson r = .70 - .97 | Moderate to strong convergent validity of the negative emotion elicitation compared to other child observer ratings (r = .25 – .76; r = .27 – .41) | Evaluation objectivity through blinded coders | (Buss & Goldsmith, 1998; Day. et al., 2022; Gratz et al., 2014; Kiel et al., 2020; Macari et al., 2021; Nuske et al., 2018; Premo & Kiel, 2014, 2016; Price & Kiel, 2022; Smith. et al., 2006) | 10 (3.1%) |
|  |  |  |  |  |  |  |  |  |
|  | Masks (Lab-TAB) | 10 - 15 sec | Lab-TAB (Goldsmith, 1999) | Cohen's κ = .40 – .99; ICC = .82 - .95; Pearson r = .89 - .97 |  | Evaluation objectivity through blinded coders | (Abraham et al., 2018; Day. et al., 2022; Macari et al., 2021; Ursache et al., 2013; Witt et al., 2014; Wu et al., 2020; Wu et al., 2022; Wu & Gazelle, 2021; Wu et al., 2021) | 8 (2.5%) |
|  |  |  |  |  |  |  |  |  |
|  | Clown Episode (Lab-TAB) | 1 min |  | Cohen's κ = .91 - 1.0; ICC = .77 – .98 |  |  | (Kiel et al., 2020; Premo & Kiel, 2014, 2016; Price & Kiel, 2022) | 4 (1.2%) |
|  |  |  |  |  |  |  |  |  |
|  | Unpredictable Mechanical Toy (Lab-TAB) | 10 sec | Lab-TAB Manual (Goldsmith & Rothbart, 1996) | Cohen's κ = .57 - .94; ICC = .70 - .89 | Moderate to strong convergent validity of the negative emotion elicitation compared to other child observer ratings (r = .25 – .76; r = .27 – .41) | Evaluation objectivity through blinded coders | (Buss & Goldsmith, 1998; Nuske et al., 2018; Roque et al., 2013) | 3 (0.9%) |
|  |  |  |  |  |  |  |  |  |
|  | Observations During Vaccination Appointments | 3 min | LAB-TAB (Goldsmith & Rothbart, 1996) | Cohen's κ = .63 - .96 |  | Evaluation objectivity through blinded coders | (Gennis et al., 2023) | 1 (0.3%) |
|  | **Jealousy Eliciting Tasks** | | | | | | | |
|  | Triadic Interaction Paradigm | 9 min |  | Cohen's κ = .60 - .87 |  |  | (Kolak & Volling, 2011; Miller et al., 2000; Volling et al., 2002) | 3 (0.9%) |
|  | **Empathy Eliciting Tasks** | | | | | | | |
|  | Interaction Situation | 2 min |  | Cohen's κ = .66 - .67 |  |  | (Friedlmeier & Trommsdorff, 1999, 2001) | 2 (0.6%) |
|  | **Joy Eliciting Tasks** | | | | | | | |
|  | Puppet Show (Lab-TAB) | 1 - 2 min |  | Cohen's κ = .91-1; Pearson r = .70 |  |  | (Kiel et al., 2020; Premo & Kiel, 2014, 2016; Smith. et al., 2006) | 4 (1.2%) |
|  |  |  |  |  |  |  |  |  |
|  | Popping Bubbles (Lab-TAB) | 30 sec - 2 min | Lab-TAB Manual (Goldsmith & Rothbart, 1996) | Cohen's κ = .40 – .67; ICC = .89 |  | Evaluation objectivity through blinded coders | (Abraham et al., 2018; Day. et al., 2022) | 2 (0.6%) |
|  |  |  |  |  |  |  |  |  |
|  | Musical Toy |  |  |  |  | Evaluation objectivity through blinded coders, | (Roque et al., 2013) | 1 (0.3%) |
|  |  |  |  |  |  |  |  |  |
|  | Make that Car Go (Lab-TAB) | 30 sec - 2 min | Lab-TAB Manual (Goldsmith, 1999) | Cohen's κ = .40 – .67 |  | Evaluation objectivity through blinded coders | (Day. et al., 2022) | 1 (0.3%) |
|  |  |  |  |  |  |  |  |  |
|  | Cognitive Assimilation Game Task | 2.4 min |  | ICC = .84 - .93 |  | Evaluation objectivity through blinded coders | (Bocknek et al., 2020) | 1 (0.3%) |
|  |  |  |  |  |  |  |  |  |
|  | Peek a Boo (Lab-TAB) | 30 sec - 2 min | Lab-TAB Manual (Goldsmith & Rothbart, 1996) | Cohen's κ = .40 – .67 |  | Evaluation objectivity through blinded coders | (Day. et al., 2022) | 1 (0.3%) |
|  | **Non Specific Emotion Eliciting Task or Mixed Assessments** | | | | | | | |
|  | Strange Situation; Stranger Approach; Lab-TAB;Stranger-Infant-Episode | 30 sec - 3 min | Lab-TAB Manual (Goldsmith, 1999); Infant Regulatory Scoring System (IRSS; (Weinberg & Tronick, 1996); Infant and Caregiver Engagement Phases (ICEP (Weinberg & Tronick, 1999) | Cohen's κ = .40 - 1.0; ICC = .82 - .95; Pearson r = .89 - .97 | Moderate to strong convergent validity of the negative emotion elicitation compared to other child observer ratings (r = .25 – .76; r = .27 – .41) | Evaluation objectivity through blinded coders | (Day. et al., 2022; Diener et al., 2002; Gilliom et al., 2002; Macari et al., 2021; Mangelsdorf et al., 1995; Nuske et al., 2018; Riva Crugnola et al., 2011) | 7 (2.2%) |
|  |  |  |  |  |  |  |  |  |
|  | Problem-Solving Task | 5 - 15 min | Coping Scale (Matas et al., 1978); Children’s Emotion Regulation Scale (Boyd-Soisson, 2002; Hazen et al., 2010) | Cohen's κ = .96; ICC = .82 - .99 |  | Evaluation objectivity through blinded coders | (Aviles et al., 2022; Carter et al., 1999; Davies et al., 2013; Gallegos et al., 2017; Hazen et al., 2010; Woodward et al., 2017) | 6 (1.8%) |
|  |  |  |  |  |  |  |  |  |
|  | Parent-Child Interaction | 8 - 15 min | Leiter International Performance Scale, Revised (Roid & Miller, 1997); Dysregulation Coding System (Lin & Crnic, 2012) | ICC = .79 - .90; Pearson r = .68 - .90 |  | Evaluation objectivity through blinded coders | (Cimino & Cerniglia, 2018; Lin et al., 2021; Malin et al., 2014; Woodward et al., 2017) | 4 (1.2%) |
|  |  |  |  |  |  |  |  |  |
|  | Semi-structured Play Tasks | 5 - 10 min | Emotion regulation subscale (BBRS); Bayley Scales of Infant Development–II (Bayley, 1993) | Cohen's κ = .82 - .87; Cronbach α > .90 | Construct validity across different populations | Implementation objectivity through blinded assessors | (Mortensen & Barnett, 2018, 2019; Nowak, 2020; Volling et al., 2002) | 4 (1.2%) |
|  |  |  |  |  |  |  |  |  |
|  | Free Play | 10 min | C-CARES coding system (Tamis-LeMonda et al., 2001) | Cohen's κ = .78 - .85 |  | Evaluation objectivity through blinded coders | (Cabrera et al., 2017; Erickson et al., 2013; Feldman et al., 2011) | 3 (0.9%) |
|  |  |  |  |  |  |  |  |  |
|  | Separation Episode; Separation from mother (LAB-TAB) | 30 sec - 15 min |  | Cohen's κ = .79 - 1.00;  ICC = .74–.94 |  | Evaluation objectivity through blinded coders | (Lozano, 2005; Molitor et al., 2003; Volling et al., 2002) | 3 (0.9%) |
|  |  |  |  |  |  |  |  |  |
|  | Puppet Interview | 1.5 min |  | Cohen's κ = .82 - .87 |  |  | (Volling et al., 2002) | 1 (0.3%) |
|  |  |  |  |  |  |  |  |  |
|  | Toddler-Sibling Interaction; Toddler-Sibling-Stranger Interaction | 8 min |  | Cohen's κ = .75 |  |  | (Garner, 1995) | 1 (0.3%) |
|  |  |  |  |  |  |  |  |  |
|  | Teaching Task | 3 min |  | Cohen's κ = .64 - .79 |  |  | (Gilliom et al., 2002) | 1 (0.3%) |
|  |  |  |  |  |  |  |  |  |
|  | Puzzle Task | 5 min | Coping Scale (Matas et al., 1978) | ICC = .55 - .96 |  |  | (Davies et al., 2013) | 1 (0.3%) |
|  |  |  |  |  |  |  |  |  |
|  | Shape Sorter Task | 5 min | Coping Scale (Matas et al., 1978) | ICC = .55 - .92 |  |  | (Davies et al., 2013) | 1 (0.3%) |
|  |  |  |  |  |  |  |  |  |
|  | Preschool Self-Regulation Assessment (PSRA); Balance Beam, Pencil Tap |  |  |  | Support for the construct and concurrent validity |  | (Housman et al., 2023) | 1 (0.3%) |
|  |  |  |  |  |  |  |  |  |
|  | Day-Care Activities |  |  | Cohen's κ = .70 - 0.83 |  |  | (Kurki et al., 2017) | 1 (0.3%) |
| **25 to 36 months** | **Frustration/Anger/Disappointment Eliciting Tasks** | | | | | | | |
|  | Delay of Gratification | 2 - 8 min | Dysregulation Coding System (Hoffman et al., 2006) | Cohen's κ = .68 - .92; ICC = .54 - .98; Cronbach α = .64 – 1.0;  Pearson r = .69 - .99 | Moderate to strong convergent validity of the negative emotion elicitation compared to other child observer ratings (r = .25 – .76; r = .27 – .41) | Evaluation objectivity through blinded coders | (Bendezú et al., 2018; Benga et al., 2018; Boldt et al., 2020; Byrd et al., 2021; Cole et al., 2013; Dennis, Cole et al., 2009; Gilliom et al., 2002; Harrington, 2021; Hoffman et al., 2006; Housman et al., 2023; Kochanska et al., 2009; Nuske et al., 2018; Roben et al., 2013; Schoppmann et al., 2023; Senehi & Brophy-Herb, 2020; Supplee et al., 2011; Zimmermann & Stansbury, 2003) | 17 (5.3%) |
|  |  |  |  |  |  |  |  |  |
|  | Attractive Toy in a Transparent Box | 2 - 8 min |  | Cohen's κ = .68 - 1.0; ICC = .78;  Pearson r = .95 - .71 | Moderate to strong convergent validity of the negative emotion elicitation compared to other child observer ratings (r = .25 – .76, Gagne et al. 2011; r = .27 – .41); Concurrent validity: Correlation of ER with established temperament measures (r = .45, p < .01); (Yang et al., 2021); Predictive validity: higher IQ predicted more constructive ER strategies (β = .71, p < .001) and poorer language skills predicted more avoidance strategies (β = −0.41, p = .03) | Evaluation objectivity through blinded coders | (Binion & Zalewski, 2018; Blandon et al., 2010; Blankson et al., 2017; Byrd et al., 2021; Cole et al., 2009; Dennis, Cole et al., 2009; Graziano et al., 2011; Jahromi et al., 2012; Lee et al., 2023; Ntourou et al., 2013; Nuske et al., 2018; Wu et al., 2020; Wu et al., 2022; Yan et al., 2021) | 14 (4.4%) |
|  |  |  |  |  |  |  |  |  |
|  | Disappointment Task | 1 - 5 min |  | Cohen's κ = .60 - 1.0 | Convergent Validity: Emotion regulation strategies in Disappointment Task were positively correlated with mother-reported emotion regulation strategies (adaptive: r = .30, p = .046; maladaptive: r = .32, p = .035) | Implementation and evaluation objectivity through blinded assessors and coders | (Dennis et al., 2010; Forbes, Shaw et al., 2006; Nuske et al., 2018; Ren et al., 2016; Wu et al., 2020; Wu et al., 2017; Wu et al., 2022; Xie et al., 2021) | 8 (2.5%) |
|  |  |  |  |  |  |  |  |  |
|  | Clean-up Task | 2 - 5 min | Dysregulation Coding System (Hoffman et al., 2006) | Cohen's κ =.64 - 1.0; Pearson r = .90 |  | Evaluation objectivity through blinded coders | (Gilliom et al., 2002; Hoffman et al., 2006; Stansbury & Sigman, 2000; Wu et al., 2017) | 4 (1.2%) |
|  |  |  |  |  |  |  |  |  |
|  | Toy Removal Task | 3 min |  | Cohen's κ = .78 - .85 |  | Evaluation objectivity through blinded coders | (Feldman et al., 2011; Kerr et al., 2021; Roque et al., 2013) | 3 (0.9%) |
|  |  |  |  |  |  |  |  |  |
|  | Attractive Toy behind Barrier (Lab-TAB) | 4.5 min |  | Cohen's κ = .83;  ICC = .84 - .93 |  |  | (Bocknek et al., 2018; Costa et al., 2019) | 2 (0.6%) |
|  |  |  |  |  |  |  |  |  |
|  | Preschool Self-Regulation Assessment (PSRA): Toy Wrap |  | PSRA-AR (Smith-Donald et al., 2007) | ICC = .72 |  |  | (Bailey et al., 2016; Bailey et al., 2022) | 2 (0.6%) |
|  |  |  |  |  |  |  |  |  |
|  | No Toy Task | 5 min |  | ICC=.76 – .96 |  | Evaluation objectivity through blinded coders | (Supplee et al., 2009; Supplee et al., 2011) | 2 (0.6%) |
|  |  |  |  |  |  |  |  |  |
|  | Impossibly Perfect Circles task (IPC; LAB-TAB) | 2 min |  | Cohen's κ = .60 – 1.0 | The validity of the task and the coding is demonstrated in previous studies (Calkins & Keane, 2004; Graziano et al., 2011; Mazzer, 2023; Rodríguez et al., 2014; Zimmermann & Stansbury, 2003) |  | (Dennis et al., 2010; Mazzer, 2023) | 2 (0.6%) |
|  |  |  |  |  |  |  |  |  |
|  | Still-Face Task | 1.5 min | Child and Caregiver Mutual Regulation (CCMR) scoring system (Weinberg et al., 2008) | Cohen's κ = .78 - .85 |  | Implementation objectivity through blinded assessors | (Ding et al., 2024; Feldman et al., 2011; Gago Galvagno, Grandis et al., 2022) | 3 (0.9%) |
|  |  |  |  |  |  |  |  |  |
|  | High Chair (Lab-TAB) | 2 - 5 min |  | Cohen's κ = .72 - 1.0 |  |  | (Graziano et al., 2011) | 1 (0.3%) |
|  |  |  |  |  |  |  |  |  |
|  | Arm Restraint Task (Lab-TAB) | 30 sec - 2 min | Lab TAB-Manual (Goldsmith, 1999) | Cohen's κ = .40 - .94 |  | Evaluation objectivity through blinded coders | (Day. et al., 2022) | 1 (0.3%) |
|  |  |  |  |  |  |  |  |  |
|  | No Stickers Left Task (Lab-TAB) | 2 min |  | Cohen's κ = .76 - 1.0 |  | Implementation objectivity through blinded assessors | (Wu & Feng, 2020) | 1 (0.3%) |
|  |  |  |  |  |  |  |  |  |
|  | End of the Line Tsk (Lab-TAB) | 30 sec - 2 min | Lab TAB-Manual (Goldsmith, 1999) | Cohen's κ = .40 – .67 |  | Evaluation objectivity through blinded coders | (Day. et al., 2022) | 1 (0.3%) |
|  |  |  |  |  |  |  |  |  |
|  | Not Sharing Task (Lab-TAB) | 90 min |  | ICC = .82 - .96 |  |  | (Hassan et al., 2023) | 1 (0.3%) |
|  |  |  |  |  |  |  |  |  |
|  | Knotted Sack |  |  | ICC = .78 |  | Evaluation objectivity through blinded coders | (Byrd et al., 2021) | 1 (0.3%) |
|  |  |  |  |  |  |  |  |  |
|  | Busy Caregiver Paradigm | 2 min |  | Cohen's κ = .85 - 1.0 |  | Evaluation objectivity through blinded coders | (Zimmermann & Stansbury, 2003) | 1 (0.3%) |
|  |  |  |  |  |  |  |  |  |
|  | Child waiting quietly | 3 min |  |  |  | Evaluation objectivity through blinded coders | (Arnold et al., 2011) | 1 (0.3%) |
|  |  |  |  |  |  |  |  |  |
|  | Inter Adult Anger | 15 min |  | Cohen's κ = .74 - .88; ICC = .75 - 1.0 |  |  | (Maughan et al., 2007) | 1 (0.3%) |
|  |  |  |  |  |  |  |  |  |
|  | Non-Opening Bottle | 30 - 60 sec. |  |  |  |  | (Wiefferink et al., 2012) | 1 (0.3%) |
|  |  |  |  |  |  |  |  |  |
|  | Candy given, then denied | 2 min |  | Cohen's κ =.68 - 1.0 |  |  | (Stansbury & Sigman, 2000) | 1 (0.3%) |
|  | **Fear Eliciting Tasks** | | | | | | | |
|  | Masks (Lab-TAB) | 15 - 30 sec | Lab-TAB Manual (Goldsmith, 1999) | Cohen's κ = .40 – .94; ICC = .86;  Pearson r = .80 – .94 |  | Evaluation objectivity through blinded coders | (Abraham et al., 2018; Day. et al., 2022; Hirschler-Guttenberg, Feldman et al., 2015; Hirschler-Guttenberg, Golan et al., 2015) | 4 (1.2%) |
|  |  |  |  |  |  |  |  |  |
|  | Spider (Lab-TAB) | 2 - 4 min | Lab-TAB Manual (Goldsmith, 1999) | Cohen's κ = .40 – .67; Pearson r = 0.69 - .99 |  | Evaluation objectivity through blinded coders | (Calkins & Dedmon, 2000; Day. et al., 2022) | 2 (0.6%) |
|  |  |  |  |  |  |  |  |  |
|  | Unpredictable Mechanical Toy (Lab-TAB) |  |  | Agreement: 70.51 – 89.10 % | Moderate to strong convergent validity of the negative emotion elicitation compared to other child observer ratings (r = .25 – .76; r = .27 – .41) | Evaluation objectivity through blinded coders | (Nuske et al., 2018; Roque et al., 2013) | 2 (0.6%) |
|  |  |  |  |  |  |  |  |  |
|  | Wiggle Ball |  |  | Cohen's κ = .59 - .76 |  |  | (Forbes, Fox et al., 2006) | 1 (0.3%) |
|  | **Jealousy Eliciting Tasks** | | | | | | | |
|  | Triadic Interaction Paradigm | 9 min |  | Cohen's κ = .70 |  |  | (Miller et al., 2000) | 1 (0.3%) |
|  | **Empathy Eliciting Tasks** | | | | | | | |
|  | Audiotape of Crying Toddler | 2 min |  | Pearson r = 0.69 - .99 |  | Implementation and evaluation objectivity through blinded assessors/coders | (Calkins & Dedmon, 2000) | 1 (0.3%) |
|  |  |  |  |  |  |  |  |  |
|  | Interaction Situation | 2 min |  | Cohen's κ = .66 - .67 |  |  | (Friedlmeier & Trommsdorff, 2001) | 1 (0.3%) |
|  | **Joy Eliciting Tasks** | | | | | | | |
|  | Peek a Boo (Lab-TAB) | 30 sec - 4 min | Lab-TAB Manual (Goldsmith & Rothbart, 1996) | Cohen's κ = .40 – .67; Pearson r = 0.69 - .99 |  | Implementation objectivity through blinded assessors | (Calkins & Dedmon, 2000; Day. et al., 2022) | 2 (0.6%) |
|  |  |  |  |  |  |  |  |  |
|  | Popping Bubbles (Lab-TAB) | 30 sec - 2 min | Lab-TAB Manual (Goldsmith, 1999) | Cohen's κ = .40 – .67; ICC = .89 |  | Evaluation objectivity through blinded coder | (Abraham et al., 2018; Day. et al., 2022) | 2 (0.6%) |
|  |  |  |  |  |  |  |  |  |
|  | Puppet Show (Lab-TAB) | 5 min |  | Cohen's κ = .75 – .94 |  | Evaluation objectivity through blinded coders | (Hirschler-Guttenberg, Golan et al., 2015) | 1 (0.3%) |
|  |  |  |  |  |  |  |  |  |
|  | Musical Toy |  |  |  |  | Evaluation objectivity through blinded coders | (Roque et al., 2013) | 1 (0.3%) |
|  |  |  |  |  |  |  |  |  |
|  | Make that Car Go (Lab-TAB) | 30 sec - 2 min | Lab-TAB Manual (Goldsmith, 1999) | Cohen's κ = .40 – .67 |  | Evaluation objectivity through blinded coders | (Day. et al., 2022) | 1 (0.3%) |
|  | Cognitive Assimilation Game Task | 2.4 min |  | ICC .84 - .93 |  |  | (Bocknek et al., 2020) | 1 (0.3%) |
|  |  |  |  |  |  |  |  |  |
|  | Tickle-Me-Elmo Toy | 5 min |  | Cohen's κ = .84 - .96 |  |  | (Wu et al., 2017) | 1 (0.3%) |
|  | **Non Specific Emotion Eliciting Task** | | | | | | | |
|  | Stranger Approach; Strange Situation | 30 sec - 3 min |  | Cohen's κ = .40 - 1.0 | Moderate to strong convergent validity of the negative emotion elicitation compared to other child observer ratings (r = .25 – .76, Gagne et al. 2011; r = .27 – .41) | Evaluation objectivity through blinded coders | (Day. et al., 2022; Gilliom et al., 2002; Nuske et al., 2018; Zimmermann & Stansbury, 2003, 2004) | 5 (1.5%) |
|  |  |  |  |  |  |  |  |  |
|  | Puzzle Task | 2 - 5 min |  | Cohen's κ = .59 - 1.0; ICC = .55 - .94; Pearson r = .69 - .99 |  | Implementation objectivity through blinded/ trained assessors | (Calkins & Dedmon, 2000; Davies et al., 2013; Forbes, Fox et al., 2006; Jahromi et al., 2012; Perry, Swingler et al., 2016) | 5 (1.5%) |
|  |  |  |  |  |  |  |  |  |
|  | Parent-Child Interaction: mothers; fathers | 3 - 10 min | Dysregulation Coding System (Hoffman et al., 2006); Emotion Dysregulation subscale (adapted from (Cole et al., 1994); Leiter International Performance Scale, Revised (Roid & Miller, 1997) | ICC = .61 - .90; Cronbach α = .63 - 1.0 |  | Evaluation objectivity through blinded coders | (Cabrera et al., 2017; Gulsrud et al., 2010; Malin et al., 2014; Norona & Baker, 2017; Schatz et al., 2008) | 4 (1.2%) |
|  |  |  |  |  |  |  |  |  |
|  | Problem-Solving Task | 2 - 5 min | Dysregulation Coding System (Hoffman et al., 2006); Emotion Dysregulation subscale (adapted from (Cole et al., 1994) | ICC = .79 - .90, Pearson r = .69 - .99 |  | Implementation and evaluation objectivity through blinded assessors/coders; | (Calkins & Dedmon, 2000; Hoffman et al., 2006; Norona & Baker, 2014; Noroña et al., 2018) | 4 (1.2%) |
|  |  |  |  |  |  |  |  |  |
|  | Free Play |  |  | Cohen's κ = .79 - .92; Cronbach α > .70 |  | Evaluation objectivity through blinded coders | (Black et al., 1996; Feldman et al., 2011; Garner, 2006) | 3 (0.9%) |
|  |  |  |  |  |  |  |  |  |
|  | Shape Sorter Task; Toy Sort Task | 10 min |  | Cohen's κ = .77 - .82; ICC = .55 - .92 |  | Evaluation objectivity through blinded coders | (Davies et al., 2013; Kalpidou et al., 2004; Martins et al., 2016) | 3 (0.9%) |
|  |  |  |  |  |  |  |  |  |
|  | Bayley Scales of Infant Development |  | Bayley Scales of Infant Development–II (Bayley, 1993) | ICC .81 - .94; Cronbach α > .90 | Construct validity across different populations | Evaluation objectivity through blinded coders | (Feldman & Klein, 2003; Mortensen & Barnett, 2018) | 2 (0.6%) |
|  |  |  |  |  |  |  |  |  |
|  | Preschool Self-Regulation Assessment (PSRA): Pencil Tap, Toy Sort, Balance Beam |  | PSRA-AR (Smith-Donald et al., 2007) | ICC = .72 | Support for the construct and concurrent validity | Evaluation objectivity through blinded coders | (Bailey et al., 2016; Housman et al., 2023) | 2 (0.6%) |
|  |  |  |  |  |  |  |  |  |
|  | Emotional Oddball Task |  |  | Cronbach α = .88; Spearman Brown split half = .76 |  |  | (Clark et al., 2016) | 1 (0.3%) |
|  |  |  |  |  |  |  |  |  |
|  | Motor Tasks |  |  | Cohen's κ = .59 - 1.00 |  |  | (Forbes, Fox et al., 2006) | 1 (0.3%) |
|  |  |  |  |  |  |  |  |  |
|  | Teaching Task | 3 min |  | Cohen's κ = .64 - .79 |  |  | (Gilliom et al., 2002) | 1 (0.3%) |
|  |  |  |  |  |  |  |  |  |
|  | Class Room Observations | 10 min |  |  |  |  | (Onchwari & Keengwe, 2011) | 1 (0.3%) |
|  |  |  |  |  |  |  |  |  |
|  | Day-Care Activities |  |  | Cohen's κ = .70 - .83 |  |  | (Kurki et al., 2017) | 1 (0.3%) |

**Table 1c**

*Behavioral observation methods of emotion regulation for preschoolers (37 to 62 months)*

| **Age Range (months)** | **Task** | **Duration (sec/min/h)** | **Coding Manual** | **Reliability** | **Validity** | **Objectivity** | **Studies using this task** | **Number/ Percentage of studies using task** |
| --- | --- | --- | --- | --- | --- | --- | --- | --- |
| **Preschool** | | | | | | | | |
| **37 to 49 months** | **Frustration/Anger/Disappointment Eliciting Tasks** | | | | | | | |
|  | Delay of Gratification (Forbidden Toy paradigm, Preschool Self-Regulation Assessment, PSRA) | 3 - 8 min | Dysregulation Coding System (Hoffman et al., 2006) | Cohen's κ = .64 - 1.0; ICC = .54 - .98; Cronbach α = .81 - 1.0;  Krippendorf’s α = .62- .99;  Pearson r = .90 | Support for construct and concurrent validity (Housman et al., 2022); Discriminant validity: sing. greater sadness during delay than during neutral task (F = 6.01, p < .05) | Evaluation objectivity through blinded coders | (Bailey et al., 2016; Bailey et al., 2022; Baker et al., 2007; Bendezú et al., 2018; Byrd et al., 2021; Cole et al., 2009; Cole et al., 2013; Feng et al., 2008; Green et al., 2023; Harrington, 2021; Hoffman et al., 2006; Housman et al., 2023; Kochanska et al., 2009; Morales & Bridges, 1996; Nuske et al., 2018; Roben et al., 2013; Santucci et al., 2008; Schoppmann et al., 2023; Silk, Shaw, Forbes et al., 2006; Silk, Shaw, Skuban et al., 2006; Snyder & Arnold, 2022) | 21 (6.6%) |
|  |  |  |  |  |  |  |  |  |
|  | Attractive Toy in a Transparent Box (Lab-TAB) | 3.5 - 8 min | Dysregulation Coding System (Hoffman et al., 2006) | Cohen's κ = .68 - 1.0; ICC = .70 - .98 | Moderate to strong convergent validity of the negative emotion elicitation compared to other child observer ratings (r = .25 – .76; r = .27 – .41); Concurrent validity: Correlation of ER with established temperament measures (r = .45, p < .01); Predictive validity: higher IQ predicted more constructive ER strategies (β = .71, p < .001) and poorer language skills predicted more avoidance strategies (β = −0.41, p = .03) | Evaluation objectivity through blinded coders | (Baker et al., 2019; Berry et al., 2019; Binion & Zalewski, 2018; Byrd et al., 2021; Cole et al., 2009; Dennis, Cole et al., 2009; Distefano et al., 2021; Fenning et al., 2018; Graziano et al., 2011; Harden et al., 2017; Hill et al., 2006; Jahromi et al., 2012; Lee et al., 2023; Ntourou et al., 2013; Nuske et al., 2018; Qu et al., 2016; Wu et al., 2020; Wu et al., 2022; Yan et al., 2021; Zantinge et al., 2017) | 20 (6.2%) |
|  |  |  |  |  |  |  |  |  |
|  | Disappointing Paradigm | 1 - 5 min |  | Cohen's κ = .61 - 1.0; ICC = .98;  Cronbach α = .79 - .94 | Disappointing Paradigm: Emotion regulation strategies in Disappointment Task correlate with mother reported emotion regulation strategies (adaptive: r = .30, p = .046; maladaptive: r = .32, p = .035; convergent validity) ; Lab-TAB Tasks: Moderate to strong convergent validity of the negative emotion elicitation compared to other child observer ratings (r = .25 – .76; r = .27 – .41) | Evaluation objectivity through blinded coders | (Dennis et al., 2010; Feng et al., 2008; Forbes, Fox et al., 2006; Forbes, Shaw et al., 2006; Hernandez et al., 2019; Morris et al., 2011; Nuske et al., 2018; Oeri & Roebers, 2020; Petersen & Holodynski, 2020; Power et al., 2016; Ren et al., 2016; Ren et al., 2018; Tan et al., 2013; Wu & Feng, 2020; Wu et al., 2022; Xie et al., 2021) | 16 (5%) |
|  |  |  |  |  |  |  |  |  |
|  | Clean-Up Task | 2 - 5 min |  | Cohen's κ = .68 - 1.00;  Pearson r = .90 |  | Evaluation objectivity through blinded coders | (Hoffman et al., 2006; Rodríguez et al., 2014; Stansbury & Sigman, 2000) | 3 (0.9%) |
|  |  |  |  |  |  |  |  |  |
|  | Toy Behind Barrier Task | 1,5 min |  | Cohen's κ = .83 - .87; Krippendorf’s α = .80 - 1.0 |  | Implementation objectivity through blinded/ trained assessors | (Costa et al., 2019; Murnan et al., 2021) | 2 (0.6%) |
|  |  |  |  |  |  |  |  |  |
|  | Impossibly Perfect Circles Task (IPC; LAB-TAB) | 3.5 min |  | Cohen's κ = .60 – 1.0 | The validity of the task and the coding is demonstrated in previous studies (Calkins & Keane, 2004; Graziano et al., 2011; Mazzer, 2023; Rodríguez et al., 2014; Zimmermann & Stansbury, 2003) |  | (Dennis et al., 2010; Mazzer, 2023) | 2 (0.6%) |
|  |  |  |  |  |  |  |  |  |
|  | Inter Adult Anger | 15 min |  | Cohen's κ = .74 - .88; ICC = .75 - 1.0 |  |  | (Maughan & Cicchetti, 2002; Maughan et al., 2007) | 2 (0.6%) |
|  |  |  |  |  |  |  |  |  |
|  | Not Sharing Task (LAB-TAB) | 5 min |  | Cohen's κ = .83 - .98 |  |  | (Kim et al., 2023) | 1 (0.3%) |
|  |  |  |  |  |  |  |  |  |
|  | No Stickers Left Task (Lab-TAB) | 2 min |  | Cohen's κ = .76 - 1.0 |  | Evaluation objectivity through blinded coders | (Wu et al., 2020) | 1 (0.3%) |
|  |  |  |  |  |  |  |  |  |
|  | Frustration Task according to Montessori button frame | 3 min |  | ICC > .70 |  |  | (Helmsen, 2011) | 1 (0.3%) |
|  | Still-Face Paradigm | 6 min | Infant Regulatory Scoring System (Tronick, 1990) | Cohen's κ = .96 - .98 |  |  | (Maclean et al., 2014) | 1 (0.3%) |
|  | Not Sharing Task (Lab-TAB) | 90 min |  | ICC= .82 - .96 |  |  | (Hassan et al., 2023) | 1 (0.3%) |
|  | Knotted Sack |  |  | ICC = .78 |  | Implementation objectivity through blinded/ trained assessors | (Byrd et al., 2021) | 1 (0.3%) |
|  | Non-Opening Bottle | 30 - 60 sec |  |  |  |  | (Wiefferink et al., 2012) | 1 (0.3%) |
|  |  |  |  |  |  |  |  |  |
|  | Child waiting quietly | 3 min |  |  |  | Evaluation objectivity through blinded coders | (Arnold et al., 2011) | 1 (0.3%) |
|  |  |  |  |  |  |  |  |  |
|  | Candy given, then denied | 2 min |  | Cohen's κ =.68 - 1.0 |  |  | (Stansbury & Sigman, 2000) | 1 (0.3%) |
|  | **Fear Eliciting Tasks** | | | | | | | |
|  | Masks (Lab-TAB) | 15 - 30 sec |  | Cohen's κ = .40 – .94; ICC = .86;  Pearson r = .80 – .94 |  | Evaluation objectivity through blinded coders | (Abraham et al., 2018; Hirschler-Guttenberg, Feldman et al., 2015; Hirschler-Guttenberg, Golan et al., 2015) | 3 (0.9%) |
|  |  |  |  |  |  |  |  |  |
|  | Spider (Lab-TAB); Lizard |  |  | ICC = .81 - .99 |  |  | (Tsotsi et al., 2023) | 1 (0.3%) |
|  |  |  |  |  |  |  |  |  |
|  | Unpredictable Mechanical Toy (Lab-TAB) |  |  | Agreement: 70.51 – 89.10 % | Moderate to strong convergent validity of the negative emotion elicitation compared to other child observer ratings (r = .25 – .76; r = .27 – .41) | Evaluation objectivity through blinded coders | (Nuske et al., 2018) | 1 (0.3%) |
|  |  |  |  |  |  |  |  |  |
|  | Clown Task | 10 sec |  | Cohen's κ = .31 - .88 |  |  | (Root et al., 2015) | 1 (0.3%) |
|  | **Jealousy Eliciting Tasks** | | | | | | | |
|  | Triadic Interaction Paradigm | 9 min |  | Cohen's κ = .70 |  |  | (Miller et al., 2000) | 1 (0.3%) |
|  | **Joy Eliciting Tasks** | | | | | | | |
|  | Popping Bubbles (Lab-TAB) | 2 min |  | ICC = .89 - .98 | Good construct and predictive validity across a range of studies | Evaluation objectivity through blinded coders | (Abraham et al., 2018; Harden et al., 2017) | 2 (0.6%) |
|  |  |  |  |  |  |  |  |  |
|  | Secret Keeping Task | 3,5 min |  | Cohen's κ = .61 - 1.0 |  |  | (Hernandez et al., 2019) | 1 (0.3%) |
|  | **Non Specific/ Mixed Emotion Eliciting Tasks** | | | | | | | |
|  | Toy Sort | 10 min |  | Cohen's κ = .69 - 1.0; ICC = .73 - .99; Cronbach α = .87 | Support for the construct and concurrent validity |  | (Bailey et al., 2016; Bailey et al., 2022; Housman et al., 2023; Kalpidou et al., 2004; McCoy & Raver, 2011) | 5 (1.5%) |
|  |  |  |  |  |  |  |  |  |
|  | Problem Solving Task | 10 - 15 min | Emotion Dysregulation subscale (Cole et al., 1994); Dysregulation coding system (Hoffman et al., 2006) | ICC = .79 - .90; Pearson r = .79 - .90 |  | Evaluation objectivity through blinded coders | (Hoffman et al., 2006; Norona & Baker, 2014; Noroña et al., 2018; Woodward et al., 2017) | 4 (1.2%) |
|  |  |  |  |  |  |  |  |  |
|  | Free Play | 10 min |  | Cohen's κ = .72 - .96 |  |  | (Garner, 2006; Garner & Spears, 2000; Rodríguez et al., 2014; Silkenbeumer et al., 2018) | 4 (1.2%) |
|  |  |  |  |  |  |  |  |  |
|  | Puzzle Task | 4 - 5 min | Dysregulation Coding System (Hoffman et al., 2006) | Cohen's κ = .81 - 1.0; ICC = .90 | Validity supported by prior studies of preschool- and school-aged children with and without neurodevelopmental disorders (Baker et al., 2007; Fenning et al., 2018; Hoffman et al., 2006; Norona & Baker, 2014) | Evaluation objectivity through blinded coders | (Baker et al., 2019; Blankson et al., 2017; Fenning et al., 2018; Jahromi et al., 2012) | 4 (1.2%) |
|  |  |  |  |  |  |  |  |  |
|  | Preschool Self-Regulation Assessment (PSRA): Balance Beam; Pencil Tap |  |  | ICC = .73 - .99; Cronbach α = .87 | Support for the construct and concurrent validity across different populations | Evaluation objectivity through blinded coders | (Bailey et al., 2016; Bailey et al., 2022; Housman et al., 2023; McCoy & Raver, 2011) | 4 (1.2%) |
|  |  |  |  |  |  |  |  |  |
|  | Stranger Approach | 2 min |  | Cohen's κ = .85 - 1.0; Agreement: 70.51 – 89.10 % | Moderate to strong convergent validity of the negative emotion elicitation compared to other child observer ratings (r = .25 – .76; r = .27 – .41) | Evaluation objectivity through blinded coders | (Nuske et al., 2018; Zimmermann & Stansbury, 2003, 2004) | 3 (0.9%) |
|  |  |  |  |  |  |  |  |  |
|  | Film Clips | 3 – 3.5 min | Child Behavior Coding Scheme | Cohen's κ = .61 - 1.0; Cronbach α = .99 |  | Evaluation objectivity through blinded coders | (Dadds et al., 2016; Gottman & Katz, 1989; Hernandez et al., 2019) | 3 (0.9%) |
|  |  |  |  |  |  |  |  |  |
|  | Class Room Observations | 10 min |  | Cohen's κ = .79; Cronbach α = .57 - .96 |  | Evaluation objectivity through blinded coders | (Miller et al., 2004; Onchwari & Keengwe, 2011; Yeh & Li, 2008) | 3 (0.9%) |
|  |  |  |  |  |  |  |  |  |
|  | Children’s Play Therapy Instrument (CPTI) |  |  | ICC = .75 - .97; Cronbach α = .71 - .75 |  | Implementation objectivity through blinded/ trained assessors | (Halfon et al., 2017; Halfon & Bulut, 2019; Halfon et al., 2019) | 3 (0.9%) |
|  |  |  |  |  |  |  |  |  |
|  | Emotional Background Conversations |  |  | Cohen's κ = .79 |  | Evaluation objectivity through blinded coders | (Jones et al., 2014) | 1 (0.3%) |
|  |  |  |  |  |  |  |  |  |
|  | Challenging Situations Task | 30 min |  | Cohen's κ = 0.94 |  | Evaluation objectivity through blinded coders | (Leyva et al., 2020) | 1 (0.3%) |
|  |  |  |  |  |  |  |  |  |
|  | Overheard Conversations | 1 - 2 min |  | Pearson r = .83 - .99 |  |  | (Walden et al., 2012) | 1 (0.3%) |
|  |  |  |  |  |  |  |  |  |
|  | Developmental Testing | 2.5 - 4 h |  | Cronbach α = .82 |  |  | (Jones, 2013) | 1 (0.3%) |
|  |  |  |  |  |  |  |  |  |
|  | Everyday Routines | 3 - 4 h |  | Cohen's κ = .72 - .87 |  |  | (Silkenbeumer et al., 2018) | 1 (0.3%) |
|  |  |  |  |  |  |  |  |  |
|  | Pretend Play |  |  | Cohen's κ = .84 - .92 |  |  | (Galyer & Evans, 2001) | 1 (0.3%) |
|  |  |  |  |  |  |  |  |  |
|  | Hungry Crocodile |  |  | Cohen's κ = .84 - .92 |  |  | (Galyer & Evans, 2001) | 1 (0.3%) |
|  |  |  |  |  |  |  |  |  |
|  | Day-Care Activities |  |  | Cohen's κ = .70 - .83 |  |  | (Kurki et al., 2017) | 1 (0.3%) |
|  |  |  |  |  |  |  |  |  |
|  | Emotional Oddball Task |  |  | Cronbach α = .88; Spearman Brown split half = .76 |  |  | (Clark et al., 2016) | 1 (0.3%) |
|  |  |  |  |  |  |  |  |  |
|  | Mother-Child Interaction | 3 min | Dysregulation Coding System (Hoffman et al., 2006) | ICC = .61 - .90 |  |  | (Norona & Baker, 2017) | 1 (0.3%) |
| **50 to 62 months** | **Frustration/Anger/Disappointment Eliciting Tasks** | | | | | | | |
|  | Delay of Gratification; Preschool Self-Regulation Assessment, (PSRA) | 1 - 10 min | Emotion Regulation Strategy Coding Scheme (Babkirk et al., 2015) | Cohen's κ = .64 - 1.0; ICC = .54 - .90; Cronbach α = .81 - 1.0;  Pearson r = .79 - .90 | Support for the construct and concurrent validity; Discriminant validity: sing. greater sadness during delay than during neutral task (F = 6.01, p < .05) | Evaluation objectivity through blinded coders | (Babkirk et al., 2015; Bailey et al., 2016; Baker et al., 2007; Boldt et al., 2020; Caplan & Baker, 2017; Dennis, Cole et al., 2009; Feldman, 2015; Fung et al., 2020; Green et al., 2023; Harrington, 2021; Housman et al., 2023; Hughes et al., 2015; Kochanska et al., 2009; Myruski et al., 2022; Myruski & Dennis-Tiwary, 2021; Nuske et al., 2018; Power et al., 2016; Ratcliff et al., 2021; Santucci et al., 2008; Silk, Shaw, Forbes et al., 2006; Silk, Shaw, Skuban et al., 2006) | 21 (6.6%) |
|  |  |  |  |  |  |  |  |  |
|  | Disappointing Paradigm (e.g. Box Empty task, Lab Tab) | 10 sec – 3.5 min | Facial Action Coding System; FACS, (Ekman, 1994) | Cohen's κ = .50 – 1.0; ICC = .79 - .98; Cronbach α = .87 - .94;  Agreement: 89% - 91% | Disappointing Paradigm: Emotion regulation strategies in Disappointment Task correlate with mother reported emotion regulation strategies (adaptive: r = .30, p = .046; maladaptive: r = .32, p = .035; convergent validity); Lab-TAB: Moderate to strong convergent validity of the negative emotion elicitation compared to other child observer ratings (r = .25 – .76; r = .27 – .41) | Evaluation objectivity through blinded coders | (Babkirk et al., 2015; Carlson & Wang, 2007; Dennis et al., 2010; Endriga et al., 2003; Forbes, Fox et al., 2006; Forbes, Shaw et al., 2006; Hernandez et al., 2019; Morris et al., 2011; Morris et al., 2010; Nuske et al., 2018; Oeri & Roebers, 2020; Penela et al., 2015; Petersen & Holodynski, 2020; Ren et al., 2016; Ren et al., 2018; Smith, 2001; Spinrad et al., 2004; Stifter et al., 2011; Suarez et al., 2021; Wu & Feng, 2020; Xie et al., 2021) | 21 (6.6%) |
|  |  |  |  |  |  |  |  |  |
|  | Attractive Toy in a Transparent Box (e.g. Lab-TAB) | 3 - 8 min | Dysregulation Coding System (Hoffman et al., 2006) | Cohen's κ = .68 - 1.0; ICC = .69 - .98 | Moderate to strong convergent validity of the negative emotion elicitation compared to other child observer ratings (r = .25 – .76; r = .27 – .41) | Evaluation objectivity through blinded coders | (Baker et al., 2019; Berry et al., 2019; Calkins et al., 1999; Cole et al., 2009; Day & Smith, 2013; Dennis, Cole et al., 2009; Distefano et al., 2021; Dollar et al., 2020; Eisenberg, Fabes, Karbon et al., 1996; Fenning et al., 2018; Gulsrud et al., 2010; Harden et al., 2017; Jahromi et al., 2012; Lee et al., 2023; Ntourou et al., 2013; Nuske et al., 2018; Wu et al., 2020; Zantinge et al., 2017) | 18 (5.6%) |
|  |  |  |  |  |  |  |  |  |
|  | Not Sharing Task (Lab-TAB) | 30 sec - 4 min |  | Cohen's κ = .78 - .80; ICC= .80 - .96; Pearson r = .94 - .99 |  |  | (Dunbar et al., 2022; Graziano et al., 2022; Graziano et al., 2014; Hassan et al., 2023; Landis et al., 2021; Perry, Dollar et al., 2018; Ros & Graziano, 2020) | 7 (2.2%) |
|  |  |  |  |  |  |  |  |  |
|  | Impossibly Perfect Circles task (IPC; LAB-TAB) | 30 sec – 3.5 min |  | Cohen's κ = .60 – 1.0; Pearson r = .94 - .99 | The validity of the task and the coding is demonstrated in previous studies (Calkins & Keane, 2004; Graziano et al., 2011; Mazzer, 2023; Rodríguez et al., 2014; Zimmermann & Stansbury, 2003) |  | (Dennis et al., 2010; Graziano et al., 2022; Graziano et al., 2014; Landis et al., 2021; Mazzer, 2023) | 5 (1.5%) |
|  |  |  |  |  |  |  |  |  |
|  | Clean-Up-Task | 2 - 5 min | Dysregulation Coding System (Hoffman et al., 2006) | Cohen's κ = .70 - .94; ICC = .96 - 1.0; Pearson r = .79 - .90 |  | Evaluation objectivity through blinded coders | (Chan & Neece, 2018; Costa et al., 2019; Feldman, 2015) | 3 (0.9%) |
|  |  |  |  |  |  |  |  |  |
|  | Toy Behind Barrier task | 1.5 min |  | Cohen's κ = .83 - .87; Krippendorf’s α = .80 - 1.0 |  | Implementation and evaluation objectivity through blinded assessors and coders | (Costa et al., 2019; Murnan et al., 2021) | 2 (0.6%) |
|  |  |  |  |  |  |  |  |  |
|  | Toy Removal Task | 2 min |  | ICC = .70 - .91 |  | Evaluation objectivity through blinded coders | (Zeytinoglu et al., 2022) | 1 (0.3%) |
|  |  |  |  |  |  |  |  |  |
|  | No Stickers Left Task (Lab-TAB) | 2 min |  | Cohen's κ = .76 - 1.0 |  | Evaluation objectivity through blinded coders | (Wu & Feng, 2020) | 1 (0.3%) |
|  |  |  |  |  |  |  |  |  |
|  | Frustration Task according to Montessori button frame | 3 min |  | ICC > .70 |  |  | (Helmsen, 2011) | 1 (0.3%) |
|  |  |  |  |  |  |  |  |  |
|  | Still-Face Paradigm |  |  | Cohen's κ = .96 - .98 |  |  | (Maclean et al., 2014) | 1 (0.3%) |
|  |  |  |  |  |  |  |  |  |
|  | Non-Opening Bottle | 30 - 60 sec. |  |  |  |  | (Wiefferink et al., 2012) | 1 (0.3%) |
|  |  |  |  |  |  |  |  |  |
|  | Inter Adult Anger | 15 min |  | ICC = .70 - .93 |  |  | (Maughan et al., 2007)^341^ | 1 (0.3%) |
|  |  |  |  |  |  |  |  |  |
|  | Balloons Game | 2 - 6 min | Facial Action Coding System (FACS; (Ekman, 1994) |  |  | Evaluation objectivity through blinded coders | (Roy et al., 2013)^409^ | 1 (0.3%) |
|  |  |  |  |  |  |  |  |  |
|  | High Chair (Lab-TAB) | 5 min |  | Cohen's κ = .83 - 1.0 |  |  | (Hill et al., 2006)^276^ | 1 (0.3%) |
|  |  |  |  |  |  |  |  |  |
|  | Child waiting quietly | 3 min |  |  |  | Evaluation objectivity through blinded coders | (Arnold et al., 2011)^340^ | 1 (0.3%) |
|  | **Fear Eliciting Tasks** | | | | | | | |
|  | Masks (Lab-TAB) | 15 - 30 sec |  | Cohen's κ = .40 – .94; ICC = .86;  Pearson r = .80 – .94 |  | Evaluation objectivity through blinded coders | (Abraham et al., 2018; Hirschler-Guttenberg, Feldman et al., 2015; Hirschler-Guttenberg, Golan et al., 2015) | 3 (0.9%) |
|  |  |  |  |  |  |  |  |  |
|  | Unpredictable Mechanical Toy (Lab-TAB) |  |  | Agreement: 70.51 – 89.10 % | Moderate to strong convergent validity of the negative emotion elicitation compared to other child observer ratings (r = .25 – .76; r = .27 – .41) | Evaluation objectivity through blinded coders | (Nuske et al., 2018) | 1 (0.3%) |
|  | **Jealousy Eliciting Tasks** | | | | | | | |
|  | Triadic Interaction Paradigm | 9 min |  | Cohen's κ = .70 |  |  | (Miller et al., 2000)^240^ | 1 (0.3%) |
|  | **Joy Eliciting Tasks** | | | | | | | |
|  | Popping Bubbles (Lab-TAB) | 2 min |  | ICC = .89 - .98 |  | Evaluation objectivity through blinded coders | (Abraham et al., 2018; Harden et al., 2017) | 2 (0.6%) |
|  |  |  |  |  |  |  |  |  |
|  | Secret Keeping Task | 15 sec – 3.5 min |  | Cohen's κ = .61 - 1.0 |  | Evaluation objectivity through blinded coders | (Carlson & Wang, 2007; Hernandez et al., 2019)^364,393^ | 2 (0.6%) |
|  | **Non Specific/ Mixed Emotion Eliciting Tasks** | | | | | | | |
|  | Preschool Self-Regulation Assessment (PSRA): Balance Beam, Pencil Tap | 3 min |  | ICC = .73 - .99; Cronbach α = .87 | Support for the construct and concurrent validity across different populations (Smith-Donald et al., 2007) |  | ^76,375,410^ ^411^(Bailey et al., 2016; Bailey et al., 2022; Housman et al., 2023; Mascheroni et al., 2022; McCoy & Raver, 2011; Provenzi et al., 2020) | 6 (1.8%) |
|  |  |  |  |  |  |  |  |  |
|  | Toy Sort Task | 10 min |  | Cohen's κ = .69 - 1.0; ICC = .73 - .99; Cronbach α = .87 |  |  | (Bailey et al., 2016; Bailey et al., 2022; Kalpidou et al., 2004; McCoy & Raver, 2011)^45,338,350,375^ | 4 (1.2%) |
|  |  |  |  |  |  |  |  |  |
|  | Puzzle Task | 2.5 - 5 min | Dysregulation Coding System (Hoffman et al., 2006) | Cohen's κ = .81 - 1.0; ICC = .90; Pearson r = .97 - .98 |  | Evaluation objectivity through blinded coders | (Baker et al., 2019; Nancy Eisenberg et al., 2003; Fenning et al., 2018; Jahromi et al., 2012) | 4 (1.2%) |
|  |  |  |  |  |  |  |  |  |
|  | Problem Solving Task | 3 - 10 min | Emotion Dysregulation subscale (Cole et al., 1994); Dysregulation coding system (Hoffman et al., 2006) | ICC = .79 - .90; Pearson r = .79 - .90 |  | Evaluation objectivity through blinded coders | (Caplan & Baker, 2017; Norona & Baker, 2014; Noroña et al., 2018)^90,346,389^ | 3 (0.9%) |
|  |  |  |  |  |  |  |  |  |
|  | Film Clips | 3 – 3.5 min | Child Behavior Coding Scheme | Cohen's κ = .61 - 1.0; Cronbach α = .99 |  | Evaluation objectivity through blinded coders | (Dadds et al., 2016; Gottman & Katz, 1989; Hernandez et al., 2019)^62,364,378^ | 3 (0.9%) |
|  |  |  |  |  |  |  |  |  |
|  | Children’s Play Therapy Instrument (CPTI) |  |  | ICC = .75 - .97; Cronbach α = .71 - .75 |  | Implementation objectivity through blinded/ trained assessors | (Halfon et al., 2017; Halfon & Bulut, 2019; Halfon et al., 2019)^43,381,382^ | 3 (0.9%) |
|  |  |  |  |  |  |  |  |  |
|  | Class Room Observations | 10 min |  | Cronbach α = .57 - .96 |  |  | ^352,379,380^(Miller et al., 2004; Onchwari & Keengwe, 2011; Yeh & Li, 2008) | 3 (0.9%) |
|  |  |  |  |  |  |  |  |  |
|  | Free Play |  |  | Cohen's κ = .72 - .96 |  |  | (Garner, 2006; Garner & Spears, 2000; Silkenbeumer et al., 2018) | 3 (0.9%) |
|  |  |  |  |  |  |  |  |  |
|  | Parent-Child-Interaction (e.g. Boxes procedure) | 5 - 10 min | Dysregulation Coding System (Hoffman et al., 2006) | Cohen's κ = 0.82; ICC = .96 - 1.0 |  | Evaluation objectivity through blinded coders | (Chan & Neece, 2018; Valentovich et al., 2018)^407,413^ | 2 (0.6%) |
|  |  |  |  |  |  |  |  |  |
|  | Buzz Wire Assessment | 3 min |  | ICC = .76 - .94; Cronbach α = .68 |  | Evaluation objectivity through blinded coders | (Halligan et al., 2013)^182^ | 1 (0.3%) |
|  |  |  |  |  |  |  |  |  |
|  | Stranger Approach |  |  | Agreement: 70.51 – 89.10 % | Moderate to strong convergent validity of the negative emotion elicitation compared to other child observer ratings (r = .25 – .76; r = .27 – .41; | Evaluation objectivity through blinded coders | (Nuske et al., 2018) | 1 (0.3%) |
|  |  |  |  |  |  |  |  |  |
|  | Emotional Oddball Task |  |  | Cronbach α = .88; Spearman Brown split half = .76 |  |  | (Clark et al., 2016) | 1 (0.3%) |
|  |  |  |  |  |  |  |  |  |
|  | Motor Task |  |  | Cohen's κ = .59 - .76 |  |  | (Forbes, Fox et al., 2006) | 1 (0.3%) |
|  |  |  |  |  |  |  |  |  |
|  | Challenging Situations Task | 30 min |  | Cohen's κ = 0.94 |  | Evaluation objectivity through blinded coders | (Leyva et al., 2020)^384^ | 1 (0.3%) |
|  |  |  |  |  |  |  |  |  |
|  | Pretend Play |  |  | Cohen's κ = .84 - .92 |  |  | (Galyer & Evans, 2001)^387^ | 1 (0.3%) |
|  |  |  |  |  |  |  |  |  |
|  | Entry Situation | 6 min |  | Cohen's κ = .73 - .84 |  |  | (Wilson, 1999) | 1 (0.3%) |
|  |  |  |  |  |  |  |  |  |
|  | Mother and child discussing emotional salient events | 60 - 90 min |  | Cohen's κ = .79 |  |  | (Waters et al., 2010)^415^ | 1 (0.3%) |

**Table 1d**

*Behavioral observation methods of emotion regulation for school aged children (63 to 140 months)*

| **Age Range (months)** | | **Task** | **Duration (sec/min/h)** | | **Coding Manual** | **Reliability** | **Validity** | **Objectivity** | **Studies using this task** | **Number/ Percentage of studies using task** |
| --- | --- | --- | --- | --- | --- | --- | --- | --- | --- | --- |
| **School** | | | | | | | | | | |
| **63 to 75 months** | **Frustration/Anger/Disappointment Eliciting Tasks** | | | | | | | | | |
|  | Disappointing Paradigm | | 1 - 5 min | Facial Action CodingSystem; FACS (Ekman, 1994) | | Cohen's κ = .62 - .89; ICC = .98;  Cronbach α = .87 - .94 | Emotion regulation strategies in Disappointment Task correlate with mother reported emotion regulation strategies (adaptive: r = .30, p = .046; maladaptive: r = .32, p = .035; convergent validity); Discriminant validity: sing. greater sadness during delay than during neutral task (F = 6.01, p < .05) | Evaluation objectivity through blinded coders | (Babkirk et al., 2015; Carlson & Wang, 2007; Forbes, Fox et al., 2006; Forbes, Shaw et al., 2006; Morris et al., 2011; Nakamichi, 2017; Petersen & Holodynski, 2020; Ren et al., 2016; Ren et al., 2018; Spinrad et al., 2004; Xie et al., 2021)^80,127,129,333,363,365,366,368,390,393,394,416^ | 12 (3.7%) |
|  |  | |  |  | |  |  |  |  |  |
|  | Delay of Gratification; Preschool Self-Regulation Assessment, PSRA | | 3 - 7 min | Emotion Regulation Strategy Coding Scheme (Babkirk et al., 2015) | | Cohen's κ = .64 - 1.0; ICC = .79;  Cronbach α = .81 - 1.0;  Krippendorf’s α = .62 - .99 |  | Evaluation objectivity through blinded coders | ^45,89,154,326,338,353,356–358,390,391^(Baker et al., 2019; Berry et al., 2019; Day & Smith, 2013; Distefano et al., 2021; Dollar et al., 2020; Fenning et al., 2018; Harden et al., 2017; Jahromi et al., 2012; Ntourou et al., 2013; Zantinge et al., 2017) | 10 (3.1%) |
|  |  | |  |  | |  |  |  |  |  |
|  | Attractive Toy in a Transparent Box (e.g. Lab-TAB) | | 3 - 10 min | Dysregulation Coding System (Hoffman et al., 2006) | | Cohen's κ = .66 - 1.0; ICC = .69 - .98 | Good construct and predictive validity across a range of studies | Evaluation objectivity through blinded coders | (Babkirk et al., 2015; Bailey et al., 2016; Bailey et al., 2022; Boldt et al., 2020; Myruski et al., 2022; Myruski & Dennis-Tiwary, 2021; Silk, Shaw, Forbes et al., 2006; Silk, Shaw, Skuban et al., 2006; Snyder & Arnold, 2022)^85,88,111,327,332,359–362,400^ | 10 (3.1%) |
|  |  | |  |  | |  |  |  |  |  |
|  | Not Sharing Task (Lab-TAB) | | 1.5 sec - 5 min |  | | Cohen's κ > .80;  ICC= .82 - .98;  Pearson r = .94 - .99 |  |  | (Graziano & Hart, 2016; Graziano et al., 2014; Hassan et al., 2023; Hernandez et al., 2019; Kim et al., 2023) | 5 (1.5%) |
|  |  | |  |  | |  |  |  |  |  |
|  | Impossibly Perfect Circles task (IPC; LAB-TAB) | | 3.5 min |  | | Cohen's κ = .60 – 1.0; Pearson r = .94 - .99 | The validity of the task and the coding is demonstrated in previous studies (Calkins & Keane, 2004; Graziano et al., 2011; Mazzer, 2023; Rodríguez et al., 2014; Zimmermann & Stansbury, 2003) |  | (Graziano & Hart, 2016; Graziano et al., 2014; Kahle et al., 2021; Mazzer, 2023)^272,339,364,401,417^ | 4 (1.2%) |
|  |  | |  |  | |  |  |  |  |  |
|  | Toy Behind Barrier task | |  |  | | Cohen's κ = .83 - .87 |  |  | (Costa et al., 2019)^337^ | 1 (0.3%) |
|  | Toy Removal Task | | 2 min |  | | ICC = .70 - .91 |  | Evaluation objectivity through blinded coders | (Zeytinoglu et al., 2022)^408^ | 1 (0.3%) |
|  |  | |  |  | |  |  |  |  |  |
|  | Frustration Task according to Montessori button frame | | 3 min |  | | ICC > .70 |  |  | (Helmsen, 2011)^371^ | 1 (0.3%) |
|  |  | |  |  | |  |  |  |  |  |
|  | Inter Adult Anger | | 15 min |  | | ICC = .70 - .93 |  |  | (Maughan et al., 2007) | 1 (0.3%) |
|  |  | |  |  | |  |  |  |  |  |
|  | Balloons Game | | 2 - 6 min | Facial Action Coding System (FACS; (Ekman, 1994) | |  |  | Evaluation objectivity through blinded coders | (Roy et al., 2013)^409^ | 1 (0.3%) |
|  |  | |  |  | |  |  |  |  |  |
|  | Impossible Timed Game | | 5 min | System for Coding Affect Regulation in the Family (SCARF; (Lindahl, 1998) | | ICC = .68 - .84; Cronbach α = .75 - .88 |  | Evaluation objectivity through blinded coders | (Klinnert et al., 2000)^418^ | 1 (0.3%) |
|  |  | |  |  | |  |  |  |  |  |
|  | Age-appropriate Lego model with missing pieces | | as much time as they needed |  | | Cronbach α = .79 - .93; Pearson r = .80 |  | Evaluation objectivity through blinded coders | (Melnick & Hinshaw, 2000)^419^ | 1 (0.3%) |
|  |  | |  |  | |  |  |  |  |  |
|  | Impossible Building Tower Task | | 2 - 4 min | Software Eudico Linguistic Annotator (ELAN;(Wittenburg et al., 2006) | | Krippendorf’s α = .71 - .79 | The measurement models of the validation constructs all showed a very good fit with the data (all RMSEAs < 0.05, SRMRs < 0.02, CFIs > 0.99) | Evaluation objectivity through blinded coders | (Rohlf & Krahé, 2015) | 1 (0.3%) |
|  |  | |  |  | |  |  |  |  |  |
|  | Clean-Up Task | |  |  | | Cohen's κ = .71 - .83 |  | Implementation objectivity through standardized/blinded observations procedures | (Zimmermann & Stansbury, 2004)^420^ | 1 (0.3%) |
|  |  | |  |  | |  |  |  |  |  |
|  | Child waiting quietly | | 3 min |  | |  |  | Evaluation objectivity through blinded coders | (Arnold et al., 2011) | 1 (0.3%) |
|  | **Fear Eliciting Tasks** | | | | | | | | | |
|  | Masks (Lab-TAB) | | 15 - 30 sec |  | | Cohen's κ = .40 – .94; ICC = .86;  Pearson r = .80 – .94 |  | Evaluation objectivity through blinded coders | (Abraham et al., 2018; Hirschler-Guttenberg, Feldman et al., 2015; Hirschler-Guttenberg, Golan et al., 2015)^47,342,343^ | 3 (0.9%) |
|  |  | |  |  | |  |  |  |  |  |
|  | Brief interview regarding parent-child separations | | 5 min | System for Coding Affect Regulation in the Family (SCARF; (Lindahl, 1998) | | ICC = .68 - .84; Cronbach α = .75 - .88 |  | Evaluation objectivity through blinded coders | (Klinnert et al., 2000)^418^ | 1 (0.3%) |
|  | **Jealousy Eliciting Tasks** | | | | | | | | | |
|  | Triadic Interaction Paradigm | | 9 min |  | | Cohen's κ = .70 |  |  | (Miller et al., 2000)^240^ | 1 (0.3%) |
|  | **Joy Eliciting Tasks** | | | | | | | | | |
|  | Popping Bubbles (Lab-TAB; Goldsmith et al., 1996) | | 2 min |  | | ICC = .89 - .98 | Good construct and predictive validity across a range of studies | Evaluation objectivity through blinded coders | (Abraham et al., 2018; Harden et al., 2017) | 2 (0.6%) |
|  |  | |  |  | |  |  |  |  |  |
|  | Secret Keeping Task | | 15 sec |  | | Cohen's κ > .90 |  | Evaluation objectivity through blinded coders | (Carlson & Wang, 2007)^393^ | 1 (0.3%) |
|  |  | |  |  | |  |  |  |  |  |
|  | Playing a game with the experimenter | | 5 min | System for Coding Affect Regulation in the Family (SCARF; (Lindahl, 1998) | | ICC = .68 - .84; Cronbach α = .75 - .88 |  | Evaluation objectivity through blinded coders | (Klinnert et al., 2000) | 1 (0.3%) |
|  | **Non Specific/ Mixed Emotion Eliciting Tasks** | | | | | | | | | |
|  | Puzzle Task | | 2,5 - 5 min | Dysregulation Coding System (Hoffman et al., 2006) | | Cohen's κ = .81 - 1.0; ICC = .39 - .92; Pearson r = .97 - .98 | Convergent validity: Weak corr. with Emotion Control Scale (*r* = −.20, p = .079); medium corr. with DESR (*r* = −.28, *p* = .016); Discriminant validity: medium, corr. with total IQ (*r* = .30, *p* = .011) weak corr. with verbal comprehension (*r* = .27, *p* = .026), and perceptual reasoning (*r* = .28, *p* = .018); Validity supported by prior studies of preschool- and school-aged children with and without neurodevelopmental disorders (Baker et al., 2007; Hoffman et al., 2006; Norona & Baker, 2014) | Evaluation objectivity through blinded coders | (Baker et al., 2019; Dunbar et al., 2022; Nancy Eisenberg et al., 2003; Fenning et al., 2018; Jahromi et al., 2012; Walcott & Landau, 2004)^88,327,360,405,412,421^ | 7 (2.2%) |
|  |  | |  |  | |  |  |  |  |  |
|  | Class Room Observations | | 15 min - 20 h | California chi Q-sort (CCQ; (Block & Block, 1980) | | Cohen's κ = .76;  ICC = .93 |  |  | (Fernandes et al., 2021; Kurki et al., 2015; Meroño & Ventura, 2022; Yeh & Li, 2008)^379,422–424^ | 4 (1.2%) |
|  |  | |  |  | |  |  |  |  |  |
|  | Preschool Self-Regulation Assessment (PSRA): Balance Beam; Pencil Tap | |  |  | | ICC > .72 | Support for construct and concurrent validity of the measure |  | (Bailey et al., 2016; Bailey et al., 2022; Housman et al., 2023)^45,76,338^ | 3 (0.9%) |
|  |  | |  |  | |  |  |  |  |  |
|  | Children’s Play Therapy Instrument (CPTI) | |  |  | | ICC = .75 - .97; Cronbach α = .71 - .75 |  | Implementation objectivity through standardized/blinded observations procedures | (Halfon et al., 2017; Halfon & Bulut, 2019; Halfon et al., 2019)^43,381,382^ | 3 (0.9%) |
|  |  | |  |  | |  |  |  |  |  |
|  | Free Play | |  |  | | Cohen's κ = .72 - .87 |  |  | (Garner & Spears, 2000; Silkenbeumer et al., 2018) | 2 (0.6%) |
|  |  | |  |  | |  |  |  |  |  |
|  | Toy Sort | |  |  | | ICC > .72 |  |  | (Bailey et al., 2016; Bailey et al., 2022)^45,338^ | 2 (0.6%) |
|  |  | |  |  | |  |  |  |  |  |
|  | Problem Solving Task | | 10 min | Emotion Dysregulation subscale (Cole et al., 1994); Dysregulation coding system (Hoffman et al., 2006) | | ICC = .79 - .90; Pearson r = .79 - .90 |  | Evaluation objectivity through blinded coders | (Norona & Baker, 2014; Noroña et al., 2018)^90,348^ | 2 (0.6%) |
|  |  | |  |  | |  |  |  |  |  |
|  | Virtually Impossible Dexterity Task | | 2 - 4 min |  | | Krippendorf’s α = .71 - .99 | Structural equation models testing concurrent and predictive validity showed good to adequate model fit, with .912< CFI < .99 and .035 < RMSEA < .073: (Kirsch et al., 2019) |  | (Kirsch et al., 2019; Kirsch et al., 2015)^87,425^ | 2 (0.6%) |
|  |  | |  |  | |  |  |  |  |  |
|  | Structured Play, Mother-Child Interaction | |  |  | | Cohen's κ = .71 - .83 |  | Implementation objectivity through standardized observations procedures | (Zimmermann & Stansbury, 2004)^230^ | 1 (0.3%) |
|  |  | |  |  | |  |  |  |  |  |
|  | Emotional Oddball Task | |  |  | | Cronbach α = .88; Spearman Brown split half = .76 |  |  | (Clark et al., 2016) | 1 (0.3%) |
|  |  | |  |  | |  |  |  |  |  |
|  | Challenge Task | | 3 min | Dysregulation Coding System (Baker et al., 2007) | | ICC = .91 |  | Evaluation objectivity through blinded coders | (Baker et al., 2022) | 1 (0.3%) |
|  |  | |  |  | |  |  |  |  |  |
|  | Collaborative Task (parent and child) | | 4 min |  | | ICC = .94 |  |  | (Jin et al., 2017)^427^ | 1 (0.3%) |
|  |  | |  |  | |  |  |  |  |  |
|  | Emotional Background Conversations | |  |  | | Cohen's κ = .79 |  | Evaluation objectivity through blinded coders | (Jones et al., 2017)^383^ | 1 (0.3%) |
|  |  | |  |  | |  |  |  |  |  |
|  | Entry Situation | | 6 min |  | | Cohen's κ = .73 - .84 |  |  | (Wilson, 1999)^414^ | 1 (0.3%) |
|  |  | |  |  | |  |  |  |  |  |
|  | Film Clips | | 3 min | Child Behaviour Coding Scheme | | Cronbach α = .99 |  | Evaluation objectivity through blinded coders | (Dadds et al., 2016)^378^ | 1 (0.3%) |
|  |  | |  |  | |  |  |  |  |  |
|  | Cognitive Task: Circling letters | | 5 min | System for Coding Affect Regulation in the Family (SCARF; (Lindahl, 1998) | | ICC = .68 - .84; Cronbach α = .75 - .88 |  | Evaluation objectivity through blinded coders | (Klinnert et al., 2000) | 1 (0.3%) |
|  |  | |  |  | |  |  |  |  |  |
|  | Tangram Construction Task | | 5 min | Tangram Emotion Coding Manual (TEC-M; (Hagstrøm et al., 2019) | | ICC = .79 |  | Implementation objectivity through blinded observations procedures | (Spang et al., 2022)^428^ | 1 (0.3%) |
| **76 to 88 months** | **Frustration/Anger/Disappointment Eliciting Tasks** | | | | | | | | | |
|  | Delay of Gratification | | 10 min | Emotion Regulation Strategy Coding Scheme (Babkirk et al., 2015; Gilliom et al., 2002) | | Cohen's κ = .66 - 1.0 | Discriminant validity: sing. greater sadness during delay than during neutral task (F = 6.01, p < .05) | Evaluation objectivity through blinded coders | (Babkirk et al., 2015; Myruski et al., 2022; Myruski & Dennis-Tiwary, 2021; Santucci et al., 2008; Silk, Shaw, Forbes et al., 2006; Silk, Shaw, Skuban et al., 2006)^154,356,358,390,391^ | 6 (1.8%) |
|  |  | |  |  | |  |  |  |  |  |
|  | Attractive Toy in a Transparent Box | | 3 - 4 min | Dysregulation Coding System (Hoffman et al., 2006) | | Cohen's κ = .78 - .90; ICC = .69 - .90 |  | Evaluation objectivity through blinded coders | (Baker et al., 2019; Distefano et al., 2021; Fenning et al., 2018; Kuiper et al., 2023; Zantinge et al., 2017)^88,97,359,360,362^ | 5 (1.5%) |
|  |  | |  |  | |  |  |  |  |  |
|  | Disappointing Paradigm | | 3 min |  | | Cohen's κ = .66 - 1.0; Cronbach α = .87 - .94 |  |  | (Babkirk et al., 2015; Forbes, Fox et al., 2006; Forbes, Shaw et al., 2006; Morris et al., 2011; Petersen & Holodynski, 2020)^127,129,363,365,390^ | 5 (1.5%) |
|  |  | |  |  | |  |  |  |  |  |
|  | Virtually Impossible Dexterity Task | | 2 - 4 min |  | | Krippendorf’s α = .71 - .99 | Structural equation models testing concurrent and predictive validity showed good to adequate model fit, with .912< CFI < .99 and .035 < RMSEA < .073; Kirsch et al., 2019 |  | (Kirsch et al., 2019; Kirsch et al., 2015) | 2 (0.6%) |
|  |  | |  |  | |  |  |  |  |  |
|  | Toy Behind Barrier task | |  |  | | Cohen's κ = .83 - .87 |  |  | (Costa et al., 2019)^337^ | 1 (0.3%) |
|  |  | |  |  | |  |  |  |  |  |
|  | Impossibly Perfect Circles task (IPC; LAB-TAB) | | 3.5 min |  | | Cohen's κ = .60–1.0 | The validity of the task and the coding is demonstrated in previous studies (Calkins & Keane, 2004; Graziano et al., 2011; Mazzer, 2023; Rodríguez et al., 2014; Zimmermann & Stansbury, 2003) |  | (Mazzer, 2023)^339^ | 1 (0.3%) |
|  |  | |  |  | |  |  |  |  |  |
|  | Toy Behind Barrier Task | | 1.5 min |  | | Krippendorf’s α = .80 - 1.0 |  | Implementation objectivity through standardized observations procedures | (Murnan et al., 2021) | 1 (0.3%) |
|  |  | |  |  | |  |  |  |  |  |
|  | Frustration Task according to Montessori button frame | | 3 min |  | | ICC > .70 |  |  | (Helmsen, 2011)^371^ | 1 (0.3%) |
|  |  | |  |  | |  |  |  |  |  |
|  | Balloons Game | | 2 - 6 min | Facial Action Coding System (FACS; (Ekman, 1994) | |  |  | Evaluation objectivity through blinded coders | (Roy et al., 2013)^409^ | 1 (0.3%) |
|  |  | |  |  | |  |  |  |  |  |
|  | Challenge Task | | 3 min | Dysregulation Coding System (Baker et al., 2007)) | | ICC = .91 |  | Evaluation objectivity through blinded coders | (Baker et al., 2022) | 1 (0.3%) |
|  |  | |  |  | |  |  |  |  |  |
|  | Collaborative Task (parent and child) | | 4 min |  | | ICC = .94 |  |  | ^427^(Jin et al., 2017) | 1 (0.3%) |
|  |  | |  |  | |  |  |  |  |  |
|  | Age-appropriate Lego model with missing pieces | | as much time as they needed |  | | Cronbach α = .79 - .93;  Pearson r = .80 |  | Evaluation objectivity through blinded coders | (Melnick & Hinshaw, 2000)^419^ | 1 (0.3%) |
|  |  | |  |  | |  |  |  |  |  |
|  | Impossible Building Tower Task | | 2 - 4 min | Software Eudico Linguistic Annotator (ELAN;(Wittenburg et al., 2006) | | Krippendorf’s α = .71 - .79 | The measurement models of the validation constructs showed a very good fit with the data RMSEAs < 0.05, SRMRs < 0.02, CFIs > 0.99 | Evaluation objectivity through blinded coders | (Rohlf & Krahé, 2015)^425^ | 1 (0.3%) |
|  |  | |  |  | |  |  |  |  |  |
|  | Conflict Discourse Task | | 3 min |  | | ICC = .66 |  |  | (Hurrell et al., 2017)^429^ | 1 (0.3%) |
|  |  | |  |  | |  |  |  |  |  |
|  | Impossible Timed Game | | 5 min | System for Coding Affect Regulation in the Family (SCARF; (Lindahl, 1998) | | ICC = .68 - .84; Cronbach α = .75 - .88 |  | Evaluation objectivity through blinded coders | (Klinnert et al., 2000) | 1 (0.3%) |
|  | **Fear Eliciting Tasks** | | | | | | | | | |
|  | Masks (Lab-TAB) | | 15 sec |  | | ICC = .86 |  |  | (Abraham et al., 2018) | 1 (0.3%) |
|  |  | |  |  | |  |  |  |  |  |
|  | Brief interview regarding parent-child separations | | 5 min | System for Coding Affect Regulation in the Family (SCARF; (Lindahl, 1998) | | ICC = .68 - .84; Cronbach α = .75 - .88 |  | Evaluation objectivity through blinded coders | (Klinnert et al., 2000)^418^ | 1 (0.3%) |
|  | **Joy Eliciting Tasks** | | | | | | | | | |
|  | Popping Bubbles (Lab-TAB) | | 2 min |  | | ICC = .89 | Good construct and predictive validity across a range of studies (Harden et al., 2017) | Evaluation objectivity through blinded coders | (Abraham et al., 2018; Harden et al., 2017) | 2 (0.6%) |
|  |  | |  |  | |  |  |  |  |  |
|  | Playing a game with the experimenter | | 5 min | System for Coding Affect Regulation in the Family (SCARF; (Lindahl, 1998) | | ICC = .68 - .84; Cronbach α = .75 - .88 |  | Evaluation objectivity through blinded coders | (Klinnert et al., 2000)^418^ | 1 (0.3%) |
|  | **Non Specific/ Mixed Emotion Eliciting Tasks** | | | | | | | | | |
|  | Puzzle Task; Tangram Construction Task; Regulation Puzzle Box Task | | 2.5 - 5 min | Dysregulation Coding System (Hoffman et al., 2006); Emotion Regulation Score | | Cohen's κ = .81 - 1.0; ICC = .39 - .92; Pearson r = .93 - .98 | Convergent validity: Weak corr. with Emotion Control Scale (*r* = −.20, p = .079); medium corr. with DESR (*r* = −.28, *p* = .016); Discriminant validity: medium, corr. with total IQ (*r* = .30, *p* = .011) weak corr. with verbal comprehension (*r* = .27, *p* = .026), and Perceptual Reasoning (*r* = .28, *p* = .018); Validity supported by prior studies of preschool- and school-aged children with and without neurodevelopmental disorders (Baker et al., 2007; Hoffman et al., 2006; Norona & Baker, 2014) | Evaluation objectivity through blinded coders | (Baker et al., 2019; Eisenberg & Morris, 2003; N. Eisenberg et al., 2000; Fenning et al., 2018; Hagstrøm et al., 2019; Hagstrøm et al., 2021; Jahromi et al., 2012; Walcott & Landau, 2004)^70,71,88,327,360,412,421,430^ | 8 (2.5%) |
|  |  | |  |  | |  |  |  |  |  |
|  | Children’s Play Therapy Instrument (CPTI) | |  |  | | ICC = .75 - .97; Cronbach α = .71 - .75 |  | Implementation objectivity through blinded observations procedures | (Halfon et al., 2017; Halfon & Bulut, 2019; Halfon et al., 2019)^43,381,382^ | 3 (0.9%) |
|  |  | |  |  | |  |  |  |  |  |
|  | Class Room Observations | | 15 - 20 min |  | | Cohen's κ = .76 |  |  | (Kurki et al., 2015; Meroño & Ventura, 2022) | 2 (0.6%) |
|  |  | |  |  | |  |  |  |  |  |
|  | Free Play | |  |  | | Cohen's κ = .72 - .87 |  |  | (Silkenbeumer et al., 2018)^377^ | 1 (0.3%) |
|  |  | |  |  | |  |  |  |  |  |
|  | Cognitive Task: Circling letters | | 5 min | System for Coding Affect Regulation in the Family (SCARF; (Lindahl, 1998) | | ICC = .68 - .84; Cronbach α = .75 - .88 |  | Evaluation objectivity through blinded coders | (Klinnert et al., 2000)^418^ | 1 (0.3%) |
|  |  | |  |  | |  |  |  |  |  |
|  | Tangram Construction Task | | 5 min | Tangram Emotion Coding Manual (TEC-M(Hagstrøm et al., 2019) | | ICC = .79 |  | Evaluation objectivity through blinded coders | (Spang et al., 2022)^428^ | 1 (0.3%) |
|  |  | |  |  | |  |  |  |  |  |
|  | Mother-Child Interaction | | 3 min | Dysregulation Coding System (Hoffman et al., 2006) | | ICC = .61 - .90 |  |  | (Norona & Baker, 2017)^346^ | 1 (0.3%) |
|  |  | |  |  | |  |  |  |  |  |
|  | Problem Solving Task | | 3 min | Dysregulation Coding System (Hoffman et al., 2006); Emotion Dysregulation subscale (Cole et al., 1994) | | ICC = .79 - .90 |  |  | (Norona & Baker, 2014) | 1 (0.3%) |
|  |  | |  |  | |  |  |  |  |  |
|  | Regulating Taste Experience | |  |  | |  |  |  | (Hoffmann et al., 2015) | 1 (0.3%) |
|  |  | |  |  | |  |  |  |  |  |
|  | Conflict Discourse Task | | 3 min |  | | ICC = .66 |  |  | (Hurrell et al., 2017)^429^ | 1 (0.3%) |
| **89 to 101 months** | **Frustration/Anger/Disappointment Eliciting Tasks** | | | | | | | | | |
|  | Disappointing Paradigm | |  |  | | Cohen's κ = .62 - .89 |  |  | (Forbes, Fox et al., 2006; Forbes, Shaw et al., 2006; Morris et al., 2011)^127,129,365^ | 3 (0.9%) |
|  |  | |  |  | |  |  |  |  |  |
|  | Attractive Toy in a Transparent Box (Lab-TAB) | |  | Dysregulation Coding System (Hoffman et al., 2006) | | ICC = .90 |  | Evaluation objectivity through blinded coders | (Baker et al., 2019; Fenning et al., 2018)^88,360^ | 2 (0.6%) |
|  |  | |  |  | |  |  |  |  |  |
|  | Delay of Gratification (cookie task) | | 3 - 10 min |  | | Cohen's κ = .64 - .79 | Predictive validity: Focus on cookie and less active distraction associated with higher rates of externalizing problems at age 6 |  | (Myruski & Dennis-Tiwary, 2021; Trentacosta & Shaw, 2009)^391,432^ | 2 (0.6%) |
|  |  | |  |  | |  |  |  |  |  |
|  | Balloons Game | | 2 - 6 min | Facial Action Coding System (FACS; (Ekman, 1994) | |  |  | Evaluation objectivity through blinded coders | (Roy et al., 2013) | 1 (0.3%) |
|  |  | |  |  | |  |  |  |  |  |
|  | Challenge Task | | 3 min | Dysregulation Coding System (Baker et al., 2007) | | ICC = .91 |  | Evaluation objectivity through blinded coders | (Baker et al., 2022)^426^ | 1 (0.3%) |
|  |  | |  |  | |  |  |  |  |  |
|  | Toy Behind Barrier task | |  |  | | Cohen's κ = .83 - .87 |  |  | (Costa et al., 2019)^337^ | 1 (0.3%) |
|  |  | |  |  | |  |  |  |  |  |
|  | Virtually Impossible Dexterity Task | | 2 - 4 min |  | | Krippendorf’s α = .71 - .99 | Structural equation models testing concurrent and predictive validity showed good to adequate model fit, with .912< CFI < .99 and .035 < RMSEA < .073: |  | (Kirsch et al., 2019) | 1 (0.3%) |
|  |  | |  |  | |  |  |  |  |  |
|  | Age-appropriate Lego model with missing pieces | | as much time as they needed |  | | Cronbach α = .79 - .93; Pearson r = .80 |  | Evaluation objectivity through blinded coders | (Melnick & Hinshaw, 2000)^419^ | 1 (0.3%) |
|  |  | |  |  | |  |  |  |  |  |
|  | Impossible Building Tower Task | | 2 - 4 min | Software Eudico Linguistic Annotator (ELAN; (Wittenburg et al., 2006)) | | Krippendorf’s α = .71 - .79 | The measurement models of the validation constructs all showed a very good fit with the data (all RMSEAs < 0.05, SRMRs < 0.02, CFIs > 0.99 | Evaluation objectivity through blinded coders | (Rohlf & Krahé, 2015) | 1 (0.3%) |
|  |  | |  |  | |  |  |  |  |  |
|  | Conflict Discourse Task | | 3 min |  | | ICC = .66 |  |  | (Hurrell et al., 2017)^429^ | 1 (0.3%) |
|  |  | |  |  | |  |  |  |  |  |
|  | Computerized Mirror Tracing Persistence Task (MTPT) | | 6 - 13 min | Emotion Dysregulation Inventory-Short Form (EDI; (Mazefsky et al., 2018) | | ICC = .88 - .91; Cronbach α = .94 - .99 |  |  | (Mills et al., 2022) | 1 (0.3%) |
|  | **Non Specific/ Mixed Emotion Eliciting Tasks** | | | | | | | | | |
|  | Puzzle Task; Tangram Construction Task; Regulation Puzzle Box Task) | | 4 - 5 min | Dysregulation Coding System (Hoffman et al., 2006) | | ICC = .39 - .92; Pearson r = .93 - .98 | Convergent validity: Weak corr. with Emotion Control Scale (*r* = −.20, p = .079); medium corr. with DESR (*r* = −.28, *p* = .016); Discriminant validity: medium, corr. with total IQ (*r* = .30, *p* = .011) weak corr. with verbal comprehension (*r* = .27, *p* = .026), and Perceptual Reasoning (*r* = .28, *p* = .018); Validity supported by prior studies of preschool- and school-aged children with and without neurodevelopmental disorders (Baker et al., 2007; Hoffman et al., 2006; Norona & Baker, 2014) | Evaluation objectivity through blinded coders | (Baker et al., 2019; Eisenberg et al., 2000; Eisenberg & Morris, 2003; Fenning et al., 2018; Hagstrøm et al., 2019; Hagstrøm et al., 2021; Lee et al., 2018; Walcott & Landau, 2004)^70,71,88,360,412,421,430,434^ | 8 (2.5%) |
|  |  | |  |  | |  |  |  |  |  |
|  | Children’s Play Therapy Instrument (CPTI) | |  |  | | ICC = .75 - .97; Cronbach α = .71 - .75 |  | Implementation objectivity through blinded observations procedures | (Halfon et al., 2017; Halfon & Bulut, 2019; Halfon et al., 2019)^43,381,382^ | 3 (0.9%) |
|  |  | |  |  | |  |  |  |  |  |
|  | Problem Solving Task | | 3 min | Dysregulation Coding System (Hoffman et al., 2006); Emotion Dysregulation subscale (Cole et al., 1994) | | ICC = .79 - .90 |  | Evaluation objectivity through blinded coders | (Lee et al., 2018; Noroña et al., 2018)^90,434^ | 2 (0.6%) |
|  |  | |  |  | |  |  |  |  |  |
|  |  | |  |  | |  |  |  |  |  |
|  | Free Play | |  |  | | ICC = .88 |  | Evaluation objectivity through blinded coders | (Lee et al., 2018)^434^ | 1 (0.3%) |
|  |  | |  |  | |  |  |  |  |  |
|  | Banner Task | |  |  | | ICC = .88 |  | Evaluation objectivity through blinded coders | (Lee et al., 2018) | 1 (0.3%) |
|  |  | |  |  | |  |  |  |  |  |
|  | Collaborative Task parents and child | | 4 min |  | | ICC = .94 |  |  | (Jin et al., 2017)^427^ | 1 (0.3%) |
|  |  | |  |  | |  |  |  |  |  |
|  | Class Room Observations | |  |  | | Cohen's κ = .76 |  |  | (Kurki et al., 2015)^423^ | 1 (0.3%) |
|  |  | |  |  | |  |  |  |  |  |
|  | Tangram Construction Task | | 5 min | Tangram Emotion Coding Manual (TEC-M(Hagstrøm et al., 2019) | | ICC = .79 |  | Evaluation objectivity through blinded coders | (Spang et al., 2022)^428^ | 1 (0.3%) |
|  |  | |  |  | |  |  |  |  |  |
|  | Parent-Child Interaction | | 8 min |  | |  |  | Evaluation objectivity through blinded coders | (Cimino & Cerniglia, 2018) | 1 (0.3%) |
|  |  | |  |  | |  |  |  |  |  |
|  | Film Clips | | 3 min | Child Behavior Coding Scheme | | Cronbach α = .99 |  | Evaluation objectivity through blinded coders | (Dadds et al., 2016)^378^ | 1 (0.3%) |
|  | Regulating Taste Experience | |  |  | |  |  |  | (Hoffmann et al., 2015) | 1 (0.3%) |
|  |  | |  |  | |  |  |  |  |  |
|  | Child-Peer and -Counselors Interaction | | 10 min |  | | Agreement: 76 - 96% |  | Evaluation objectivity through blinded coders | (Shields et al., 1994)^435^ | 1 (0.3%) |
|  |  | |  |  | |  |  |  |  |  |
|  | Emotion Task: Discussing time when child was emotional | | 15 min |  | |  |  | Evaluation objectivity through blinded coders | (Suveg et al., 2008)^436^ | 1 (0.3%) |
|  |  | |  |  | |  |  |  |  |  |
|  | Social Group Session Activities | |  |  | | ICC = .89 |  |  | (Taylor et al., 2020)^437^ | 1 (0.3%) |
|  |  | |  |  | |  |  |  |  |  |
|  | Phonological Working Memory Task | |  |  | | Cohen's κ = .85 - 1.0 |  |  | (Tarle et al., 2021)^438^ | 1 (0.3%) |
|  |  | |  |  | |  |  |  |  |  |
|  | Picture Reappraisal Task (PRT) | |  | Reappraisal Tactic Coding Guide (McRae et al., 2012) | |  |  |  | (Hagstrøm et al., 2020) | 1 (0.3%) |
| **102 to 114 months** | **Frustration/Anger/Disappointment Eliciting Tasks** | | | | | | | | | |
|  | Attractive Toy in a Transparent Box (e.g. Lab-TAB) | |  | Dysregulation Coding System (Hoffman et al., 2006) | | ICC = .90 |  | Evaluation objectivity through blinded coders | (Baker et al., 2019; Fenning et al., 2018)^88,360^ | 2 (0.6%) |
|  |  | |  |  | |  |  |  |  |  |
|  | Disappointing Paradigm | |  |  | | Cohen's κ = .62 - .83 |  |  | (Forbes, Fox et al., 2006; Forbes, Shaw et al., 2006) | 2 (0.6%) |
|  |  | |  |  | |  |  |  |  |  |
|  | Delay of Gratification (cookie task) | | 3 - 10 min |  | | Cohen's κ = .64 - .79 | Predictive validity: Focus on cookie and less active distraction associated with higher rates of externalizing problems at age 6 |  | (Myruski & Dennis-Tiwary, 2021; Trentacosta & Shaw, 2009)^391,432^ | 2 (0.6%) |
|  |  | |  |  | |  |  |  |  |  |
|  | Virtually Impossible Dexterity Task | | 2 - 4 min |  | | Krippendorf’s α = .71 - .99 | Structural equation models testing concurrent and predictive validity showed good to adequate model fit, with .912< CFI < .99 and .035 < RMSEA < .073 |  | (Kirsch et al., 2019; Kirsch et al., 2015)^87,425^ | 2 (0.6%) |
|  |  | |  |  | |  |  |  |  |  |
|  | Backward Math Task | | 1 min |  | | Cohen’s kappa > .70; ICC > .80 |  | Implementation objectivity through standardized observations procedures | (Lo et al., 2021)^440^ | 1 (0.3%) |
|  |  | |  |  | |  |  |  |  |  |
|  | Balloons Game | | 2 - 6 min | Facial Action Coding System (FACS; (Ekman, 1994) | |  |  | Evaluation objectivity through blinded coders | (Roy et al., 2013) | 1 (0.3%) |
|  |  | |  |  | |  |  |  |  |  |
|  | Challenge Task | | 3 min | Dysregulation Coding System (Baker et al., 2007) | | ICC = .91 |  | Evaluation objectivity through blinded coders | (Baker et al., 2022)^426^ | 1 (0.3%) |
|  |  | |  |  | |  |  |  |  |  |
|  | Age-appropriate Lego model with missing pieces | | as much time as they needed |  | | Cronbach α = .79 - .93; Pearson r = .80 |  | Evaluation objectivity through blinded coders | (Melnick & Hinshaw, 2000)^419^ | 1 (0.3%) |
|  |  | |  |  | |  |  |  |  |  |
|  | Toy Behind Barrier task | |  |  | | Cohen's κ = .83 - .87 |  |  | ^337^(Costa et al., 2019) | 1 (0.3%) |
|  |  | |  |  | |  |  |  |  |  |
|  | Conflict Discourse Task | | 3 min |  | | ICC = .66 |  |  | (Hurrell et al., 2017)^429^ | 1 (0.3%) |
|  |  | |  |  | |  |  |  |  |  |
|  | Computerized Mirror Tracing Persistence Task (MTPT) | | 6 - 13 min | Emotion Dysregulation Inventory-Short Form (EDI; (Mazefsky et al., 2018) | | ICC = .88 - .91; Cronbach α = .94 - .99 |  |  | (Mills et al., 2022) | 1 (0.3%) |
|  |  | |  |  | |  |  |  |  |  |
|  | Impossible Building Tower Task | | 2 - 4 min | Software Eudico Linguistic Annotator (ELAN;(Wittenburg et al., 2006)) | | Krippendorf’s α = .71 - .79 | The measurement models of the validation constructs all showed a very good fit with the data (all RMSEAs < 0.05, SRMRs < 0.02, CFIs > 0.99 | Evaluation objectivity through blinded coders | (Rohlf & Krahé, 2015) | 1 (0.3%) |
|  | **Non Specific/ Mixed Emotion Eliciting Tasks** | | | | | | | | | |
|  | Puzzle Task; Tangram Construction Task; Regulation Puzzle Box Task) | | 5 min | Dysregulation Coding System (Hoffman et al., 2006) | | ICC = .39 - .92 ; Pearson r = .93 | Convergent validity: Weak corr. with Emotion Control Scale (*r* = −.20, p = .079); medium corr. with DESR (*r* = −.28, *p* = .016); Discriminant validity: medium, corr. with total IQ (*r* = .30, *p* = .011) weak corr. with verbal comprehension (*r* = .27, *p* = .026), and Perceptual Reasoning (*r* = .28, *p* = .018); Validity supported by prior studies of preschool- and school-aged children with and without neurodevelopmental disorders (Baker et al., 2007; Hoffman et al., 2006; Norona & Baker, 2014) | Evaluation objectivity through blinded coders | (Baker et al., 2019; Eisenberg et al., 2000; Fenning et al., 2018; Hagstrøm et al., 2019; Hagstrøm et al., 2021; Lee et al., 2018; Walcott & Landau, 2004)^70,71,88,360,421,430,434^ | 7 (2.2%) |
|  |  | |  |  | |  |  |  |  |  |
|  | Children’s Play Therapy Instrument (CPTI) | |  |  | | ICC = .75 - .97; Cronbach α = .71 - .75 |  | Evaluation objectivity through blinded coders | (Halfon et al., 2017; Halfon & Bulut, 2019; Halfon et al., 2019)^43,381,382,382^ | 3 (0.9%) |
|  |  | |  |  | |  |  |  |  |  |
|  | Free Play | |  |  | | ICC = .88 |  | Evaluation objectivity through blinded coders | (Lee et al., 2018)^434^ | 1 (0.3%) |
|  |  | |  |  | |  |  |  |  |  |
|  | Banner Task | |  |  | | ICC = .88 |  | Evaluation objectivity through blinded coders | (Lee et al., 2018)^434^ | 1 (0.3%) |
|  | Collaborative Task (parent and child) | | 4 min |  | | ICC = .94 |  |  | (Jin et al., 2017) | 1 (0.3%) |
|  |  | |  |  | |  |  |  |  |  |
|  | Class Room Observations | |  |  | | Cohen's κ = .76 |  |  | (Kurki et al., 2015)^423^ | 1 (0.3%) |
|  |  | |  |  | |  |  |  |  |  |
|  | Problem Solving Task | |  |  | | ICC = .88 |  | Evaluation objectivity through blinded coders | (Lee et al., 2018)^434^ | 1 (0.3%) |
|  |  | |  |  | |  |  |  |  |  |
|  | Film Clips | | 3 min | Child Behavior Coding Scheme | | Cronbach α = .99 |  | Evaluation objectivity through blinded coders | (Dadds et al., 2016)^378^ | 1 (0.3%) |
|  |  | |  |  | |  |  |  |  |  |
|  | Regulating Taste Experience | |  |  | |  |  |  | (Hoffmann et al., 2015)^431^ | 1 (0.3%) |
|  |  | |  |  | |  |  |  |  |  |
|  | Child-Peer and -Counselors Interaction | | 10 min |  | | Agreement: 76 - 96% |  | Evaluation objectivity through blinded coders | (Shields et al., 1994) | 1 (0.3%) |
|  |  | |  |  | |  |  |  |  |  |
|  | Emotion Task: Discussing time when child was emotional | | 15 min |  | |  |  | Evaluation objectivity through blinded coders | (Suveg et al., 2008)^436^ | 1 (0.3%) |
|  |  | |  |  | |  |  |  |  |  |
|  | Phonological Working Memory Task | |  |  | | Cohen's κ = .85 - 1.0 |  |  | (Tarle et al., 2021)^438^ | 1 (0.3%) |
|  |  | |  |  | |  |  |  |  |  |
|  | Social Group Session Activities | |  |  | | ICC = .89 |  |  | (Taylor et al., 2020)^437^ | 1 (0.3%) |
|  |  | |  |  | |  |  |  |  |  |
|  | Viewing images under ER conditions | | 25 - 30 min |  | |  |  |  | (Bettis et al., 2019)^441^ | 1 (0.3%) |
|  |  | |  |  | |  |  |  |  |  |
|  | Picture Distraction and Reappraisal Task Developmental Affective Photo System (DAPS) | |  | Self-Assessment Manikin (Bradley & Lang, 1994) | |  |  |  | (Kiani et al., 2024) | 1 (0.3%) |
|  |  | |  |  | |  |  |  |  |  |
|  | Different Games, child was given responsibility during the absence of the adult | | 10 min |  | |  |  |  | (Alarcón, 2010)^443^ | 1 (0.3%) |
|  |  | |  |  | |  |  |  |  |  |
|  | Picture Reappraisal Task (PRT) | |  | Reappraisal Tactic Coding Guide (McRae et al., 2012) | |  |  |  | (Hagstrøm et al., 2020)^439^ | 1 (0.3%) |
| **115 to 127 months** | **Frustration/Anger/Disappointment Eliciting Tasks** | | | | | | | | | |
|  | Attractive Toy in a Transparent Box (e.g. Lab-TAB) | |  | Dysregulation Coding System (Hoffman et al., 2006) | | ICC = .90 |  | Evaluation objectivity through blinded coders | (Baker et al., 2019; Fenning et al., 2018)^88,360^ | 2 (0.6%) |
|  |  | |  |  | |  |  |  |  |  |
|  | Virtually Impossible Dexterity Task | | 2 - 4 min |  | | Krippendorf’s α = .71 - .99 | Structural equation models testing concurrent and predictive validity showed good to adequate model fit, with .912< CFI < .99 and .035 < RMSEA < .073 |  | (Kirsch et al., 2019; Kirsch et al., 2015)^87,425^ | 2 (0.6%) |
|  |  | |  |  | |  |  |  |  |  |
|  | Delay of Gratification (cookie task) | | 3 min |  | | Cohen's κ = .64 - .79 | Predictive validity: Focus on cookie and less active distraction associated with higher rates of externalizing problems at age 6 |  | (Trentacosta & Shaw, 2009) | 1 (0.3%) |
|  |  | |  |  | |  |  |  |  |  |
|  | Balloons Game | | 2 - 6 min | Facial Action Coding System (FACS; (Ekman, 1994) | |  |  | Evaluation objectivity through blinded coders | (Roy et al., 2013)^409^ | 1 (0.3%) |
|  |  | |  |  | |  |  |  |  |  |
|  | Challenge Task | | 3 min | Dysregulation Coding System (Baker et al., 2007) | | ICC = .91 |  | Evaluation objectivity through blinded coders | (Baker et al., 2022)^426^ | 1 (0.3%) |
|  |  | |  |  | |  |  |  |  |  |
|  | Age-appropriate Lego model with pieces missing | | as much time as they needed |  | | Cronbach α = .79 - .93; Pearson r = .80 |  | Evaluation objectivity through blinded coders | (Melnick & Hinshaw, 2000)^419^ | 1 (0.3%) |
|  |  | |  |  | |  |  |  |  |  |
|  | Toy Behind Barrier task | |  |  | | Cohen's κ = .83 - .87 |  |  | (Costa et al., 2019)^337^ | 1 (0.3%) |
|  |  | |  |  | |  |  |  |  |  |
|  | Conflict Discourse Task | | 3 min |  | | ICC = .66 |  |  | (Hurrell et al., 2017) | 1 (0.3%) |
|  |  | |  |  | |  |  |  |  |  |
|  | Computerized Mirror Tracing Persistence Task (MTPT) | | 6 - 13 min | Emotion Dysregulation Inventory-Short Form (EDI; (Mazefsky et al., 2018) | | ICC = .88 - .91; Cronbach α = .94 - .99 |  |  | (Mills et al., 2022)^433^ | 1 (0.3%) |
|  |  | |  |  | |  |  |  |  |  |
|  | Impossible Building Tower Task | | 2 - 4 min | Software Eudico Linguistic Annotator (ELAN;(Wittenburg et al., 2006) | | Krippendorf’s α = .71 - .79 | The measurement models of the validation constructs all showed a very good fit with the data (all RMSEAs < 0.05, SRMRs < 0.02, CFIs > 0.99 | Evaluation objectivity through blinded coders | (Rohlf & Krahé, 2015) | 1 (0.3%) |
|  | **Non Specific/ Mixed Emotion Eliciting Tasks** | | | | | | | | | |
|  | Puzzle Task; Tangram Construction Task; Puzzle Box Task | | 5 min | Dysregulation Coding System (Hoffman et al., 2006) | | Cohen's κ = .81 - .88; ICC = .39 - .92 | Convergent validity: Weak corr. with Emotion Control Scale (*r* = −.20, p = .079); medium corr. with DESR (*r* = −.28, *p* = .016); Discriminant validity: medium, corr. with total IQ (*r* = .30, *p* = .011) weak corr. with verbal comprehension (*r* = .27, *p* = .026), and Perceptual Reasoning (*r* = .28, *p* = .018); Validity supported by prior studies of preschool- and school-aged children with and without neurodevelopmental disorders (Baker et al., 2007; Hoffman et al., 2006; Norona & Baker, 2014) | Evaluation objectivity through blinded coders | (Baker et al., 2019; Fenning et al., 2018; Hagstrøm et al., 2019; Hagstrøm et al., 2021; Lee et al., 2018; Perry et al., 2020; Walcott & Landau, 2004)^70,71,88,110,360,421,434^ | 7 (2.2%) |
|  |  | |  |  | |  |  |  |  |  |
|  | Children’s Play Therapy Instrument (CPTI) | |  |  | | ICC = .75 - .97; Cronbach α = .71 - .75 |  | Implementation objectivity through blinded observations procedures | (Halfon et al., 2017; Halfon & Bulut, 2019; Halfon et al., 2019)^43,381,382^ | 3 (0.9%) |
|  |  | |  |  | |  |  |  |  |  |
|  | Free Play | |  |  | | ICC = .88 |  | Evaluation objectivity through blinded coders | (Lee et al., 2018)^434^ | 1 (0.3%) |
|  |  | |  |  | |  |  |  |  |  |
|  | Banner Task | |  |  | | ICC = .88 |  | Evaluation objectivity through blinded coders | (Lee et al., 2018) | 1 (0.3%) |
|  |  | |  |  | |  |  |  |  |  |
|  | Collaborative Task (parent and child) | | 4 min |  | | ICC = .94 |  |  | (Jin et al., 2017)^427^ | 1 (0.3%) |
|  |  | |  |  | |  |  |  |  |  |
|  | Problem Solving Task | |  |  | | ICC = .88 |  | Evaluation objectivity through blinded coders | (Lee et al., 2018)^434^ | 1 (0.3%) |
|  |  | |  |  | |  |  |  |  |  |
|  | Film Clips | | 3 min | Child Behavior Coding Scheme | | Cronbach α = .99 |  | Evaluation objectivity through blinded coders | (Dadds et al., 2016)^378^ | 1 (0.3%) |
|  |  | |  |  | |  |  |  |  |  |
|  | Regulating Taste Experience | |  |  | |  |  |  | ^431^(Hoffmann et al., 2015) | 1 (0.3%) |
|  |  | |  |  | |  |  |  |  |  |
|  | Picture Reappraisal Task (PRT) | |  | Reappraisal Tactic Coding Guide (McRae et al., 2012) | |  |  |  | (Hagstrøm et al., 2020) | 1 (0.3%) |
|  |  | |  |  | |  |  |  |  |  |
|  | Child-Peer and -Counselors Interaction | | 10 min |  | | Agreement: 76 - 96% |  | Evaluation objectivity through blinded coders | (Shields et al., 1994)^435^ | 1 (0.3%) |
|  |  | |  |  | |  |  |  |  |  |
|  | Emotion Task: Discussing time when child was emotional | | 15 min |  | |  |  | Evaluation objectivity through blinded coders | (Suveg et al., 2008)^436^ | 1 (0.3%) |
|  |  | |  |  | |  |  |  |  |  |
|  | Phonological Working Memory Task | |  |  | | Cohen's κ = .85 - 1.0 |  |  | (Tarle et al., 2021)^438^ | 1 (0.3%) |
|  |  | |  |  | |  |  |  |  |  |
|  | Social Group Session Activities | |  |  | | ICC = .89 |  |  | (Taylor et al., 2020)^437^ | 1 (0.3%) |
|  |  | |  |  | |  |  |  |  |  |
|  | Viewing images under ER conditions | | 25 - 30 min |  | |  |  |  | (Bettis et al., 2019) | 1 (0.3%) |
|  |  | |  |  | |  |  |  |  |  |
|  | Survivor Computer Game | | 5 min |  | |  |  |  | (Reijntjes et al., 2006)^444^ | 1 (0.3%) |
|  |  | |  |  | |  |  |  |  |  |
|  | Class Room Observations | |  |  | | Cohen's κ = .71 - 1.0 |  | Evaluation objectivity through blinded coders | (Wilton et al., 2000)^445^ | 1 (0.3%) |
|  |  | |  |  | |  |  |  |  |  |
|  | Caregiver-Child Interaction Task | | 30 min |  | |  |  |  | (Sprecher et al., 2023)^446^ | 1 (0.3%) |
|  |  | |  |  | |  |  |  |  |  |
|  | Series of physical challenges | |  | Response to Challenge Scale (RCS) | | ICC = .91 - .93 |  | Evaluation objectivity through blinded coders | (Lakes, 2013)^447^ | 1 (0.3%) |
|  |  | |  |  | |  |  |  |  |  |
|  | Picture Distraction and Reappraisal Task; Developmental Affective Photo System (DAPS; Cordon et al., 2013) | |  | Self-Assessment Manikin (SAM; (Bradley & Lang, 1994) | |  |  |  | (Kiani et al., 2024) | 1 (0.3%) |
| **128 to 140 months** | **Frustration/Anger/Disappointment Eliciting Tasks** | | | | | | | | | |
|  | Attractive Toy in a Transparent Box (e.g. Lab-TAB) | |  | Dysregulation Coding System (Hoffman et al., 2006) | | ICC = .90 |  |  | (Baker et al., 2019)^360^ | 1 (0.3%) |
|  |  | |  |  | |  |  |  |  |  |
|  | Toy Behind Barrier task | |  |  | | Cohen's κ = .83 - .87 |  |  | ^337^(Costa et al., 2019) | 1 (0.3%) |
|  |  | |  |  | |  |  |  |  |  |
|  | Conflict Discourse Task | | 3 min |  | | ICC = .66 |  |  | (Hurrell et al., 2017)^429^ | 1 (0.3%) |
|  |  | |  |  | |  |  |  |  |  |
|  | Computerized Mirror Tracing Persistence Task (MTPT) | | 6 - 13 min | Emotion Dysregulation Inventory-Short Form (EDI; (Mazefsky et al., 2018) | | ICC = .88 - .91; Cronbach α = .94 - .99 |  |  | (Mills et al., 2022)^433^ | 1 (0.3%) |
|  |  | |  |  | |  |  |  |  |  |
|  | Age-appropriate Lego model with pieces missing | | as much time as they needed |  | | Cronbach α = .79 - .93; Pearson r = .80 |  | Evaluation objectivity through blinded coders | (Melnick & Hinshaw, 2000) | 1 (0.3%) |
|  | **Non Specific/ Mixed Emotion Eliciting Tasks** | | | | | | | | | |
|  | Puzzle Task (e.g.Tangram Construction Task) | | 5 min | Dysregulation Coding System (Hoffman et al., 2006) | | ICC = .39 - .92 | Convergent validity: Weak corr. with Emotion Control Scale (*r* = −.20, p = .079); medium corr. with DESR (*r* = −.28, *p* = .016); Discriminant validity: medium, corr. with total IQ (*r* = .30, *p* = .011) weak corr. with verbal comprehension (*r* = .27, *p* = .026), and Perceptual Reasoning (*r* = .28, *p* = .018); Validity supported by prior studies of preschool- and school-aged children with and without neurodevelopmental disorders (Baker et al., 2007; Hoffman et al., 2006; Norona & Baker, 2014) |  | (Baker et al., 2019; Hagstrøm et al., 2019; Hagstrøm et al., 2021; Walcott & Landau, 2004)^70,71,360,421^ | 4 (1.2%) |
|  |  | |  |  | |  |  |  |  |  |
|  | Collaborative Task (parent and child) | | 4 min |  | | ICC = .94 |  |  | (Jin et al., 2017)^427^ | 1 (0.3%) |
|  |  | |  |  | |  |  |  |  |  |
|  | Film Clips | | 3 min | Child Behavior Coding Scheme | | Cronbach α = .99 |  | Evaluation objectivity through blinded coders | (Dadds et al., 2016)^378^ | 1 (0.3%) |
|  |  | |  |  | |  |  |  |  |  |
|  | Picture Reappraisal Task (PRT) | |  | Reappraisal Tactic Coding Guide (McRae et al., 2012) | |  |  |  | ^439^(Hagstrøm et al., 2020) | 1 (0.3%) |
|  |  | |  |  | |  |  |  |  |  |
|  | Child-Peer and -Counselors Interaction | | 10 min |  | | Agreement: 76 - 96% |  | Evaluation objectivity through blinded coders | (Shields et al., 1994) | 1 (0.3%) |
|  |  | |  |  | |  |  |  |  |  |
|  | Phonological Working Memory Task | |  |  | | Cohen's κ = .85 - 1.0 |  |  | (Tarle et al., 2021)^438^ | 1 (0.3%) |
|  |  | |  |  | |  |  |  |  |  |
|  | Discussing time when child was emotional | | 15 min |  | |  |  | Evaluation objectivity through blinded coders | (Suveg et al., 2008)^436^ | 1 (0.3%) |
|  |  | |  |  | |  |  |  |  |  |
|  | Images with regulation task | | 25 - 30 min |  | |  |  |  | (Bettis et al., 2019)^441^ | 1 (0.3%) |
|  | Survivor Computer Game | | 5 min |  | |  |  |  | (Reijntjes et al., 2006)^444^ | 1 (0.3%) |
|  |  | |  |  | |  |  |  |  |  |
|  | Picture Distraction and Reappraisal Task | |  | Self-Assessment Manikin (SAM; (Bradley & Lang, 1994) | |  |  |  | (Kiani et al., 2024) | 1 (0.3%) |
|  |  | |  |  | |  |  |  |  |  |
|  | Regulating Taste Experience | |  |  | |  |  |  | (Hoffmann et al., 2015)^431^ | 1 (0.3%) |

**Table 1e**

*Behavioral observation methods of emotion regulation for teens (141 to 192 months)*

| **Age Range (months)** | **Task** | **Duration (sec/min/h)** | | **Coding Manual** | **Reliability** | **Validity** | **Objectivity** | **Studies using this task** | **Number/ Percentage of studies using task** |
| --- | --- | --- | --- | --- | --- | --- | --- | --- | --- |
| **Teen** | | | | | | | | | |
| **141 to 153 months** | **Frustration/Anger/Disappointment Eliciting Tasks** | | | | | | | | |
|  | Toy Behind Barrier task |  |  | | Cohen's κ = .83 - .87 |  |  | (Costa et al., 2019)^337^ | 1 (0.3%) |
|  |  |  |  | |  |  |  |  |  |
|  | Age-appropriate Lego model with missing pieces | as much time as they needed |  | | Cronbach α = .79 - .93;  Pearson r = .80 |  | Evaluation objectivity through blinded coders | (Melnick & Hinshaw, 2000)^419^ | 1 (0.3%) |
|  |  |  |  | |  |  |  |  |  |
|  | Conflict Discourse Task | 3 min |  | | ICC = .66 |  |  | (Hurrell et al., 2017)^429^ | 1 (0.3%) |
|  |  |  |  | |  |  |  |  |  |
|  | Computerized Mirror Tracing Persistence Task (MTPT) | 6 - 13 min | Emotion Dysregulation Inventory-Short Form (EDI; (Mazefsky et al., 2018) | | ICC = .88 - .91; Cronbach α = .94 - .99 |  |  | (Mills et al., 2022) | 1 (0.3%) |
|  |  |  |  | |  |  |  |  |  |
|  | Computer-based jump and run game |  |  | | Cohen's κ = .72 |  |  | (Zimmermann & Spangler, 2016)^448^ | 1 (0.3%) |
|  |  |  |  | |  |  |  |  |  |
|  | Dyadic Computer Game |  |  | |  |  |  | (Spangler & Zimmermann, 2014)^420^ | 1 (0.3%) |
|  | **Fear Eliciting Task** | | | | | | | | |
|  | Dyadic ‘‘talk show’’ situation | 10 min |  | |  |  |  | (Spangler & Zimmermann, 2014)^420^ | 1 (0.3%) |
|  | **Non Specific/ Mixed Emotion Eliciting Tasks** | | | | | | | | |
|  | Puzzle Task (e.g. Tangram Construction Task) | 5 min |  | | ICC = .60 - .83 | Convergent validity: Weak corr. with Emotion Control Scale (*r* = −.20, p = .079); medium corr. with DESR (*r* = −.28, *p* = .016); Discriminant validity: medium, corr. with total IQ (*r* = .30, *p* = .011) weak corr. with verbal comprehension (*r* = .27, *p* = .026), and Perceptual Reasoning (*r* = .28, *p* = .018); Validity supported by prior studies of preschool- and school-aged children with and without neurodevelopmental disorders (Baker et al., 2007; Hoffman et al., 2006; Norona & Baker, 2014) |  | (Hagstrøm et al., 2019; Hagstrøm et al., 2021) | 2 (0.3%) |
|  |  |  |  | |  |  |  |  |  |
|  | Collaborative Task (parent and child) | 4 min |  | | ICC = .94 |  |  | (Jin et al., 2017)^427^ | 1 (0.3%) |
|  |  |  |  | |  |  |  |  |  |
|  | Parent-Child Interaction | 8 min |  | |  |  | Evaluation objectivity through blinded coders | (Cimino & Cerniglia, 2018) | 1 (0.3%) |
|  |  |  |  | |  |  |  |  |  |
|  | Film Clips | 3 min | Child Behavior Coding Scheme | | Cronbach α = .99 |  | Evaluation objectivity through blinded coders | (Dadds et al., 2016) | 1 (0.3%) |
|  |  |  |  | |  |  |  |  |  |
|  | Regulating Taste Experience |  |  | |  |  |  | ^431^(Hoffmann et al., 2015) | 1 (0.3%) |
|  |  |  |  | |  |  |  |  |  |
|  | Picture Reappraisal Task (PRT) |  | Reappraisal Tactic Coding Guide (McRae et al., 2012) | |  |  |  | (Hagstrøm et al., 2020)^439^ | 1 (0.3%) |
|  |  |  |  | |  |  |  |  |  |
|  | Emotion Task: Discussing time when child was emotional | 15 min |  | |  |  | Evaluation objectivity through blinded coders | (Suveg et al., 2008)^436^ | 1 (0.3%) |
|  |  |  |  | |  |  |  |  |  |
|  | Phonological Working Memory Task |  |  | | Cohen's κ = .85 - 1.00 |  |  | (Tarle et al., 2021)^438^ | 1 (0.3%) |
|  |  |  |  | |  |  |  |  |  |
|  | Viewing images under ER conditions | 25 - 30 min |  | |  |  |  | (Bettis et al., 2019) | 1 (0.3%) |
|  |  |  |  | |  |  |  |  |  |
|  | Survivor Computer Game | 5 min |  | |  |  |  | (Reijntjes et al., 2006)^444^ | 1 (0.3%) |
|  |  |  |  | |  |  |  |  |  |
|  | Class Room Observation | 37 min |  | | Cohen's κ = .63 |  |  | (Geldhof et al., 2021)^449^ | 1 (0.3%) |
|  |  |  |  | |  |  |  |  |  |
|  | Picture Distraction and Reappraisal Task; Developmental Affective Photo System (DAPS) |  | Self-Assessment Manikin (SAM; (Bradley & Lang, 1994) | |  |  |  | ^442^(Kiani et al., 2024) | 1 (0.3%) |
|  |  |  |  | |  |  |  |  |  |
|  | Child-Peer and -Counselors Interaction | 10 min |  | | Agreement: 76 - 96% |  | Evaluation objectivity through blinded coders | (Shields et al., 1994)^435^ | 1 (0.3%) |
| **154 to 166 months** | **Frustration/Anger/Disappointment Eliciting Tasks** | | | | | | | | |
|  | Toy Behind Barrier task |  |  | | Cohen's κ = .83 - .87 |  |  | (Costa et al., 2019) | 1 (0.3%) |
|  | Conflict Discourse Task | 3 min |  | | ICC = .66 |  |  | (Hurrell et al., 2017)^429^ | 1 (0.3%) |
|  |  |  |  | |  |  |  |  |  |
|  | Computerized Mirror Tracing Persistence Task (MTPT) | 6 - 13 min | Emotion Dysregulation Inventory-Short Form (EDI; (Mazefsky et al., 2018) | | ICC = .88 - .91; Cronbach α = .94 - .99 |  |  | (Mills et al., 2022)^433^ | 1 (0.3%) |
|  | **Non Specific/ Mixed Emotion Eliciting Tasks** | | | | | | | | |
|  | Film Clips | 3 min | Child Behavior Coding Scheme ( | | Cronbach α = .99 |  | Evaluation objectivity through blinded coders | (Dadds et al., 2016)^378^ | 1 (0.3%) |
|  |  |  |  | |  |  |  |  |  |
|  | Emotion Task: Discussing time when the child was emotional | 15 min |  | |  |  | Evaluation objectivity through blinded coders | (Suveg et al., 2008)^436^ | 1 (0.3%) |
|  |  |  |  | |  |  |  |  |  |
|  | Viewing images under ER conditions | 25 - 30 min |  | |  |  |  | (Bettis et al., 2019) | 1 (0.3%) |
|  |  |  |  | |  |  |  |  |  |
|  | Picture Distraction and Reappraisal Task; Developmental Affective Photo System (DAPS) |  | Self-Assessment Manikin (SAM; (Bradley & Lang, 1994) | |  |  |  | (Kiani et al., 2024)^442^ | 1 (0.3%) |
|  |  |  |  | |  |  |  |  |  |
|  | Survivor Computer Game | 5 min |  | |  |  |  | (Reijntjes et al., 2006)^444^ | 1 (0.3%) |
| **167 to 179 months** | **Frustration/Anger/Disappointment Eliciting Tasks** | | | | | | | | |
|  | Conflict Discourse Task | 3 min |  | | ICC = .66 |  |  | (Hurrell et al., 2017)^429^ | 1 (0.3%) |
|  | **Non Specific/ Mixed Emotion Eliciting Tasks** | | | | | | | | |
|  | Film Clips | 3 min | Child Behavior Coding Scheme | | Cronbach α = .99 |  | Evaluation objectivity through blinded coders | (Dadds et al., 2016)^378^ | 1 (0.3%) |
|  |  |  |  | |  |  |  |  |  |
|  | Viewing images under ER conditions | 25 - 30 min |  | |  |  |  | (Bettis et al., 2019) | 1 (0.3%) |
|  |  |  |  | |  |  |  |  |  |
|  | Picture Distraction and Reappraisal Task; Developmental Affective Photo System (DAPS) |  | Self-Assessment Manikin (SAM; (Bradley & Lang, 1994) | |  |  |  | (Kiani et al., 2024)^442^ | 1 (0.3%) |
| **180 to 192 months** | **Frustration/Anger/Disappointment Eliciting Tasks** | | | | | | | | |
|  | Conflict Discourse Task | 3 min |  | | ICC = .66 |  |  | (Hurrell et al., 2017)^429^ | 1 (0.3%) |

**Table 2a**

*Physiological measurement of emotion regulation for neonates and infants (0 to 12 months old)*

| **Age range (months)** | **Measurement Method** | **Physiological Phenomenon (PP)** | **Task** | **Reliability** | **Validity** | **Objectivity** | **Studies measuring PP** | **Number/ Percentage of studies measuring PP** |
| --- | --- | --- | --- | --- | --- | --- | --- | --- |
| **Neonates** | | | | | | | | |
|  | ECG | Vagal Tone | Resting State |  |  |  | (Feldman, 2015) | 1 (0.5%) |
| **Infant** | | | | | | | | |
| **1 to 12 months** | **Frustration/Anger/Disappointment Eliciting Tasks** | | | | | | | |
|  | ECG | Vagal Tone | Still-Face (Fivaz-Depeursinge et al., 2010); Parent-Child Discussion; Arm-Restraint Procedure^208^ |  |  |  | (Abney et al., 2021; Calkins et al., 2002; Conradt & Ablow, 2010; Perry, Calkins & Bell, 2016; Rattaz et al., 2023; Stifter. et al., 1999; Suurland et al., 2017) | 7 (3.5%) |
|  |  |  |  |  |  |  |  |  |
|  | ECG | Heart Rate; Heart Rate Variability | Still Face Paradigm; Attractive Toy in a Transparent Box (Lab-TAB^183^); Arm-Restraint Task^450^ |  |  | Evaluation objectivity through blinded coders | (Conradt & Ablow, 2010; Kuiper et al., 2023; Porter et al., 2022; Suurland et al., 2017) | 4 (2%) |
|  |  |  |  |  |  |  |  |  |
|  | ECG | RSA | Still Face Paradigm (e.g. (Tronick et al., 1978) |  |  |  | (Gao et al., 2022; Ham & Tronick, 2009; Ostlund et al., 2017) | 3 (1.5%) |
|  |  |  |  |  |  |  |  |  |
|  | Accelerometer | Movements (e.g. head velocity and foot movement) | Still Face Paradigm |  |  | Evaluation objectivity through blinded coders | (Conradt & Ablow, 2010; Kuiper et al., 2023; Porter et al., 2022) | 3 (1.5%) |
|  |  |  |  |  |  |  |  |  |
|  | EEG | Asymmetry | Arm restraint task; Toy removal task^208,210,450^; Still Face Paradigm (DiCorcia et al., 2016) |  |  |  | (Perone et al., 2020; Swingler et al., 2014) | 2 (1%) |
|  |  |  |  |  |  |  |  |  |
|  | Salvia Sample | Cortisol | Arm restraint task; Barrier Task; Masks Task; Umbrella Task; Puppets Task (Lab-TAB; (Goldsmith, 1999) |  |  |  | (Habersaat et al., 2013; Wu & Feng, 2020) | 2 (1%) |
|  |  |  |  |  |  |  |  |  |
|  | "Wet" Electrodes | Skin Conductance | Still Face Paradigm | ICC = .94 |  |  | (Ham & Tronick, 2009) | 1 (0.5%) |
|  |  |  |  |  |  |  |  |  |
|  | ECG | Pre-Ejection Period (PEP) | Still Face Paradigm |  |  | Evaluation objectivity through blinded coders | (Suurland et al., 2017) | 1 (0.5%) |
|  |  |  |  |  |  |  |  |  |
|  | Functional near-infrared spectroscopy (fNIRS) | Brain Oxygenation | Still Face Paradigm |  |  |  | (Behrendt et al., 2020) | 1 (0.5%) |
|  | **Fear Eliciting Tasks** | | | | | | | |
|  | Salvia Sample | Cortisol | Masks Task; Umbrella Task (Lab-TAB; (Goldsmith, 1999) |  |  |  | (Frost et al., 2018; Habersaat et al., 2013; Wu & Feng, 2020) | 3 (1.5%) |
|  |  |  |  |  |  |  |  |  |
|  | ECG | Heart Rate Variability | Stranger Task (Lab-TAB^183^) |  |  |  | (Zeegers et al., 2018) | 1 (0.5%) |
|  | **Joy Eliciting Tasks** | | | | | | | |
|  | Salvia Sample | Cortisol | Puppets Task (Lab-TAB; (Goldsmith, 1999) |  |  |  | (Habersaat et al., 2013; Wu & Feng, 2020)^211,215^ | 2 (1%) |
|  | **Non Specific Emotion Eliciting Tasks or Mixed Emotions Tasks** | | | | | | | |
|  | ECG | Heart Rate; Heart Rate Variability ; Heart Period | Stimulus battery with dynamic and static viewing materials (Barbaro et al., 2016); Basline; Resting State; Attention Task |  |  | Evaluation objectivity through blinded coders | (Calkins et al., 2002; Krzeczkowski et al., 2021; Kuiper et al., 2023; Mortaji et al., 2023; Porter et al., 2022; Stifter. et al., 1999; Wass et al., 2018; Wu & Gazelle, 2021)^97,208,220,231,454,457–459^ | 8 (4%) |
|  |  |  |  |  |  |  |  |  |
|  | ECG | Vagal Tone | Parent-Child Interactions; Lausanne Trilogue Play (LTP; (Fivaz-Depeursinge et al., 2010); Diaper Change Play ((Rime et al., 2018); Resting State; Attention Task |  |  |  | (Calkins et al., 2002; Perry, Swingler et al., 2016; Puglisi et al., 2023; Rattaz et al., 2023; Stifter. et al., 1999)^208,231,345,460,461^ | 5 (2.5%) |
|  |  |  |  |  |  |  |  |  |
|  | EEG | Asymmetry | Baseline Tasks; Resting State |  |  |  | (Krzeczkowski et al., 2021; Perone et al., 2020)^458,462^ | 2 (1%) |
|  |  |  |  |  |  |  |  |  |
|  | ECG | RSA | Resting State |  |  |  | (Lin et al., 2021)^241^ | 1 (0.5%) |
|  |  |  |  |  |  |  |  |  |
|  | Salvia Sample | Cortisol | Daily Measuring Points |  |  |  | (Frost et al., 2018)^456^ | 1 (0.5%) |
|  |  |  |  |  |  |  |  |  |
|  | EDA | Electrodermal Activity | Stimulus battery with dynamic and static viewing materials (Barbaro et al., 2016) |  |  |  | (Wass et al., 2018)^457^ | 1 (0.5%) |
|  |  |  |  |  |  |  |  |  |
|  | Accelerometer | Movements (e.g. head velocity and foot movement) | Stimulus battery with dynamic and static viewing materials (Barbaro et al., 2016) |  |  |  | (Wass et al., 2018)^457^ | 1 (0.5%) |

**Table 2b**

*Physiological measurement of emotion regulation for toddlers (13 to 36 months old)*

| **Age range (months)** | **Measurement Method** | **Physiological Phenomenon (PP)** | **Task** | **Reliability** | **Validity** | **Objectivity** | **Studies measuring PP** | **Number/ Percentage of studies measuring PP** |  |
| --- | --- | --- | --- | --- | --- | --- | --- | --- | --- |
| **Toddler** | | | | | | | | | |
| **13 to 24 months** | **Frustration/Anger/Disappointment Eliciting Tasks** | | | | | | | | |
|  | ECG | RSA | Snack Delay Task; Tasks with different stress levels; Delay of Gratification; Not Sharing Task (Lab-TAB; (Goldsmith et al., 2010); Attractive Toy Behind the Barrier Task (Lab-TAB^183^;Disappointment Task (e.g., (Cole, 1986) | ICC = .95 - .98 |  | Evaluation and implementation objectivity through blinded coders and assessors | (Bocknek et al., 2020; Bocknek et al., 2018; Calkins & Keane, 2004; Cho et al., 2017; Dollar et al., 2020; Hassan et al., 2023; Kuiper et al., 2023; Richardson et al., 2019; Scrimgeour et al., 2016) | 9 (4.5%) |  |
|  |  |  |  |  |  |  |  |  |  |
|  | ECG | Heart Rate; Heart Period | Arm-Restraint Task^450^; Delay of Gratification; Attractive Toy in a Transparent Box (Lab-TAB^183^) |  |  | Evaluation objectivity through blinded coders | (Calkins & Johnson, 1998; Kuiper et al., 2023; Porter et al., 2022)^95,238,436^ | 3 (1.5%) |  |
|  |  |  |  |  |  |  |  |  |  |
|  | Salvia Sample | Cortisol | Arm restraint task; Barrier Task (Lab-TAB; (Goldsmith, 1999)); Delay of Gratification (Braungart-Rieker. & Stifter, 1996) |  |  |  | (Frost et al., 2018; Wu & Feng, 2020)^211,456^ | 2 (1%) |  |
|  |  |  |  |  |  |  |  |  |  |
|  | ECG | Vagal Tone | Delay of Gratification |  |  |  | (Calkins & Johnson, 1998)^258^ | 1 (0.5%) |  |
|  | **Fear Eliciting Task** | | | | | | | | |
|  | Salvia Sample | Cortisol | Masks Task (Lab-TAB; (Goldsmith, 1999) |  |  |  | (Wu & Feng, 2020)^211^ | 1 (0.5%) |  |
|  |  |  |  |  |  |  |  |  |  |
|  | EEG | ERP; Asymmetry | Mother Separation |  |  |  | (Dawson et al., 1992)^463^ | 1 (0.5%) |  |
|  |  |  |  |  |  |  |  |  |  |
|  | ECG | RSA | Clown Episode; Stranger Approach; Jumping Spider |  |  |  | (Cho et al., 2017)^464^ | 1 (0.5%) |  |
|  | **Empathy Eliciting Tasks** | | | | | | | | |
|  | ECG | RSA | Empathy Situation |  |  |  | (Calkins & Keane, 2004)^465^ | 1 (0.5%) |  |
|  | **Joy Eliciting Tasks** | | | | | | | | |
|  | ECG | Vagal Tone | Positive Emotion Puppet Play Episode; Attractive Musical Toy |  |  |  | (Calkins & Johnson, 1998)^258^ | 1 (0.5%) |  |
|  |  |  |  |  |  |  |  |  |  |
|  | ECG | Heart Period | Positive Emotion Puppet Play Episode; Attractive Musical Toy |  |  |  | (Calkins & Johnson, 1998)^258^ | 1 (0.5%) |  |
|  |  |  |  |  |  |  |  |  |  |
|  | ECG | RSA | Interactive Puppet Show |  |  |  | ^464^(Cho et al., 2017) | 1 (0.5%) |  |
|  | **Non Specific Emotion Eliciting Tasks or Mixed Emotions Tasks** | | | | | | | | |
|  | ECG | RSA | Resting State; Neutral Video; Enjoyable Family Book Sharing Task |  |  |  | (Grady & Callan, 2019; Hassan et al., 2023; Hastings et al., 2008; Lin et al., 2021; Perry, Calkins et al., 2018; Richardson et al., 2019; Scrimgeour et al., 2016; Smith. et al., 2019)^143,144,241,299,406,466,467^ | 7 (3.5%) |  |
|  |  |  |  |  |  |  |  |  |  |
|  | ECG | Heart Rate; Heart Rate Variability; Heart Period | Resting State; Baseline; Neutral Video |  |  | Evaluation objectivity through blinded coders | (Calkins & Johnson, 1998; Kuiper et al., 2023; Porter et al., 2022; Stifter. et al., 1999; Wu & Gazelle, 2021)^97,220,231,258,454^ | 5 (2.5%) |  |
|  |  |  |  |  |  |  |  |  |  |
|  | ECG | Vagal Tone | Resting State; Neutral Video |  |  |  | (Kennedy et al., 2004; Skibo et al., 2020; Stifter. et al., 1999)^231,468,469^ | 3 (1.5%) |  |
|  |  |  |  |  |  |  |  |  |  |
|  | Salvia Sample | Cortisol | Daily Measuring Points |  |  |  | (Frost et al., 2018)^456^ | 1 (0.5%) |  |
|  |  |  |  |  |  |  |  |  |  |
|  | EEG | ERP; Asymmetry | Resting State |  |  |  | ^463^(Dawson et al., 1992) | 1 (0.5%) |  |
| **25 to 36 months** | **Frustration/Anger/Disappointment Eliciting Tasks** | | | | | | | | |
|  | ECG | RSA | Tasks with different stress levels; Not Sharing Task (Lab-TAB; (Goldsmith et al., 2010); Attractive Toy in a Transparent Box (Lab-TAB^183^); Problem Solving Tasks; Delay of Gratification^254^ (Calkins, 1997); Emotional Video Clip (e.g. (Leupoldt et al., 2007); Still Face Paradigm; Disappointment Task (Saarni, 1984)^232^; Impossibly Perfect Circles task (IPC; LAB-TAB; (Goldsmith, 1993);Puzzle Task | ICC = .95 - .98 | Support for convergent validity: corr. RSA with behavioral observation | Implement objectivity through blinded assessors | ^126,265,266,280,325,451,452,455,459–464^(Bocknek et al., 2020; Bocknek et al., 2018; Calkins & Keane, 2004; Calkins & Dedmon, 2000; Forbes, Fox et al., 2006; Gatzke-Kopp et al., 2015; Hassan et al., 2023; Hastings et al., 2008; Nelson et al., 2012; Perry et al., 2012; Pruett et al., 2023; Richardson et al., 2019; Shih et al., 2018; Smith. et al., 2019) | 14 (7%) |  |
|  |  |  |  |  |  |  |  |  |  |
|  | EEG | Asymmetry; Alpha Power | Disappointment Task (Cole et al., 1994) |  |  |  | (Forbes, Fox et al., 2006) | 1 (0.5%) |  |
|  |  |  |  |  |  |  |  |  |  |
|  | ECG | Heart Rate | Attractive Toy in a Transparent Box (Lab-TAB; (Goldsmith & Rothbart, 1996) |  |  | Evaluation objectivity through blinded coders | (Kuiper et al., 2023) | 1 (0.5%) |  |
|  |  |  |  |  |  |  |  |  |  |
|  | Functional near-infrared spectroscopy (fNIRS) | Brain Oxygenation | Frustration Emotion Task for Children (FETCH; (Perlman et al., 2015) |  |  |  | ^475^(Grabell et al., 2018) | 1 (0.5%) |  |
|  | **Fear Eliciting Tasks** | | | | | | | |  |
|  | Salvia Samples | Cortisol | Stranger Approach Situation (LAB-TAB, (Goldsmith, 1999) |  |  |  | (Zimmermann & Stansbury, 2004)^230^ | 1 (0.5%) |  |
|  | **Empathy Eliciting Tasks** | | | | | | | |  |
|  | ECG | RSA | Empathy Situation |  |  |  | (Calkins & Keane, 2004; Calkins & Dedmon, 2000)^344,465^ | 2 (1%) |  |
|  | **Joy Eliciting Tasks** | | | | | | | |  |
|  | ECG | RSA | Peek-a-Boo (LAB-TAB, (Goldsmith, 1993) |  |  |  | (Calkins & Keane, 2004; Calkins & Dedmon, 2000)^344,465^ | 2 (1%) |  |
|  | **Non Specific Emotion Eliciting Tasks or Mixed Emotions Tasks** | | | | | | | |  |
|  | ECG | RSA | Attention task; Enjoyable Family Book Sharing Task.; Resting State; Free Play; Joint Interaction Episodes; Memory Recall Task | ICC = .95 - .98;  Pearson r = .99 |  | Implement objectivity through blinded assessors | (Calkins & Keane, 2004; Clark et al., 2016; Gray et al., 2018; Hastings et al., 2008; Nelson et al., 2012; Perry et al., 2012; Pruett et al., 2023; Richardson et al., 2019; Smith. et al., 2019)^75,143,146,465,466,471–473,476^ | 9 (4.5%) |  |
|  |  |  |  |  |  |  |  |  |  |
|  | ECG | Vagal Tone | Resting State |  |  |  | (Kennedy et al., 2004; Skibo et al., 2020; Stifter. et al., 1999)^231,468,469^ | 3 (1.5%) |  |
|  |  |  |  |  |  |  |  |  |  |
|  | ECG | Heart Rate; Heart Rate Variability | Resting State |  |  | Evaluation objectivity through blinded coders | (Kuiper et al., 2023; Stifter. et al., 1999; Wu & Gazelle, 2021)^97,220,231^ | 3 (1.5%) |  |
|  |  |  |  |  |  |  |  |  |  |
|  | EEG | ERP; Asymmetry | Story Preview – Background Conversation – Child Narrative Sequence | For frequency band, background conversation and ROI: Cronbach α = .91 - .99 |  |  | (Arnold et al., 2011; Forbes, Shaw et al., 2006)^129,340^ | 2 (1%) |  |
|  |  |  |  |  |  |  |  |  |  |
|  | Salvia Samples | Cortisol | Daily Measuring Points |  |  |  | ^456^(Frost et al., 2018) | 1 (0.5%) |  |
|  |  |  |  |  |  |  |  |  |  |
|  | fMRI | ROI | Resting State |  |  |  | (Ewell et al., 2023) | 1 (0.5%) |  |
|  |  |  |  |  |  |  |  |  |  |
|  | ECG | RSA | Emotional audio/video clip | ICC = .99 |  |  | (Jones et al., 2017) | 1 (0.5%) |  |
|  |  |  |  |  |  |  |  |  |  |
|  | ECG | R Waves | Memory Recall Task |  |  |  | ^476^(Gray et al., 2018) | 1 (0.5%) |  |

**Table 2c**

*Physiological measurement of emotion regulation for preschoolers (13 to 62 months)*

| **Age range (months)** | **Measurement Method** | **Physiological Phenomenon (PP)** | **Task** | **Reliability** | **Validity** | **Objectivity** | **Studies measuring PP** | **Number/ Percentage of studies measuring PP** |  |
| --- | --- | --- | --- | --- | --- | --- | --- | --- | --- |
| **Preschool** | | | | | | | | | |
| **37 to 49 months** | **Frustration/Anger/Disappointment Eliciting Tasks** | | | | | | | | |
|  | ECG | RSA | Delay of Gratification; Impossible Perfect Circle; Not Sharing Task (Lab-TAB; (Goldsmith et al., 2010); Disappointment Task (e.g. (Cole et al., 1994; Saarni, 1984); Attractive Toy in a Transparent Box (Lab-TAB^183^); Puzzle Task |  | Support for convergent validity: corr. RSA with behavioral observation |  | (Calkins & Keane, 2004; Distefano et al., 2021; Dollar et al., 2020; Forbes, Fox et al., 2006; Hassan et al., 2023; Kahle et al., 2018; Kahle et al., 2021; Nelson et al., 2012; Perry et al., 2012; Pruett et al., 2023; Shih et al., 2018) | 11 (5.5%) |  |
|  |  |  |  |  |  |  |  |  |  |
|  | Functional near-infrared spectroscopy (fNIRS) | Brain Oxygenation | Frustration Emotion Task for Children (FETCH^477^; (Perlman et al., 2015); Incredible Cake Kids (ICK^478^) |  |  |  | (Grabell et al., 2019; Grabell et al., 2018; Santana & Grabell, 2023)^465,469,470^ | 3 (1.5%) |  |
|  |  |  |  |  |  |  |  |  |  |
|  | EEG | ERP: Asymmetrie; Alpha Power | Adapted Affective Posner task (Perez-Edgar & Fox, 2005); Disappointment Task^232^ |  |  |  | (Forbes, Fox et al., 2006; Lugo-Candelas et al., 2017)^126,471^ | 2 (1%) |  |
|  |  |  |  |  |  |  |  |  |  |
|  | ECG | Heart Rate Variability | Attractive Toy in a Transparent Box (Lab-TAB^183^) |  |  | Evaluation objectivity through blinded coders | (Berry et al., 2019; Kuiper et al., 2023)^95,342^ | 2 (1%) |  |
|  |  |  |  |  |  |  |  |  |  |
|  | ECG | PEP | Impossible Perfect Circle; Not Sharing Task (Lab-TAB; (Goldsmith et al., 2010) |  | Support for convergent validity: corr. PEP with behavioral observation |  | (Kahle et al., 2016)^479^ | 1 (0.5%) |  |
|  |  |  |  |  |  |  |  |  |  |
|  | ECG | Vagal Tone | Delay of Gratification (Martin, 1981) |  |  |  | (Santucci et al., 2008)^356^ | 1 (0.5%) |  |
|  |  |  |  |  |  |  |  |  |  |
|  | Salvia Samples | Cortisol | Disappointing Task (Cole, 1986; Saarni, 1984) |  |  |  | (Grabell et al., 2015)^480^ | 1 (0.5%) |  |
|  | **Fear Eliciting Tasks** | | | | | | | |  |
|  | Salvia Samples | Cortisol | Stranger Approach Situation (LAB-TAB); Masks Task (Lab-TAB) |  |  |  | (Abraham et al., 2018; Zimmermann & Stansbury, 2004)^47,230^ | 2 (1%) |  |
|  |  |  |  |  |  |  |  |  |  |
|  | ECG | RSA | Jumping Lizard (Lab-TAB) |  |  |  | (Tsotsi et al., 2023)^236^ | 1 (0.5%) |  |
|  | **Empathy Eliciting Tasks** | | | | | | | |  |
|  | ECG | RSA | Empathy Situation |  |  |  | (Calkins & Keane, 2004)^465^ | 1 (0.5%) |  |
|  | **Non Specific Emotion Eliciting Tasks or Mixed Emotions Tasks** | | | | | | | |  |
|  | ECG | RSA | Attention Task; Resting State; Joint Interaction Episodes; International Affective Picture System (IAPS; (Lang, 2005); Emotional audio/video clip; Memory Recall Task; Resting State | ICC = .99 |  |  | (Calkins & Keane, 2004; Clark et al., 2016; M. Davis et al., 2017; Gray et al., 2018; Jones et al., 2017; Nelson et al., 2012; Perry et al., 2012; Pruett et al., 2023; Smith. et al., 2019; Song et al., 2018; Tumanova et al., 2020)^75,145–147,465,466,472,473,476,481–483^ | 12 (6%) |  |
|  |  |  |  |  |  |  |  |  |  |
|  | EEG | ERP; LPP; Asymmetry | Affective Posner Task (Perez-Edgar & Fox, 2005); Emotional Go/No-go Task; Task involving directed reappraisal (e.g., (DeCicco et al., 2012); Story Preview – Background Conversation – Child Narrative Sequence; Reappraisal Task | For frequency band, background conversation and ROI: Cronbach α = .91 - .99; for LPP: ICC = .43 - .70 |  |  | (Arnold et al., 2011; Forbes, Shaw et al., 2006; Gair et al., 2022; Hua et al., 2015; Kling & Brooker, 2024; Lewis et al., 2007; Zengin-Bolatkale et al., 2018)^129,149,152,340,484,485^ | 6 (3%) |  |
|  |  |  |  |  |  |  |  |  |  |
|  | ECG | Vagal Tone | Animated Cartoon Film; Resting State |  |  |  | (Gottman et al., 1996; Kennedy et al., 2004; Leary & Katz, 2004; Skibo et al., 2020)^122,468,469,486^ | 4 (2%) |  |
|  |  |  |  |  |  |  |  |  |  |
|  | EDA | Skin Conductance | Imaginative Storytelling Task; International Affective Picture System (IAPS; (Lang, 2005) | ICC = .99 |  |  | (Thibodeau‐Nielsen et al., 2021; Tumanova et al., 2020)^147,487^ | 2 (1%) |  |
|  |  |  |  |  |  |  |  |  |  |
|  | ECG | Heart Rate Variability | Resting State |  |  | Evaluation objectivity through blinded coders | (Berry et al., 2019; Kuiper et al., 2023)^97,361^ | 2 (1%) |  |
|  |  |  |  |  |  |  |  |  |  |
|  | Salvia Samples | Cortisol | Daily Measuring Points |  |  |  | (Frost et al., 2018)^456^ | 1 (0.5%) |  |
|  |  |  |  |  |  |  |  |  |  |
|  | fMRI | ROI | Resting State |  | Support for convergent validity: corr. ROI activity with ER in ERC |  | (Ewell et al., 2023) | 1 (0.5%) |  |
|  |  |  |  |  |  |  |  |  |  |
|  | ECG | R Waves | Memory Recall Task |  |  |  | (Gray et al., 2018)^476^ | 1 (0.5%) |  |
| **50 to 62 months** | **Frustration/Anger/Disappointment Eliciting Tasks** | | | | | | | |  |
|  | ECG | RSA | Not Sharing Task (Lab-TAB; (Goldsmith et al., 2010); Challenging Puzzles; Disappointment Task (e.g. (Cole et al., 1994; Saarni, 1984); Attractive Toy in a Transparent Box (e.g. (Gagne et al., 2011; Goldsmith, 1999)); Toy Removal; Puzzle Box Task (Eisenberg, Fabes, Karbon et al., 1996) |  | Support for convergent validity: corr. RSA with behavioral observation |  | (Dollar et al., 2020; Forbes, Fox et al., 2006; Gleason et al., 2021; Hassan et al., 2023; Perry et al., 2020; Shih et al., 2018; Zeytinoglu et al., 2022)^110,111,127,299,408,474,488^ | 7 (3.5%) |  |
|  |  |  |  |  |  |  |  |  |  |
|  | Functional near-infrared spectroscopy (fNIRS) | Brain Oxygenation | Frustration Emotion Task for Children (FETCH;(Perlman et al., 2015); Incredible Cake Kids (ICK; (Grabell et al., 2019) |  |  |  | (Grabell et al., 2019; Grabell et al., 2018; Santana & Grabell, 2023)^475,478,489^ | 3 (1.5%) |  |
|  |  |  |  |  |  |  |  |  |  |
|  | ECG | Heart Rate Variability | Attractive Toy in a Transparent Box (Lab-TAB; (Goldsmith & Rothbart, 1996) |  |  | Evaluation objectivity through blinded coders | (Berry et al., 2019; Kuiper et al., 2023; Zantinge et al., 2017)^97,359,361^ | 3 (1.5%) |  |
|  |  |  |  |  |  |  |  |  |  |
|  | Salvia Samples | Cortisol | Disappointing Task (Cole, 1986; Saarni, 1984); Challenging Tasks |  |  |  | (Grabell et al., 2015; Ip et al., 2024)^480,490^ | 2 (1%) |  |
|  |  |  |  |  |  |  |  |  |  |
|  | EEG | ERP; Asymmetry; Alpha Power | Adapted Affective Posner task (Perez-Edgar & Fox, 2005); Disappointment Task^232^ |  |  |  | (Forbes, Fox et al., 2006; Lugo-Candelas et al., 2017)^126,471^ | 2 (1%) |  |
|  |  |  |  |  |  |  |  |  |  |
|  | ECG | Vagal Tone | Delay of Gratification (Martin, 1981); Negative Mood Induction Stimulus for Children (MISC; (Cole, 1990) |  |  |  | (Cole, 1986; Santucci et al., 2008)^356,491^ | 2 (1%) |  |
|  | **Fear Eliciting Tasks** | | | | | | | |  |
|  | ECG | Heart Rate; Heart Rate Variability | Speech Task |  |  |  | (Hannesdóttir et al., 2010)^492^ | 1 (0.5%) |  |
|  | **Non Specific Emotion Eliciting Tasks or Mixed Emotions Tasks** | | | | | | | |  |
|  | EEG | ERP; LPP; Asymmetry | Story Preview – Background Conversation – Child narrative Sequence; International Affective Picture (IAPS; (Lang, 2005); Directed Reappraisal Task (DRT^493^); Affective Posner Task (Perez-Edgar & Fox, 2005)); Emotional Go/No-go Task; Adapted Attention Network Test – Child Version (Rueda et al., 2004); Audio-Image Pairing Task; Reappraisal Task | Split-half reliability for ERP = .48 - .86; For frequency band, background conversation and ROI: Cronbach α = .91 - .99; for LPP: ICC = .43 - .70 | Support for convergent validity: corr. of delta-beta correlation with ER strategies in questionnaires |  | (Arnold et al., 2011; Babkirk et al., 2015; DeCicco et al., 2012; Dennis, Cole et al., 2009; Dennis & Hajcak, 2009; Forbes, Shaw et al., 2006; Gair et al., 2022; Hua et al., 2015; Lewis et al., 2007; Myruski et al., 2022; Myruski et al., 2019; Myruski & Dennis-Tiwary, 2021; Usler et al., 2020; Zengin-Bolatkale et al., 2018) | 14 (7%) |  |
|  |  |  |  |  |  |  |  |  |  |
|  | ECG | RSA | Emotional audio/video clip; International Affective Picture System (IAPS; (Lang, 2005); Memory Recall Task; Attention Task; Neutral Video; Resting State; Joint Interaction Episodes; Interview with unfamiliar person; Emotional Go-No-Go Task; Resting State | ICC = .99; Cohen's κ > .90 |  |  | (Blandon et al., 2008; Clark et al., 2016; E. L. Davis et al., 2016; Gleason et al., 2021; Gray et al., 2018; Jones et al., 2017; Myruski et al., 2022; Perry, Dollar et al., 2018; Skibo et al., 2020; Song et al., 2018; Stifter et al., 2011; Tenenbaum et al., 2019; Tumanova et al., 2020)^75,123,145,147,154,395,406,468,476,482,483,488,494^ | 13 (6.5%) |  |
|  |  |  |  |  |  |  |  |  |  |
|  | fMRI | Volume; ROI | Resting State; Emotional Go-No-Go Task |  |  |  | (Ewell et al., 2023; Perlman & Pelphrey, 2010, 2011; Tottenham et al., 2010) | 4 (2%) |  |
|  |  |  |  |  |  |  |  |  |  |
|  | ECG | Vagal Tone | Animated Cartoon Film; Resting State |  |  |  | (Gottman et al., 1996; Kidwell & Barnett, 2007; Leary & Katz, 2004)^122,486,495^ | 3 (1.5%) |  |
|  |  |  |  |  |  |  |  |  |  |
|  | EDA | Skin Conductance | Imaginative Storytelling Task; International Affective Picture System (IAPS; (Lang, 2005) |  |  |  | (Thibodeau‐Nielsen et al., 2021; Tumanova et al., 2020)^147,487^ | 2 (1%) |  |
|  |  |  |  |  |  |  |  |  |  |
|  | ECG | Heart Rate Variability | Resting State |  |  | Evaluation objectivity through blinded coders | (Berry et al., 2019; Kuiper et al., 2023)^97,361^ | 2 (1%) |  |
|  |  |  |  |  |  |  |  |  |  |
|  | ECG | R Waves | Memory Recall Task |  |  |  | (Gray et al., 2018)^476^ | 1 (0.5%) |  |
|  |  |  |  |  |  |  |  |  |  |
|  | ECG | PEP | Interview with unfamiliar person |  |  |  | (Stifter et al., 2011)^395^ | 1 (0.5%) |  |

**Table 2d**

*Physiological measurement of emotion regulation for school-aged children (63 to 140 months old)*

| **Age range (months)** | **Measurement Method** | **Physiological Phenomenon (PP)** | **Task** | **Reliability** | **Validity** | **Objectivity** | **Studies measuring PP** | **Number/ Percentage of studies measuring PP** |  |
| --- | --- | --- | --- | --- | --- | --- | --- | --- | --- |
| **School** | | | | | | | | | |
| **63 to 75 months** | **Frustration/Anger/Disappointment Eliciting Tasks** | | | | | | | | |
|  | ECG | RSA | Not Sharing Task (Lab-TAB; (Goldsmith et al., 2010); Disappointment Task (e.g. (Cole et al., 1994; Saarni, 1984); Puzzle Task^399^; Attractive Toy in a Transparent Box (e.g. (Gagne et al., 2011; Goldsmith, 1999)); Toy Removal; Challenge task; Audiotaped Argument; Emotion Task (Gatzke-Kopp et al., 2015) | ICC = .99 | Support for convergent validity: corr. RSA with behavioral observation |  | (Baker et al., 2022; Distefano et al., 2021; El-Sheikh, 2001; Forbes, Fox et al., 2006; Fry et al., 2022; Hassan et al., 2023; Jones et al., 2017; Kahle et al., 2021; Moffitt et al., 2021; Perry et al., 2020; Shih et al., 2018; Tumanova et al., 2020; Zeytinoglu et al., 2022) | 13 (6.5%) |  |
|  |  |  |  |  |  |  |  |  |  |
|  | Functional near-infrared spectroscopy (fNIRS) | Brain Oxygenation | Frustration Emotion Task for Children (FETCH; (Perlman et al., 2015); Incredible Cake Kids (ICK) |  |  |  | (Grabell et al., 2019; Grabell et al., 2018)^475,478^ | 2 (1%) |  |
|  |  |  |  |  |  |  |  |  |  |
|  | EEG | ERP; LPP | Adapted Affective Posner task (Perez-Edgar & Fox, 2005); Reappraisal Task | for LPP: ICC = .43 - .70 |  |  | (Kling & Brooker, 2024; Lugo-Candelas et al., 2017)^149,496^ | 2 (1%) |  |
|  |  |  |  |  |  |  |  |  |  |
|  | ECG | Heart Rate Variability | Attractive Toy in a Transparent Box (Lab-TAB; (Goldsmith & Rothbart, 1996) |  |  | Evaluation objectivity through blinded coders | (Berry et al., 2019; Kuiper et al., 2023)^97,361^ | 2 (1%) |  |
|  |  |  |  |  |  |  |  |  |  |
|  | EDA | Skin Conductance | Challenge task; Emotion Task |  |  |  | (Fry et al., 2022; Moffitt et al., 2021)^497,498^ | 2 (1%) |  |
|  |  |  |  |  |  |  |  |  |  |
|  | ECG | Vagal Tone | Delay of Gratification (Martin, 1981) |  |  |  | (Santucci et al., 2008)^356^ | 1 (0.5%) |  |
|  |  |  |  |  |  |  |  |  |  |
|  | EEG | Asymmetry; Alpha Power | Disappointment Task (Cole et al., 1994) |  |  |  | (Forbes, Fox et al., 2006) | 1 (0.5%) |  |
|  |  |  |  |  |  |  |  |  |  |
|  | Salvia Samples | Cortisol | Disappointing Task (Cole, 1986; Saarni, 1984) |  |  |  | (Grabell et al., 2015)^480^ | 1 (0.5%) |  |
|  |  |  |  |  |  |  |  |  |  |
|  | fMRI | ROI | Frustrating Emotion Task for Children (FETCH) |  |  |  | (Perlman et al., 2015)^477^ | 1 (0.5%) |  |
|  | **Fear Eliciting Tasks** | | | | | | | |  |
|  | ECG | Heart Rate; Heart Rate Variability | Speech Task |  |  |  | (Hannesdóttir et al., 2010)^492^ | 1 (0.5%) |  |
|  | **Empathy Eliciting Tasks** | | | | | | | |  |
|  | ECG | Heart Rate Variability | Taped Crying Baby |  |  |  | (Fabes et al., 1994)^499^ | 1 (0.5%) |  |
|  | **Non Specific Emotion Eliciting Tasks or Mixed Emotions Tasks** | | | | | | | |  |
|  | EEG | ERP; LPP; Asymmetry | Affective Posner Task (Perez-Edgar & Fox, 2005); Emotional Go/No-go Task; Adapted Attention Network Test – Child Version (Rueda et al., 2004); Audio-Image Pairing Task; Task involving directed reappraisal (DRT^493^); Go-No-Go Task; International Affective Picture (IAPS; (Lang, 2005); Story Preview – Background Conversation – Child narrative Sequence; Resting State | Split-half reliability for ERP = .48 - .86; For frequency band, background conversation and ROI: Cronbach α = .91 - .99 | Support for convergent validity: corr. of delta-beta correlation with ER strategies in questionnaires |  | (Arnold et al., 2011; Babkirk et al., 2015; DeCicco et al., 2012; Dennis, Cole et al., 2009; Dennis & Hajcak, 2009; Forbes, Shaw et al., 2006; Gair et al., 2022; Gatzke-Kopp et al., 2020; Hua et al., 2015; Lewis et al., 2007; Myruski et al., 2022; Myruski et al., 2019; Myruski & Dennis-Tiwary, 2021; Usler et al., 2020; Zengin-Bolatkale et al., 2018)^119,131,301,321,369,372,373,478–480,488,489,491,492,499^ | 15 (7.5%) |  |
|  |  |  |  |  |  |  |  |  |  |
|  | ECG | RSA | Joint Interaction Episodes (Fries et al., 2005); Emotional audio/video clip; International Affective Picture System (IAPS; (Lang, 2005); Memory Recall Task; Attention Task; Resting State; Emotional Go-No-Go Task | ICC = .99; Cohen's κ > .90 |  |  | (Blandon et al., 2008; Clark et al., 2016; E. L. Davis et al., 2016; Gatzke-Kopp et al., 2020; Gentzler et al., 2009; Gray et al., 2018; Jones et al., 2017; Katz et al., 2015; Song et al., 2018; Tenenbaum et al., 2019; Tumanova et al., 2020; Whitson & El-Sheikh, 2003)^75,118,123–125,145,147,476,482,483,494,500^ | 12 (6%) |  |
|  |  |  |  |  |  |  |  |  |  |
|  | fMRI | Volume; ROI | Resting State; Emotional Go-No-Go Task; Reappraisal Task |  |  |  | (Dougherty et al., 2015; Ewell et al., 2023; Perlman & Pelphrey, 2010; Tottenham et al., 2010)^112,133,134,137,501^ | 5 (2.5%) |  |
|  |  |  |  |  |  |  |  |  |  |
|  | ECG | Vagal Tone | Animated Cartoon Film; Resting State; Neutral and Emotion-Eliciting Film; Resting State |  |  |  | (Klinge et al., 2023; Taskiran et al., 2018; Whitson & El-Sheikh, 2003)^122,486,495^ | 3 (1.5%) |  |
|  |  |  |  |  |  |  |  |  |  |
|  | EDA | Skin Conductance | Imaginative Storytelling Task; International Affective Picture System (IAPS; (Lang, 2005) |  |  |  | (Taskiran et al., 2018; Thibodeau‐Nielsen et al., 2021; Tumanova et al., 2020)^139,147,487^ | 3 (1.5%) |  |
|  |  |  |  |  |  |  |  |  |  |
|  | ECG | Heart Rate Variability; Heart Rate | Resting State; International Affective Picture System (IAPS) |  |  | Evaluation objectivity through blinded coders | (Klinge et al., 2023; Taskiran et al., 2018; Whitson & El-Sheikh, 2003) | 3 (1.5%) |  |
|  |  |  |  |  |  |  |  |  |  |
|  | ECG | Heart Rate Variability | Resting State |  |  |  | (Berry et al., 2019; Kuiper et al., 2023)^97,361^ | 2 (1%) |  |
|  |  |  |  |  |  |  |  |  |  |
|  | ECG | R Waves | Memory Recall Task (Fivush et al., 1990) |  |  |  | (Gray et al., 2018)^476^ | 1 (0.5%) |  |
|  |  |  |  |  |  |  |  |  |  |
|  | tDCS Stimulator | tDCS | Emotional Go-No-Go Task |  |  |  | (Estaji et al., 2024) | 1 (0.5%) |  |
|  |  |  |  |  |  |  |  |  |  |
|  | ECG | PEP | Go-No-Go Task |  |  |  | (Gatzke-Kopp et al., 2020)^500^ | 1 (0.5%) |  |
| **76 to 88 months** | **Frustration/Anger/Disappointment Eliciting Tasks** | | | | | | | |  |
|  | ECG | RSA | Disappointment Task (e.g. (Cole et al., 1994; Saarni, 1984); Puzzle Task^399^; Challenge task; Audiotaped Argument; Still Face Paradigm; Parent-Child Discussion; Card Sorting Task; Attractive Toy in a Transparent Box task (ATTB; (Goldsmith, 1999); Impossible Puzzle |  | Support for convergent validity: corr. RSA with behavioral observation |  | ^127,502^(Baker et al., 2022; Distefano et al., 2021; Dollar et al., 2020; El-Sheikh, 2001; Feurer et al., 2020; Forbes, Fox et al., 2006; Leaberry et al., 2018; Moffitt et al., 2021; Perry et al., 2020; Shih et al., 2018)^108,109,115,126,343,464,496,503–505^ | 11 (5.5%) |  |
|  |  |  |  |  |  |  |  |  |  |
|  | EEG | ERP; Asymmetry, Alpha Power | Adapted Attention Network Test – Child Version (Rueda et al., 2004); Audio-Image Pairing Task; Adapted Affective Posner Task (Perez-Edgar & Fox, 2005); Disappointment Task (Cole et al., 1994) | Split-half reliability for ERP = .48 - .86 |  |  | (Dennis & Hajcak, 2009; Forbes, Fox et al., 2006; Lugo-Candelas et al., 2017; Usler et al., 2020)^127,130,151,496^ | 4 (2%) |  |
|  |  |  |  |  |  |  |  |  |  |
|  | Functional near-infrared spectroscopy (fNIRS) | Brain Oxygenation | Frustration Emotion Task for Children (FETCH; (Perlman et al., 2015) |  |  |  | (Grabell et al., 2018)^475^ | 1 (0.5%) |  |
|  |  |  |  |  |  |  |  |  |  |
|  | ECG | Vagal Tone | Delay of Gratification (Martin, 1981) |  |  |  | (Santucci et al., 2008)^356^ | 1 (0.5%) |  |
|  |  |  |  |  |  |  |  |  |  |
|  | fMRI | ROI | Frustrating Emotion Task for Children (FETCH) |  |  |  | (Perlman et al., 2015)^477^ | 1 (0.5%) |  |
|  |  |  |  |  |  |  |  |  |  |
|  | ECG | Heart Rate | Attractive Toy in a Transparent Box (Lab-TAB; (Goldsmith & Rothbart, 1996) |  |  | Evaluation objectivity through blinded coders | (Kuiper et al., 2023) | 1 (0.5%) |  |
|  |  |  |  |  |  |  |  |  |  |
|  | EDA | Skin Conductance | Challenge task |  |  |  | (Moffitt et al., 2021)^497^ | 1 (0.5%) |  |
|  | **Fear Eliciting Tasks** | | | | | | | |  |
|  | ECG | Heart Rate; Herat Rate Variability | Speech Task; Venipuncture |  |  |  | (Constantin et al., 2022; Hannesdóttir et al., 2010)^121,492^ | 2 (1%) |  |
|  | **Empathy Eliciting Tasks** | | | | | | | |  |
|  | ECG | Heart Rate Variability | Taped Crying Baby |  |  |  | (Fabes et al., 1994)^499^ | 1 (0.5%) |  |
|  | **Non Specific Emotion Eliciting Tasks or Mixed Emotions Tasks** | | | | | | | |  |
|  | ECG | RSA | Attention Task; Resting State; Emotional Go-No-Go Task; Emotional Film Clips | Cohen's κ > .90 |  |  | (Alfano et al., 2020; Blandon et al., 2008; Feurer et al., 2020; Gentzler et al., 2009; Katz et al., 2015; Leaberry et al., 2018; Musser et al., 2013; Song et al., 2018; Tenenbaum et al., 2019; Whitson & El-Sheikh, 2003)^118,123–125,482,483,502–505^ | 10 (5%) |  |
|  |  |  |  |  |  |  |  |  |  |
|  | EEG | ERP; LPP; Asymmetry | Affective Posner Task (Perez-Edgar & Fox, 2005); Task involving directed reappraisal (e.g., DRT); Adapted Attention Network Test – Child Version (Rueda et al., 2004); International Affective Picture (IAPS; (Lang, 2005); Resting State |  | Support for convergent validity: corr. of delta-beta correlation with ER strategies in questionnaires |  | (Babkirk et al., 2015; DeCicco et al., 2014; DeCicco et al., 2012; Dennis, Cole et al., 2009; Dennis & Hajcak, 2009; Forbes, Shaw et al., 2006; Gair et al., 2022; Myruski et al., 2019; Myruski & Dennis-Tiwary, 2021; Zengin-Bolatkale et al., 2018)^129,130,320,390,391,484,485,493,506^ | 10 (5%) |  |
|  |  |  |  |  |  |  |  |  |  |
|  | (f)MRI | Volume; Whole Brain; ROI | Resting State; Emotional Go-No-Go Task; Reappraisal Task (Dennis, Malone & Chen, 2009); Card-Guessing Game (Delgado et al., 2005) |  |  |  | (Dougherty et al., 2015; Ewell et al., 2023; Gilbert et al., 2019; Perlman & Pelphrey, 2010, 2011; Tottenham et al., 2010; Urbain et al., 2019; Urbain et al., 2017)^112,133–137,501,507^ | 8 (4%) |  |
|  |  |  |  |  |  |  |  |  |  |
|  | ECG | Heart Rate Variability; Heart Rate | Resting State; International Affective Picture System (IAPS); Emotional Movie Clip; Go–Nogo; Stroop Color-word^508^; Working Memory Task (Digit span; (Miyake, 2001); Attractive Toy in a Transparent Box (Lab-TAB; (Goldsmith & Rothbart, 1996) |  |  | Evaluation objectivity through blinded coders | (Alfano et al., 2020; Constantin et al., 2022; Klinge et al., 2023; Taskiran et al., 2018; Whitson & El-Sheikh, 2003; Woltering et al., 2016)^95,116–118,127,502,506^ | 7 (3.5%) |  |
|  |  |  |  |  |  |  |  |  |  |
|  | ECG | Vagal Tone | Animated Cartoon Film; Resting State |  |  |  | (Gottman et al., 1996; Leary & Katz, 2004)^122,486^ | 2 (1%) |  |
|  |  |  |  |  |  |  |  |  |  |
|  | MEG | Whole Brain | Emotional Go-No-Go Task |  |  |  | (Urbain et al., 2019; Urbain et al., 2017) | 2 (1%) |  |
|  |  |  |  |  |  |  |  |  |  |
|  | tDCS Stimulator | tDCS | Emotional Go-No-Go Task |  |  |  | (Estaji et al., 2024) | 1 (0.5%) |  |
|  |  |  |  |  |  |  |  |  |  |
|  | EDA | Skin Conductance | International Affective Picture System (IAPS) |  |  |  | (Taskiran et al., 2018) | 1 (0.5%) |  |
|  |  |  |  |  |  |  |  |  |  |
|  | EEG | Delta-Beta Correlation | Resting State |  |  |  | (Myruski et al., 2022) | 1 (0.5%) |  |
| **89 to 101 months** | **Frustration/Anger/Disappointment Eliciting Tasks** | | | | | | | |  |
|  | ECG | RSA | Disappointment Task^232^; Puzzle Task (Eisenberg, Fabes, Karbon et al., 1996); Challenge Task; Audiotaped Argument; Parent-Child Discussion; Card Sorting Task; Social Rejection Task (Silk et al., 2012); Attractive Toy in a Transparent Box task (ATTB; (Goldsmith, 1999) |  | Support for convergent validity: corr. RSA with behavioral observation |  | ^127,502^(Baker et al., 2022; Breaux et al., 2018; Distefano et al., 2021; El-Sheikh, 2001; Feurer et al., 2020; Forbes, Fox et al., 2006; Leaberry et al., 2018; Moffitt et al., 2021; Perry et al., 2020; Tang et al., 2024)^108,109,115,126,343,464,496,503–505^ | 10 (5%) |  |
|  |  |  |  |  |  |  |  |  |  |
|  | EDA | Skin Conductance | Challenge task; Social Rejection Task (Silk et al., 2012); Puzzle Task |  |  |  | (Breaux et al., 2018; Moffitt et al., 2021)^497,509^ | 2 (1%) |  |
|  |  |  |  |  |  |  |  |  |  |
|  | EEG | Asymmetry; Alpha Power | Disappointment Task^232^ |  |  |  | (Forbes, Fox et al., 2006) | 1 (0.5%) |  |
|  |  |  |  |  |  |  |  |  |  |
|  | fMRI | ROI | Frustrating Emotion Task for Children (FETCH) |  |  |  | (Perlman et al., 2015)^477^ | 1 (0.5%) |  |
|  |  |  |  |  |  |  |  |  |  |
|  | ECG | Vagal Tone | Peer Provocation |  | Support for convergent validity: corr. vagal tone with self-reported ER |  | (Hessler & Fainsilber Katz, 2007) | 1 (0.5%) |  |
|  |  |  |  |  |  |  |  |  |  |
|  | ECG | Heart Rate | Peer Provocation |  | Support for convergent validity: corr. HR with self-reported ER |  | (Hessler & Fainsilber Katz, 2007) | 1 (0.5%) |  |
|  | **Fear Eliciting Tasks** | | | | | | | |  |
|  | ECG | Heart Rate; Herat Rate Variability | Speech Task; Venipuncture |  |  |  | (Constantin et al., 2022; Hannesdóttir et al., 2010)^121,492^ | 2 (1%) |  |
|  | **Empathy Eliciting Tasks** | | | | | | | |  |
|  | ECG | Heart Rate Variability | Taped Crying Baby |  |  |  | (Fabes et al., 1994)^499^ | 1 (0.5%) |  |
|  | **Non Specific Emotion Eliciting Tasks or Mixed Emotions Tasks** | | | | | | | |  |
|  | EEG | ERP; LPP; Asymmetry | Adapted Attention Network Test (Rueda et al., 2004); Audio-Image Pairing Task; Go/No-Go Task (e.g. (Garavan et al., 1999); International Affective Picture System (IAPS; (Lang, 2005); Cognitive Reappraisal Task; Directed Reappraisal Task (DRT^493^); Resting State | Split-half reliability for ERP = .48 - .86 |  |  | (DeCicco et al., 2014; Dennis & Hajcak, 2009; Forbes, Fox et al., 2006; Granic et al., 2012; Hum et al., 2013; Lamm et al., 2011; Leventon & Bauer, 2016; Leventon et al., 2019; Lewis et al., 2008; Liu et al., 2022; Liu et al., 2019; Myruski et al., 2019; Myruski & Dennis-Tiwary, 2021; Stieben et al., 2007; Übel et al., 2015; Usler et al., 2020; van Cauwenberge et al., 2017)^112,119,131,132,373,488,491,492,509,512–518^ | 17 (8.5%) |  |
|  |  |  |  |  |  |  |  |  |  |
|  | ECG | RSA | Resting State; Emotional Go-No-Go Task, Emotional Film Clips; Resting State; Parent-Child Interaction/Collaboration | Cohen's κ > .90 | Validity of RSA as a marker of parasympathetically mediated cardiac activity has been confirmed through pharmacological blockade |  | (Alfano et al., 2020; Eisenberg, Fabes, Guthrie et al., 1996; Feurer et al., 2020; Gentzler et al., 2009; Katz & Gurtovenko, 2015; Leaberry et al., 2018; Musser et al., 2013; Musser et al., 2018; Song et al., 2018; Spang et al., 2022; Tang et al., 2024; Tenenbaum et al., 2019; Vasilev et al., 2009; Whitson & El-Sheikh, 2003; Zhang et al., 2017)^118,123–125,219,399,483,502–505,510–513^ | 15 (7.5%) |  |
|  |  |  |  |  |  |  |  |  |  |
|  | (f)MRI | Volume; Whole Brain; ROI | Resting State; Emotional Go-No-Go Task; Reappraisal Task; Images International Affective Picture Series; Cognitive Reappraisal Task(Ochsner et al., 2004); Event-Related Facial Emotion Processing Task; Card-Guessing Game (Delgado et al., 2005) |  | Convergent validity: corr. volume, BOLD signal with ER in questionnaires (Pagliaccio et al., 2014) |  | (Belden et al., 2014; Dougherty et al., 2015; Ewell et al., 2023; Gilbert et al., 2019; Murphy et al., 2016; Pagliaccio et al., 2014; Perlman & Pelphrey, 2010, 2011; Tottenham et al., 2010; Urbain et al., 2019; Urbain et al., 2017)^113,133–137,501,507,514,515^ | 11 (5.5%) |  |
|  |  |  |  |  |  |  |  |  |  |
|  | ECG | Heart Rate Variability; Heart Rate | Resting State; International Affective Picture System (IAPS); Emotinal Movie Clip; Go–Nogo; Stroop Color-word^508^; Working Memory Task (Digit span; (Miyake, 2001) |  | RMSSD values (*M* = 4.12; *SD* = 0.46) were comparable to values reported in Nunan et al., 2010 (*M* = 3.49; *SD* = 0.26) |  | (Alfano et al., 2020; Bunford et al., 2017; Constantin et al., 2022; Hessler & Fainsilber Katz, 2007; Klinge et al., 2023; Taskiran et al., 2018; Whitson & El-Sheikh, 2003; Woltering et al., 2016)^116–118,127,502,506,511,525^ | 8 (4%) |  |
|  |  |  |  |  |  |  |  |  |  |
|  | ECG | Vagal Tone | Film Clips; Resting State |  |  |  | (Eisenberg, Fabes, Guthrie et al., 1996; Gottman et al., 1996; Hessler & Fainsilber Katz, 2007; Leary & Katz, 2004)^122,156,399,486^ | 4 (2%) |  |
|  |  |  |  |  |  |  |  |  |  |
|  | MEG | Whole Brain | Emotional Go-No-Go Task; NimStim set of facial expressions ((Tottenham et al., 2009); Karolinska Directed Emotional Faces (KDEF) |  |  |  | (Urbain et al., 2019; Urbain et al., 2017; Wessing et al., 2015) | 3 (1.5%) |  |
|  |  |  |  |  |  |  |  |  |  |
|  | tDCS Stimulator | tDCS | Emotional Go-No-Go Task |  |  |  | (Estaji et al., 2024) | 1 (0.5%) |  |
|  |  |  |  |  |  |  |  |  |  |
|  | EDA | Skin Conductance | International Affective Picture System (IAPS) |  |  |  | (Taskiran et al., 2018) | 1 (0.5%) |  |
|  |  |  |  |  |  |  |  |  |  |
|  | ECG | PEP | Resting State, Parent-Child Interaction |  |  |  | (Musser et al., 2018)^511^ | 1 (0.5%) |  |
| **102 to 114 months** | **Frustration/Anger/Disappointment Eliciting Tasks** | | | | | | | |  |
|  | ECG | RSA | Disappointment Task^232^; Puzzle Task^399^; Challenge Task; Audiotaped Argument; Parent-Child Discussion; Card Sorting Task; Social Rejection Task (Silk et al., 2012); Still-Face Paradigm (Mesman et al., 2009; Tronick et al., 1978); Iowa Gambling Task |  | Support for convergent validity: corr. RSA with behavioral observation; Validity of RSA as a marker of parasympathetically mediated cardiac activity has been confirmed through pharmacological blockade |  | (Baker et al., 2022; Breaux et al., 2018; El-Sheikh, 2001; Feurer et al., 2020; Forbes, Fox et al., 2006; Leaberry et al., 2018; Lochman et al., 2019; Moffitt et al., 2021; Perry et al., 2020; Tang et al., 2024) | 11 (5.5%) |  |
|  |  |  |  |  |  |  |  |  |  |
|  | fMRI | ROI | Frustrating Emotion Task for Children (FETCH); Sad Images International Affective Picture Series; Modified Emotion Label Task (Hariri et al., 2000; Lieberman et al., 2007) |  |  |  | (Belden et al., 2014; Colich et al., 2017; Perlman et al., 2015)^477,514,516^ | 3 (1.5%) |  |
|  |  |  |  |  |  |  |  |  |  |
|  | EDA | Skin Conductance | Challenge Task; Chatroom Interact Task with manipulated peer acceptance and rejection (Silk et al., 2012); Impossible Puzzle Task (e.g., (Somers et al., 2015); Iowa Gambling Task |  |  |  | (Lochman et al., 2019; McQuade & Breaux, 2017; Moffitt et al., 2021)^497,517,518^ | 3 (1.5%) |  |
|  |  |  |  |  |  |  |  |  |  |
|  | ECG | Heart Rate; Herat Rate Variability | Peer Provocation; Sad Film |  | Support for convergent validity: corr. vagal tone with self-reported ER |  | (Fiskum et al., 2019; Hessler & Fainsilber Katz, 2007) | 2 (1%) |  |
|  |  |  |  |  |  |  |  |  |  |
|  | EEG | Asymmetry; Alpha Power | Disappointment Task^232^ |  |  |  | (Forbes, Fox et al., 2006) | 1 (0.5%) |  |
|  |  |  |  |  |  |  |  |  |  |
|  | ECG | Vagal Tone | Peer Provocation |  | Support for convergent validity: corr. HR with self-reported ER |  | (Hessler & Fainsilber Katz, 2007) | 1 (0.5%) |  |
|  | **Fear Eliciting Tasks** | | | | | | | |  |
|  | ECG | Heart Rate; Herat Rate Variability | Speech Task; Venipuncture |  |  |  | (Constantin et al., 2022; Hannesdóttir et al., 2010)^121,492^ | 2 (1%) |  |
|  | **Empathy Eliciting Tasks** | | | | | | | |  |
|  | ECG | Heart Rate Variability | Taped Crying Baby |  |  |  | (Fabes et al., 1994)^499^ | 1 (0.5%) |  |
|  | **Non Specific Emotion Eliciting Tasks or Mixed Emotions Tasks** | | | | | | | |  |
|  | EEG | ERP; LPP; Asymmetry | Adapted Attention Network Test – Child Version (Rueda et al., 2004); Go/No-Go Task (e.g. (Garavan et al., 1999); International Affective Picture System (IAPS; (Lang, 2005); Cognitive Reappraisal Task; Resting State |  |  |  | (DeCicco et al., 2014; Dennis, Cole et al., 2009; Dennis & Hajcak, 2009; Forbes, Shaw et al., 2006; Granic et al., 2012; Hum et al., 2013; Lamm et al., 2011; Leventon & Bauer, 2016; Leventon et al., 2019; Lewis et al., 2008; Liu et al., 2022; Liu et al., 2019; Myruski & Dennis-Tiwary, 2021; Stieben et al., 2007; Übel et al., 2015; van Cauwenberge et al., 2017; Wang et al., 2018)^114,129–131,320,391,508,519–528^ | 17 (8.5%) |  |
|  |  |  |  |  |  |  |  |  |  |
|  | ECG | RSA | Emotional Go-No-Go Task; Emotional Film Clips; Resting State; Parent-Child Interaction/Collaboration | Cohen's κ > .90 |  |  | (Alfano et al., 2020; Eisenberg, Fabes, Guthrie et al., 1996; Feurer et al., 2020; Gentzler et al., 2009; Katz et al., 2015; Kiser et al., 2019; Leaberry et al., 2018; Lochman et al., 2019; Musser et al., 2013; Musser et al., 2018; Pang & Beauchaine, 2013; Tang et al., 2024; Tenenbaum et al., 2019; Vasilev et al., 2009; Whitson & El-Sheikh, 2003; Zhang et al., 2017)^118,123–125,128,219,399,502–505,510–513,518^ | 16 (8%) |  |
|  |  |  |  |  |  |  |  |  |  |
|  | fMRI | Volume, Whole Brain; ROI | Emotional Go-No-Go Task; Reappraisal Task; Cognitive Reappraisal Task (e.g. (Ochsner et al., 2004); Event-Related Facial Emotion Processing Task; Mood-Repair Task; N-Back (e.g. (Cohen & D'Esposito, 2016); Directed Reappraisal Task (DRT^493^); International Affective Picture (IAPS; (Lang, 2005); Card-Guessing Game (Delgado et al., 2005) |  | Convergent validity: corr. volume, BOLD signal with ER in questionnaires (Pagliaccio et al., 2014) |  | (Dougherty et al., 2015; Eisenberg, Fabes, Karbon et al., 1996; Geckeler et al., 2022; Gilbert et al., 2019; Huffman & Oshri, 2022; Joormann et al., 2012; Murphy et al., 2016; Pagliaccio et al., 2014; Perlman & Pelphrey, 2010, 2011; Tottenham et al., 2010; Urbain et al., 2019; Urbain et al., 2017)^110,111,121,122,128,129,381,501,508,524,535–537^ | 13 (6.5%) |  |
|  |  |  |  |  |  |  |  |  |  |
|  | ECG | Heart Rate Variability; Heart Rate | Resting State; International Affective Picture System (IAPS); Emotional Movie Clip; Go–No-Go Stroop Color-word^508^; Working Memory Task (Digit span; (Miyake, 2001) |  | RMSSD values (*M* = 4.12; *SD* = 0.46) were comparable to values reported in Nunan et al., 2010 (*M* = 3.49; *SD* = 0.26) |  | (Alfano et al., 2020; Bunford et al., 2017; Constantin et al., 2022; Fiskum et al., 2017; Fiskum et al., 2019; Hessler & Fainsilber Katz, 2007; Klinge et al., 2023; Osborne et al., 2021; Taskiran et al., 2018; Whitson & El-Sheikh, 2003; Woltering et al., 2016) | 11 (5.5%) |  |
|  |  |  |  |  |  |  |  |  |  |
|  | MEG | Whole Brain | Emotional Go-No-Go Task; NimStim set of facial expressions (Tottenham et al., 2009); Karolinska Directed Emotional Faces – KDEF set |  |  |  | (Urbain et al., 2019; Urbain et al., 2017; Wessing et al., 2015) | 3 (1.5%) |  |
|  |  |  |  |  |  |  |  |  |  |
|  | EDA | Skin Conductance | International Affective Picture System (IAPS); Resting State |  |  |  | (Lochman et al., 2019; Taskiran et al., 2018)^139,518^ | 2 (1%) |  |
|  |  |  |  |  |  |  |  |  |  |
|  | ECG | Vagal Tone | Resting State; Film Clips |  |  |  | (Hessler & Fainsilber Katz, 2007; Leary & Katz, 2004) | 2 (1%) |  |
|  |  |  |  |  |  |  |  |  |  |
|  | ECG | PEP | Resting State, Parent-Child Interaction |  |  |  | (Musser et al., 2018)^511^ | 1 (0.5%) |  |
|  |  |  |  |  |  |  |  |  |  |
|  | tDCS Stimulator | tDCS | Emotional Go-No-Go Task |  |  |  | (Estaji et al., 2024) | 1 (0.5%) |  |
| **115 to 127 months** | **Frustration/Anger/Disappointment Eliciting Tasks** | | | | | | | |  |
|  | ECG | RSA | Puzzle Task ((Eisenberg, Fabes, Guthrie et al., 1996); Challenge task; Audiotaped Argument; Parent-Child Discussion; Card Sorting Task; Social Rejection Task (Silk et al., 2012); Iowa Gambling Task; Mother‐Youth peer Problem‐Solving Conversation (Connor-Smith et al., 2000); Impossible Puzzle |  |  |  | (Baker et al., 2022; Breaux et al., 2018; Dollar et al., 2020; El-Sheikh, 2001; Feurer et al., 2020; Leaberry et al., 2018; Lochman et al., 2019; Moffitt et al., 2021; Perry et al., 2020; Tang et al., 2024; Tu et al., 2019)^110,111,117,219,426,497,504,505,509,518,529^ | 11 (5.5%) |  |
|  |  |  |  |  |  |  |  |  |  |
|  | EDA | Skin Conductance | Challenge task; Chatroom Interact Task with manipulated peer acceptance and rejection (Silk et al., 2012); Impossible Puzzle Task (e.g., (Somers et al., 2015); Iowa Gambling Task |  |  |  | (Lochman et al., 2019; McQuade & Breaux, 2017; Moffitt et al., 2021)^497,517,518^ | 3 (1.5%) |  |
|  |  |  |  |  |  |  |  |  |  |
|  | ECG | Heart Rate; Herat Rate Variability | Peer Provocation; Sad Film |  | Support for convergent validity: corr. HR with self-reported ER |  | (Fiskum et al., 2019; Hessler & Fainsilber Katz, 2007) | 2 (1%) |  |
|  |  |  |  |  |  |  |  |  |  |
|  | ECG | Vagal Tone | Peer Provocation; Stress paradigm (inter adult anger) |  | Support for convergent validity: corr. HR with self-reported ER |  | (Feldman, 2015; Hessler & Fainsilber Katz, 2007) | 2 (1%) |  |
|  |  |  |  |  |  |  |  |  |  |
|  | fMRI | ROI | Sad Images International Affective Picture Series |  |  |  | (Belden et al., 2014)^514^ | 1 (0.5%) |  |
|  | **Fear Eliciting Task** | | | | | | | |  |
|  | ECG | Heart Rate Variability | Venipuncture |  |  |  | (Constantin et al., 2022) | 1 (0.5%) |  |
|  | **Non Specific Emotion Eliciting Tasks or Mixed Emotions Tasks** | | | | | | | |  |
|  | ECG | RSA | Emotional Go-No-Go Task; Emotional/Neutral Film Clips; Resting State; Parent-Child Interaction/Collaboration | Cohen's κ > .90 |  |  | (Alfano et al., 2020; Debeuf et al., 2020; Eisenberg, Fabes, Karbon et al., 1996; Feurer et al., 2020; Gentzler et al., 2009; Katz et al., 2015; Kiser et al., 2019; Leaberry et al., 2018; Lochman et al., 2019; Musser et al., 2013; Musser et al., 2018; Neuhaus et al., 2014; Pang & Beauchaine, 2013; Tang et al., 2024; Tenenbaum et al., 2019; Vasilev et al., 2009; Whitson & El-Sheikh, 2003; Zhang et al., 2017)^118,123–125,128,219,399,502–505,510–513,518,530,531^ | 18 (9%) |  |
|  |  |  |  |  |  |  |  |  |  |
|  | (f)MRI | Volume; Whole Brain; ROI | Emotional Go-No-Go Task; Cognitive Reappraisal Task (e.g. (Ochsner et al., 2004); Event-Related Facial Emotion Processing Task; Modified Emotion Label Task (Hariri et al., 2000; Lieberman et al., 2007); Mood-Repair Task; Reappraisal Task; N-Back (e.g. (Cohen & D'Esposito, 2016); Emotional Pictures; Card-Guessing Game (Delgado et al., 2005) |  | Convergent validity: corr. volume, BOLD signal with ER in questionnaires (Pagliaccio et al., 2014) |  | (Colich et al., 2017; Geckeler et al., 2022; Gilbert et al., 2019; Huffman & Oshri, 2022; Joormann et al., 2012; Lin et al., 2024; Murphy et al., 2016; Pagliaccio et al., 2014; Perlman & Pelphrey, 2010, 2011; Tottenham et al., 2010; Urbain et al., 2019; Urbain et al., 2017; Warren et al., 2020)^112,113,133–136,138,507,515,516,532–535^ | 14 (7%) |  |
|  |  |  |  |  |  |  |  |  |  |
|  | EEG | ERP; LPP | Go-No-Go Task (e.g. (Garavan et al., 1999); International Affective Picture System (IAPS; (Lang, 2005); Cognitive Reappraisal Task; Chinese Affective Picture System (CAPS) with Reactivity and Regulation-Image Task (REAR-I) |  |  |  | (Connell et al., 2020; Deng et al., 2021; Deng & Zhang, 2020; Dennis & Hajcak, 2009; Granic et al., 2012; Hum et al., 2013; Lamm et al., 2011; Lewis et al., 2008; Liu et al., 2022; Liu et al., 2019; Stieben et al., 2007; Übel et al., 2015; van Cauwenberge et al., 2017)^114,115,130,131,508,519–524,536,537^ | 13 (6.5%) |  |
|  |  |  |  |  |  |  |  |  |  |
|  | ECG | Heart Rate Variability; Heart Rate | Resting State, International Affective Picture System (IAPS); Emotional Movie Clip; Go–Nogo; Stroop Color-word (Stieben et al., 2007); Working Memory Task (Digit span; (Miyake, 2001) |  | RMSSD values (*M* = 4.12; *SD* = 0.46) were comparable to values reported in Nunan et al., 2010 (*M* = 3.49; *SD* = 0.26) |  | (Alfano et al., 2020; Bunford et al., 2017; Constantin et al., 2022; Fiskum et al., 2017; Fiskum et al., 2019; Hessler & Fainsilber Katz, 2007; Klinge et al., 2023; Osborne et al., 2021; Taskiran et al., 2018; Whitson & El-Sheikh, 2003; Woltering et al., 2016)^118–121,139,156,157,159,503,538,539^ | 11 (5.5%) |  |
|  |  |  |  |  |  |  |  |  |  |
|  | ECG | Vagal Tone | Resting State; Film Clips |  |  |  | (Eisenberg, Fabes, Karbon et al., 1996; Feldman, 2015; Hessler & Fainsilber Katz, 2007; Leary & Katz, 2004)^93,122,156,399^ | 4 (2%) |  |
|  |  |  |  |  |  |  |  |  |  |
|  | MEG | Whole Brain | Emotional Go-No-Go Task; NimStim set of facial expressions (Tottenham et al., 2009); Karolinska Directed Emotional Faces – KDEF set |  |  |  | (Urbain et al., 2019; Urbain et al., 2017; Wessing et al., 2015) | 3 (1.5%) |  |
|  |  |  |  |  |  |  |  |  |  |
|  | EDA | Skin Conductance | International Affective Picture System (IAPS) |  |  |  | (Lochman et al., 2019; Taskiran et al., 2018)^139,518^ | 2 (1%) |  |
|  |  |  |  |  |  |  |  |  |  |
|  | ECG | PEP | Resting State; Parent-Child Interaction |  |  |  | (Musser et al., 2018)^511^ | 1 (1%) |  |
|  |  |  |  |  |  |  |  |  |  |
|  | tDCS Stimulator | tDCS | Emotional Go-No-Go Task |  |  |  | (Estaji et al., 2024) | 1 (1%) |  |
|  |  |  |  |  |  |  |  |  |  |
|  | Salvia Samples | Cortisol | Film Clips |  |  |  | (Debeuf et al., 2020)^530^ | 1 (1%) |  |
| **128 to 140 months** | **Frustration/Anger/Disappointment Eliciting Tasks** | | | | | | | |  |
|  | ECG | RSA | Parent-Child Discussion; Card Sorting Task; Social Rejection Task (Silk et al., 2012); Iowa Gambling Task; Mother‐Youth peer Problem‐Solving Conversation (Connor-Smith et al., 2000); Stressor Paradigm (unsolvable anagrams) |  |  |  | (Breaux et al., 2018; Feurer et al., 2020; Lochman et al., 2019; Tu et al., 2019; Wielgus et al., 2016)^504,509,518,529,540^ | 5 (2.5%) |  |
|  |  |  |  |  |  |  |  |  |  |
|  | EDA | Skin Conductance | Chatroom Interact Task with manipulated peer acceptance and rejection (Silk et al., 2012); Impossible Puzzle Task (e.g., (Somers et al., 2015); Iowa Gambling Task |  |  |  | (Lochman et al., 2019; McQuade & Breaux, 2017)^517,518^ | 2 (1%) |  |
|  |  |  |  |  |  |  |  |  |  |
|  | fMRI | ROI | Sad Images International Affective Picture Series |  |  |  | (Belden et al., 2014)^514^ | 1 (0.5%) |  |
|  |  |  |  |  |  |  |  |  |  |
|  | ECG | Heart Rate Variability | Sad Film |  |  |  | (Fiskum et al., 2019) | 1 (0.5%) |  |
|  | **Fear Eliciting Task** | | | | | | | |  |
|  | ECG | Heart Rate Variability | Venipuncture |  |  |  | (Constantin et al., 2022) | 1 (0.5%) |  |
|  | **Non Specific Emotion Eliciting Tasks or Mixed Emotions Tasks** | | | | | | | |  |
|  | ECG | RSA | Puzzle Task (Eisenberg, Fabes, Karbon et al., 1996); Emotional Go-No-Go Task, Audiotaped Argument; Resting State; Emotional/Neutral Film Clips; Parent-Child Interaction; Baseline Vanilla | Cohen's κ > .90; Cronbach α = .80 - .87 |  |  | (Alfano et al., 2020; Debeuf et al., 2020; Eisenberg, Fabes, Karbon et al., 1996; El-Sheikh, 2001; Feurer et al., 2020; Fox et al., 2019; Katz & Gurtovenko, 2015; Kiser et al., 2019; Lisitsa et al., 2021; Lochman et al., 2019; Mezulis et al., 2015; Musser et al., 2013; Musser et al., 2018; Pang & Beauchaine, 2013; Perry et al., 2020; Tenenbaum et al., 2019; Vasilev et al., 2009; Whitson & El-Sheikh, 2003; Wielgus et al., 2016; Zhang et al., 2017)^110,117,118,123,125,128,148,399,502–504,510–513,518,530,540–542^ | 20 (10%) |  |
|  |  |  |  |  |  |  |  |  |  |
|  | EEG | ERP | Go/No-Go Task (e.g. (Garavan et al., 1999); International Affective Picture System (IAPS; (Lang, 2005); Cognitive Reappraisal Task; Chinese Affective Picture System (CAPS); with Reactivity and Regulation-Image Task (REAR-I); Distraction Task (Zhang et al., 2013) |  |  |  | (Connell et al., 2020; Deng et al., 2021; Deng & Zhang, 2020; Granic et al., 2012; Hum et al., 2013; Lamm et al., 2011; Lewis et al., 2008; Liu et al., 2022; Liu et al., 2019; Stieben et al., 2007; Übel et al., 2015; van Cauwenberge et al., 2017; Zhang et al., 2013; Zhang et al., 2016)^114,115,131,508,519–524,536,537,543^ | 14 (7%) |  |
|  |  |  |  |  |  |  |  |  |  |
|  | (f)MRI | Volume; Whole Brain; ROI | Emotional Go-No-Go Task; Emotional Film Clips; Cognitive Reappraisal Task (e.g. (Ochsner et al., 2004); Event-Related Facial Emotion Processing Task; Modified Emotion Label Task (Hariri et al., 2000; Lieberman et al., 2007); Mood-Repair Task; Reappraisal Task; Emotional Pictures |  | Convergent validity: corr. volume, BOLD signal with ER in questionnaires (Pagliaccio et al., 2014) |  | (Colich et al., 2017; Gentzler et al., 2009; Joormann et al., 2012; Lin et al., 2024; Murphy et al., 2016; Pagliaccio et al., 2014; Perlman & Pelphrey, 2010, 2011; Tottenham et al., 2010; Urbain et al., 2019; Urbain et al., 2017; Warren et al., 2020)^112,113,124,133–136,138,515,516,532,535^ | 12 (6%) |  |
|  |  |  |  |  |  |  |  |  |  |
|  | ECG | Heart Rate Variability; Heart Rate | Resting State; International Affective Picture System (IAPS); Emotional Movie Clip; Go-No-Go Stroop Color-word (Stieben et al., 2007); Working Memory Task (Digit span; (Miyake, 2001) |  |  |  | (Alfano et al., 2020; Constantin et al., 2022; Fiskum et al., 2017; Fiskum et al., 2019; Klinge et al., 2023; Osborne et al., 2021; Taskiran et al., 2018; Whitson & El-Sheikh, 2003; Woltering et al., 2016)^118–121,139,159,503,538,539^ | 9 (4.5%) |  |
|  |  |  |  |  |  |  |  |  |  |
|  | MEG | Whole Brain | Emotional Go-No-Go Task; NimStim set of facial expressions (Tottenham et al., 2009); Karolinska Directed Emotional Faces – KDEF set |  |  |  | (Urbain et al., 2019; Urbain et al., 2017; Wessing et al., 2015) | 3 (1.5%) |  |
|  |  |  |  |  |  |  |  |  |  |
|  | ECG | Vagal Tone | Resting State; Film Clips |  |  |  | (Eisenberg, Fabes, Guthrie et al., 1996; Leary & Katz, 2004)^122,399^ | 2 (1%) |  |
|  |  |  |  |  |  |  |  |  |  |
|  | EDA | Skin Conductance | International Affective Picture System (IAPS); Resting State |  |  |  | (Lochman et al., 2019; Taskiran et al., 2018)^139,518^ | 2 (1%) |  |
|  |  |  |  |  |  |  |  |  |  |
|  | Salvia Samples | Cortisol | Film Clips; Daily Measures |  | Support for convergent validity: corr. cortisol with ER strategies in ERQ-CA |  | (Debeuf et al., 2020; Rnic et al., 2022)^155,530^ | 2 (1%) |  |
|  |  |  |  |  |  |  |  |  |  |
|  | tDCS Stimulator | tDCS | Emotional Go-No-Go Task |  |  |  | (Estaji et al., 2024) | 1 (0.5%) |  |
|  |  |  |  |  |  |  |  |  |  |
|  | ECG | PEP | Resting State, Parent-Child Interaction |  |  |  | (Musser et al., 2018)^511^ | 1 (0.5%) |  |

**Table 2e**

*Physiological measurement of emotion regulation for teens (141 to 192 months old)*

| **Age range (months)** | **Measurement Method** | **Physiological Phenomenon (PP)** | **Task** | **Reliability** | **Validity** | **Objectivity** | **Studies measuring PP** | **Number/ Percentageof studies measuring PP** |  |
| --- | --- | --- | --- | --- | --- | --- | --- | --- | --- |
| **Teen** | | | | | | | | | |
| **141 to 153 months** | **Frustration/Anger/Disappointment Eliciting Tasks** | | | | | | | | |
|  | ECG | RSA | Puzzle Task^399^; Audiotaped Argument; Card Sorting Task; Social Rejection Task ((Silk et al., 2012)Silk et al., 2012);Parent-Child Interaction; Iowa Gambling Task (Bechara et al., 1994); Mother‐Youth peer Problem‐Solving Conversation ((Connor-Smith et al., 2000)Connor‐Smith et al., 2000); Stressor Paradigm (unsolvable anagrams); Parent-Child Conflict Discussion Task^544^; Socially Challenging Task^545^ |  |  |  | (Breaux et al., 2018; Byrd et al., 2021; Eisenberg, Fabes, Karbon et al., 1996; El-Sheikh, 2001; Guy et al., 2014; Lochman et al., 2019; Musser et al., 2018; Perry et al., 2020; Tu et al., 2019; Wielgus et al., 2016)^108,115,381,510,520,527,540,547,553,554^ | 10 (5%) |  |
|  |  |  |  |  |  |  |  |  |  |
|  | EDA | Skin Conductance | Chatroom Interact Task with manipulated peer acceptance and rejection (Silk et al., 2012) Impossible Puzzle Task (e.g., (Somers et al., 2015); Iowa Gambling Task |  |  |  | (Lochman et al., 2019; McQuade & Breaux, 2017)^517,518^ | 2 (1%) |  |
|  |  |  |  |  |  |  |  |  |  |
|  | ECG | Heart Rate Variability | Sad Film, Stressful Interview |  |  |  | (Fiskum et al., 2019; McCraty et al., 1999)^159,546^ | 2 (1%) |  |
|  |  |  |  |  |  |  |  |  |  |
|  | EDA | Arousal Habituation | Conflict Discussion Task |  |  |  | (McKone et al., 2021)^547^ | 1 (0.5%) |  |
|  | **Non Specific Emotion Eliciting Tasks or Mixed Emotions Tasks** | | | | | | | |  |
|  | EEG | ERP | Go/No-Go Task (e.g. (Garavan et al., 1999)); International Affective Picture System (IAPS; (Lang, 2005); Cognitive Reappraisal Task; Chinese Affective Picture System (CAPS) with Reactivity and Regulation-Image Task (REAR-I); Distraction Task^543^; Emotional Pictures |  |  |  | (Connell et al., 2020; Deng et al., 2020; Deng et al., 2021; Deng & Zhang, 2020; Granic et al., 2012; Hum et al., 2013; Lamm et al., 2011; Latham et al., 2017; Lewis et al., 2008; Liu et al., 2022; Liu et al., 2019; Mehmood & Hyo, 2016; Stieben et al., 2007; Übel et al., 2015; van Cauwenberge et al., 2017; Yang et al., 2021; Zhang et al., 2013; Zhang et al., 2016)^114–116,131,508,519–524,536,537,543,548–551^ | 18 (9%) |  |
|  |  |  |  |  |  |  |  |  |  |
|  | ECG | RSA | Emotional Go-No-Go Task; Emotional Film Clips; Resting State; Baseline Vanilla | Cohen's κ > .90; Cronbach α = .80 - .87 |  |  | ^123–125,128,148,510,512,513,518,530,541,542,544,545^(Byrd et al., 2020; Debeuf et al., 2020; Fox et al., 2019; Gentzler et al., 2009; Guy et al., 2014; Katz & Gurtovenko, 2015; Kiser et al., 2019; Lisitsa et al., 2021; Lochman et al., 2019; Mezulis et al., 2015; Pang & Beauchaine, 2013; Tenenbaum et al., 2019; Vasilev et al., 2009; Zhang et al., 2017) | 14 (7%) |  |
|  |  |  |  |  |  |  |  |  |  |
|  | (f)MRI | Volume; Whole Brain; ROI | Images International Affective Picture Series; Cognitive Reappraisal Task (e.g. (Ochsner et al., 2004); Event-Related Facial Emotion Processing Task; Modified Emotion Label Task (Hariri et al., 2000; Lieberman et al., 2007); Mood-Repair Task; Emotional Go-No-Go Task; Emotional Pictures |  | Convergent validity: corr. volume, BOLD signal with ER in questionnaires (Pagliaccio et al., 2014) |  | (Belden et al., 2014; Colich et al., 2017; Joormann et al., 2012; Lin et al., 2024; Murphy et al., 2016; Pagliaccio et al., 2014; Poon et al., 2022; Tottenham et al., 2010; Urbain et al., 2019; Urbain et al., 2017)^112,113,135,136,138,514–516,535,552^ | 10 (5%) |  |
|  |  |  |  |  |  |  |  |  |  |
|  | ECG | Heart Rate Variability; Heart Rate | Resting State; International Affective Picture System (IAPS); Go–Nogo; Stroop Color-word^508^; Working Memory Task (Digit span; (Miyake, 2001) |  |  |  | (Constantin et al., 2022; Klinge et al., 2023; Taskiran et al., 2018)^117,118,127,502,530,538,539^ | 7 (3.5%) |  |
|  |  |  |  |  |  |  |  |  |  |
|  | MEG | Whole Brain | Emotional Go-No-Go Task; NimStim set of facial expressions (Tottenham et al., 2009); Karolinska Directed Emotional Faces – KDEF set |  |  |  | (Urbain et al., 2019; Urbain et al., 2017; Wessing et al., 2015) | 3 (1.5%) |  |
|  |  |  |  |  |  |  |  |  |  |
|  | EDA | Skin Conductance | International Affective Picture System (IAPS); Resting State |  |  |  | (Lochman et al., 2019; Taskiran et al., 2018)^139,518^ | 2 (1%) |  |
|  |  |  |  |  |  |  |  |  |  |
|  | Salvia Samples | Cortisol | Film Clips; Daily Measures |  |  |  | (Debeuf et al., 2020; Rnic et al., 2022)^155,530^ | 2 (1%) |  |
|  |  |  |  |  |  |  |  |  |  |
|  | ECG | Vagal Tone | Film Clips |  |  |  | (Eisenberg, Fabes, Karbon et al., 1996)^399^ | 1 (0.5%) |  |
|  |  |  |  |  |  |  |  |  |  |
|  | tDCS Stimulator | tDCS | Emotional Go-No-Go Task |  |  |  | (Estaji et al., 2024) | 1 (0.5%) |  |
|  |  |  |  |  |  |  |  |  |  |
|  | ECG | PEP | Resting State, Parent-Child Interaction |  |  |  | (Musser et al., 2018)^511^ | 1 (0.5%) |  |
|  |  |  |  |  |  |  |  |  |  |
|  | EMG | Startle Eye Blink | Emotional Pictures |  |  |  | (Latham et al., 2017)^550^ | 1 (0.5%) |  |
| **154 to 166 months** | **Frustration/Anger/Disappointment Eliciting Tasks** | | | | | | | |  |
|  | ECG | Heart Rate; Heart Rate Variability | Sad Film; Stressful Interview; Social Stress Test for children (TSST-C; (Buske-Kirschbaum, 1997) |  |  |  | (Fiol-Veny et al., 2019; Fiskum et al., 2019; McCraty et al., 1999)^158,159,546^ | 3 (1.5%) |  |
|  |  |  |  |  |  |  |  |  |  |
|  | ECG | RSA | Puzzle Task^399^; Stressor Paradigm (unsolvable anagrams) |  |  |  | (Perry et al., 2020; Wielgus et al., 2016)^110,540^ | 2 (1%) |  |
|  |  |  |  |  |  |  |  |  |  |
|  | EDA | Skin Conductance | Chatroom Interact Task with manipulated peer acceptance and rejection (Silk et al., 2012); Impossible Puzzle Task (e.g., (Somers et al., 2015) |  |  |  | (McQuade & Breaux, 2017)^546^ | 1 (0.5%) |  |
|  | **Non Specific Emotion Eliciting Tasks or Mixed Emotions Tasks** | | | | | | | |  |
|  | EEG | ERP | International Affective Picture System (IAPS; (Lang, 2005); Cognitive Reappraisal Task; Go/No-Go Task (e.g. (Garavan et al., 1999); Chinese Affective Picture System (CAPS) with Reactivity and Regulation-Image Task (REAR-I); Distraction Task^543^ |  |  |  | (Deng et al., 2021; Deng & Zhang, 2020; Mehmood & Hyo, 2016; Übel et al., 2015; van Cauwenberge et al., 2017; Zhang et al., 2013; Zhang et al., 2016) | 8 (4%) |  |
|  |  |  |  |  |  |  |  |  |  |
|  | (f)MRI | Volume; Whole Brain; ROI | Emotional Go-No-Go Task; Cognitive Reappraisal Task (e.g. Ochsner et al., 2004); Event-Related Facial Emotion Processing Task; Modified Emotion Label Task (Hariri et al., 2000; Lieberman et al., 2007); Mood-Repair Task; International Affective Picture System (IAPS); Emotional Face N-Back Task |  | Convergent validity: corr. volume, BOLD signal with ER in questionnaires (Pagliaccio et al., 2014) |  | (Colich et al., 2017; Fournier et al., 2021; Murphy et al., 2016; Pagliaccio et al., 2014; Poon et al., 2022; Tottenham et al., 2010; Urbain et al., 2017)^112,113,136,138,515,516,552,553^ | 8 (4%) |  |
|  |  |  |  |  |  |  |  |  |  |
|  | ECG | RSA | Emotional Go-No-Go Task; Emotional/Neutral Film Clips; Baseline Vanilla | Cohen's κ > .90; Cronbach α = .80 - .87 |  |  | (Debeuf et al., 2020; Eisenberg, Fabes, Karbon et al., 1996; Fox et al., 2019; Gentzler et al., 2009; Kiser et al., 2019; Lisitsa et al., 2021; Mezulis et al., 2015; Tenenbaum et al., 2019)^123,124,128,148,399,530,541,542^ | 8 (4%) |  |
|  |  |  |  |  |  |  |  |  |  |
|  | ECG | Heart Rate Variability | Resting State |  |  |  | (Fiskum et al., 2017; Fiskum et al., 2019; Klinge et al., 2023)^120,159,538^ | 3 (1.5%) |  |
|  |  |  |  |  |  |  |  |  |  |
|  | MEG | Whole Brain | Emotional Go-No-Go Task; NimStim set of facial expressions (Tottenham et al., 2009); Karolinska Directed Emotional Faces – KDEF set |  |  |  | (Urbain et al., 2017; Wessing et al., 2015) | 2 (1%) |  |
|  |  |  |  |  |  |  |  |  |  |
|  | Salvia Samples | Cortisol | Film Clips |  |  |  | (Debeuf et al., 2020)^530^ | 1 (0.5%) |  |
|  |  |  |  |  |  |  |  |  |  |
|  | ECG | Vagal Tone | Film Clips |  |  |  | (Eisenberg, Fabes, Karbon et al., 1996)^399^ | 1 (0.5%) |  |
| **167 to 179 months** | **Frustration/Anger/Disappointment Eliciting Tasks** | | | | | | | |  |
|  | ECG | RSA | Puzzle Task^399^; Stressor Paradigm (unsolvable anagrams) |  |  |  | (Perry et al., 2020; Wielgus et al., 2016)^110,540^ | 2 (1%) |  |
|  |  |  |  |  |  |  |  |  |  |
|  | ECG | Heart Rate Variability | Stressful Interview |  |  |  | (McCraty et al., 1999)^546^ | 1 (0.5%) |  |
|  | **Non Specific Emotion Eliciting Tasks or Mixed Emotions Tasks** | | | | | | | |  |
|  | EEG | ERP | Cognitive Reappraisal Task; Go-No-Go Task (e.g. (Garavan et al., 1999); Chinese Affective Picture System (CAPS) with Reactivity and Regulation-Image Task (REAR-I); International Affective Picture System (IAPS) |  |  |  | (Connell et al., 2020; Deng et al., 2021; Deng & Zhang, 2020; Mehmood & Hyo, 2016; van Cauwenberge et al., 2017; Zhang et al., 2016)^114,115,536,537,548,549^ | 6 (3%) |  |
|  |  |  |  |  |  |  |  |  |  |
|  | ECG | RSA | Film Clips; Baseline Vanilla | Cronbach α = .80 - .87 |  |  | (Debeuf et al., 2020; Fox et al., 2019; Kiser et al., 2019; Lisitsa et al., 2021; Mezulis et al., 2015; Wielgus et al., 2016)^128,148,530,540–542^ | 6 (3%) |  |
|  |  |  |  |  |  |  |  |  |  |
|  | MEG | Whole Brain | NimStim set of facial expressions (Tottenham et al., 2009); Karolinska Directed Emotional Faces – KDEF set |  |  |  | (Wessing et al., 2015) | 1 (0.5%) |  |
|  |  |  |  |  |  |  |  |  |  |
|  | Salvia Samples | Cortisol | Film Clips |  |  |  | (Debeuf et al., 2020)^530^ | 1 (0.5%) |  |
|  |  |  |  |  |  |  |  |  |  |
|  | (f)MRI | Volume; ROI | Emotional Go-No-Go Task; Emotion Processing Task |  | Convergent validity: corr. volume, BOLD signal with ER in questionnaires (Pagliaccio et al., 2014) |  | (Tottenham et al., 2010) | 1 (0.5%) |  |
| **180 to 192 months** | **Frustration/Anger/Disappointment Eliciting Tasks** | | | | | | | |  |
|  | ECG | RSA | Puzzle Task (Eisenberg, Fabes, Karbon et al., 1996); Tower task |  |  |  | (Dollar et al., 2020; Perry et al., 2020) | 2 (1%) |  |
|  | **Non Specific Emotion Eliciting Tasks or Mixed Emotions Tasks** | | | | | | | |  |
|  | EEG | ERP | Cognitive Reappraisal Task; Chinese Affective Picture System (CAPS) with Reactivity and Regulation-Image Task (REAR-I) |  |  |  | (Deng et al., 2021; Deng & Zhang, 2020; van Cauwenberge et al., 2017)^114,115,537^ | 3 (1.5%) |  |
|  |  |  |  |  |  |  |  |  |  |
|  | (f)MRI | Volume; ROI | Emotional Go-No-Go Task; Cognitive Reappraisal Task (e.g. (Ochsner et al., 2004) |  |  |  | (Murphy et al., 2016; Tottenham et al., 2010) | 2 (1%) |  |
|  |  |  |  |  |  |  |  |  |  |
|  | ECG | RSA | Baseline |  |  |  | (Lisitsa et al., 2021)^542^ | 1 (0.5%) |  |

# **References**

Abney, D. H., daSilva, E. B. & Bertenthal, B. I. (2021). Associations between infant-mother physiological synchrony and 4- and 6-month-old infants' emotion regulation. *Developmental psychobiology*, *63*(6), e22161. https://doi.org/10.1002/dev.22161

Abraham, E., Raz, G., Zagoory-Sharon, O. & Feldman, R. (2018). Empathy networks in the parental brain and their long-term effects on children's stress reactivity and behavior adaptation. *Neuropsychologia*, *116*(Pt A), 75–85. https://doi.org/10.1016/j.neuropsychologia.2017.04.015

Ainsworth, M. (Hrsg.). (1978). *Patterns of attachment: A psychological study of the strange situation*. Hillsdale, NJ: Erlbaum.

Alarcón, L. F. (2010). Impacto de las reglas de despliegue sobre la regulación emocional en una muestra de escolares. *Acta Colombiana de Psicología*(13(2), 79–90.

Alfano, C. A., Bower, J. L., Harvey, A. G., Beidel, D. C., Sharp, C. & Palmer, C. A. (2020). Sleep restriction alters children's positive emotional responses, but effects are moderated by anxiety. *Journal of child psychology and psychiatry, and allied disciplines*, *61*(10), 1150–1159. https://doi.org/10.1111/jcpp.13287

Anzman-Frasca, S., Paul, I. M., Moding, K. J., Savage, J. S., Hohman, E. E. & Birch, L. L. (2018). Effects of the INSIGHT Obesity Preventive Intervention on Reported and Observed Infant Temperament. *Journal of developmental and behavioral pediatrics : JDBP*, *39*(9), 736–743. https://doi.org/10.1097/DBP.0000000000000597

Arnold, H. S., Conture, E. G., Key, A. P. F. & Walden, T. (2011). Emotional reactivity, regulation and childhood stuttering: a behavioral and electrophysiological study. *Journal of communication disorders*, *44*(3), 276–293. https://doi.org/10.1016/j.jcomdis.2010.12.003

Atkinson, N. H., Jean, A. D. L. & Stack, D. M. (2021). Emotion regulation from infancy to toddlerhood: Individual and group trajectories of full-term and very-low-birthweight preterm infants. *Infancy : the official journal of the International Society on Infant Studies*, *26*(4), 570–595. https://doi.org/10.1111/infa.12405

August, E. G., Stack, D. M., Martin‐Storey, A., Serbin, L. A., Ledingham, J. & Schwartzman, A. E. (2017). Emotion Regulation in At‐Risk Preschoolers: Longitudinal Associations and Influences of Maternal Histories of Risk. *Infant and Child Development*, *26*(1), Artikel e1954. https://doi.org/10.1002/icd.1954

Aviles, A. I., Reisz, S., Jacobvitz, D. & Hazen, N. (2022). Maternal experiences of childhood psychological maltreatment: Relations with toddler emotional regulation. *Journal of Social and Personal Relationships*, *39*(7), 2022–2044. https://doi.org/10.1177/02654075221074382

Babkirk, S., Rios, V. & Dennis, T. A. (2015). The late positive potential predicts emotion regulation strategy use in school-aged children concurrently and two years later. *Developmental science*, *18*(5), 832–841. https://doi.org/10.1111/desc.12258

Bagne, A. (2021). *MEASURING INFANT EMOTION REGULATION WITHIN THE STILL FACE PROCEDURE: A NOVEL APPROACH TO ASSESSING REGULATION DEVELOPMENT IN THE CONTEXT OF PRENATAL MATERNAL STRESS* [Dissertation]. North Dakota State University.

Bailey, C. S., Denham, S. A., Curby, T. W. & Bassett, H. H [Hideko H.] (2016). Emotional and organizational supports for preschoolers' emotion regulation: Relations with school adjustment. *Emotion (Washington, D.C.)*, *16*(2), 263–279. https://doi.org/10.1037/a0039772

Bailey, C. S., Ondrusek, A. R., Curby, T. W. & Denham, S. A. (2022). Teachers' Consistency of Emotional Support Moderates the Association Between Young Children's Regulation Capacities and Their Preschool Adjustment. *Psychology in the schools*, *59*(6), 1051–1074. https://doi.org/10.1002/pits.22659

Baker, J. K., Fenning, R. M., Crnic, K. A [Keith A.], Baker, B. L [Bruce L.] & Blacher, J. (2007). Prediction of Social Skills in 6-Year-Old Children With and Without Developmental Delays: Contributions of Early Regulation and Maternal Scaffolding. *American Journal on Mental Retardation*, *112*(5), 375. https://doi.org/10.1352/0895-8017(2007)112[0375:POSSIY]2.0.CO;2

Baker, J. K., Fenning, R. M., Erath, S. A. & Fabian, S. (2022). Parasympathetic withdrawal indexes risk for emotion dysregulation in children with autism spectrum disorder. *Autism research : official journal of the International Society for Autism Research*, *15*(11), 2064–2068. https://doi.org/10.1002/aur.2814

Baker, J. K., Fenning, R. M. & Moffitt, J. (2019). A Cross-Sectional Examination of the Internalization of Emotion Co-regulatory Support in Children with ASD. *Journal of autism and developmental disorders*, *49*(10), 4332–4338. https://doi.org/10.1007/s10803-019-04091-0

Barbaro, K. de, Clackson, K. & Wass, S. (2016). Stress reactivity speeds basic encoding processes in infants. *Developmental psychobiology*, *58*(5), 546–555. https://doi.org/10.1002/dev.21399

Bayley, N. (1969). Manual for the Bayley scales of infant development.

Bayley, N. (Hrsg.). (1993). *Bayley Scales of Infant Development* (Second Edition). San Antonio, TX: Psychological Corporation.

Beck, K. B., Conner, C. M., Breitenfeldt, K. E., Northrup, J. B., White, S. W. & Mazefsky, C. A. (2020). Assessment and Treatment of Emotion Regulation Impairment in Autism Spectrum Disorder Across the Life Span: Current State of the Science and Future Directions. *Child and adolescent psychiatric clinics of North America*, *29*(3), 527–542. https://doi.org/10.1016/j.chc.2020.02.003

Behrendt, H. F., Konrad, K., Perdue, K. L. & Firk, C. (2020). Infant brain responses to live face-to-face interaction with their mothers: Combining functional near-infrared spectroscopy (fNIRS) with a modified still-face paradigm. *Infant behavior & development*, *58*, 101410. https://doi.org/10.1016/j.infbeh.2019.101410

Belden, A. C [Andy C.], Luby, J. L., Pagliaccio, D. & Barch, D. M. (2014). Neural activation associated with the cognitive emotion regulation of sadness in healthy children. *Developmental cognitive neuroscience*, *9*, 136–147. https://doi.org/10.1016/j.dcn.2014.02.003

Bendezú, J. J., Cole, P. M., Tan, P. Z., Armstrong, L. M., Reitz, E. B. & Wolf, R. M. (2018). Child language and parenting antecedents and externalizing outcomes of emotion regulation pathways across early childhood: A person-centered approach. *Development and psychopathology*, *30*(4), 1253–1268. https://doi.org/10.1017/S0954579417001675

Benga, O., Susa-Erdogan, G., Friedlmeier, W [Wolfgang], Corapci, F. & Romonti, M. (2018). Maternal Self-Construal, Maternal Socialization of Emotions and Child Emotion Regulation in a Sample of Romanian Mother-Toddler Dyads. *Frontiers in psychology*, *9*, 2680. https://doi.org/10.3389/fpsyg.2018.02680

Berry, D., Palmer, A. R., Distefano, R. & Masten, A. S. (2019). Autonomic complexity and emotion (dys-)regulation in early childhood across high- and low-risk contexts. *Development and psychopathology*, *31*(3), 1173–1190. https://doi.org/10.1017/S0954579419000683

Bettis, A. H., Henry, L., Prussien, K. V., Vreeland, A., Smith, M [Michele], Adery, L. H. & Compas, B. E. (2019). Laboratory and Self-Report Methods to Assess Reappraisal and Distraction in Youth. *Journal of clinical child and adolescent psychology : the official journal for the Society of Clinical Child and Adolescent Psychology, American Psychological Association, Division 53*, *48*(6), 855–865. https://doi.org/10.1080/15374416.2018.1466306

Binion, G. & Zalewski, M. (2018). Maternal emotion dysregulation and the functional organization of preschoolers' emotional expressions and regulatory behaviors. *Emotion (Washington, D.C.)*, *18*(3), 386–399. https://doi.org/10.1037/emo0000319

Black, M. M., Hutcheson, J. J., Dubowitz, H., Starr, R. H. & Berenson-Howard, J. (1996). The roots of competence: Mother-child interaction among low income, urban, African American families. *Journal of Applied Developmental Psychology*, *17*(3), 367–391. https://doi.org/10.1016/S0193-3973(96)90032-5

Blair, C., Calkins, S. & Kopp, L. (2010). Self‐Regulation as the Interface of Emotional and Cognitive Development. In R. H. Hoyle (Hrsg.), *Handbook of Personality and Self‐Regulation* (S. 64–90). Wiley. https://doi.org/10.1002/9781444318111.ch4

Blandon, A. Y., Calkins, S. D. & Keane, S. P [Susan P.] (2010). Predicting emotional and social competence during early childhood from toddler risk and maternal behavior. *Development and psychopathology*, *22*(1), 119–132. https://doi.org/10.1017/S0954579409990307

Blandon, A. Y., Calkins, S. D., Keane, S. P [Susan P.] & O'Brien, M. (2008). Individual differences in trajectories of emotion regulation processes: the effects of maternal depressive symptomatology and children's physiological regulation. *Developmental psychology*, *44*(4), 1110–1123. https://doi.org/10.1037/0012-1649.44.4.1110

Blankson, A. N., Weaver, J. M., Leerkes, E. M., O'Brien, M., Calkins, S. D. & Marcovitch, S. (2017). Cognitive and Emotional Processes as Predictors of a Successful Transition into School. *Early education and development*, *28*(1), 1–20. https://doi.org/10.1080/10409289.2016.1183434

Block, J. H. & Block, J. (1980). The role of ego-control and ego-resiliency in the organization of behavior. In W. A. Collins (Hrsg.), *Development of cognition, affect and social relations: The Minnesota symposia on child psychology* (S. 39–101). Hillsdale, NJ: Erlbaum.

Bocknek, E. L., Richardson, P. A., McGoron, L., Raveau, H. & Iruka, I. U. (2020). Adaptive Parenting Among Low-Income Black Mothers and Toddlers' Regulation of Distress. *Child development*, *91*(6), 2178–2191. https://doi.org/10.1111/cdev.13461

Bocknek, E. L., Richardson, P. A., van den Heuvel, M. I., Qipo, T. & Brophy-Herb, H. E. (2018). Sleep moderates the association between routines and emotion regulation for toddlers in poverty. *Journal of family psychology : JFP : journal of the Division of Family Psychology of the American Psychological Association (Division 43)*, *32*(7), 966–974. https://doi.org/10.1037/fam0000433

Boldt, L. J., Goffin, K. C. & Kochanska, G. (2020). The significance of early parent-child attachment for emerging regulation: A longitudinal investigation of processes and mechanisms from toddler age to preadolescence. *Developmental psychology*, *56*(3), 431–443. https://doi.org/10.1037/dev0000862

Bolten, M., Nast, I., Skrundz, M., Stadler, C., Hellhammer, D. H. & Meinlschmidt, G. (2013). Prenatal programming of emotion regulation: neonatal reactivity as a differential susceptibility factor moderating the outcome of prenatal cortisol levels. *Journal of psychosomatic research*, *75*(4), 351–357. https://doi.org/10.1016/j.jpsychores.2013.04.014

Boyd-Soisson, E. F. (2002). Parents' socialization of children's emotions and children's socioemotional adjustment: The role of adult attachment. *The University of Texas at Austin.*

Bozicevic, Pascalis, L. de [L.], Montirosso, R [R.], Ferrari, P. F., Giusti, L., Cooper, P. J [P. J.] & Murray, L [L.] (2021). Sculpting Culture: Early Maternal Responsiveness and Child Emotion Regulation – A UK-Italy Comparison. *Journal of Cross-Cultural Psychology*, *52*(1), 22–42. https://doi.org/10.1177/0022022120971353

Bozicevic, L., Pascalis, L. de [Leonardo], Schuitmaker, N., Tomlinson, M., Cooper, P. J [Peter J.] & Murray, L [Lynne] (2016). Longitudinal Association between Child Emotion Regulation and Aggression, and the Role of Parenting: A Comparison of Three Cultures. *Psychopathology*, *49*(4), 228–235. https://doi.org/10.1159/000447747

Bradley, M. M. & Lang, P. J. (1994). Measuring emotion: the Self-Assessment Manikin and the Semantic Differential. *Journal of behavior therapy and experimental psychiatry*, *25*(1), 49–59. https://doi.org/10.1016/0005-7916(94)90063-9

Braungart-Rieker, J. M., Garwood, M. M., Powers, B. P. & Wang, X. (2001). Parental sensitivity, infant affect, and affect regulation: predictors of later attachment. *Child development*, *72*(1), 252–270. https://doi.org/10.1111/1467-8624.00277

Braungart-Rieker, J., Garwood, M. M., Powers, B. P. & Notaro, P. C. (1998). Infant affect and affect regulation during the still-face paradigm with mothers and fathers: the role of infant characteristics and parental sensitivity. *Developmental psychology*, *34*(6), 1428–1437. https://doi.org/10.1037/0012-1649.34.6.1428

Braungart-Rieker. & Stifter, C. A. (1996). Infants' Responses to Frustrating Situations: Continuity and Change in Reactivity and Regulation. *Child development*, *67*(4), 1767–1779. https://doi.org/10.1111/j.1467-8624.1996.tb01826.x

Breaux, R. P., McQuade, J. D., Harvey, E. A. & Zakarian, R. J. (2018). Longitudinal Associations of Parental Emotion Socialization and Children's Emotion Regulation: The Moderating Role of ADHD Symptomatology. *Journal of abnormal child psychology*, *46*(4), 671–683. https://doi.org/10.1007/s10802-017-0327-0

Bridges, L. J., Grolnick, W. S. & Connell, J. P. (1997). Infant emotion regulation with mothers and fathers. *Infant Behavior and Development*, *20*(1), 47–57. https://doi.org/10.1016/S0163-6383(97)90060-6

Bridgett, D. J., Burt, N. M., Edwards, E. S. & Deater-Deckard, K [Kirby] (2015). Intergenerational Transmission of Self-Regulation: A Multidisciplinary Review and Integrative Conceptual Framework. *Psychological bulletin*, *141*(3), 602–654. https://doi.org/10.1037/a0038662

Bunford, N., Evans, S. W. & Wymbs, F. (2015). ADHD and Emotion Dysregulation Among Children and Adolescents. *Clinical child and family psychology review*, *18*(3), 185–217. https://doi.org/10.1007/s10567-015-0187-5

Bunford, N., Evans, S. W., Zoccola, P. M., Owens, J. S., Flory, K. & Spiel, C. F. (2017). Correspondence between Heart Rate Variability and Emotion Dysregulation in Children, Including Children with ADHD. *Journal of abnormal child psychology*, *45*(7), 1325–1337. https://doi.org/10.1007/s10802-016-0257-2

Buske-Kirschbaum, A. (1997). Attenuated free cortisol response to psychosocial stress in children with atopic dermatitis. *Psychosomatic medicine*(59(4), 419–426.

Buss, K. A. & Goldsmith, H. H. (1998). Fear and Anger Regulation in Infancy: Effects on the Temporal Dynamics of Affective Expression. *Child development*, *69*(2), 359. https://doi.org/10.2307/1132171

Byrd, A. L., Lee, A. H., Frigoletto, O. A., Zalewski, M. & Stepp, S. D. (2021). Applying new RDoC dimensions to the development of emotion regulation: Examining the influence of maternal emotion regulation on within-individual change in child emotion regulation. *Development and psychopathology*, *33*(5), 1821–1836. https://doi.org/10.1017/S0954579421000948

Byrd, A. L., Vine, V., Beeney, J. E., Scott, L. N., Jennings, J. R. & Stepp, S. D. (2020). RSA reactivity to parent-child conflict as a predictor of dysregulated emotion and behavior in daily life. *Psychological medicine*, 1–9. https://doi.org/10.1017/S0033291720002810

Cabrera, N. J [Natasha J.], Karberg, E [Elizabeth], Malin, J. L [Jenessa L.] & Aldoney, D [Daniela] (2017). THE MAGIC OF PLAY: LOW-INCOME MOTHERS' AND FATHERS' PLAYFULNESS AND CHILDREN'S EMOTION REGULATION AND VOCABULARY SKILLS. *Infant mental health journal*, *38*(6), 757–771. https://doi.org/10.1002/imhj.21682

Calkins (1994). Origins and Outcomes of Individual Differences in Emotion Regulation. *Monographs of the Society for Research in Child Development*, *59*(2/3), 53. https://doi.org/10.2307/1166138

Calkins (1997). Cardiac vagal tone indices of temperamental reactivity and behavioral regulation in young children. *Developmental psychobiology*, *31*(2), 125–135. https://doi.org/10.1002/(SICI)1098-2302(199709)31:2%3C125::AID-DEV5%3E3.0.CO;2-M

Calkins, Dedmon, S. E [Susan E.], Gill, K. L., Lomax, L. E. & Johnson, L. M. (2002). Frustration in Infancy: Implications for Emotion Regulation, Physiological Processes, and Temperament. *Infancy : the official journal of the International Society on Infant Studies*, *3*(2), 175–197. https://doi.org/10.1207/S15327078IN0302_4

Calkins, Gill, K. L., Johnson, M. C. & Smith, C. L. (1999). Emotional Reactivity and Emotional Regulation Strategies as Predictors of Social Behavior with Peers During Toddlerhood. *Social Development*, *8*(3), 310–334. https://doi.org/10.1111/1467-9507.00098

Calkins & Hill, A. (2007). Caregiver influence on emerging emotion regulation: biological and environmental transactions in early development. In Gross (Hrsg.), *Handbook of Emotion Regulation* (S. 229–248). New York, NY: Guilford Press.

Calkins & Howse, R. B. (2004). Individual differences self-regulation: Implications for childhood adjustment. In Philippot & R. S. Feldman (Hrsg.), *The regulation of emotion* (S. 307–332). Lawrence Erlbaum Associates Publishers.

Calkins & Johnson (1998). Toddler regulation of distress to frustrating events: temperamental and maternal correlates. *Infant Behavior and Development*, *21*(3), 379–395. https://doi.org/10.1016/S0163-6383(98)90015-7

Calkins & Keane, S. P [Susan P.] (2004). Cardiac vagal regulation across the preschool period: stability, continuity, and implications for childhood adjustment. *Developmental psychobiology*, *45*(3), 101–112. https://doi.org/10.1002/dev.20020

Calkins, Smith, C. L., Gill, K. L. & Johnson, M. C. (1998). Maternal Interactive Style Across Contexts: Relations to Emotional, Behavioral and Physiological Regulation During Toddlerhood. *Social Development*, *7*(3), 350–369. https://doi.org/10.1111/1467-9507.00072

Calkins, S. D [S. D.] & Dedmon, S. E [S. E.] (2000). Physiological and behavioral regulation in two-year-old children with aggressive/destructive behavior problems. *Journal of abnormal child psychology*, *28*(2), 103–118. https://doi.org/10.1023/A:1005112912906

Campos, J. J., Frankel, C. B. & Camras, L. (2004). On the nature of emotion regulation. *Child development*, *75*(2), 377–394. https://doi.org/10.1111/j.1467-8624.2004.00681.x

Caplan, B. & Baker, B. L [B. L.] (2017). Maternal control and early child dysregulation: Moderating roles of ethnicity and child delay status. *Journal of intellectual disability research : JIDR*, *61*(2), 115–129. https://doi.org/10.1111/jir.12280

Carlson, S. M. & Wang, T. S. (2007). Inhibitory control and emotion regulation in preschool children. *Cognitive Development*, *22*(4), 489–510. https://doi.org/10.1016/j.cogdev.2007.08.002

Carter, A. S., Little, C., Briggs-Gowan, M. J. & Kogan, N. (1999). The infant-toddler social and emotional assessment (ITSEA): Comparing parent ratings to laboratory observations of task mastery, emotion regulation, coping behaviors, and attachment status. *Infant mental health journal*, *20*(4), 375–392. https://doi.org/10.1002/(SICI)1097-0355(199924)20:4<375::AID-IMHJ2>3.0.CO;2-P

Cassidy, J. (1994). Emotion Regulation: Influences of Attachment Relationships. *Monographs of the Society for Research in Child Development*, *59*(2/3), 228. https://doi.org/10.2307/1166148

Castro, V. L., Halberstadt, A. G., Lozada, F. T. & Craig, A. B. (2015). Parents' Emotion-Related Beliefs, Behaviors, and Skills Predict Children's Recognition of Emotion. *Infant and Child Development*, *24*(1), 1–22. https://doi.org/10.1002/icd.1868

Chan, N. & Neece, C. L. (2018). Parenting Stress and Emotion Dysregulation among Children with Developmental Delays: The Role of Parenting Behaviors. *Journal of Child and Family Studies*, *27*(12), 4071–4082. https://doi.org/10.1007/s10826-018-1219-9

Chang, H., Shelleby, E. C., Cheong, J. & Shaw, D. S [Daniel S.] (2012). Cumulative Risk, Negative Emotionality, and Emotion Regulation as Predictors of Social Competence in Transition to School: A Mediated Moderation Model. *Social Development*, *21*(4), 780–800. https://doi.org/10.1111/j.1467-9507.2011.00648.x

Chavez Arana, C., Catroppa, C., Yáñez-Téllez, G., Prieto-Corona, B., León, M. A. de, García, A., Gómez-Raygoza, R., Hearps, S. J. C. & Anderson, V. (2020). A Parenting Program to Reduce Disruptive Behavior in Hispanic Children with Acquired Brain Injury: A Randomized Controlled Trial Conducted in Mexico. *Developmental neurorehabilitation*, *23*(4), 218–230. https://doi.org/10.1080/17518423.2019.1645224

Cho, S., Philbrook, L. E., Davis, E. L. & Buss, K. A. (2017). Sleep duration and RSA suppression as predictors of internalizing and externalizing behaviors. *Developmental psychobiology*, *59*(1), 60–69. https://doi.org/10.1002/dev.21467

Cibralic, S., Kohlhoff, J., Wallace, N., McMahon, C. & Eapen, V. (2024). Emotional Regulation and Language in Young Children With and Without Autism Traits. *Journal of Early Intervention*, *46*(3), 428–447. https://doi.org/10.1177/10538151231176188

Cicchetti. (1991). Contributions from the study of high-risk populations to understanding the development of emotion regulation. In J. Garber & K. Dodge (Hrsg.), *The development of emotion regulation and dysregulation* (S. 15–48). Cambridge: Cambridge University Press.

Cicchetti, Ackerman, B. P. & Izard, C. E [Carroll E.] (1995). Emotions and emotion regulation in developmental psychopathology. *Development and psychopathology*, *7*(1), 1–10. https://doi.org/10.1017/S0954579400006301

Cimino, S. & Cerniglia, L. (2018). A Longitudinal Study for the Empirical Validation of an Etiopathogenetic Model of Internet Addiction in Adolescence Based on Early Emotion Regulation. *BioMed research international*, *2018*, 4038541. https://doi.org/10.1155/2018/4038541

Clark, C. A. C., Skowron, E. A., Giuliano, R. J. & Fisher, P. A. (2016). Intersections between cardiac physiology, emotion regulation and interpersonal warmth in preschoolers: Implications for drug abuse prevention from translational neuroscience. *Drug and alcohol dependence*, *163 Suppl 1*(Suppl 1), S60-9. https://doi.org/10.1016/j.drugalcdep.2016.01.033

Cohen & D'Esposito, M. (2016). The Segregation and Integration of Distinct Brain Networks and Their Relationship to Cognition. *The Journal of neuroscience : the official journal of the Society for Neuroscience*, *36*(48), 12083–12094. https://doi.org/10.1523/JNEUROSCI.2965-15.2016

Cole (1986). Children's Spontaneous Control of Facial Expression. *Child development*, *57*(6), 1309. https://doi.org/10.2307/1130411

Cole. (1990). *Mood induction stimulus for children*. Bethesda, MD: National Institute of Mental Health.

Cole, P. M., Dennis, T. A., Smith‐Simon, K. E. & Cohen, L. H. (2009). Preschoolers' Emotion Regulation Strategy Understanding: Relations with Emotion Socialization and Child Self‐regulation. *Social Development*, *18*(2), 324–352. https://doi.org/10.1111/j.1467-9507.2008.00503.x

Cole, P. M., Ledonne, E. N. & Tan, P. Z. (2013). A Longitudinal Examination of Maternal Emotions in Relation to Young Children's Developing Self-Regulation. *Parenting*, *13*(2), 113–132. https://doi.org/10.1080/15295192.2012.709152

Cole, P. M., Martin, S. E. & Dennis, T. A. (2004). Emotion regulation as a scientific construct: methodological challenges and directions for child development research. *Child development*, *75*(2), 317–333. https://doi.org/10.1111/j.1467-8624.2004.00673.x

Cole, P. M., Zahn-Waxler, C. & Smith, K. D. (1994). Expressive control during a disappointment: Variations related to preschoolers' behavior problems. *Developmental psychology*, *30*(6), 835–846. https://doi.org/10.1037/0012-1649.30.6.835

Colich, N. L., Williams, E. S., Ho, T. C., King, L. S., Humphreys, K. L., Price, A. N., Ordaz, S. J. & Gotlib, I. H. (2017). The association between early life stress and prefrontal cortex activation during implicit emotion regulation is moderated by sex in early adolescence. *Development and psychopathology*, *29*(5), 1851–1864. https://doi.org/10.1017/S0954579417001444

Connell, A., Danzo, S., Magee, K. & Dawson, G [Glen] (2020). Rumination in Early Adolescent Girls: An EEG Study of Cognitive Control and Emotional Responding in an Emotional Go/NoGo Task. *Cognitive, affective & behavioral neuroscience*, *20*(1), 181–194. https://doi.org/10.3758/s13415-019-00761-9

Connor-Smith, J. K., Compas, B. E., Wadsworth, M. E., Thomsen, A. H. & Saltzman, H. (2000). Responses to stress in adolescence: Measurement of coping and involuntary stress responses. *Journal of consulting and clinical psychology*, *68*(6), 976–992. https://doi.org/10.1037/0022-006X.68.6.976

Conradt, E. & Ablow, J. (2010). Infant physiological response to the still-face paradigm: contributions of maternal sensitivity and infants' early regulatory behavior. *Infant behavior & development*, *33*(3), 251–265. https://doi.org/10.1016/j.infbeh.2010.01.001

Constantin, K. L., Moline, R. L., Pillai Riddell, R., Spence, J. R., Fiacconi, C. M., Lupo-Flewelling, K. & McMurtry, C. M. (2022). Parent and child self- and co-regulation during pediatric venipuncture: Exploring heart rate variability and the effects of a mindfulness intervention. *Developmental psychobiology*, *64*(5), e22277. https://doi.org/10.1002/dev.22277

Costa, A. P., Steffgen, G. & Vögele, C. (2019). The role of alexithymia in parent-child interaction and in the emotional ability of children with autism spectrum disorder. *Autism research : official journal of the International Society for Autism Research*, *12*(3), 458–468. https://doi.org/10.1002/aur.2061

Dadds, M. R., Gale, N., Godbee, M., Moul, C., Pasalich, D. S., Fink, E. & Hawes, D. J. (2016). Expression and Regulation of Attachment-Related Emotions in Children with Conduct Problems and Callous-Unemotional Traits. *Child psychiatry and human development*, *47*(4), 647–656. https://doi.org/10.1007/s10578-015-0598-z

Davies, P. T., Manning, L. G. & Cicchetti, D [Dante] (2013). Tracing the cascade of children's insecurity in the interparental relationship: the role of stage-salient tasks. *Child development*, *84*(1), 297–312. https://doi.org/10.1111/j.1467-8624.2012.01844.x

Davis, E. L., Quiñones-Camacho, L. E. & Buss, K. A. (2016). The effects of distraction and reappraisal on children's parasympathetic regulation of sadness and fear. *Journal of experimental child psychology*, *142*, 344–358. https://doi.org/10.1016/j.jecp.2015.09.020

Davis, M., Thomassin, K., Bilms, J., Suveg, C., Shaffer, A. & Beach, S. R. H. (2017). Preschoolers' genetic, physiological, and behavioral sensitivity factors moderate links between parenting stress and child internalizing, externalizing, and sleep problems. *Developmental psychobiology*, *59*(4), 473–485. https://doi.org/10.1002/dev.21510

Dawson, G [G.], Panagiotides, H., Klinger, L. G. & Hill, D. (1992). The role of frontal lobe functioning in the development of infant self-regulatory behavior. *Brain and cognition*, *20*(1), 152–175. https://doi.org/10.1016/0278-2626(92)90066-U

Day, K. L. & Smith, C. L. (2013). Understanding the role of private speech in children's emotion regulation. *Early childhood research quarterly*, *28*(2), 405–414. https://doi.org/10.1016/j.ecresq.2012.10.003

Day., Mazefsky, C. A. & Wetherby, A. M. (2022). Characterizing difficulties with emotion regulation in toddlers with autism spectrum disorder. *Research in autism spectrum disorders*, *96.* https://doi.org/10.1016/j.rasd.2022.101992

Deater-Deckard, K [K.] (1997). Parent-Child Interaction System (PARCHISY) Institute of Psychiatry, *London, UK*.

Debeuf, T., Verbeken, S., Boelens, E., Volkaert, B., van Malderen, E., Michels, N. & Braet, C. (2020). Emotion regulation training in the treatment of obesity in young adolescents: protocol for a randomized controlled trial. *Trials*, *21*(1), 153. https://doi.org/10.1186/s13063-019-4020-1

DeCicco, J. M., O'Toole, L. J. & Dennis, T. A. (2014). The late positive potential as a neural signature for cognitive reappraisal in children. *Developmental neuropsychology*, *39*(7), 497–515. https://doi.org/10.1080/87565641.2014.959171

DeCicco, J. M., Solomon, B. & Dennis, T. A. (2012). Neural correlates of cognitive reappraisal in children: an ERP study. *Developmental cognitive neuroscience*, *2*(1), 70–80. https://doi.org/10.1016/j.dcn.2011.05.009

Delgado, M. R., Miller, M. M., Inati, S. & Phelps, E. A. (2005). An fMRI study of reward-related probability learning. *NeuroImage*, *24*(3), 862–873. https://doi.org/10.1016/j.neuroimage.2004.10.002

Deng, X., Gao, Q., Zhang, L. & Li, Y [Yanzhen] (2020). Neural Underpinnings of the Role of Trait Mindfulness in Emotion Regulation in Adolescents. *Mindfulness*, *11*(5), 1120–1130. https://doi.org/10.1007/s12671-019-01276-7

Deng, X., Sang, B., Ku, Y. & Sai, L. (2019). Age-Related Differences in the Late Positive Potential during Emotion Regulation between Adolescents and Adults. *Scientific reports*, *9.* https://doi.org/10.1038/s41598-019-42139-4

Deng, X., Yang, M. & An, S. (2021). Differences in frontal EEG asymmetry during emotion regulation between high and low mindfulness adolescents. *Biological psychology*, *158*, 107990. https://doi.org/10.1016/j.biopsycho.2020.107990

Deng, X. & Zhang, L. (2020). Neural underpinnings of the relationships between sensation seeking and emotion regulation in adolescents. *International journal of psychology : Journal international de psychologie*, *55*(5), 851–860. https://doi.org/10.1002/ijop.12649

Denham, Bassett, H. H [H. H.] & Wyatt, T. (2007). The Socialization of Emotional Competence. In J. E. Grusec & P. D. Hastings (Hrsg.), *Handbook of Socialization: Theory and Research* (S. 614–637). Guilford Press.

Dennis, T. A., Cole, P. M., Wiggins, C. N., Cohen, L. H. & Zalewski, M. (2009). The functional organization of preschool-age children's emotion expressions and actions in challenging situations. *Emotion (Washington, D.C.)*, *9*(4), 520–530. https://doi.org/10.1037/a0016514

Dennis, T. A. & Hajcak, G. (2009). The late positive potential: a neurophysiological marker for emotion regulation in children. *Journal of child psychology and psychiatry, and allied disciplines*, *50*(11), 1373–1383. https://doi.org/10.1111/j.1469-7610.2009.02168.x

Dennis, T. A., Hong, M. & Solomon, B. (2010). Do the associations between exuberance and emotion regulation depend on effortful control? *International Journal of Behavioral Development*, *34*(5), 462–472. https://doi.org/10.1177/0165025409355514

Dennis, T. A., Malone, M. M. & Chen, C.‑C. (2009). Emotional face processing and emotion regulation in children: an ERP study. *Developmental neuropsychology*, *34*(1), 85–102. https://doi.org/10.1080/87565640802564887

DiCorcia, J. A., Snidman, N., Sravish, A. V. & Tronick, E [Ed] (2016). Evaluating the Nature of the Still‐Face Effect in the Double Face‐to‐Face Still‐Face Paradigm Using Different Comparison Groups. *Infancy : the official journal of the International Society on Infant Studies*, *21*(3), 332–352. https://doi.org/10.1111/infa.12123

Diener, M. L., Mangelsdorf, S. C., McHale, J. L. & Frosch, C. A. (2002). Infants' Behavioral Strategies for Emotion Regulation With Fathers and Mothers: Associations With Emotional Expressions and Attachment Quality. *Infancy : the official journal of the International Society on Infant Studies*, *3*(2), 153–174. https://doi.org/10.1207/S15327078IN0302_3

Ding, N., Fu, L., Qian, L., Sun, B., Li, C., Gao, H [Huiyun], Lei, T. & Ke, X. (2024). The correlation between brain structure characteristics and emotion regulation ability in children at high risk of autism spectrum disorder. *European child & adolescent psychiatry*, *33*(9), 3247–3262. https://doi.org/10.1007/s00787-024-02369-y

Distefano, R., Grenell, A., Palmer, A. R., Houlihan, K., Masten, A. S. & Carlson, S. M. (2021). Self-regulation as promotive for academic achievement in young children across risk contexts. *Cognitive Development*, *58.* https://doi.org/10.1016/j.cogdev.2021.101050

Dollar, J. M., Calkins, S. D., Berry, N. T., Perry, N. B., Keane, S. P [Susan P.], Shanahan, L. & Wideman, L. (2020). Developmental patterns of respiratory sinus arrhythmia from toddlerhood to adolescence. *Developmental psychology*, *56*(4), 783–794. https://doi.org/10.1037/dev0000894

Dougherty, L. R., Blankenship, S. L., Spechler, P. A., Padmala, S. & Pessoa, L. (2015). An fMRI Pilot Study of Cognitive Reappraisal in Children: Divergent Effects on Brain and Behavior. *Journal of Psychopathology and Behavioral Assessment*, *37*(4), 634–644. https://doi.org/10.1007/s10862-015-9492-z

Dunbar, A. S., Lozada, F. T., Ahn, L. H. & Leerkes, E. M. (2022). Mothers' preparation for bias and responses to children's distress predict positive adjustment among Black children: an attachment perspective. *Attachment & human development*, *24*(3), 287–303. https://doi.org/10.1080/14616734.2021.1976922

Eiden, R. D., McAuliffe, S., Kachadourian, L., Coles, C., Colder, C. & Schuetze, P. (2009). Effects of prenatal cocaine exposure on infant reactivity and regulation. *Neurotoxicology and teratology*, *31*(1), 60–68. https://doi.org/10.1016/j.ntt.2008.08.005

Eisenberg, Cumberland, A. & Spinrad, T. L [Tracy L.] (1998). Parental Socialization of Emotion. *Psychological Inquiry*, *9*(4), 241–273. https://doi.org/10.1207/s15327965pli0904_1

Eisenberg, Fabes, R. A [Richard A.], Guthrie, I. K [Ivanna K.], Murphy, B. C [Bridget C.], Maszk, P., Holmgren, R. & Suh, K. (1996). The relations of regulation and emotionality to problem behavior in elementary school children. *Development and psychopathology*, *8*(1), 141–162. https://doi.org/10.1017/S095457940000701X

Eisenberg, Fabes, R. A [Richard A.], Guthrie, I. K [Ivanna K.] & Reiser, M [Mark] (2000). Dispositional emotionality and regulation: Their role in predicting quality of social functioning. *Journal of personality and social psychology*, *78*(1), 136–157. https://doi.org/10.1037/0022-3514.78.1.136

Eisenberg, Fabes, R. A [Richard A.], Karbon, M., Murphy, B. C [Bridget C.], Wosinski, M., Polazzi, L., Carlo, G. & Juhnke, C. (1996). The Relations of Children's Dispositional Prosocial Behavior to Emotionality, Regulation, and Social Functioning. *Child development*, *67*(3), 974. https://doi.org/10.2307/1131874

Eisenberg, Hofer, C. & Vaughan, J. (2007). Effortful Control and Its Socioemotional Consequences. In Gross (Hrsg.), *Handbook of Emotion Regulation* (S. 287–306). New York, NY: Guilford Press.

Eisenberg & Morris, A. S. (2003). Children’s Emotion-Related Regulation. In *Advances in Child Development and Behavior* (Bd. 30, S. 189–229). Elsevier. https://doi.org/10.1016/S0065-2407(02)80042-8

Eisenberg & Spinrad, T. L [Tracy L.] (2004). Emotion-related regulation: sharpening the definition. *Child development*, *75*(2), 334–339. https://doi.org/10.1111/j.1467-8624.2004.00674.x

Eisenberg, N [N.], Guthrie, I. K [I. K.], Fabes, R. A [R. A.], Shepard, S., Losoya, S [S.], Murphy, B. C [B. C.], Jones, S., Poulin, R. & Reiser, M [M.] (2000). Prediction of elementary school children's externalizing problem behaviors from attentional and behavioral regulation and negative emotionality. *Child development*, *71*(5), 1367–1382. https://doi.org/10.1111/1467-8624.00233

Eisenberg, N [Nancy], Valiente, C., Morris, A. S., Fabes, R. A [Richard A.], Cumberland, A., Reiser, M [Mark], Gershoff, E. T., Shepard, S. A. & Losoya, S [Sandra] (2003). Longitudinal relations among parental emotional expressivity, children's regulation, and quality of socioemotional functioning. *Developmental psychology*, *39*(1), 3–19. https://doi.org/10.1037/0012-1649.39.1.3

Ekas, N. V., Lickenbrock, D. M. & Braungart-Rieker, J. M [Julia M.] (2013). Developmental Trajectories of Emotion Regulation Across Infancy: Do Age and the Social Partner Influence Temporal Patterns? *Infancy : the official journal of the International Society on Infant Studies*, *18*(5). https://doi.org/10.1111/infa.12003

Ekman, P. (Hrsg.). (1994). *Series in affective science*. *The nature of emotion: Fundamental questions*. Oxford Univ. Press. http://www.loc.gov/catdir/enhancements/fy0603/94018638-d.html

El-Sheikh (2001). Parental drinking problems and children's adjustment: vagal regulation and emotional reactivity as pathways and moderators of risk. *Journal of Abnormal Psychology*, *110*(4), 499–515. https://doi.org/10.1037/0021-843X.110.4.499

Endriga, M. C., Jordan, J. R. & Speltz, M. L. (2003). Emotion self-regulation in preschool-aged children with and without orofacial clefts. *Journal of developmental and behavioral pediatrics : JDBP*, *24*(5), 336–344. https://doi.org/10.1097/00004703-200310000-00004

Erickson, S. J., Duvall, S. W., Fuller, J., Schrader, R., MacLean, P. & Lowe, J. R. (2013). Differential associations between maternal scaffolding and toddler emotion regulation in toddlers born preterm and full term. *Early human development*, *89*(9), 699–704. https://doi.org/10.1016/j.earlhumdev.2013.05.003

Estaji, R., Hosseinzadeh, M., Arabgol, F. & Nejati, V. (2024). Transcranial direct current stimulation (tDCS) improves emotion regulation in children with attention-deficit hyperactivity disorder (ADHD). *Scientific reports*, *14*(1), 13889. https://doi.org/10.1038/s41598-024-64886-9

Evrard, D., Charollais, A., Marret, S., Radi, S., Rezrazi, A. & Mellier, D. (2011). Cognitive and emotional regulation developmental issues in preterm infants 12 and 24 months after birth. *European Journal of Developmental Psychology*, *8*(2), 171–184. https://doi.org/10.1080/17405620903504538

Ewell, A., Allard, T., Botdorf, M., Ji, A. & Riggins, T. (2023). Emotion regulation and reactivity are associated with cortical thickness in early to mid-childhood. *Developmental psychobiology*, *65*(6), e22412. https://doi.org/10.1002/dev.22412

Fabes, R. A [Richard A.], Eisenberg, N [Nancy], Karbon, M., Troyer, D. & Switzer, G. (1994). The Relations of Children's Emotion Regulation to Their Vicarious Emotional Responses and Comforting Behaviors. *Child development*, *65*(6), 1678. https://doi.org/10.2307/1131287

Faure, N., Habersaat, S [Stéphanie], Nessi, J., Forcada-Guex, M., Pierrehumbert, B., Ansermet, F., Müller Nix, C. & Borghini, A. (2013). Validation d'une nouvelle grille de codage de la régulation émotionnelle avec le Laboratory Temperament Assessment Battery (Lab-TAB). *Devenir*, *Vol. 25*(1), 27–48. https://doi.org/10.3917/dev.131.0027

Feldman, R. (2015). Mutual influences between child emotion regulation and parent-child reciprocity support development across the first 10 years of life: Implications for developmental psychopathology. *Development and psychopathology*, *27*(4 Pt 1), 1007–1023. https://doi.org/10.1017/S0954579415000656

Feldman, R., Dollberg, D. & Nadam, R. (2011). The expression and regulation of anger in toddlers: relations to maternal behavior and mental representations. *Infant behavior & development*, *34*(2), 310–320. https://doi.org/10.1016/j.infbeh.2011.02.001

Feldman, R. & Greenbaum, C. W. (1997). Affect regulation and synchrony in mother—infant play as precursors to the development of symbolic competence. *Infant mental health journal*, *18*(1), 4–23. https://doi.org/10.1002/(SICI)1097-0355(199721)18:1%3C4::AID-IMHJ2%3E3.0.CO;2-R

Feldman, R. & Klein, P. S. (2003). Toddlers' self-regulated compliance to mothers, caregivers, and fathers: implications for theories of socialization. *Developmental psychology*, *39*(4), 680–692. https://doi.org/10.1037/0012-1649.39.4.680

Feng, X., Shaw, D. S [Daniel S.], Kovacs, M., Lane, T., O'Rourke, F. E. & Alarcon, J. H. (2008). Emotion regulation in preschoolers: the roles of behavioral inhibition, maternal affective behavior, and maternal depression. *Journal of child psychology and psychiatry, and allied disciplines*, *49*(2), 132–141. https://doi.org/10.1111/j.1469-7610.2007.01828.x

Feng, X., Shaw, D. S [Daniel S.] & Moilanen, K. L. (2011). Parental negative control moderates the shyness-emotion regulation pathway to school-age internalizing symptoms. *Journal of abnormal child psychology*, *39*(3), 425–436. https://doi.org/10.1007/s10802-010-9469-z

Fenning, R. M., Baker, J. K. & Moffitt, J. (2018). Intrinsic and Extrinsic Predictors of Emotion Regulation in Children with Autism Spectrum Disorder. *Journal of autism and developmental disorders*, *48*(11), 3858–3870. https://doi.org/10.1007/s10803-018-3647-1

Fernandes, C., Fernandes, M [Marilia], Santos, A. J., Antunes, M., Monteiro, L., Vaughn, B. E. & Verissimo, M. (2021). Early Attachment to Mothers and Fathers: Contributions to Preschoolers' Emotional Regulation. *Frontiers in psychology*, *12*, 660866. https://doi.org/10.3389/fpsyg.2021.660866

Feurer, C., Woody, M. L., James, K. M., Kudinova, A. Y. & Gibb, B. E. (2020). Neighborhood crime risk and resting respiratory sinus arrhythmia in middle childhood: Evidence of gender differences. *Developmental psychobiology*, *62*(2), 232–239. https://doi.org/10.1002/dev.21893

Fiol-Veny, A., Balle, M., La Torre-Luque, A. de & Bornas, X. (2019). Negative cognitive emotion regulation as a predictor of adolescent heart rate variability and entropy under social stress. *Anxiety, stress, and coping*, *32*(6), 641–653. https://doi.org/10.1080/10615806.2019.1641199

Fiskum, C., Andersen, T. G [Tonje Grønning], Aslaksen, P. M., Svendsen, B., Flaten, M. A. & Jacobsen, K. (2017). Cardiac complexity and emotional dysregulation in children. *International journal of psychophysiology : official journal of the International Organization of Psychophysiology*, *121*, 38–45. https://doi.org/10.1016/j.ijpsycho.2017.08.005

Fiskum, C., Andersen, T. G [Tonje G.], Flaten, M. A., Aslaksen, P. M., Bornas, X. & Jacobsen, K. (2019). Reactive Heart Rate Variability and Cardiac Entropy in Children with Internalizing Disorder and Healthy Controls. *Applied psychophysiology and biofeedback*, *44*(4), 309–319. https://doi.org/10.1007/s10484-019-09444-0

Fivaz-Depeursinge, E., Lavanchy-Scaiola, C. & Favez, N. (2010). The Young Infant's Triangular Communication in the Family: Access to Threesome Intersubjectivity? Conceptual Considerations and Case Illustrations. *Psychoanalytic Dialogues*, *20*(2), 125–140. https://doi.org/10.1080/10481881003716214

Forbes, E., Fox, N. A., Cohn, J. F., Galles, S. F. & Kovacs, M. (2006). Children's affect regulation during a disappointment: psychophysiological responses and relation to parent history of depression. *Biological psychology*, *71*(3), 264–277. https://doi.org/10.1016/j.biopsycho.2005.05.004

Forbes, E., Shaw, D. S [Daniel S.], Fox, N. A., Cohn, J. F., Silk, J. S. & Kovacs, M. (2006). Maternal depression, child frontal asymmetry, and child affective behavior as factors in child behavior problems. *Journal of child psychology and psychiatry, and allied disciplines*, *47*(1), 79–87. https://doi.org/10.1111/j.1469-7610.2005.01442.x

Fournier, J. C., Bertocci, M., Ladouceur, C. D., Bonar, L., Monk, K., Abdul-Waalee, H., Versace, A., Santos, J. P. L., Iyengar, S., Birmaher, B. & Phillips, M. L. (2021). Neural function during emotion regulation and future depressive symptoms in youth at risk for affective disorders. *Neuropsychopharmacology : official publication of the American College of Neuropsychopharmacology*, *46*(7), 1340–1347. https://doi.org/10.1038/s41386-021-01001-w

Fox, A. R., Aldrich, J. T., Ahles, J. J. & Mezulis, A. H. (2019). Stress and parenting predict changes in adolescent respiratory sinus arrhythmia. *Developmental psychobiology*, *61*(8), 1214–1224. https://doi.org/10.1002/dev.21863

Frick, M. A., Forslund, T., Fransson, M., Johansson, M., Bohlin, G. & Brocki, K. C. (2018). The role of sustained attention, maternal sensitivity, and infant temperament in the development of early self-regulation. *British journal of psychology (London, England : 1953)*, *109*(2), 277–298. https://doi.org/10.1111/bjop.12266

Friedlmeier, Corapci, F. & Cole, P. M. (2011). Emotion Socialization in Cross‐Cultural Perspective. *Social and Personality Psychology Compass*, *5*(7), 410–427. https://doi.org/10.1111/j.1751-9004.2011.00362.x

Friedlmeier, W [W.] & Trommsdorff, G. (1999). Emotion Regulation in Early Childhood. *Journal of Cross-Cultural Psychology*, *30*(6), 684–711. https://doi.org/10.1177/0022022199030006002

Friedlmeier, W [W.] & Trommsdorff, G. (2001). Entwicklung der Emotionsregulation bei 2- und 3jährigen Mädchen. *Zeitschrift für Entwicklungspsychologie und Pädagogische Psychologie*, *33*(4), 204–214. https://doi.org/10.1026//0049-8637.33.4.204

Fries, W., Alison, B., Ziegler, T. E., Kurian, J. R., Jacoris, S. & Pollak, S. D. (2005). Early experience in humans is associated with changes in neuropeptides critical for regulating social behavior. *Proceedings of the National Academy of Sciences of the United States of America*, *102*(47), 17237–17240. https://doi.org/10.1073/pnas.0504767102

Frost, A., Jelinek, C., Bernard, K., Lind, T. & Dozier, M. (2018). Longitudinal associations between low morning cortisol in infancy and anger dysregulation in early childhood in a CPS-referred sample. *Developmental science*, *21*(3), e12573. https://doi.org/10.1111/desc.12573

Fry, C. M., Ram, N. & Gatzke-Kopp, L. M. (2022). Integrating dynamic and developmental time scales: Emotion-specific autonomic coordination predicts baseline functioning over time. *International journal of psychophysiology : official journal of the International Organization of Psychophysiology*, *171*, 29–37. https://doi.org/10.1016/j.ijpsycho.2021.12.001

Fuertes (2009). Coding system for regulatory patterns in the FFSF.

Fuertes, M., Da Costa Ribeiro, C., Barbosa, M., Gonçalves, J., Teodoro, A. T., Almeida, R., Beeghly, M [Marjorie], Lopes Dos Santos, P. & Lamônica, D. A. C. (2021). Patterns of regulatory behavior in the still-face paradigm at 3 months: A comparison of Brazilian and Portuguese infants. *PloS one*, *16*(6), e0252562. https://doi.org/10.1371/journal.pone.0252562

Fujiki, M., Brinton, B. & Clarke, D. (2002). Emotion Regulation in Children With Specific Language Impairment. *Language, speech, and hearing services in schools*, *33*(2), 102–111. https://doi.org/10.1044/0161-1461(2002/008)

Fung, W. K., Chung, K. K. H., Lam, I. C. B. & Li, N. X. (2020). Bidirectionality in kindergarten children's school readiness and emotional regulation. *Social Development*, *29*(3), 801–817. https://doi.org/10.1111/sode.12434

Gagne, J. R., van Hulle, C. A., Aksan, N., Essex, M. J. & Goldsmith, H. H. (2011). Deriving childhood temperament measures from emotion-eliciting behavioral episodes: scale construction and initial validation. *Psychological Assessment*, *23*(2), 337–353. https://doi.org/10.1037/a0021746

Gago Galvagno, Elgier, A. M [Angel Manuel] & Azzollini, S. C [Susana Celeste] (2022). La asignación universal por hijo y su relación con la regulación emocional en la infancia temprana. Un estudio comportamental. *Ciencias Psicológicas.* Vorab-Onlinepublikation. https://doi.org/10.22235/cp.v16i1.2629

Gago Galvagno, Grandis, M. C. de, Clerici, G. D., Mustaca, A. E., Miller, S. E. & Elgier, A. M [Angel M.] (2019). Regulation During the Second Year: Executive Function and Emotion Regulation Links to Joint Attention, Temperament, and Social Vulnerability in a Latin American Sample. *Frontiers in psychology*, *10*, 1473. https://doi.org/10.3389/fpsyg.2019.01473

Gago Galvagno, Grandis, M. C. de, Jaume, L. C. & Elgier, A. M [Angel M.] (2022). Home environment and its contribution to early childhood regulatory capabilities. *Early Child Development and Care*, *192*(5), 710–723. https://doi.org/10.1080/03004430.2020.1796655

Gago-Galvagno, Miller, S. E., Grandis, C. de, Elgier, A. M [Angel M.], Mustaca, A. E. & Azzollini, S. C [Susana C.] (2022). The still-face paradigm in Latin American mother-child dyads at 2 and 3 years: Effects of socioeconomic status and temperament. *Journal of experimental child psychology*, *217*, 105357. https://doi.org/10.1016/j.jecp.2021.105357

Gair, S. L., Brown, H. R., Breaux, R., Lugo-Candelas, C. I., McDermott, J. M. & Harvey, E. A. (2022). Neural Activity and Emotion Socialization as Predictors of Later Emotion Regulation Difficulties in Children With and Without Hyperactivity/Impulsivity. *Journal of attention disorders*, *26*(12), 1668–1681. https://doi.org/10.1177/10870547221092171

Gallegos, M. I., Murphy, S. E., Benner, A. D., Jacobvitz, D. B. & Hazen, N. L. (2017). Marital, parental, and whole-family predictors of toddlers' emotion regulation: The role of parental emotional withdrawal. *Journal of family psychology : JFP : journal of the Division of Family Psychology of the American Psychological Association (Division 43)*, *31*(3), 294–303. https://doi.org/10.1037/fam0000245

Galyer, K. T. & Evans, I. M. (2001). Pretend Play and the Development of Emotion Regulation in Preschool Children. *Early Child Development and Care*, *166*(1), 93–108. https://doi.org/10.1080/0300443011660108

Gao, M. M., Kaliush, P. R., Brown, M. A., Shakiba, N., Raby, K. L., Crowell, S. E. & Conradt, E. (2022). Unique Contributions of Maternal Prenatal and Postnatal Emotion Dysregulation on Infant Respiratory Sinus Arrhythmia. *Research on Child and Adolescent Psychopathology*, *50*(9), 1219–1232. https://doi.org/10.1007/s10802-022-00914-4

Garavan, H., Ross, T. J. & Stein, E. A. (1999). Right hemispheric dominance of inhibitory control: an event-related functional MRI study. *Proceedings of the National Academy of Sciences of the United States of America*, *96*(14), 8301–8306. https://doi.org/10.1073/pnas.96.14.8301

Garner (1995). Toddlers' emotion regulation behaviors: the roles of social context and family expressiveness. *The Journal of genetic psychology*, *156*(4), 417–430. https://doi.org/10.1080/00221325.1995.9914834

Garner, P. W. (2006). Prediction of prosocial and emotional competence from maternal behavior in African American preschoolers. *Cultural diversity & ethnic minority psychology*, *12*(2), 179–198. https://doi.org/10.1037/1099-9809.12.2.179

Garner, P. W. & Spears, F. M. (2000). Emotion Regulation in Low‐income Preschoolers. *Social Development*, *9*(2), 246–264. https://doi.org/10.1111/1467-9507.00122

Gatzke-Kopp, L. M., Benson, L., Ryan, P. J. & Ram, N. (2020). Cortical and affective regulation of autonomic coordination. *Psychophysiology*, *57*(5), e13544. https://doi.org/10.1111/psyp.13544

Gatzke-Kopp, L. M., Greenberg, M. & Bierman, K. (2015). Children's parasympathetic reactivity to specific emotions moderates response to intervention for early-onset aggression. *Journal of clinical child and adolescent psychology : the official journal for the Society of Clinical Child and Adolescent Psychology, American Psychological Association, Division 53*, *44*(2), 291–304. https://doi.org/10.1080/15374416.2013.862801

Geangu, E., Benga, O., Stahl, D. & Striano, T. (2011). Individual Differences in Infants' Emotional Resonance to a Peer in Distress: Self–Other Awareness and Emotion Regulation. *Social Development*, *20*(3), 450–470. https://doi.org/10.1111/j.1467-9507.2010.00596.x

Geckeler, K. C., Barch, D. M. & Karcher, N. R. (2022). Associations between social behaviors and experiences with neural correlates of implicit emotion regulation in middle childhood. *Neuropsychopharmacology : official publication of the American College of Neuropsychopharmacology*, *47*(6), 1169–1179. https://doi.org/10.1038/s41386-022-01286-5

Geldhof, G. J., Flynn, E., Olsen, S. G., Mueller, M. K., Gandenberger, J., Witzel, D. D. & Morris, K. N. (2021). Emotion regulation and specificity: The impact of animal-assisted interventions on classroom behavior. *Journal of Applied Developmental Psychology*, *73*, 101253. https://doi.org/10.1016/j.appdev.2021.101253

Gennis, H. G., Flora, D. B., Norton, L., McMurtry, C. M., Merlano, T. E., Zaghi, A., Flanders, D., Weinberg, E., Savlov, D., Garfield, H. & Pillai Riddell, R. R. (2023). Understanding the concurrent and predictive relations between child-led emotion regulation behaviors and pain during vaccination in toddlerhood. *Pain*, *164*(6), 1291–1302. https://doi.org/10.1097/j.pain.0000000000002816

Gentzler, A. L., Santucci, A. K., Kovacs, M. & Fox, N. A. (2009). Respiratory sinus arrhythmia reactivity predicts emotion regulation and depressive symptoms in at-risk and control children. *Biological psychology*, *82*(2), 156–163. https://doi.org/10.1016/j.biopsycho.2009.07.002

Gianino, A. & Tronick, E. Z. (2013). The Mutual Regulation Model: The Infant's Self and Interactive Regulation and Coping and Defensive Capacities. In T. M. Field, P. Mccabe & N. Schneiderman (Hrsg.), *Stress and Coping Across Development* (S. 1–22). Psychology Press.

Gilbert, K. E., Luking, K. R [Katherine Rose], Pagliaccio, D., L Luby, J. & Barch, D. M. (2019). Dampening Positive Affect and Neural Reward Responding in Healthy Children: Implications for Affective Inflexibility. *Journal of clinical child and adolescent psychology : the official journal for the Society of Clinical Child and Adolescent Psychology, American Psychological Association, Division 53*, *48*(1), 120–130. https://doi.org/10.1080/15374416.2016.1233502

Gilliom, M., Shaw, D. S [Daniel S.], Beck, J. E., Schonberg, M. A. & Lukon, J. L. (2002). Anger regulation in disadvantaged preschool boys: strategies, antecedents, and the development of self-control. *Developmental psychology*, *38*(2), 222–235. https://doi.org/10.1037/0012-1649.38.2.222

Ginnell, L., O'Carroll, S., Ledsham, V., Jiménez Sánchez, L., Stoye, D. Q., Sullivan, G., Hall, J., Homer, N. Z. M., Boardman, J. P., Fletcher-Watson, S. & Reynolds, R. M. (2022). Emotion regulation and cortisol response to the still-face procedure in preterm and full-term infants. *Psychoneuroendocrinology*, *141*, 105760. https://doi.org/10.1016/j.psyneuen.2022.105760

Gleason, T. R., Tarsha, M. S., Kurth, A. M. & Narvaez, D. (2021). Opportunities for free play and young children's autonomic regulation. *Developmental psychobiology*, *63*(6), e22134. https://doi.org/10.1002/dev.22134

Goldsmith. (1993). Temperament: Variability in developing emotion systems. In M. Lewis & J. Haviland (Hrsg.), *Handbook of emotions* (S. 353–364). New York: Guilford Press.

Goldsmith (1999). The laboratory temperament assessment battery manual (Lab-TAB).

Goldsmith, Lemery, K. S., Schmidt, N. L., Schmidt, C. K., Chow, O. K. & Justen, A. A. (2010). Field administration manual for the Laboratory Temperament Assessment Battery: Middle Childhood Version. *Technical Report. Department of Psychology, University of Wisconsin–Madison.*

Goldsmith & Rothbart, M. K [M. K.] (1996). Prelocomotor and Locomotor Laboratory Temperament Assessment Battery, Lab-TAB; version 3.0. Technical Manual, Department of Psychology, University of Wisconsin, Madison, WI.

Gottman, J. M. & Katz, L. F [Lynn F.] (1989). Effects of marital discord on young children's peer interaction and health. *Developmental psychology*, *25*(3), 373–381. https://doi.org/10.1037/0012-1649.25.3.373

Gottman, J. M., Katz, L. F [Lynn Fainsilber] & Hooven, C. (1996). Parental meta-emotion philosophy and the emotional life of families: Theoretical models and preliminary data. *Journal of Family Psychology*, *10*(3), 243–268. https://doi.org/10.1037/0893-3200.10.3.243

Grabell, A. S., Huppert, T. J., Fishburn, F. A., Li, Y [Yanwei], Hlutkowsky, C. O., Jones, H. M., Wakschlag, L. S. & Perlman, S. B. (2019). Neural correlates of early deliberate emotion regulation: Young children's responses to interpersonal scaffolding. *Developmental cognitive neuroscience*, *40*, 100708. https://doi.org/10.1016/j.dcn.2019.100708

Grabell, A. S., Huppert, T. J., Fishburn, F. A., Li, Y [Yanwei], Jones, H. M., Wilett, A. E., Bemis, L. M. & Perlman, S. B. (2018). Using facial muscular movements to understand young children's emotion regulation and concurrent neural activation. *Developmental science*, *21*(5), e12628. https://doi.org/10.1111/desc.12628

Grabell, A. S., Olson, S. L., Miller, A. L., Kessler, D. A., Felt, B., Kaciroti, N., Wang, L. & Tardif, T. (2015). The impact of culture on physiological processes of emotion regulation: a comparison of US and Chinese preschoolers. *Developmental science*, *18*(3), 420–435. https://doi.org/10.1111/desc.12227

Grady, J. S. & Callan, D. (2019). Shy toddlers act bold: The roles of respiratory sinus arrhythmia and parent emotion language. *Infant behavior & development*, *55*, 32–37. https://doi.org/10.1016/j.infbeh.2019.02.005

Granat, A., Gadassi, R., Gilboa-Schechtman, E. & Feldman, R. (2017). Maternal depression and anxiety, social synchrony, and infant regulation of negative and positive emotions. *Emotion (Washington, D.C.)*, *17*(1), 11–27. https://doi.org/10.1037/emo0000204

Granic, I., Meusel, L.‑A., Lamm, C., Woltering, S. & Lewis, M. D. (2012). Emotion regulation in children with behavior problems: linking behavioral and brain processes. *Development and psychopathology*, *24*(3), 1019–1029. https://doi.org/10.1017/S095457941200051X

Gratz & Roemer, L. (2004). Multidimensional Assessment of Emotion Regulation and Dysregulation: Development, Factor Structure, and Initial Validation of the Difficulties in Emotion Regulation Scale. *Journal of Psychopathology and Behavioral Assessment*, *26*(1), 41–54. https://doi.org/10.1023/B:JOBA.0000007455.08539.94

Gratz, K. L., Kiel, E. J., Latzman, R. D., Elkin, T. D., Moore, S. A. & Tull, M. T. (2014). Emotion: empirical contribution. Maternal borderline personality pathology and infant emotion regulation: examining the influence of maternal emotion-related difficulties and infant attachment. *Journal of personality disorders*, *28*(1), 52–69. https://doi.org/10.1521/pedi.2014.28.1.52

Gray, S. A. O., Lipschutz, R. S. & Scheeringa, M. S. (2018). Young Children's Physiological Reactivity during Memory Recall: Associations with Posttraumatic Stress and Parent Physiological Synchrony. *Journal of abnormal child psychology*, *46*(4), 871–880. https://doi.org/10.1007/s10802-017-0326-1

Graziano, Calkins, S. D [S. D.] & Keane, S. P [S. P.] (2010). Toddler self-regulation skills predict risk for pediatric obesity. *International journal of obesity (2005)*, *34*(4), 633–641. https://doi.org/10.1038/ijo.2009.288

Graziano, Calkins, S. D. & Keane, S. P [Susan P.] (2011). Sustained Attention Development during the Toddlerhood to Preschool Period: Associations with Toddlers' Emotion Regulation Strategies and Maternal Behavior. *Infant and Child Development*, *20*(6), 389–408. https://doi.org/10.1002/icd.731

Graziano & Hart, K. (2016). Beyond behavior modification: Benefits of social-emotional/self-regulation training for preschoolers with behavior problems. *Journal of school psychology*, *58*, 91–111. https://doi.org/10.1016/j.jsp.2016.07.004

Graziano, Keane, S. P [S. P.] & Calkins, S. D [S. D.] (2010). Maternal Behavior and Children's Early Emotion Regulation Skills Differentially Predict Development of Children's Reactive Control and Later Effortful Control. *Infant and Child Development*, *19*(4), 333–353. https://doi.org/10.1002/ICD.670

Graziano, Kelleher, R., Calkins, S. D [S. D.], Keane, S. P [S. P.] & Brien, M. O. (2013). Predicting weight outcomes in preadolescence: the role of toddlers' self-regulation skills and the temperament dimension of pleasure. *International journal of obesity (2005)*, *37*(7), 937–942. https://doi.org/10.1038/ijo.2012.165

Graziano, P. A., Landis, T., Maharaj, A., Ros-Demarize, R., Hart, K. C. & Garcia, A. (2022). Differentiating Preschool Children with Conduct Problems and Callous-Unemotional Behaviors through Emotion Regulation and Executive Functioning. *Journal of clinical child and adolescent psychology : the official journal for the Society of Clinical Child and Adolescent Psychology, American Psychological Association, Division 53*, *51*(2), 170–182. https://doi.org/10.1080/15374416.2019.1666399

Graziano, P. A., Slavec, J., Hart, K., Garcia, A. & Pelham, W. E. (2014). Improving School Readiness in Preschoolers with Behavior Problems: Results from a Summer Treatment Program. *Journal of Psychopathology and Behavioral Assessment*, *36*(4), 555–569. https://doi.org/10.1007/s10862-014-9418-1

Green, L. M., Genaro, B. G., Ratcliff, K. A [Kizzann Ashana], Cole, P. M. & Ram, N. (2023). Investigating the developmental timing of self-regulation in early childhood. *International Journal of Behavioral Development*, *47*(2), 101–110. https://doi.org/10.1177/01650254221111788

Grolnick, W. S., Bridges, L. J. & Connell, J. P. (1996). Emotion Regulation in Two-Year-Olds: Strategies and Emotional Expression in Four Contexts. *Child development*, *67*(3), 928. https://doi.org/10.2307/1131871

Gross (1998). The Emerging Field of Emotion Regulation: An Integrative Review. *Review of General Psychology*, *2*(3), 271–299. https://doi.org/10.1037/1089-2680.2.3.271

Gross. (1999a). Emotion and emotion regulation. In J. O. P. Pervin LA (Hrsg.), *Handbook of personality: Theory and research* (2nd ed, S. 525–552). Guilford; New York.

Gross (1999b). Emotion Regulation: Past, Present, Future. *Cognition & emotion*, *13*(5), 551–573. https://doi.org/10.1080/026999399379186

Gross (Hrsg.). (2007). *Handbook of Emotion Regulation*. New York, NY: Guilford Press.

Gross & Muñoz (1995). Emotion regulation and mental health. *Clinical Psychology: Science and Practice*(2(2), 151–164.

Gross, J. J. (2015). Emotion Regulation: Current Status and Future Prospects. *Psychological Inquiry*, *26*(1), 1–26. https://doi.org/10.1080/1047840X.2014.940781

Gross, J. J., Sheppes, G. & Urry, H. L. (2011). Cognition and Emotion Lecture at the 2010 SPSP Emotion Preconference. *Cognition & emotion*, *25*(5), 765–781. https://doi.org/10.1080/02699931.2011.555753

Gulsrud, A. C., Jahromi, L. B. & Kasari, C. (2010). The co-regulation of emotions between mothers and their children with autism. *Journal of autism and developmental disorders*, *40*(2), 227–237. https://doi.org/10.1007/s10803-009-0861-x

Guy, L., Souders, M., Bradstreet, L., DeLussey, C. & Herrington, J. D. (2014). Brief report: emotion regulation and respiratory sinus arrhythmia in autism spectrum disorder. *Journal of autism and developmental disorders*, *44*(10), 2614–2620. https://doi.org/10.1007/s10803-014-2124-8

Gyurak, A., Gross, J. J. & Etkin, A. (2011). Explicit and Implicit Emotion Regulation: A Dual-Process Framework. *Cognition & emotion*, *25*(3), 400–412. https://doi.org/10.1080/02699931.2010.544160

Habersaat, S [Stephanie], Borghini, A., Faure, N., Nessi, J., Forcada-Guex, M., Pierrehumbert, B., Ansermet, F. & Müller-Nix, C. (2013). Emotional and neuroendocrine regulation in very preterm and full-term infants at six months of age. *European Journal of Developmental Psychology*, *10*(6), 691–706. https://doi.org/10.1080/17405629.2013.787924

Hagstrøm, J., Maigaard, K., Pagsberg, A. K., Skov, L., Plessen, K. J. & Vangkilde, S. (2020). Reappraisal is an effective emotion regulation strategy in children with Tourette syndrome and ADHD. *Journal of behavior therapy and experimental psychiatry*, *68*, 101541. https://doi.org/10.1016/j.jbtep.2019.101541

Hagstrøm, J., Spang, K. S [Katrine S.], Christiansen, B. M., Maigaard, K., Vangkilde, S., Esbjørn, B. H., Jepsen, J. R. M. & Plessen, K. J. (2019). The Puzzle of Emotion Regulation: Development and Evaluation of the Tangram Emotion Coding Manual for Children. *Frontiers in psychiatry*, *10*, 723. https://doi.org/10.3389/fpsyt.2019.00723

Hagstrøm, J., Spang, K. S [Katrine S.], Vangkilde, S., Maigaard, K., Skov, L., Pagsberg, A. K., Jepsen, J. R. M. & Plessen, K. J. (2021). An observational study of emotion regulation in children with Tourette syndrome. *Journal of child psychology and psychiatry, and allied disciplines*, *62*(6), 790–797. https://doi.org/10.1111/jcpp.13375

Halfon, S., Bekar, Ö., Ababay, S. & Dorlach, G. Ç. (2017). Dyadic Mental State Talk and Sophistication of Symbolic Play between Parents and Children with Behavioral Problems. *Journal of Infant, Child, and Adolescent Psychotherapy*, *16*(4), 291–307. https://doi.org/10.1080/15289168.2017.1370952

Halfon, S. & Bulut, P. (2019). Mentalization and the growth of symbolic play and affect regulation in psychodynamic therapy for children with behavioral problems. *Psychotherapy research : journal of the Society for Psychotherapy Research*, *29*(5), 666–678. https://doi.org/10.1080/10503307.2017.1393577

Halfon, S., Yılmaz, M. & Çavdar, A. (2019). Mentalization, session-to-session negative emotion expression, symbolic play, and affect regulation in psychodynamic child psychotherapy. *Psychotherapy (Chicago, Ill.)*, *56*(4), 555–567. https://doi.org/10.1037/pst0000201

Halligan, S. L., Cooper, P. J [Peter J.], Fearon, P., Wheeler, S. L., Crosby, M. & Murray, L [Lynne] (2013). The longitudinal development of emotion regulation capacities in children at risk for externalizing disorders. *Development and psychopathology*, *25*(2), 391–406. https://doi.org/10.1017/S0954579412001137

Ham, J. & Tronick, E [Ed] (2009). Relational psychophysiology: lessons from mother-infant physiology research on dyadically expanded states of consciousness. *Psychotherapy research : journal of the Society for Psychotherapy Research*, *19*(6), 619–632. https://doi.org/10.1080/10503300802609672

Hannesdóttir, D. K., Doxie, J., Bell, M. A., Ollendick, T. H. & Wolfe, C. D. (2010). A longitudinal study of emotion regulation and anxiety in middle childhood: Associations with frontal EEG asymmetry in early childhood. *Developmental psychobiology*, *52*(2), 197–204. https://doi.org/10.1002/dev.20425

Harden, B. J., Panlilio, C., Morrison, C., Duncan, A. D., Duchene, M. & Clyman, R. B. (2017). Emotion Regulation of Preschool Children in Foster Care: The Influence of Maternal Depression and Parenting. *Journal of Child and Family Studies*, *26*(4), 1124–1134. https://doi.org/10.1007/s10826-016-0636-x

Hariri, A. R., Bookheimer, S. Y. & Mazziotta, J. C. (2000). Modulating emotional responses: effects of a neocortical network on the limbic system. *Neuroreport*, *11*(1), 43–48. https://doi.org/10.1097/00001756-200001170-00009

Harrington, E. (2021). *Child Emotional Reactivity and Regulation: Parental Influences and Implications for Socioemotional and Academic Components of School ReadinessREADINESS* [Dissertation].

Hart, S. L. & Behrens, K. Y. (2013). Regulation of Jealousy Protest in the Context of Reunion Following Differential Treatment. *Infancy : the official journal of the International Society on Infant Studies*, *18*(6), 1076–1110. https://doi.org/10.1111/infa.12024

Hassan, R., Smith, C. L., Schmidt, L. A., Brook, C. A. & Bell, M. A. (2023). Developmental patterns of children's shyness: Relations with physiological, emotional, and regulatory responses to being treated unfairly. *Child development*, *94*(6), 1745–1761. https://doi.org/10.1111/cdev.13961

Hastings, P. D [Paul D.], Nuselovici, J. N., Utendale, W. T., Coutya, J., McShane, K. E. & Sullivan, C. (2008). Applying the polyvagal theory to children's emotion regulation: Social context, socialization, and adjustment. *Biological psychology*, *79*(3), 299–306. https://doi.org/10.1016/j.biopsycho.2008.07.005

Hazen, N. L., McFarland, L., Jacobvitz, D. & Boyd‐Soisson, E. (2010). Fathers’ frightening behaviours and sensitivity with infants: relations with fathers’ attachment representations, father–infant attachment, and children’s later outcomes. *Early Child Development and Care*, *180*(1-2), 51–69. https://doi.org/10.1080/03004430903414703

Heeman, E. J., Forslund, T., Frick, M. A., Frick, A., Jónsdóttir, L. K. & Brocki, K. C. (2024). Predicting emotion regulation in typically developing toddlers: Insights into the joint and unique influences of various contextual predictors. *International Journal of Behavioral Development*, *48*(5), 398–410. https://doi.org/10.1177/01650254241239956

Helmsen, J. (2011). *Einfluss emotionaler und sozial-kognitiver Prozesse auf das aggressive Verhalten bei Kindern im Kindergartenalter* [Dissertation].

Hernandez, E., Carmichael, K., Kiliç, Ş. & Dunsmore, J. C. (2019). Linguistic indirectness in parent–preschooler reminiscing about emotion‐related events: Links with emotion regulation and psychosocial adjustment. *Social Development*, *28*(4), 761–781. https://doi.org/10.1111/sode.12345

Heron-Delaney, M., Kenardy, J. A., Brown, E. A., Jardine, C., Bogossian, F., Neuman, L., Dassel, T. de & Pritchard, M. (2016). Early Maternal Reflective Functioning and Infant Emotional Regulation in a Preterm Infant Sample at 6 Months Corrected Age. *Journal of pediatric psychology*, *41*(8), 906–914. https://doi.org/10.1093/jpepsy/jsv169

Hessler, D. M. & Fainsilber Katz, L. (2007). Children's emotion regulation: Self-report and physiological response to peer provocation. *Developmental psychology*, *43*(1), 27–38. https://doi.org/10.1037/0012-1649.43.1.27

Hill, A. L., Degnan, K. A., Calkins, S. D. & Keane, S. P [Susan P.] (2006). Profiles of externalizing behavior problems for boys and girls across preschool: the roles of emotion regulation and inattention. *Developmental psychology*, *42*(5), 913–928. https://doi.org/10.1037/0012-1649.42.5.913

Hirschler-Guttenberg, Y., Feldman, R., Ostfeld-Etzion, S., Laor, N. & Golan, O. (2015). Self- and Co-regulation of Anger and Fear in Preschoolers with Autism Spectrum Disorders: The Role of Maternal Parenting Style and Temperament. *Journal of autism and developmental disorders*, *45*(9), 3004–3014. https://doi.org/10.1007/s10803-015-2464-z

Hirschler-Guttenberg, Y., Golan, O., Ostfeld-Etzion, S. & Feldman, R. (2015). Mothering, fathering, and the regulation of negative and positive emotions in high-functioning preschoolers with autism spectrum disorder. *Journal of child psychology and psychiatry, and allied disciplines*, *56*(5), 530–539. https://doi.org/10.1111/jcpp.12311

Hoffman, C., Crnic, K. A [Keith A.] & Baker, J. K. (2006). Maternal Depression and Parenting: Implications for Children's Emergent Emotion Regulation and Behavioral Functioning. *Parenting*, *6*(4), 271–295. https://doi.org/10.1207/s15327922par0604_1

Hoffmann, F., Singer, T. & Steinbeis, N. (2015). Children's Increased Emotional Egocentricity Compared to Adults Is Mediated by Age-Related Differences in Conflict Processing. *Child development*, *86*(3), 765–780. https://doi.org/10.1111/cdev.12338

Housman, D. K., Cabral, H., Aniskovich, K. & Denham, S. A. (2023). The impact of begin to ECSEL on children’s self-regulation, executive functions and learning. *Early Child Development and Care*, *193*(2), 159–173. https://doi.org/10.1080/03004430.2022.2071869

Hua, M., Han, Z. R. & Zhou, R. (2015). Cognitive Reappraisal in Preschoolers: Neuropsychological Evidence of Emotion Regulation From an ERP Study. *Developmental neuropsychology*, *40*(5), 279–290. https://doi.org/10.1080/87565641.2015.1069827

Huffman, L. G. & Oshri, A. (2022). Continuity versus change in latent profiles of emotion regulation and working memory during adolescence. *Developmental cognitive neuroscience*, *58*, 101177. https://doi.org/10.1016/j.dcn.2022.101177

Hughes, S. O., Power, T. G., O'Connor, T. M. & Orlet Fisher, J. (2015). Executive functioning, emotion regulation, eating self-regulation, and weight status in low-income preschool children: how do they relate? *Appetite*, *89*, 1–9. https://doi.org/10.1016/j.appet.2015.01.009

Hum, K. M., Manassis, K. & Lewis, M. D. (2013). Neural mechanisms of emotion regulation in childhood anxiety. *Journal of child psychology and psychiatry, and allied disciplines*, *54*(5), 552–564. https://doi.org/10.1111/j.1469-7610.2012.02609.x

Hurrell, K. E., Houwing, F. L. & Hudson, J. L. (2017). Parental Meta-Emotion Philosophy and Emotion Coaching in Families of Children and Adolescents with an Anxiety Disorder. *Journal of abnormal child psychology*, *45*(3), 569–582. https://doi.org/10.1007/s10802-016-0180-6

Ip, K. I., Miller, A. L., Wang, L., Felt, B., Olson, S. L. & Tardif, T. (2024). Emotion regulation as a complex system: A multi-contextual and multi- level approach to understanding emotion expression and cortisol reactivity among Chinese and US preschoolers. *Developmental science*, *27*(5), e13446. https://doi.org/10.1111/desc.13446

Izard, C. E [C. E.], Dougherty, L. M. & Hembree, E. A. (1995). *A System for Identifying Affect Expressions by Holistic Judgements (Affex).: Carroll E. Izard, Linda M. Dougherty, and Elizabeth A. Hembree. Manual*. University Media Services, University of Delaware. https://books.google.de/books?id=wz10PwAACAAJ

Jahromi, L. B., Meek, S. E. & Ober-Reynolds, S. (2012). Emotion regulation in the context of frustration in children with high functioning autism and their typical peers. *Journal of child psychology and psychiatry, and allied disciplines*, *53*(12), 1250–1258. https://doi.org/10.1111/j.1469-7610.2012.02560.x

Jin, Z., Zhang, X. & Han, Z. R. (2017). Parental Emotion Socialization and Child Psychological Adjustment among Chinese Urban Families: Mediation through Child Emotion Regulation and Moderation through Dyadic Collaboration. *Frontiers in psychology*, *8*, 2198. https://doi.org/10.3389/fpsyg.2017.02198

Jones (2013). Social competence of preschool children born very preterm. *Early human development*, *89*(10), 795–802. https://doi.org/10.1016/j.earlhumdev.2013.06.008

Jones, R. M., Conture, E. G. & Walden, T. A. (2014). Emotional reactivity and regulation associated with fluent and stuttered utterances of preschool-age children who stutter. *Journal of communication disorders*, *48*, 38–51. https://doi.org/10.1016/j.jcomdis.2014.02.001

Jones, R. M., Walden, T. A., Conture, E. G., Erdemir, A., Lambert, W. E. & Porges, S. W. (2017). Executive Functions Impact the Relation Between Respiratory Sinus Arrhythmia and Frequency of Stuttering in Young Children Who Do and Do Not Stutter. *Journal of speech, language, and hearing research : JSLHR*, *60*(8), 2133–2150. https://doi.org/10.1044/2017_JSLHR-S-16-0113

Joormann, J., Cooney, R. E., Henry, M. L. & Gotlib, I. H. (2012). Neural correlates of automatic mood regulation in girls at high risk for depression. *Journal of Abnormal Psychology*, *121*(1), 61–72. https://doi.org/10.1037/a0025294

Kahle, S., Miller, J. G., Helm, J. L. & Hastings, P. D [Paul D.] (2018). Linking autonomic physiology and emotion regulation in preschoolers: The role of reactivity and recovery. *Developmental psychobiology*, *60*(7), 775–788. https://doi.org/10.1002/dev.21746

Kahle, S., Miller, J. G., Lopez, M. & Hastings, P. D [Paul D.] (2016). Sympathetic recovery from anger is associated with emotion regulation. *Journal of experimental child psychology*, *142*, 359–371. https://doi.org/10.1016/j.jecp.2015.10.004

Kahle, S., Miller, J. G., Troxel, N. R. & Hastings, P. D [Paul D.] (2021). The development of frustration regulation over early childhood: Links between attention diversion and parasympathetic activity. *Emotion (Washington, D.C.)*, *21*(6), 1252–1267. https://doi.org/10.1037/emo0000947

Kalpidou, M. D., Power, T. G., Cherry, K. E. & Gottfried, N. W. (2004). Regulation of emotion and behavior among 3- and 5-year-olds. *The Journal of general psychology*, *131*(2), 159–178. https://doi.org/10.3200/GENP.131.2.159-180

Katz, L. F [Lynn Fainsilber] & Gurtovenko, K. (2015). Posttraumatic stress and emotion regulation in survivors of intimate partner violence. *Journal of Family Psychology*, *29*(4), 528–536. https://doi.org/10.1037/fam0000128

Katz, L. F [Lynn Fainsilber], Heleniak, C., Kawamura, J. & Jakubiak, J. (2015). Emotion regulation, internalizing symptoms and somatic complaints in pediatric survivors of acute lymphoblastic leukemia. *Psycho-oncology*, *24*(11), 1536–1544. https://doi.org/10.1002/pon.3762

Kennedy, A. E., Rubin, K. H., Hastings, P. D [Paul D.] & Maisel, B. (2004). Longitudinal relations between child vagal tone and parenting behavior: 2 to 4 years. *Developmental psychobiology*, *45*(1), 10–21. https://doi.org/10.1002/dev.20013

Kerr, M. L., Rasmussen, H. F., Smiley, P. A., Buttitta, K. V. & Borelli, J. L. (2021). The development of toddlers’ emotion regulation within the family system: associations with observed parent-child synchrony and interparental relationship satisfaction. *Early childhood research quarterly*, *57*, 215–227. https://doi.org/10.1016/j.ecresq.2021.06.004

Khoury, J. E., Gonzalez, A., Levitan, R., Masellis, M., Basile, V. & Atkinson, L. (2016). Infant Emotion Regulation Strategy Moderates Relations between Self‐Reported Maternal Depressive Symptoms and Infant HPA Activity. *Infant and Child Development*, *25*(1), 64–83. https://doi.org/10.1002/icd.1916

Kiani, B., Hadianfard, H., Mitchell, J. T. & Weiss, M. D. (2024). Examining emotion regulation using a distraction and reappraisal task in children and adolescents with and without ADHD. *Current Psychology*, *43*(6), 5652–5660. https://doi.org/10.1007/s12144-023-04766-z

Kidwell, S. L. & Barnett, D. (2007). Adaptive Emotion Regulation among Low-Income African American Children. *Merrill-Palmer Quarterly*, *53*(2), 155–183. https://doi.org/10.1353/mpq.2007.0011

Kiel, E. J., Price, N. N. & Premo, J. E. (2020). Maternal comforting behavior, toddlers' dysregulated fear, and toddlers' emotion regulatory behaviors. *Emotion (Washington, D.C.)*, *20*(5), 793–803. https://doi.org/10.1037/emo0000600

Kim, Stifter, C. A., Philbrook, L. E. & Teti, D. M. (2014). Infant emotion regulation: relations to bedtime emotional availability, attachment security, and temperament. *Infant behavior & development*, *37*(4), 480–490. https://doi.org/10.1016/j.infbeh.2014.06.006

Kim, Y., Williams, A. I., Liu, C. & Zhou, Q. (2023). Dynamic associations between emotion expressions and strategy use in Chinese American and Mexican American preschoolers. *Emotion (Washington, D.C.)*, *23*(2), 460–472. https://doi.org/10.1037/emo0001100

Kirsch, F., Busching, R., Rohlf, H. & Krahé, B. (2019). Using behavioral observation for the longitudinal study of anger regulation in middle childhood. *Applied Developmental Science*, *23*(2), 105–118. https://doi.org/10.1080/10888691.2017.1325325

Kirsch, F., Rohlf, H. & Krahé, B. (2015). Measuring anger regulation in middle childhood through behavioural observation: a longitudinal validation. *European Journal of Developmental Psychology*, *12*(6), 718–727. https://doi.org/10.1080/17405629.2015.1101375

Kiser, L., Fishbein, D., Gatzke-Kopp, L., Vivrette, R., Creavey, K., Stevenson, J., Medoff, D. & Busuito, A. (2019). Physiological Regulation among Caregivers and their Children: Relations with Trauma History, Symptoms, and Parenting Behavior. *Journal of Child and Family Studies*, *28*(11), 3098–3109. https://doi.org/10.1007/s10826-019-01487-5

Kling, J. L. & Brooker, R. J. (2024). Socioeconomic status moderates neural markers of cognitive reappraisal across preschool. *Biological psychology*, *186*, 108738. https://doi.org/10.1016/j.biopsycho.2023.108738

Klinge, J. L., Warschburger, P., Busching, R. & Klein, A. M. (2023). Self-regulation facets differentially predict internalizing symptom trajectories from middle childhood to early adolescence: a longitudinal multimethod study. *Child and adolescent psychiatry and mental health*, *17*(1), 120. https://doi.org/10.1186/s13034-023-00670-3

Klinnert, M. D., McQuaid, E. L., McCormick, D., Adinoff, A. D. & Bryant, N. E. (2000). A multimethod assessment of behavioral and emotional adjustment in children with asthma. *Journal of pediatric psychology*, *25*(1), 35–46. https://doi.org/10.1093/jpepsy/25.1.35

Kochanska, G., Philibert, R. A. & Barry, R. A. (2009). Interplay of genes and early mother-child relationship in the development of self-regulation from toddler to preschool age. *Journal of child psychology and psychiatry, and allied disciplines*, *50*(11), 1331–1338. https://doi.org/10.1111/j.1469-7610.2008.02050.x

Kogan, N. & Carter, A. S. (1996). Mother-infant reengagement following the still-face: The role of maternal emotional availability an infant affect regulation. *Infant Behavior and Development*, *19*(3), 359–370. https://doi.org/10.1016/S0163-6383(96)90034-X

Kolak, A. M. & Volling, B. L. (2011). Sibling jealousy in early childhood: longitudinal links to sibling relationship quality. *Infant and Child Development*, *20*(2), 213–226. https://doi.org/10.1002/icd.690

Konishi, H., Karsten, A. & Vallotton, C. D. (2018). TODDLERS' USE OF GESTURE AND SPEECH IN SERVICE OF EMOTION REGULATION DURING DISTRESSING ROUTINES. *Infant mental health journal*, *39*(6), 730–750. https://doi.org/10.1002/imhj.21740

Krzeczkowski, J. E., Schmidt, L. A. & van Lieshout, R. J. (2021). Changes in infant emotion regulation following maternal cognitive behavioral therapy for postpartum depression. *Depression and anxiety*, *38*(4), 412–421. https://doi.org/10.1002/da.23130

Kuiper, K., Swaab, H., Tartaglia, N. & van Rijn, S. (2023). (Not) getting what you want: frustration and emotion regulation in children with sex chromosome trisomies. *Endocrine connections*, *12*(6). https://doi.org/10.1530/EC-22-0442

Kurki, K., Järvelä, S., Mykkänen, A. & Määttä, E. (2015). Investigating children's emotion regulation in socio-emotionally challenging classroom situations. *Early Child Development and Care*, *185*(8), 1238–1254. https://doi.org/10.1080/03004430.2014.988710

Kurki, K., Järvenoja, H., Järvelä, S. & Mykkänen, A. (2017). Young children’s use of emotion and behaviour regulation strategies in socio-emotionally challenging day-care situations. *Early childhood research quarterly*, *41*, 50–62. https://doi.org/10.1016/j.ecresq.2017.06.002

Lakes, K. D. (2013). Measuring self-regulation in a physically active context: Psychometric analyses of scores derived from an observer-rated measure of self-regulation. *Mental health and physical activity*, *8*(3), 189–196. https://doi.org/10.1016/j.mhpa.2013.09.003

Lamm, C., Granic, I., Zelazo, P. D. & Lewis, M. D. (2011). Magnitude and chronometry of neural mechanisms of emotion regulation in subtypes of aggressive children. *Brain and cognition*, *77*(2), 159–169. https://doi.org/10.1016/j.bandc.2011.06.008

Landis, T. D., Garcia, A. M., Hart, K. C. & Graziano, P. A. (2021). Differentiating Symptoms of ADHD in Preschoolers: The Role of Emotion Regulation and Executive Function. *Journal of attention disorders*, *25*(9), 1260–1271. https://doi.org/10.1177/1087054719896858

Lang, P. J. (2005). *International affective picture system (IAPS): Affective ratings of pictures and instruction manual*. Technical Report A-6). University of Florida, Gainesville.

Lapierre, M. A. (2016). Emotion regulation and young children’s consumer behavior. *Young Consumers*, *17*(2), 168–182. https://doi.org/10.1108/YC-11-2015-00566

Latham, M. D., Cook, N., Simmons, J. G., Byrne, M. L., Kettle, J. W. L., Schwartz, O., Vijayakumar, N., Whittle, S. & Allen, N. B. (2017). Physiological correlates of emotional reactivity and regulation in early adolescents. *Biological psychology*, *127*, 229–238. https://doi.org/10.1016/j.biopsycho.2017.07.018

Leaberry, K. D., Rosen, P. J., Fogleman, N. D., Walerius, D. M. & Slaughter, K. E. (2018). Physiological Emotion Regulation in Children with ADHD with and without Comorbid Internalizing Disorders: a Preliminary Study. *Journal of Psychopathology and Behavioral Assessment*, *40*(3), 452–464. https://doi.org/10.1007/s10862-018-9644-z

Leary, A. & Katz, L. F [Lynn Fainsilber] (2004). Coparenting, family-level processes, and peer outcomes: the moderating role of vagal tone. *Development and psychopathology*, *16*(3), 593–608. https://doi.org/10.1017/S0954579404004687

Lee, O'Brien, J. R., Binion, G., Lewis, J. K. & Zalewski, M. (2023). Supportive Emotion Socialization Mitigates Risk Between Maternal Emotion Regulation Difficulties and Preschooler Emotion Regulation. *Journal of Child and Family Studies*, *32*(3), 824–832. https://doi.org/10.1007/s10826-022-02404-z

Lee, C. A., Milich, R., Lorch, E. P., Flory, K., Owens, J. S., Lamont, A. E. & Evans, S. W. (2018). Forming first impressions of children: the role of attention-deficit/hyperactivity disorder symptoms and emotion dysregulation. *Journal of child psychology and psychiatry, and allied disciplines*, *59*(5), 556–564. https://doi.org/10.1111/jcpp.12835

Leupoldt, A. von, Rohde, J., Beregova, A., Thordsen-Sörensen, I., zur Nieden, J. & Dahme, B. (2007). Films for eliciting emotional states in children. *Behavior research methods*, *39*(3), 606–609. https://doi.org/10.3758/BF03193032

Leventon, J. S. & Bauer, P. J. (2016). Emotion regulation during the encoding of emotional stimuli: Effects on subsequent memory. *Journal of experimental child psychology*, *142*, 312–333. https://doi.org/10.1016/j.jecp.2015.09.024

Leventon, J. S., Merrill, N. A. & Bauer, P. J. (2019). Neural response to emotion related to narrative socialization of emotion in school-age girls. *Journal of experimental child psychology*, *178*, 155–169. https://doi.org/10.1016/j.jecp.2018.09.015

Lewis, M. D., Granic, I., Lamm, C., Zelazo, P. D., Stieben, J., Todd, R. M., Moadab, I. & Pepler, D. (2008). Changes in the neural bases of emotion regulation associated with clinical improvement in children with behavior problems. *Development and psychopathology*, *20*(3), 913–939. https://doi.org/10.1017/S0954579408000448

Lewis, M. D., Todd, R. M. & Honsberger, M. J. M. (2007). Event-related potential measures of emotion regulation in early childhood. *Neuroreport*, *18*(1), 61–65. https://doi.org/10.1097/WNR.0b013e328010a216

Leyva, D., Reese, E., Laible, D., Schaughency, E., Das, S. & Clifford, A. (2020). Measuring Parents’ Elaborative Reminiscing: Differential Links of Parents’ Elaboration to children’s Autobiographical Memory and Socioemotional Skills. *Journal of Cognition and Development*, *21*(1), 23–45. https://doi.org/10.1080/15248372.2019.1668395

Lieberman, M. D., Eisenberger, N. I., Crockett, M. J., Tom, S. M., Pfeifer, J. H. & Way, B. M. (2007). Putting feelings into words: affect labeling disrupts amygdala activity in response to affective stimuli. *Psychological science*, *18*(5), 421–428. https://doi.org/10.1111/j.1467-9280.2007.01916.x

Lin, Lemery-Chalfant, K., Beekman, C., Crnic, K. A [Keith A.], Gonzales, N. A. & Luecken, L. J. (2021). Infant Temperament Profiles, Cultural Orientation, and Toddler Behavioral and Physiological Regulation in Mexican-American Families. *Child development*, *92*(6), e1110-e1125. https://doi.org/10.1111/cdev.13637

Lin, Pozzi, E., Kehoe, C. E., Havighurst, S., Schwartz, O. S., Yap, M. B. H., Zhao, J., Telzer, E. H. & Whittle, S. (2024). Family and parenting factors are associated with emotion regulation neural function in early adolescent girls with elevated internalizing symptoms. *European child & adolescent psychiatry.* Vorab-Onlinepublikation. https://doi.org/10.1007/s00787-024-02481-z

Lin, B. & Crnic, K. A [K. A.] (2012). Dysregulation coding system. *Unpublished Manual. Arizona State University.*

Lindahl, K. (1998). The Development of Marriage: A 9-Year Perspective. Development. In T. N. Bradbury (Hrsg.), *The Developmental Course of Marital Dysfunction. Cambridge Studies in Social and Emotional Development.* (S. 205–236). Cambridge University Press.

Lisitsa, E., Bolden, C. R., Johnson, B. D. & Mezulis, A. H. (2021). Impact of stress and parenting on respiratory sinus arrythmia trajectories in early adolescence. *Developmental psychobiology*, *63*(6), e22165. https://doi.org/10.1002/dev.22165

Little, C. & Carter, A. S. (2005). Negative emotional reactivity and regulation in 12-month-olds following emotional challenge: Contributions of maternal-infant emotional availability in a low-income sample. *Infant mental health journal*, *26*(4), 354–368. https://doi.org/10.1002/imhj.20055

Liu, Gao, C., Gao, H [Heming] & Liu, W. (2022). The Automatic Emotion Regulation of Children Aged 8-12: An ERP Study. *Frontiers in behavioral neuroscience*, *16*, 921802. https://doi.org/10.3389/fnbeh.2022.921802

Liu, Liu, F., Chen, L., Jiang, Z. & Shang, J. (2019). Cognitive Reappraisal in Children: Neuropsychological Evidence of Up-Regulating Positive Emotion From an ERP Study. *Frontiers in psychology*, *10*, 147. https://doi.org/10.3389/fpsyg.2019.00147

Liu, C., Moore, G. A., Roben, C. K. P., Ganiban, J. M., Leve, L. D., Shaw, D. S [Daniel S.], Natsuaki, M. N., Reiss, D. & Neiderhiser, J. M. (2022). Examining Research Domain Criteria (RDoC) constructs for anger expression and regulation in toddlers. *Journal of psychopathology and clinical science*, *131*(6), 588–597. https://doi.org/10.1037/abn0000658

Lo, S. L., Gearhardt, A. N., Fredericks, E. M., Katz, B., Sturza, J., Kaciroti, N., Gonzalez, R., Hunter, C. M., Sonneville, K., Chaudhry, K., Lumeng, J. C. & Miller, A. L. (2021). Targeted self-regulation interventions in low-income children: Clinical trial results and implications for health behavior change. *Journal of experimental child psychology*, *208*, 105157. https://doi.org/10.1016/j.jecp.2021.105157

Lochman, J. E., Glenn, A. L., Powell, N. P., Boxmeyer, C. L., Bui, C., Kassing, F., Qu, L., Romerro, D. E. & Dishion, T. (2019). Group versus individual format of intervention for aggressive children: Moderators and predictors of outcomes through 4 years after intervention. *Development and psychopathology*, *31*(5), 1757–1775. https://doi.org/10.1017/S0954579419000968

Lozano, E. A. (2005). Reacción de malestar y autorregulación emocional en la infancia. *Psicothema*, *Vol. 17, no 3*, 375–381.

Lugo-Candelas, C., Flegenheimer, C., Harvey, E. & McDermott, J. M. (2017). Neural Correlates of Emotion Reactivity and Regulation in Young Children with ADHD Symptoms. *Journal of abnormal child psychology*, *45*(7), 1311–1324. https://doi.org/10.1007/s10802-017-0297-2

Macari, S. L., Vernetti, A. & Chawarska, K. (2021). Attend Less, Fear More: Elevated Distress to Social Threat in Toddlers With Autism Spectrum Disorder. *Autism research : official journal of the International Society for Autism Research*, *14*(5), 1025–1036. https://doi.org/10.1002/aur.2448

Macklem, G. L. (2008). *Practitioner’s Guide to Emotion Regulation in School-Aged Children*. Springer US. https://doi.org/10.1007/978-0-387-73851-2

Maclean, P. C., Erickson, S. J. & Lowe, J. R. (2009). Comparing emotional reactivity and regulation in infants born ELGA and VLGA. *Infant behavior & development*, *32*(3), 336–339. https://doi.org/10.1016/j.infbeh.2009.02.005

Maclean, P. C., Rynes, K. N., Aragón, C., Caprihan, A., Phillips, J. P. & Lowe, J. R. (2014). Mother-infant mutual eye gaze supports emotion regulation in infancy during the Still-Face paradigm. *Infant behavior & development*, *37*(4), 512–522. https://doi.org/10.1016/j.infbeh.2014.06.008

Malin, J. L [J. L.], Cabrera, N. J [N. J.], Karberg, E [E.], Aldoney, D [D.] & Rowe, M. L. (2014). Low-income, minority fathers’ control strategies and their children’s regulatory skills. *Infant mental health journal*, *35*(5), 462–472. https://doi.org/10.1002/imhj.21467

Mangelsdorf, S. C., Shapiro, J. R. & Marzolf, D. (1995). Developmental and Temperamental Differences in Emotion Regulation in Infancy. *Child development*, *66*(6), 1817. https://doi.org/10.2307/1131912

Manian, N. & Bornstein, M. H [Marc H.] (2009). Dynamics of emotion regulation in infants of clinically depressed and nondepressed mothers. *Journal of child psychology and psychiatry, and allied disciplines*, *50*(11), 1410–1418. https://doi.org/10.1111/j.1469-7610.2009.02166.x

Martin, J. A. (1981). *A longitudinal study of the consequences of early mother interaction: A microanalytic approach* (46(190), 59). Monographs of the Society for Research in Child Development.

Martins, E. C., Soares, I., Martins, C. & Osório, A. (2016). Infants’ Style of Emotion Regulation with Their Mothers and Fathers: Concordance between Parents and the Contribution of Father–Infant Interaction Quality. *Social Development*, *25*(4), 812–827. https://doi.org/10.1111/sode.12171

Martins, E. C., Soares, I., Martins, C., Tereno, S. & Osório, A. (2012). Can We Identify Emotion Over‐regulation in Infancy? Associations with Avoidant Attachment, Dyadic Emotional Interaction and Temperament. *Infant and Child Development*, *21*(6), 579–595. https://doi.org/10.1002/icd.1760

Mascheroni, E., Schiavolin, P., Mariani Wigley, I. L. C., Giorda, R., Pozzoli, U., Morandi, F., Fontana, C., Mosca, F., Fumagalli, M. & Montirosso, R [Rosario] (2022). Serotonin transporter gene methylation and emotional regulation in preschool children born preterm: A longitudinal evaluation of the role of negative emotionality in infancy. *Infant mental health journal*, *43*(4), 589–596. https://doi.org/10.1002/imhj.21990

Matas, L., Arend, R. A. & Sroufe, L. A. (1978). Continuity of Adaptation in the Second Year: The Relationship between Quality of Attachment and Later Competence. *Child development*, *49*(3), 547. https://doi.org/10.2307/1128221

Maughan, A. & Cicchetti, D [Dante] (2002). Impact of child maltreatment and interadult violence on children's emotion regulation abilities and socioemotional adjustment. *Child development*, *73*(5), 1525–1542. https://doi.org/10.1111/1467-8624.00488

Maughan, A., Cicchetti, D [Dante], Toth, S. L. & Rogosch, F. A. (2007). Early-occurring maternal depression and maternal negativity in predicting young children's emotion regulation and socioemotional difficulties. *Journal of abnormal child psychology*, *35*(5), 685–703. https://doi.org/10.1007/s10802-007-9129-0

Mauss, I. B., Cook, C. L. & Gross, J. J. (2007). Automatic emotion regulation during anger provocation. *Journal of Experimental Social Psychology*, *43*(5), 698–711. https://doi.org/10.1016/j.jesp.2006.07.003

Mazefsky, C. A., Day, T. N., Siegel, M., White, S. W., Yu, L. & Pilkonis, P. A. (2018). Development of the Emotion Dysregulation Inventory: A PROMIS®ing Method for Creating Sensitive and Unbiased Questionnaires for Autism Spectrum Disorder. *Journal of autism and developmental disorders*, *48*(11), 3736–3746. https://doi.org/10.1007/s10803-016-2907-1

Mazzer, C. (2023). *Examining the Impact of Standard Parent-Child Interaction Therapy on Children’s Emotion Regulation* [Dissertation].

McClelland, M. M., John Geldhof, G., Cameron, C. E. & Wanless, S. B. (2015). Development and Self‐Regulation. In R. M. Lerner (Hrsg.), *Handbook of Child Psychology and Developmental Science* (S. 1–43). Wiley. https://doi.org/10.1002/9781118963418.childpsy114

McCoy, D. C. & Raver, C. C. (2011). Caregiver Emotional Expressiveness, Child Emotion Regulation, and Child Behavior Problems among Head Start Families. *Social Development*, *20*(4), 741–761. https://doi.org/10.1111/j.1467-9507.2011.00608.x

McCraty, R., Atkinson, M., Tomasino, D., Goelitz, J. & Mayrovitz, H. N. (1999). The impact of an emotional self-management skills course on psychosocial functioning and autonomic recovery to stress in middle school children. *Integrative physiological and behavioral science : the official journal of the Pavlovian Society*, *34*(4), 246–268. https://doi.org/10.1007/BF02688693

McKone, K. M. P., Woody, M. L., Ladouceur, C. D. & Silk, J. S. (2021). Mother-Daughter Mutual Arousal Escalation and Emotion Regulation in Adolescence. *Research on Child and Adolescent Psychopathology*, *49*(5), 615–628. https://doi.org/10.1007/s10802-020-00763-z

McQuade, J. D. & Breaux, R. P. (2017). Are Elevations in ADHD Symptoms Associated with Physiological Reactivity and Emotion Dysregulation in Children? *Journal of abnormal child psychology*, *45*(6), 1091–1103. https://doi.org/10.1007/s10802-016-0227-8

McRae, K., Gross, J. J., Weber, J., Robertson, E. R., Sokol-Hessner, P., Ray, R. D., Gabrieli, J. D. E. & Ochsner, K. N. (2012). The development of emotion regulation: an fMRI study of cognitive reappraisal in children, adolescents and young adults. *Social cognitive and affective neuroscience*, *7*(1), 11–22. https://doi.org/10.1093/scan/nsr093

Mehmood, R. M. & Hyo, J. L. (2016). Toward an analysis of emotion regulation in children using late positive potential. *Annual International Conference of the IEEE Engineering in Medicine and Biology Society. IEEE Engineering in Medicine and Biology Society. Annual International Conference*, *2016*, 279–282. https://doi.org/10.1109/EMBC.2016.7590694

Melnick, S. M. & Hinshaw, S. P. (2000). Emotion regulation and parenting in AD/HD and comparison boys: linkages with social behaviors and peer preference. *Journal of abnormal child psychology*, *28*(1), 73–86. https://doi.org/10.1023/A:1005174102794

Meroño, G. & Ventura, A. C. (2022). Estrategias de regulación emocional de niños/as en el aprendizaje de la escritura en situación de clase y de entrevista. *Interdisciplinaria. Revista de Psicología y Ciencias Afines*, *39*(3). https://doi.org/10.16888/interd.2022.39.3.12

Mesman, J., van IJzendoorn, M. H. & Bakermans-Kranenburg, M. J. (2009). The many faces of the Still-Face Paradigm: A review and meta-analysis. *Developmental Review*, *29*(2), 120–162. https://doi.org/10.1016/j.dr.2009.02.001

Mezulis, A. H., Crystal, S. I., Ahles, J. J. & Crowell, S. E. (2015). Examining biological vulnerability in environmental context: Parenting moderates effects of low resting respiratory sinus arrhythmia on adolescent depressive symptoms. *Developmental psychobiology*, *57*(8), 974–983. https://doi.org/10.1002/dev.21347

Miller, A. L., Kiely Gouley, K., Seifer, R., Dickstein, S. & Shields, A. (2004). Emotions and Behaviors in the Head Start Classroom: Associations Among Observed Dysregulation, Social Competence, and Preschool Adjustment. *Early education and development*, *15*(2), 147–166. https://doi.org/10.1207/s15566935eed1502_2

Miller, A. L., Volling, B. L. & McElwain, N. L. (2000). Sibling Jealousy in a Triadic Context with Mothers and Fathers. *Social Development*, *9*(4), 433–457. https://doi.org/10.1111/1467-9507.00137

Millman, T. P. (2007). Infant Self-Regulation Scheme (ISRS). *Unpublished undergraduate thesis, Concordia University, Montreal, Canada.*

Mills, A. S., Tablon-Modica, P., Mazefksy, C. A. & Weiss, J. A. (2022). Emotion dysregulation in children with autism: A multimethod investigation of the role of child and parent factors. *Research in autism spectrum disorders*, *91*, 101911. https://doi.org/10.1016/j.rasd.2021.101911

Mirabile, S. P., Scaramella, L. V., Sohr-Preston, S. L. & Robison, S. D. (2009). Mothers' Socialization of Emotion Regulation: The Moderating Role of Children's Negative Emotional Reactivity. *Child & youth care forum*, *38*(1), 19–37. https://doi.org/10.1007/s10566-008-9063-5

Miyake, A. (2001). How are visuospatial working memory, executive functioning, and spatial abilities related? A latent-variable analysis. *Journal of experimental psychology: General*(130(4), 621.

Moffitt, J. M., Baker, J. K., Fenning, R. M., Erath, S. A., Messinger, D. S., Zeedyk, S. M., Paez, S. A. & Seel, S. (2021). Parental Socialization of Emotion and Psychophysiological Arousal Patterns in Children with Autism Spectrum Disorder. *Research on Child and Adolescent Psychopathology*, *49*(3), 401–412. https://doi.org/10.1007/s10802-020-00745-1

Molitor, A., Mayes, L. C. & Ward, A. (2003). Emotion regulation behavior during a separation procedure in 18-month-old children of mothers using cocaine and other drugs. *Development and psychopathology*, *15*(1), 39–54. https://doi.org/10.1017/S0954579403000038

Morales, M. & Bridges, L. J. (1996). Associations between nonparental care experience and preschooler's emotion regulation in the presence of the mother. *Journal of Applied Developmental Psychology*, *17*(4), 577–596. https://doi.org/10.1016/S0193-3973(96)90017-9

Morales, M., Mundy, P., Crowson, M., Neal, A. R. & Delgado, C. (2005). Individual differences in infant attention skills, joint attention, and emotion regulation behaviour. *International Journal of Behavioral Development*, *29*(3), 259–263. https://doi.org/10.1080/01650250444000432

Morris, A. S., Silk, J. S., Morris, M. D. S., Steinberg, L., Aucoin, K. J. & Keyes, A. W. (2011). The influence of mother-child emotion regulation strategies on children's expression of anger and sadness. *Developmental psychology*, *47*(1), 213–225. https://doi.org/10.1037/a0021021

Morris, A. S., Silk, J. S., Steinberg, L., Myers, S. S. & Robinson, L. R. (2007). The Role of the Family Context in the Development of Emotion Regulation. *Social Development*, *16*(2), 361–388. https://doi.org/10.1111/j.1467-9507.2007.00389.x

Morris, A. S., Silk, J. S., Steinberg, L., Terranova, A. M. & Kithakye, M. (2010). Concurrent and Longitudinal Links Between Children’s Externalizing Behavior in School and Observed Anger Regulation in the Mother–Child Dyad. *Journal of Psychopathology and Behavioral Assessment*, *32*(1), 48–56. https://doi.org/10.1007/s10862-009-9166-9

Mortaji, N., Krzeczkowski, J., Atkinson, S., Amani, B., Schmidt, L. A. & van Lieshout, R. (2023). Preliminary findings of emotion regulation in 12-month-old infants of mothers enrolled in a randomized controlled trial assessing a nutrition + exercise intervention. *Developmental psychobiology*, *65*(2), e22376. https://doi.org/10.1002/dev.22376

Mortensen, J. A. & Barnett, M. A. (2018). Emotion Regulation, Harsh Parenting, and Teacher Sensitivity Among Socioeconomically Disadvantaged Toddlers in Child Care. *Early education and development*, *29*(2), 143–160. https://doi.org/10.1080/10409289.2017.1371560

Mortensen, J. A. & Barnett, M. A. (2019). Intrusive parenting, teacher sensitivity, and negative emotionality on the development of emotion regulation in early head start toddlers. *Infant behavior & development*, *55*, 10–21. https://doi.org/10.1016/j.infbeh.2019.01.004

Murnan, A. W., Keim, S. A., Yeates, K. O., Boone, K. M., Sheppard, K. W. & Klebanoff, M. A. (2021). Behavioral and Cognitive Differences in Early Childhood related to Prenatal Marijuana Exposure. *Journal of Applied Developmental Psychology*, *77.* https://doi.org/10.1016/j.appdev.2021.101348

Murphy, E. R., Barch, D. M., Pagliaccio, D., Luby, J. L. & Belden, A. C [Andy C.] (2016). Functional connectivity of the amygdala and subgenual cingulate during cognitive reappraisal of emotions in children with MDD history is associated with rumination. *Developmental cognitive neuroscience*, *18*, 89–100. https://doi.org/10.1016/j.dcn.2015.11.003

Musser, E. D., Galloway-Long, H. S., Frick, P. J. & Nigg, J. T. (2013). Emotion regulation and heterogeneity in attention-deficit/hyperactivity disorder. *Journal of the American Academy of Child and Adolescent Psychiatry*, *52*(2), 163-171.e2. https://doi.org/10.1016/j.jaac.2012.11.009

Musser, E. D., Lugo, Y., Ward, A. R., Tenenbaum, R. B., Morris, S., Brijmohan, N. & Martinez, J. (2018). Parent Emotion Expression and Autonomic-Linked Emotion Dysregulation in Childhood ADHD. *Journal of Psychopathology and Behavioral Assessment*, *40*(4), 593–605. https://doi.org/10.1007/s10862-018-9685-3

Myruski, S., Bagrodia, R. & Dennis-Tiwary, T. (2022). Delta-beta correlation predicts adaptive child emotion regulation concurrently and two years later. *Biological psychology*, *167*, 108225. https://doi.org/10.1016/j.biopsycho.2021.108225

Myruski, S., Birk, S., Karasawa, M., Kamikubo, A., Kazama, M., Hirabayashi, H. & Dennis-Tiwary, T. (2019). Neural signatures of child cognitive emotion regulation are bolstered by parental social regulation in two cultures. *Social cognitive and affective neuroscience*, *14*(9), 947–956. https://doi.org/10.1093/scan/nsz070

Myruski, S. & Dennis-Tiwary, T. (2021). Biological signatures of emotion regulation flexibility in children: Parenting context and links with child adjustment. *Cognitive, affective & behavioral neuroscience*, *21*(4), 805–821. https://doi.org/10.3758/s13415-021-00888-8

Nakamichi, K. (2017). Differences in Young Children's Peer Preference by Inhibitory Control and Emotion Regulation. *Psychological reports*, *120*(5), 805–823. https://doi.org/10.1177/0033294117709260

Neault, I. (2015). Le développement de la régulation des émotions chez des nourrissons de mères adolescents. *Enfance*, *2015/2 N° 2*, 179–198.

Nelson, J. A., O'Brien, M., Calkins, S. D., Leerkes, E. M., Marcovitch, S. & Blankson, A. N. (2012). Maternal Expressive Style and Children's Emotional Development. *Infant and Child Development*, *21*(3), 267–286. https://doi.org/10.1002/icd.748

Neuhaus, E., Bernier, R. & Beauchaine, T. P. (2014). Brief report: social skills, internalizing and externalizing symptoms, and respiratory sinus arrhythmia in autism. *Journal of autism and developmental disorders*, *44*(3), 730–737. https://doi.org/10.1007/s10803-013-1923-7

Nigg, J. T. (2017). Annual Research Review: On the relations among self-regulation, self-control, executive functioning, effortful control, cognitive control, impulsivity, risk-taking, and inhibition for developmental psychopathology. *Journal of child psychology and psychiatry, and allied disciplines*, *58*(4), 361–383. https://doi.org/10.1111/jcpp.12675

Norona & Baker, B. L [Bruce L.] (2014). The transactional relationship between parenting and emotion regulation in children with or without developmental delays. *Research in developmental disabilities*, *35*(12), 3209–3216. https://doi.org/10.1016/j.ridd.2014.07.048

Norona, A. N. & Baker, B. L [B. L.] (2017). The effects of early positive parenting and developmental delay status on child emotion dysregulation. *Journal of intellectual disability research : JIDR*, *61*(2), 130–143. https://doi.org/10.1111/jir.12287

Noroña, A. N., Tung, I., Lee, S. S., Blacher, J., Crnic, K. A [Keith A.] & Baker, B. L [Bruce L.] (2018). Developmental Patterns of Child Emotion Dysregulation as Predicted by Serotonin Transporter Genotype and Parenting. *Journal of clinical child and adolescent psychology : the official journal for the Society of Clinical Child and Adolescent Psychology, American Psychological Association, Division 53*, *47*(sup1), S354-S368. https://doi.org/10.1080/15374416.2017.1326120

Nowak, A. L. (2020). *TRAJECTORIES OF MATERNAL SUPPORTIVENESS DURING TODDLERHOOD DIFFERENTIALLY PREDICTING CHILD OUTCOMES* [Dissertation].

Ntourou, K., Conture, E. G. & Walden, T. A. (2013). Emotional reactivity and regulation in preschool-age children who stutter. *Journal of fluency disorders*, *38*(3), 10.1111/infa.12101260–274. https://doi.org/10.1016/j.jfludis.2013.06.002

Nuske, H. J., Hedley, D., Tseng, C. H., Begeer, S. & Dissanayake, C. (2018). Emotion Regulation Strategies in Preschoolers with Autism: Associations with Parent Quality of Life and Family Functioning. *Journal of autism and developmental disorders*, *48*(4), 1287–1300. https://doi.org/10.1007/s10803-017-3391-y

Ochsner, K. N., Ray, R. D., Cooper, J. C., Robertson, E. R., Chopra, S., Gabrieli, J. D. E. & Gross, J. J. (2004). For better or for worse: neural systems supporting the cognitive down- and up-regulation of negative emotion. *NeuroImage*, *23*(2), 483–499. https://doi.org/10.1016/j.neuroimage.2004.06.030

Oeri, N. & Roebers, C. M. (2020). Regulating disappointment can impair cognitive performance in kindergarten children: Individual differences in ego depletion. *Journal of experimental child psychology*, *190*, 104728. https://doi.org/10.1016/j.jecp.2019.104728

Onchwari, G. & Keengwe, J. (2011). Examining the Relationship of Children’s Behavior to Emotion Regulation Ability. *Early Childhood Education Journal*, *39*(4), 279–284. https://doi.org/10.1007/s10643-011-0466-9

Osborne, K., Duprey, E., Caughy, M. O. & Oshri, A. (2021). Parents' Maltreatment Histories, Dimensions of Emotion Regulation, and Connections to Offspring Self-Regulation: A Sex-Specific Transmission Pathway. *Journal of Psychopathology and Behavioral Assessment*, *43*(4), 717–729. https://doi.org/10.1007/s10862-021-09881-4

Ostlund, B. D., Measelle, J. R., Laurent, H. K., Conradt, E. & Ablow, J. C. (2017). Shaping emotion regulation: attunement, symptomatology, and stress recovery within mother-infant dyads. *Developmental psychobiology*, *59*(1), 15–25. https://doi.org/10.1002/dev.21448

Pagliaccio, D., Luby, J. L., Luking, K. R [Katherine R.], Belden, A. C [Andrew C.] & Barch, D. M. (2014). Brain-behavior relationships in the experience and regulation of negative emotion in healthy children: implications for risk for childhood depression. *Development and psychopathology*, *26*(4 Pt 2), 1289–1303. https://doi.org/10.1017/S0954579414001035

Pang, K. C. & Beauchaine, T. P. (2013). Longitudinal patterns of autonomic nervous system responding to emotion evocation among children with conduct problems and/or depression. *Developmental psychobiology*, *55*(7), 698–706. https://doi.org/10.1002/dev.21065

Penela, E. C., Walker, O. L., Degnan, K. A., Fox, N. A. & Henderson, H. A. (2015). Early Behavioral Inhibition and Emotion Regulation: Pathways Toward Social Competence in Middle Childhood. *Child development*, *86*(4), 1227–1240. https://doi.org/10.1111/cdev.12384

Perez-Edgar, K. & Fox, N. A. (2005). A Behavioral and Electrophysiological Study of Children's Selective Attention Under Neutral and Affective Conditions. *Journal of Cognition and Development*, *6*(1), 89–118. https://doi.org/10.1207/s15327647jcd0601_6

Perlman, S. B., Jones, B. M., Wakschlag, L. S., Axelson, D., Birmaher, B. & Phillips, M. L. (2015). Neural substrates of child irritability in typically developing and psychiatric populations. *Developmental cognitive neuroscience*, *14*, 71–80. https://doi.org/10.1016/j.dcn.2015.07.003

Perlman, S. B. & Pelphrey, K. A. (2010). Regulatory brain development: balancing emotion and cognition. *Social neuroscience*, *5*(5-6), 533–542. https://doi.org/10.1080/17470911003683219

Perlman, S. B. & Pelphrey, K. A. (2011). Developing connections for affective regulation: age-related changes in emotional brain connectivity. *Journal of experimental child psychology*, *108*(3), 607–620. https://doi.org/10.1016/j.jecp.2010.08.006

Perone, S., Gartstein, M. A. & Anderson, A. J. (2020). Dynamics of frontal alpha asymmetry in mother-infant dyads: Insights from the Still Face Paradigm. *Infant behavior & development*, *61*, 101500. https://doi.org/10.1016/j.infbeh.2020.101500

Perry, N. B., Calkins, S. D. & Bell, M. A. (2016). Indirect Effects of Maternal Sensitivity on Infant Emotion Regulation Behaviors: The Role of Vagal Withdrawal. *Infancy : the official journal of the International Society on Infant Studies*, *21*(2), 128–153. https://doi.org/10.1111/infa.12101

Perry, N. B., Calkins, S. D., Dollar, J. M., Keane, S. P [Susan P.] & Shanahan, L. (2018). Self-regulation as a predictor of patterns of change in externalizing behaviors from infancy to adolescence. *Development and psychopathology*, *30*(2), 497–510. https://doi.org/10.1017/S0954579417000992

Perry, N. B., Calkins, S. D., Nelson, J. A., Leerkes, E. M. & Marcovitch, S. (2012). Mothers' responses to children's negative emotions and child emotion regulation: the moderating role of vagal suppression. *Developmental psychobiology*, *54*(5), 503–513. https://doi.org/10.1002/dev.20608

Perry, N. B., Dollar, J. M., Calkins, S. D., Keane, S. P [Susan P.] & Shanahan, L. (2018). Childhood self-regulation as a mechanism through which early overcontrolling parenting is associated with adjustment in preadolescence. *Developmental psychology*, *54*(8), 1542–1554. https://doi.org/10.1037/dev0000536

Perry, N. B., Dollar, J. M., Calkins, S. D., Keane, S. P [Susan P.] & Shanahan, L. (2020). Maternal socialization of child emotion and adolescent adjustment: Indirect effects through emotion regulation. *Developmental psychology*, *56*(3), 541–552. https://doi.org/10.1037/dev0000815

Perry, N. B., Swingler, M. M., Calkins, S. D. & Bell, M. A. (2016). Neurophysiological correlates of attention behavior in early infancy: Implications for emotion regulation during early childhood. *Journal of experimental child psychology*, *142*, 245–261. https://doi.org/10.1016/j.jecp.2015.08.007

Petersen, H. & Holodynski, M. (2020). Bewitched to Be Happy? The Impact of Pretend Play on Emotion Regulation of Expression in 3- to 6-Year-Olds. *The Journal of genetic psychology*, *181*(2-3), 111–126. https://doi.org/10.1080/00221325.2020.1734909

Poon, J. A., Thompson, J. C. & Chaplin, T. M. (2022). Task-based functional connectivity patterns: Links to adolescent emotion regulation and psychopathology. *Journal of affective disorders*, *302*, 33–40. https://doi.org/10.1016/j.jad.2022.01.092

Porges (2001). The polyvagal theory: phylogenetic substrates of a social nervous system. *International journal of psychophysiology : official journal of the International Organization of Psychophysiology*, *42*(2), 123–146. https://doi.org/10.1016/s0167-8760(01)00162-3

Porges (2006). The Polyvagal Perspective. *Biological psychology*, *74*(2), 116–143. https://doi.org/10.1016/j.biopsycho.2006.06.009

Porter, C. L., Stockdale, L. A., Reschke, P., Booth, M., Memmott-Elison, M. K. & Coyne, S. M. (2022). "Katerina gets mad": Infants' physiological and behavioral responses to co-viewing educational, self-regulatory media. *Developmental psychobiology*, *64*(8), e22337. https://doi.org/10.1002/dev.22337

Posner, M. I., Rothbart, M. K [Mary K.], Sheese, B. E. & Tang, Y [Yiyuan] (2007). The anterior cingulate gyrus and the mechanism of self-regulation. *Cognitive, affective & behavioral neuroscience*, *7*(4), 391–395. https://doi.org/10.3758/CABN.7.4.391

Power, T. G., Olivera, Y. A., Hill, R. A., Beck, A. D., Hopwood, V., Garcia, K. S., Ramos, G. G., Fisher, J. O., O'Connor, T. M. & Hughes, S. O. (2016). Emotion regulation strategies and childhood obesity in high risk preschoolers. *Appetite*, *107*, 623–627. https://doi.org/10.1016/j.appet.2016.09.008

Premo, J. E. & Kiel, E. J. (2014). The effect of toddler emotion regulation on maternal emotion socialization: Moderation by toddler gender. *Emotion (Washington, D.C.)*, *14*(4), 782–793. https://doi.org/10.1037/a0036684

Premo, J. E. & Kiel, E. J. (2016). Maternal depressive symptoms, toddler emotion regulation, and subsequent emotion socialization. *Journal of Family Psychology*, *30*(2), 276–285. https://doi.org/10.1037/fam0000165

Price, N. N. & Kiel, E. J. (2022). Longitudinal Links among Mother and Child Emotion Regulation, Maternal Emotion Socialization, and Child Anxiety. *Research on Child and Adolescent Psychopathology*, *50*(2), 241–254. https://doi.org/10.1007/s10802-021-00804-1

Provenzi, L., Fumagalli, M., Di Scotto Minico, G., Giorda, R., Morandi, F., Sirgiovanni, I., Schiavolin, P., Mosca, F., Borgatti, R. & Montirosso, R [Rosario] (2020). Pain-related increase in serotonin transporter gene methylation associates with emotional regulation in 4.5-year-old preterm-born children. *Acta paediatrica (Oslo, Norway : 1992)*, *109*(6), 1166–1174. https://doi.org/10.1111/apa.15077

Pruett, D. G., Porges, S. W., Walden, T. A. & Jones, R. M. (2023). A study of respiratory sinus arrhythmia and stuttering persistence. *Journal of communication disorders*, *102*, 106304. https://doi.org/10.1016/j.jcomdis.2023.106304

Puglisi, N., Tissot, H., Rattaz, V., Epiney, M., Razurel, C. & Favez, N. (2023). Father-infant synchrony and infant vagal tone as an index of emotion regulation: father-infant shared times in Switzerland as moderators. *Early Child Development and Care*, *193*(15-16), 1714–1727. https://doi.org/10.1080/03004430.2023.2274287

Qu, J., Leerkes, E. M. & King, E. K. (2016). Preschoolers' distress and regulatory behaviors vary as a function of infant-mother attachment security. *Infant behavior & development*, *44*, 144–147. https://doi.org/10.1016/j.infbeh.2016.06.008

Ratcliff, K. A [K. Ashana], Vazquez, L. C., Lunkenheimer, E. S. & Cole, P. M. (2021). Longitudinal changes in young children's strategy use for emotion regulation. *Developmental psychology*, *57*(9), 1471–1486. https://doi.org/10.1037/dev0001235

Rattaz, V., Tissot, H., Puglisi, N., Razurel, C., Epiney, M. & Favez, N. (2023). Parental sensitivity, family alliance and infants' vagal tone: Influences of early family interactions on physiological emotion regulation. *Infant mental health journal*, *44*(6), 741–751. https://doi.org/10.1002/imhj.22085

Reck, C. (2009). Infant and caregiver engagement phases (icep)-revised version. *Unpublished manuscript, Heidelberg, Department of General Psychiatry.*

Reijntjes, A., Stegge, H., Terwogt, M. M., Kamphuis, J. H. & Telch, M. J. (2006). Emotion regulation and its effects on mood improvement in response to an in vivo peer rejection challenge. *Emotion (Washington, D.C.)*, *6*(4), 543–552. https://doi.org/10.1037/1528-3542.6.4.543

Reis, A. H., Oliveira, S. E. S., Bandeira, D. R., Andrade, N. C., Abreu, N. & Sperb, T. M. (2016). Emotion regulation checklist (ERC): preliminary studies of cross-cultural adaptation and validation for use in Brazil. *Temas em Psicologia*, *24*(1), 97–116. https://doi.org/10.9788/TP2016.1-07

Ren, Y., Wyver, S., Xu Rattanasone, N. & Demuth, K. (2016). Social Competence and Language Skills in Mandarin–English Bilingual Preschoolers: The Moderation Effect of Emotion Regulation. *Early education and development*, *27*(3), 303–317. https://doi.org/10.1080/10409289.2015.1066639

Ren, Y., Xu Rattanasone, N., Demuth, K., Andronos, F. & Wyver, S. (2018). Relationships between proficiency with grammatical morphemes and emotion regulation: a study of Mandarin–English preschoolers. *Early Child Development and Care*, *188*(8), 1055–1062. https://doi.org/10.1080/03004430.2016.1245189

Richardson, P. A., Bocknek, E. L., McGoron, L. & Trentacosta, C. J. (2019). Fathering across contexts: The moderating role of respiratory sinus arrhythmia in predicting toddler emotion regulation. *Developmental psychobiology*, *61*(6), 903–919. https://doi.org/10.1002/dev.21836

Rime, J., Tissot, H., Favez, N., Watson, M. & Stadlmayr, W. (2018). The Diaper Change Play: Validation of a New Observational Assessment Tool for Early Triadic Family Interactions in the First Month Postpartum. *Frontiers in psychology*, *9*, 497. https://doi.org/10.3389/fpsyg.2018.00497

Riva Crugnola, C., Ierardi, E., Bottini, M., Verganti, C. & Albizzati, A. (2019). Childhood experiences of maltreatment, reflective functioning and attachment in adolescent and young adult mothers: Effects on mother-infant interaction and emotion regulation. *Child abuse & neglect*, *93*, 277–290. https://doi.org/10.1016/j.chiabu.2019.03.024

Riva Crugnola, C., Tambelli, R., Spinelli, M., Gazzotti, S., Caprin, C. & Albizzati, A. (2011). Attachment patterns and emotion regulation strategies in the second year. *Infant behavior & development*, *34*(1), 136–151. https://doi.org/10.1016/j.infbeh.2010.11.002

Rnic, K., Jopling, E., Tracy, A. & LeMoult, J. (2022). Emotion regulation and diurnal cortisol: A longitudinal study of early adolescents. *Biological psychology*, *167*, 108212. https://doi.org/10.1016/j.biopsycho.2021.108212

Roben, C. K. P., Cole, P. M. & Armstrong, L. M. (2013). Longitudinal relations among language skills, anger expression, and regulatory strategies in early childhood. *Child development*, *84*(3), 891–905. https://doi.org/10.1111/cdev.12027

Rodríguez, G. M., Bagner, D. M. & Graziano, P. A. (2014). Parent training for children born premature: a pilot study examining the moderating role of emotion regulation. *Child psychiatry and human development*, *45*(2), 143–152. https://doi.org/10.1007/s10578-013-0385-7

Rohlf, H. L. & Krahé, B. (2015). Assessing anger regulation in middle childhood: development and validation of a behavioral observation measure. *Frontiers in psychology*, *6*, 453. https://doi.org/10.3389/fpsyg.2015.00453

Roid, G. H. & Miller, L. J. (1997). Leiter international performance scale-revised (Leiter-R). *Wood Dale, IL: Stoelting, 10(10.1037).*

Root, A. E., Byrne, R. & Watson, S. M. (2015). The regulation of fear: the contribution of inhibition and emotion socialisation. *Early Child Development and Care*, *185*(4), 647–657. https://doi.org/10.1080/03004430.2014.946503

Roque, L., Veríssimo, M., Fernandes, M [Marília] & Rebelo, A. (2013). Emotion regulation and attachment: relationships with children's secure base, during different situational and social contexts in naturalistic settings. *Infant behavior & development*, *36*(3), 298–306. https://doi.org/10.1016/j.infbeh.2013.03.003

Ros, R. & Graziano, P. A. (2020). A Transdiagnostic Examination of Self-Regulation: Comparisons Across Preschoolers with ASD, ADHD, and Typically Developing Children. *Journal of clinical child and adolescent psychology : the official journal for the Society of Clinical Child and Adolescent Psychology, American Psychological Association, Division 53*, *49*(4), 493–508. https://doi.org/10.1080/15374416.2019.1591280

Rothbart & Posner, M. I. (1985). Temperament and the Development of Self-Regulation. In L. C. Hartlage & C. F. Telzrow (Hrsg.), *The Neuropsychology of Individual Differences* (S. 93–123). Springer US. https://doi.org/10.1007/978-1-4899-3484-0_5

Rothbart, M. K [M. K.]. (1989). Temperament in childhood: A framework. In G. A. Kohnstamm, J. E. Bates & M. K. Rothbart (Hrsg.), *Temperament in childhood* (S. 59–73). John Wiley & Sons.

Rothbart. & Derryberry, D. (1981). Development of Individual Difference in Temperament. In Lamb, E. A., Brown & A. L. (Hrsg.), *Advances in Developmental Psychology Volume 1* (1st Edition, S. 37–86).

Roy, A. K., Klein, R. G., Angelosante, A., Bar-Haim, Y., Leibenluft, E., Hulvershorn, L., Dixon, E., Dodds, A. & Spindel, C. (2013). Clinical features of young children referred for impairing temper outbursts. *Journal of child and adolescent psychopharmacology*, *23*(9), 588–596. https://doi.org/10.1089/cap.2013.0005

Rueda, M. R., Fan, J., McCandliss, B. D., Halparin, J. D., Gruber, D. B., Lercari, L. P. & Posner, M. I. (2004). Development of attentional networks in childhood. *Neuropsychologia*, *42*(8), 1029–1040. https://doi.org/10.1016/j.neuropsychologia.2003.12.012

Saarni (Hrsg.). (1999). *The development of emotional competence*. New York: Guilford.

Saarni, C. (1984). An Observational Study of Children's Attempts to Monitor Their Expressive Behavior. *Child development*, *55*(4), 1504. https://doi.org/10.2307/1130020

Sameroff, A. (2009). *The transactional model of development: How children and contexts shape each other*. American Psychological Association. https://doi.org/10.1037/11877-000

Santana, M. & Grabell, A. S. (2023). Incongruent affect in early childhood: Neurobiological markers and links to psychopathology. *Emotion (Washington, D.C.)*, *23*(6), 1562–1574. https://doi.org/10.1037/emo0001180

Santucci, A. K., Silk, J. S., Shaw, D. S [Daniel S.], Gentzler, A., Fox, N. A. & Kovacs, M. (2008). Vagal tone and temperament as predictors of emotion regulation strategies in young children. *Developmental psychobiology*, *50*(3), 205–216. https://doi.org/10.1002/dev.20283

Schatz, J. N., Smith, L. E., Borkowski, J. G., Whitman, T. L. & Keogh, D. A. (2008). Maltreatment risk, self-regulation, and maladjustment in at-risk children. *Child abuse & neglect*, *32*(10), 972–982. https://doi.org/10.1016/j.chiabu.2008.09.001

Scherhag, J. & Burgard, T. (2023). *Performance of Semi-Automated Screening Using Rayyan and ASReview: A Retrospective Analysis of Potential Work Reduction and Different Stopping Rules*. ZPID (Leibniz Institute for Psychology). https://psycharchives.org/en/item/cb2c6618-0acf-4a5a-976d-23f1d54ecd8e

Schoppmann, J., Schneider, S. & Seehagen, S. (2019). Wait and See: Observational Learning of Distraction as an Emotion Regulation Strategy in 22-Month-Old Toddlers. *Journal of abnormal child psychology*, *47*(5), 851–863. https://doi.org/10.1007/s10802-018-0486-7

Schoppmann, J., Schneider, S. & Seehagen, S. (2022). Can you teach me not to be angry? Relations between temperament and the emotion regulation strategy distraction in 2-year-olds. *Child development*, *93*(1), 165–179. https://doi.org/10.1111/cdev.13682

Schoppmann, J., Severin, F., Schneider, S. & Seehagen, S. (2023). The effect of picture book reading on young children's use of an emotion regulation strategy. *PloS one*, *18*(8), e0289403. https://doi.org/10.1371/journal.pone.0289403

Scrimgeour, M. B., Davis, E. L. & Buss, K. A. (2016). You get what you get and you don't throw a fit! Emotion socialization and child physiology jointly predict early prosocial development. *Developmental psychology*, *52*(1), 102–116. https://doi.org/10.1037/dev0000071

Senehi, N. & Brophy-Herb, H. E. (2020). Role of maternal affect and regulatory strategies in toddlers' emotion and behavior regulation. *Infant behavior & development*, *60*, 101472. https://doi.org/10.1016/j.infbeh.2020.101472

Shapiro, B., Fagen, J., Prigot, J., Carroll, M. & Shalan, J. (1998). Infants' emotional and regulatory behaviors in response to violations of expectancies. *Infant Behavior and Development*, *21*(2), 299–313. https://doi.org/10.1016/S0163-6383(98)90008-X

Shields, A. M., Cicchetti, D [D.] & Ryan, R. M. (1994). The development of emotional and behavioral self-regulation and social competence among maltreated school-age children. *Development and psychopathology*, *6*(1), 57–75. https://doi.org/10.1017/S0954579400005885

Shields. & Cicchetti, D [D.] (1998). Reactive aggression among maltreated children: the contributions of attention and emotion dysregulation. *Journal of clinical child psychology*, *27*(4), 381–395. https://doi.org/10.1207/s15374424jccp2704_2

Shih, E. W., Quiñones-Camacho, L. E. & Davis, E. L. (2018). Parent emotion regulation socializes children's adaptive physiological regulation. *Developmental psychobiology*, *60*(5), 615–623. https://doi.org/10.1002/dev.21621

Shonkoff, J. P. (2000). *From Neurons to Neighborhoods: The Science of Early Childhood Development.: Acquiring Self-Regulation.* National Research Council (US) and Institute of Medicine (US) Committee on Integrating the Science of Early Childhood Development. https://www.ncbi.nlm.nih.gov/books/NBK225568/

Silk, J. S., Shaw, D. S [Daniel S.], Forbes, E. E., Lane, T. L. & Kovacs, M. (2006). Maternal depression and child internalizing: the moderating role of child emotion regulation. *Journal of clinical child and adolescent psychology : the official journal for the Society of Clinical Child and Adolescent Psychology, American Psychological Association, Division 53*, *35*(1), 116–126. https://doi.org/10.1207/s15374424jccp3501_10

Silk, J. S., Shaw, D. S [Daniel S.], Skuban, E. M [Emily M.], Oland, A. A. & Kovacs, M. (2006). Emotion regulation strategies in offspring of childhood-onset depressed mothers. *Journal of child psychology and psychiatry, and allied disciplines*, *47*(1), 69–78. https://doi.org/10.1111/j.1469-7610.2005.01440.x

Silk, J. S., Stroud, L. R., Siegle, G. J., Dahl, R. E., Lee, K. H. & Nelson, E. E. (2012). Peer acceptance and rejection through the eyes of youth: pupillary, eyetracking and ecological data from the Chatroom Interact task. *Social cognitive and affective neuroscience*, *7*(1), 93–105. https://doi.org/10.1093/scan/nsr044

Silkenbeumer, J. R., Schiller, E.‑M. & Kärtner, J. (2018). Co- and self-regulation of emotions in the preschool setting. *Early childhood research quarterly*, *44*, 72–81. https://doi.org/10.1016/j.ecresq.2018.02.014

Skibo, M. A., Sturge-Apple, M. L. & Suor, J. H. (2020). Early experiences of insensitive caregiving and children's self-regulation: Vagal tone as a differential susceptibility factor. *Development and psychopathology*, *32*(4), 1460–1472. https://doi.org/10.1017/S0954579419001408

Smith, M [Maureen] (2001). Social and Emotional Competencies: Contributions to Young African-American Children's Peer Acceptance. *Early education and development*, *12*(1), 49–72. https://doi.org/10.1207/s15566935eed1201_4

Smith., Calkins, S. D. & Keane, S. P [Susan P.] (2006). The Relation of Maternal Behavior and Attachment Security to Toddlers' Emotions and Emotion Regulation. *Research in Human Development*, *3*(1), 21–31. https://doi.org/10.1207/s15427617rhd0301_3

Smith., Hastings, P. D [Paul D.], Henderson, H. A. & Rubin, K. H. (2019). Multidimensional Emotion Regulation Moderates the Relation Between Behavioral Inhibition at Age 2 and Social Reticence with Unfamiliar Peers at Age 4. *Journal of abnormal child psychology*, *47*(7), 1239–1251. https://doi.org/10.1007/s10802-018-00509-y

Smith-Donald, R., Raver, C. C., Hayes, T. & Richardson, B. (2007). Preliminary construct and concurrent validity of the Preschool Self-regulation Assessment (PSRA) for field-based research. *Early childhood research quarterly*, *22*(2), 173–187. https://doi.org/10.1016/j.ecresq.2007.01.002

Snyder, M. C. & Arnold, H. S. (2022). Emotion-related regulation strategy use in preschool-age children who stutter. *Journal of communication disorders*, *97*, 106219. https://doi.org/10.1016/j.jcomdis.2022.106219

Somers, J. A., Borelli, J. L., Smiley, P. A., West, J. L. & Hilt, L. M. (2015). Concurrent and Prospective Associations Between Emotion Reactivity and Depressive Symptoms in Middle Childhood. *Journal of Psychopathology and Behavioral Assessment*, *37*(4), 692–704. https://doi.org/10.1007/s10862-015-9491-0

Song, J.‑H., Colasante, T. & Malti, T. (2018). Helping yourself helps others: Linking children's emotion regulation to prosocial behavior through sympathy and trust. *Emotion (Washington, D.C.)*, *18*(4), 518–527. https://doi.org/10.1037/emo0000332

Soussignan, R., Boivin, M., Girard, A., Pérusse, D., Liu, X [Xuecheng] & Tremblay, R. E. (2009). Genetic and environmental etiology of emotional and social behaviors in 5-month-old infant twins: influence of the social context. *Infant behavior & development*, *32*(1), 1–9. https://doi.org/10.1016/j.infbeh.2008.09.002

Spang, K. S [Katrine Søborg], Hagstrøm, J., Ellersgaard, D., Christiani, C., Hemager, N., Burton, B. K., Greve, A. N., Rohr, K., Gantriis, D., Vangkilde, S., Mors, O., Nordentoft, M., Obel, C., Plessen, K. J., Jepsen, J. R. M. & Thorup, A. A. E. (2022). Emotion regulation in 7-year-old children with familial high risk for schizophrenia or bipolar disorder compared to controls - The Danish High Risk and Resilience Study - VIA 7, a population-based cohort study. *The British journal of clinical psychology*, *61*(4), 1103–1118. https://doi.org/10.1111/bjc.12382

Spangler & Zimmermann, P. (2014). Emotional and adrenocortical regulation in early adolescence. *International Journal of Behavioral Development*, *38*(2), 142–154. https://doi.org/10.1177/0165025414520808

Speranza, A. M. (2002). Il gioco e le strategie di regolazione affettiva nella prima infanzia. *Psicologia Clinica dello Sviluppo*, *Volume 6, Issue 1*, 95–118.

Spinrad, T. L [Tracy L.], Stifter, C. A., Donelan‐McCall, N. & Turner, L. (2004). Mothers’ Regulation Strategies in Response to Toddlers’ Affect: Links to Later Emotion Self‐Regulation. *Social Development*, *13*(1), 40–55. https://doi.org/10.1111/j.1467-9507.2004.00256.x

Sprecher, E. A., Cresswell, C., Kerr-Davis, A., Sleed, M. & Midgley, N. (2023). Caregiver Mentalizing and Child Emotional Regulation: A Novel Approach to Examining Bidirectional Impact. *Journal of Infant, Child, and Adolescent Psychotherapy*, *22*(4), 358–374. https://doi.org/10.1080/15289168.2023.2274200

Squires, J., Bricker, D. & Twombly, E. (2002). *PsycTESTS Dataset.* https://doi.org/10.1037/t11524-000

Stansbury, K [K.] & Sigman, M. (2000). Responses of preschoolers in two frustrating episodes: emergence of complex strategies for emotion regulation. *The Journal of genetic psychology*, *161*(2), 182–202. https://doi.org/10.1080/00221320009596705

Stępień-Nycz, M., Rostek, I., Byczewska-Konieczny, K., Kosno, M., Białecka-Pikul, M. & Białek, A. (2015). Emotional and attentional predictors of self-regulation in early childhood. *Polish Psychological Bulletin*, *46*(3), 421–432. https://doi.org/10.1515/ppb-2015-0049

Stieben, J., Lewis, M. D., Granic, I., Zelazo, P. D., Segalowitz, S. & Pepler, D. (2007). Neurophysiological mechanisms of emotion regulation for subtypes of externalizing children. *Development and psychopathology*, *19*(2), 455–480. https://doi.org/10.1017/S0954579407070228

Stifter & Augustine, M. (2019). Emotion Regulation. In V. LoBue, K. Pérez-Edgar & K. A. Buss (Hrsg.), *Handbook of Emotional Development* (S. 405–430). Springer International Publishing. https://doi.org/10.1007/978-3-030-17332-6_16

Stifter & Spinrad, T. L [Tracy L.] (2002). The Effect of Excessive Crying on the Development of Emotion Regulation. *Infancy*, *3*(2), 133–152. https://doi.org/10.1207/s15327078in0302_2

Stifter, C. A., Dollar, J. M. & Cipriano, E. A. (2011). Temperament and emotion regulation: the role of autonomic nervous system reactivity. *Developmental psychobiology*, *53*(3), 266–279. https://doi.org/10.1002/dev.20519

Stifter., Spinrad, T. L [T. L.] & Braungart-Rieker, J. M. (1999). Toward a developmental model of child compliance: the role of emotion regulation in infancy. *Child development*, *70*(1), 21–32. https://doi.org/10.1111/1467-8624.00003

Suarez, G. L., Morales, S., Miller, N. V., Penela, E. C., Chronis-Tuscano, A., Henderson, H. A. & Fox, N. A. (2021). Examining a developmental pathway from early behavioral inhibition to emotion regulation and social anxiety: The moderating role of parenting. *Developmental psychology*, *57*(8), 1261–1273. https://doi.org/10.1037/dev0001225

Sullivan, E. L., Holton, K. F., Nousen, E. K., Barling, A. N., Sullivan, C. A., Propper, C. B. & Nigg, J. T. (2015). Early identification of ADHD risk via infant temperament and emotion regulation: a pilot study. *Journal of child psychology and psychiatry, and allied disciplines*, *56*(9), 949–957. https://doi.org/10.1111/jcpp.12426

Supplee, L., Skuban, E. M [E. M.], Shaw, D. S [D. S.] & Prout, J. (2009). Emotion regulation strategies and later externalizing behavior among European American and African American children. *Development and psychopathology*, *21*(2), 393–415. https://doi.org/10.1017/S0954579409000224

Supplee, L. H., Skuban, E. M [Emily Moye], Trentacosta, C. J., Shaw, D. S [Daniel S.] & Stoltz, E. (2011). Preschool boys' development of emotional self-regulation strategies in a sample at risk for behavior problems. *The Journal of genetic psychology*, *172*(2), 95–120. https://doi.org/10.1080/00221325.2010.510545

Susa, G., Mone, I., Salagean, D., Mihalca, L., Benga, O. & Friedlmeier, W [Wolfgang] (2014). The Relation between Maternal Perception of Toddler Emotion Regulation Abilities and Emotion Regulation Abilities Displayed by Children in a Frustration Inducing Task. *Procedia - Social and Behavioral Sciences*, *128*, 493–497. https://doi.org/10.1016/j.sbspro.2014.03.194

Suurland, J., van der Heijden, K. B., Smaling, H. J. A., Huijbregts, S. C. J., van Goozen, S. H. M. & Swaab, H. (2017). Infant autonomic nervous system response and recovery: Associations with maternal risk status and infant emotion regulation. *Development and psychopathology*, *29*(3), 759–773. https://doi.org/10.1017/S0954579416000456

Suveg, C., Sood, E., Barmish, A., Tiwari, S., Hudson, J. L. & Kendall, P. C. (2008). "I'd rather not talk about it": emotion parenting in families of children with an anxiety disorder. *Journal of Family Psychology*, *22*(6), 875–884. https://doi.org/10.1037/a0012861

Swingler, M. M., Perry, N. B., Calkins, S. D. & Bell, M. A. (2014). Maternal sensitivity and infant response to frustration: the moderating role of EEG asymmetry. *Infant behavior & development*, *37*(4), 523–535. https://doi.org/10.1016/j.infbeh.2014.06.010

Tamis-LeMonda, C. S., Bornstein, M. H [M. H.] & Baumwell, L. (2001). Maternal responsiveness and children's achievement of language milestones. *Child development*, *72*(3), 748–767. https://doi.org/10.1111/1467-8624.00313

Tan, P. Z., Armstrong, L. M. & Cole, P. M. (2013). Relations between Temperament and Anger Regulation over Early Childhood. *Social Development*, *22*(4). https://doi.org/10.1111/j.1467-9507.2012.00674.x

Tang, Y [Yingying], Perry, N. B., He, T., Wu, D., Zhou, N. & Lin, X. (2024). Grandmother-grandchild physiological synchrony in Chinese three-generation families: Links with child emotion regulation. *Developmental psychobiology*, *66*(5), e22498. https://doi.org/10.1002/dev.22498

Tarle, S. J., Alderson, R. M., Arrington, E. F. & Roberts, D. K. (2021). Emotion Regulation and Children With Attention-Deficit/Hyperactivity Disorder: The Effect of Varying Phonological Working Memory Demands. *Journal of attention disorders*, *25*(6), 851–864. https://doi.org/10.1177/1087054719864636

Taskiran, C., Karaismailoglu, S., Cak Esen, H. T., Tuzun, Z., Erdem, A., Balkanci, Z. D., Dolgun, A. B. & Cengel Kultur, S. E. (2018). Clinical features and subjective/physiological responses to emotional stimuli in the presence of emotion dysregulation in attention-deficit hyperactivity disorder. *Journal of clinical and experimental neuropsychology*, *40*(4), 389–404. https://doi.org/10.1080/13803395.2017.1353952

Taylor, S. G., Siceloff, E. R., Roberts, A. M., Bradley, W. J., Bridges, R. M., Lorch, E. P., Danielson, C. K. & Flory, K. (2020). Emotion Dysregulation and Sluggish Cognitive Tempo as Moderators of Cortisol Responsivity in Children with ADHD. *Advances in Neurodevelopmental Disorders*, *4*(3), 227–240. https://doi.org/10.1007/s41252-020-00156-9

Tenenbaum, R. B., Musser, E. D., Morris, S., Ward, A. R., Raiker, J. S., Coles, E. K. & Pelham, W. E. (2019). Response Inhibition, Response Execution, and Emotion Regulation among Children with Attention-Deficit/Hyperactivity Disorder. *Journal of abnormal child psychology*, *47*(4), 589–603. https://doi.org/10.1007/s10802-018-0466-y

Thibodeau‐Nielsen, R. B., Turley, D., DeCaro, J. A., Gilpin, A. T. & Nancarrow, A. F. (2021). Physiological substrates of imagination in early childhood. *Social Development*, *30*(3), 867–882. https://doi.org/10.1111/sode.12505

Thompson, R. A. (1994). Emotion Regulation: A Theme in Search of Definition: Monographs of the Society for Research in Child Development, 59(2/3), 25. *Monographs of the Society for Research in Child Development*, *59*(2/3), 25. https://doi.org/10.2307/1166137

Thompson, R. A., Lewis, M. D. & Calkins, S. D. (2008). Reassessing Emotion Regulation. *Child Development Perspectives*, *2*(3), 124–131. https://doi.org/10.1111/j.1750-8606.2008.00054.x

Tottenham, N., Hare, T. A., Quinn, B. T., McCarry, T. W., Nurse, M., Gilhooly, T., Millner, A., Galvan, A., Davidson, M. C., Eigsti, I.‑M., Thomas, K. M., Freed, P. J., Booma, E. S., Gunnar, M. R., Altemus, M., Aronson, J. & Casey, B. J. (2010). Prolonged institutional rearing is associated with atypically large amygdala volume and difficulties in emotion regulation. *Developmental science*, *13*(1), 46–61. https://doi.org/10.1111/j.1467-7687.2009.00852.x

Tottenham, N., Tanaka, J. W., Leon, A. C., McCarry, T., Nurse, M., Hare, T. A., Marcus, D. J., Westerlund, A., Casey, B. J. & Nelson, C. (2009). The NimStim set of facial expressions: judgments from untrained research participants. *Psychiatry research*, *168*(3), 242–249. https://doi.org/10.1016/j.psychres.2008.05.006

Trentacosta, C. J. & Shaw, D. S [Daniel S.] (2009). Emotional Self-Regulation, Peer Rejection, and Antisocial Behavior: Developmental Associations from Early Childhood to Early Adolescence. *Journal of Applied Developmental Psychology*, *30*(3), 356–365. https://doi.org/10.1016/j.appdev.2008.12.016

Tronick (1990). The infant regulatory scoring system (IRSS). . *Unpublished manuscript, Children’s Hospital/Harvard Medical School, Boston.*

Tronick, Als, H., Adamson, L., Wise, S. & Brazelton, T. B. (1978). The infant's response to entrapment between contradictory messages in face-to-face interaction. *Journal of the American Academy of Child Psychiatry*, *17*(1), 1–13. https://doi.org/10.1016/s0002-7138(09)62273-1

Tronick, E., Als, H. & Brazelton, Τ. B. (1980). The Infant’s Communicative Competencies and the Achievement of Intersubjectivity. In M. R. Key (Hrsg.), *The Relationship of Verbal and Nonverbal Communication* (S. 261–274). DE GRUYTER MOUTON. https://doi.org/10.1515/9783110813098.261

Troy, A. S., Wilhelm, F. H., Shallcross, A. J. & Mauss, I. B. (2010). Seeing the silver lining: cognitive reappraisal ability moderates the relationship between stress and depressive symptoms. *Emotion (Washington, D.C.)*, *10*(6), 783–795. https://doi.org/10.1037/a0020262

Tsotsi, S., Borelli, J. L., Abdulla, N. B., Tan, H. M., Sim, L. W., Sanmugam, S., Tan, K. H., Chong, Y. S., Qiu, A., Chen, H. & Rifkin-Graboi, A. (2020). Maternal sensitivity during infancy and the regulation of startle in preschoolers. *Attachment & human development*, *22*(2), 207–224. https://doi.org/10.1080/14616734.2018.1542737

Tsotsi, S., Borelli, J. L., Backer, M., Veragoo, N., Abdulla, N., Tan, K. H., Chong, Y. S., Chen, H., Meaney, M. J., Broekman, B. & Rifkin-Graboi, A. (2023). Preschoolers' emotion reactivity and regulation: Links with maternal psychological distress and child behavior problems. *Development and psychopathology*, *35*(3), 1079–1091. https://doi.org/10.1017/S0954579421000936

Tu, K. M., Li, X [Xiaomei] & Cohen, J. R. (2019). The "Heart" of depression during early adolescence. *Developmental psychobiology*, *61*(8), 1168–1179. https://doi.org/10.1002/dev.21862

Tumanova, V., Wilder, B., Gregoire, J., Baratta, M. & Razza, R. (2020). Emotional Reactivity and Regulation in Preschool-Age Children Who Do and Do Not Stutter: Evidence From Autonomic Nervous System Measures. *Frontiers in human neuroscience*, *14*, 600790. https://doi.org/10.3389/fnhum.2020.600790

Übel, S., Leutgeb, V. & Schienle, A. (2015). Electrocortical effects of a disgust placebo in children. *Biological psychology*, *108*, 78–84. https://doi.org/10.1016/j.biopsycho.2015.03.015

Urbain, C., Sato, J., Hammill, C., Duerden, E. G. & Taylor, M. J. (2019). Converging function, structure, and behavioural features of emotion regulation in very preterm children. *Human brain mapping*, *40*(11), 3385–3397. https://doi.org/10.1002/hbm.24604

Urbain, C., Sato, J., Pang, E. W. & Taylor, M. J. (2017). The temporal and spatial brain dynamics of automatic emotion regulation in children. *Developmental cognitive neuroscience*, *26*, 62–68. https://doi.org/10.1016/j.dcn.2017.05.004

Ursache, A., Blair, C., Stifter, C. & Voegtline, K. (2013). Emotional reactivity and regulation in infancy interact to predict executive functioning in early childhood. *Developmental psychology*, *49*(1), 127–137. https://doi.org/10.1037/a0027728

Usler, E., Foti, D. & Weber, C. (2020). Emotional reactivity and regulation in 5- to 8-year-old children: An ERP study of own-age face processing. *International journal of psychophysiology : official journal of the International Organization of Psychophysiology*, *156*, 60–68. https://doi.org/10.1016/j.ijpsycho.2020.07.004

Valentovich, V., Goldberg, W. A., Garfin, D. R. & Guo, Y. (2018). Emotion Coregulation Processes between Mothers and their Children With and Without Autism Spectrum Disorder: Associations with Children's Maladaptive Behaviors. *Journal of autism and developmental disorders*, *48*(4), 1235–1248. https://doi.org/10.1007/s10803-017-3375-y

van Cauwenberge, V., van Leeuwen, K., Hoppenbrouwers, K. & Wiersema, J. R. (2017). Developmental changes in neural correlates of cognitive reappraisal: An ERP study using the late positive potential. *Neuropsychologia*, *95*, 94–100. https://doi.org/10.1016/j.neuropsychologia.2016.12.015

Vasilev, C. A., Crowell, S. E., Beauchaine, T. P., Mead, H. K. & Gatzke-Kopp, L. M. (2009). Correspondence between physiological and self-report measures of emotion dysregulation: a longitudinal investigation of youth with and without psychopathology. *Journal of child psychology and psychiatry, and allied disciplines*, *50*(11), 1357–1364. https://doi.org/10.1111/j.1469-7610.2009.02172.x

Villani, V., Ludmer, J., Gonzalez, A., Levitan, R., Kennedy, J., Masellis, M., Basile, V. S., Wekerle, C. & Atkinson, L. (2018). Dopamine receptor D2 (DRD2), dopamine transporter solute carrier family C6, member 4 (SLC6A3), and catechol-O-methyltransferase (COMT) genes as moderators of the relation between maternal history of maltreatment and infant emotion regulation. *Development and psychopathology*, *30*(2), 581–592. https://doi.org/10.1017/S0954579417001122

Volling, B. L., McElwain, N. L. & Miller, A. L. (2002). Emotion regulation in context: the jealousy complex between young siblings and its relations with child and family characteristics. *Child development*, *73*(2), 581–600. https://doi.org/10.1111/1467-8624.00425

Walcott, C. M. & Landau, S. (2004). The relation between disinhibition and emotion regulation in boys with attention deficit hyperactivity disorder. *Journal of clinical child and adolescent psychology : the official journal for the Society of Clinical Child and Adolescent Psychology, American Psychological Association, Division 53*, *33*(4), 772–782. https://doi.org/10.1207/s15374424jccp3304_12

Walden, T. A., Frankel, C. B., Buhr, A. P., Johnson, K. N., Conture, E. G. & Karrass, J. M. (2012). Dual diathesis-stressor model of emotional and linguistic contributions to developmental stuttering. *Journal of abnormal child psychology*, *40*(4), 633–644. https://doi.org/10.1007/s10802-011-9581-8

Wang & Saudino, K. J. (2011). Emotion Regulation and Stress. *Journal of Adult Development*, *18*(2), 95–103. https://doi.org/10.1007/s10804-010-9114-7

Wang, H., Mai, X., Han, Z. R., Hu, Y. & Lei, X. (2018). Linkage between Parent-Child Frontal Resting Electroencephalogram (EEG) Asymmetry: The Moderating Role of Emotional Parenting. *Journal of Child and Family Studies*, *27*(9), 2990–2998. https://doi.org/10.1007/s10826-018-1121-5

Warren, S. L., Zhang, Y., Duberg, K., Mistry, P., Cai, W., Qin, S., Bostan, S.‑N., Padmanabhan, A., Carrion, V. G. & Menon, V. (2020). Anxiety and Stress Alter Decision-Making Dynamics and Causal Amygdala-Dorsolateral Prefrontal Cortex Circuits During Emotion Regulation in Children. *Biological psychiatry*, *88*(7), 576–586. https://doi.org/10.1016/j.biopsych.2020.02.011

Wass, S. V., Clackson, K. & Leong, V. (2018). Increases in Arousal are More Long‐Lasting than Decreases in Arousal: On Homeostatic Failures During Emotion Regulation in Infancy. *Infancy : the official journal of the International Society on Infant Studies*, *23*(5), 628–649. https://doi.org/10.1111/infa.12243

Waters, S. F., Virmani, E. A., Thompson, R. A., Meyer, S., Raikes, H. A. & Jochem, R. (2010). Emotion Regulation and Attachment: Unpacking Two Constructs and Their Association. *Journal of Psychopathology and Behavioral Assessment*, *32*(1), 37–47. https://doi.org/10.1007/s10862-009-9163-z

Weinberg, Beeghly, M [M.] & Tronick, E. Z. (2003). Child and Caregiver Mutual Regulation (CCMR) Scoring System: Manual for scoring preschoolers’ self-regulatory, social, and affective behavior during the maternal still-face and other contexts. *Boston: Harvard Medical School & Children’s Hospital.*

Weinberg & Tronick, E. Z. (1994). Beyond the face: an empirical study of infant affective configurations of facial, vocal, gestural, and regulatory behaviors. *Child development*, *65*(5), 1503–1515. https://doi.org/10.1111/j.1467-8624.1994.tb00832.x

Weinberg & Tronick, E. Z [Edward Z.] (1996). Infant Affective Reactions to the Resumption of Maternal Interaction after the Still-Face. *Child development*, *67*(3), 905. https://doi.org/10.2307/1131869

Weinberg & Tronick (1999). Infant and caregiver engagement phases (ICEP). Unpublished Manual. *Children’s Hospital and Harvard Medical School, Boston.*

Weinberg, M. K., Beeghly, M [Marjorie], Olson, K. L. & Tronick, E [Ed] (2008). A Still-face Paradigm for Young Children: 2½ Year-olds’ Reactions to Maternal Unavailability during the Still-face. *The journal of developmental processes*, *3*(1), 4–22.

Wessing, I., Rehbein, M. A., Romer, G., Achtergarde, S., Dobel, C., Zwitserlood, P., Fürniss, T. & Junghöfer, M. (2015). Cognitive emotion regulation in children: Reappraisal of emotional faces modulates neural source activity in a frontoparietal network. *Developmental cognitive neuroscience*, *13*, 1–10. https://doi.org/10.1016/j.dcn.2015.01.012

Whitson, S. M. & El-Sheikh, M. (2003). Moderators of Family Conflict and Children's Adjustment and Health. *Journal of Emotional Abuse*, *3*(1-2), 47–73. https://doi.org/10.1300/J135v03n01_03

Wiefferink, C. H., Rieffe, C., Ketelaar, L. & Frijns, J. H. M. (2012). Predicting social functioning in children with a cochlear implant and in normal-hearing children: the role of emotion regulation. *International journal of pediatric otorhinolaryngology*, *76*(6), 883–889. https://doi.org/10.1016/j.ijporl.2012.02.065

Wielgus, M. D., Aldrich, J. T., Mezulis, A. H. & Crowell, S. E. (2016). Respiratory sinus arrhythmia as a predictor of self-injurious thoughts and behaviors among adolescents. *International journal of psychophysiology : official journal of the International Organization of Psychophysiology*, *106*, 127–134. https://doi.org/10.1016/j.ijpsycho.2016.05.005

Williford, A. P., Calkins, S. D. & Keane, S. P [Susan P.] (2007). Predicting change in parenting stress across early childhood: child and maternal factors. *Journal of abnormal child psychology*, *35*(2), 251–263. https://doi.org/10.1007/s10802-006-9082-3

Wilson, B. J. (1999). Entry behavior and emotion regulation abilities of developmentally delayed boys. *Developmental psychology*, *35*(1), 214–222. https://doi.org/10.1037/0012-1649.35.1.214

Wilton, M., Craig, W. M. & Pepler, D. J. (2000). Emotional Regulation and Display in Classroom Victims of Bullying: Characteristic Expressions of Affect, Coping Styles and Relevant Contextual Factors. *Social Development*, *9*(2), 226–245. https://doi.org/10.1111/1467-9507.00121

Witt, A., Theurel, A., Tolsa, C. B., Lejeune, F., Fernandes, L., van Jonge, L. H. de, Monnier, M., Bickle Graz, M., Barisnikov, K., Gentaz, E. & Hüppi, P. S. (2014). Emotional and effortful control abilities in 42-month-old very preterm and full-term children. *Early human development*, *90*(10), 565–569. https://doi.org/10.1016/j.earlhumdev.2014.07.008

Wittenburg, P., Brugman, H., Russel, A., Klassmann, A. & Sloetjes, H. (2006). ELAN: a Professional Framework for Multimodality Research. In N. Calzolari, K. Choukri, A. Gangemi, B. Maegaard, J. Mariani, J. Odijk & D. Tapias (Hrsg.), *Proceedings of the Fifth International Conference on Language Resources and Evaluation (LREC‘06).* European Language Resources Association (ELRA). https://aclanthology.org/L06-1082/

Woltering, S., Lishak, V., Hodgson, N., Granic, I. & Zelazo, P. D. (2016). Executive function in children with externalizing and comorbid internalizing behavior problems. *Journal of child psychology and psychiatry, and allied disciplines*, *57*(1), 30–38. https://doi.org/10.1111/jcpp.12428

Woodward, L. J., Lu, Z., Morris, A. R. & Healey, D. M. (2017). Preschool self regulation predicts later mental health and educational achievement in very preterm and typically developing children. *The Clinical neuropsychologist*, *31*(2), 404–422. https://doi.org/10.1080/13854046.2016.1251614

Wu, Q. & Feng, X. (2020). Infant emotion regulation and cortisol response during the first 2 years of life: Association with maternal parenting profiles. *Developmental psychobiology*, *62*(8), 1076–1091. https://doi.org/10.1002/dev.21965

Wu, Q., Feng, X., Gerhardt, M. & Wang, L. (2020). Maternal depressive symptoms, rumination, and child emotion regulation. *European child & adolescent psychiatry*, *29*(8), 1125–1134. https://doi.org/10.1007/s00787-019-01430-5

Wu, Q., Feng, X., Hooper, E. & Ku, S. (2017). Maternal Emotion Socialization, Depressive Symptoms and Child Emotion Regulation: Child Emotionality as a Moderator. *Infant and Child Development*, *26*(1), Artikel e1979. https://doi.org/10.1002/icd.1979

Wu, Q., Feng, X., Yan, J., Hooper, E. G., Gerhardt, M. & Ku, S. (2022). Maternal emotion coaching styles in the context of maternal depressive symptoms: Associations with preschoolers' emotion regulation. *Emotion (Washington, D.C.)*, *22*(6), 1171–1184. https://doi.org/10.1037/emo0000916

Wu, Q. & Gazelle, H. (2021). Development of Infant High-Intensity Fear and Fear Regulation from 6 to 24 Months: Maternal Sensitivity and Depressive Symptoms as Moderators. *Research on Child and Adolescent Psychopathology*, *49*(11), 1473–1487. https://doi.org/10.1007/s10802-021-00842-9

Wu, Q., Yan, J. & Cui, M. (2021). A Developmental Hierarchical-Integrative Perspective on the Emergence of Self-Regulation: A Replication and Extension. *Child development*, *92*(5), e997-e1016. https://doi.org/10.1111/cdev.13559

Xie, J., Liu, S. & Fang, P. (2021). Cognitive training improves emotion regulation in Chinese preschool children. *Pediatrics international : official journal of the Japan Pediatric Society*, *63*(11), 1303–1310. https://doi.org/10.1111/ped.14661

Yaari, M., Rotzak, N. L., Mankuta, D., Harel-Gadassi, A., Friedlander, E., Eventov-Friedman, S., Bar-Oz, B., Zucker, D., Shinar, O. & Yirmiya, N. (2018). Preterm-infant emotion regulation during the still-face interaction. *Infant behavior & development*, *52*, 56–65. https://doi.org/10.1016/j.infbeh.2018.05.008

Yan, J. J., Feng, X., Shoppe-Sullivan, S. J., Gerhardt, M. & Wu, Q. (2021). Maternal Depressive Symptoms Predict Girls' but Not Boys' Emotion Regulation: A Prospective Moment-to-Moment Observation Study. *Research on Child and Adolescent Psychopathology*, *49*(9), 1227–1240. https://doi.org/10.1007/s10802-021-00806-z

Yang, M., Deng, X. & An, S. (2021). The Immediate and Lasting Effect of Emotion Regulation in Adolescents: An ERP Study. *International journal of environmental research and public health*, *18*(19). https://doi.org/10.3390/ijerph181910242

Yeh, Y.‑C. & Li, M.‑L. (2008). Age, Emotion Regulation Strategies, Temperament, Creative Drama, and Preschoolers' Creativity. *The Journal of Creative Behavior*, *42*(2), 131–149. https://doi.org/10.1002/j.2162-6057.2008.tb01291.x

Zantinge, G., van Rijn, S., Stockmann, L. & Swaab, H. (2017). Physiological Arousal and Emotion Regulation Strategies in Young Children with Autism Spectrum Disorders. *Journal of autism and developmental disorders*, *47*(9), 2648–2657. https://doi.org/10.1007/s10803-017-3181-6

Zeegers, M. A. J., Vente, W. de, Nikolić, M., Majdandžić, M., Bögels, S. M. & Colonnesi, C. (2018). Mothers' and fathers' mind-mindedness influences physiological emotion regulation of infants across the first year of life. *Developmental science*, *21*(6), e12689. https://doi.org/10.1111/desc.12689

Zelkowitz, R. L. & Cole, D. A. (2016). Measures of Emotion Reactivity and Emotion Regulation: Convergent and Discriminant Validity. *Personality and Individual Differences*, *102*, 123–132. https://doi.org/10.1016/j.paid.2016.06.045

Zengin-Bolatkale, H., Conture, E. G., Key, A. P., Walden, T. A. & Jones, R. M. (2018). Cortical associates of emotional reactivity and regulation in childhood stuttering. *Journal of fluency disorders*, *56*, 81–99. https://doi.org/10.1016/j.jfludis.2018.04.001

Zeytinoglu, S., Calkins, S. D. & Leerkes, E. M. (2022). Autonomic profiles and self-regulation outcomes in early childhood. *Developmental science*, *25*(5), e13215. https://doi.org/10.1111/desc.13215

Zhang, Fagan, S. E. & Gao, Y. (2017). Respiratory Sinus Arrhythmia Activity Predicts Internalizing and Externalizing Behaviors in Non-referred Boys. *Frontiers in psychology*, *8*, 1496. https://doi.org/10.3389/fpsyg.2017.01496

Zhang, Li, X [Xiying], Liu, X [Xia], Duan, X., Wang, D. & Shen, J. (2013). Distraction reduces theta synchronization in emotion regulation during adolescence. *Neuroscience letters*, *550*, 81–86. https://doi.org/10.1016/j.neulet.2013.05.070

Zhang, W., Ding, Q., Chen, N., Wei, Q., Zhao, C., Zhang, P., Li, X [Xiying], Liu, Q. & Li, H. (2016). The development of automatic emotion regulation in an implicit emotional Go/NoGo paradigm and the association with depressive symptoms and anhedonia during adolescence. *NeuroImage. Clinical*, *11*, 116–123. https://doi.org/10.1016/j.nicl.2016.01.018

Zimmermann & Spangler, G. (2016). Effects of Gene × Attachment Interaction on Adolescents' Emotion Regulation and Aggressive Hostile Behavior Towards their Mothers during a Computer Game. *Frontiers in human neuroscience*, *10*, 254. https://doi.org/10.3389/fnhum.2016.00254

Zimmermann, L. K. & Stansbury, K [Kathy] (2003). The influence of temperamental reactivity and situational context on the emotion-regulatory abilities of 3-year-old children. *The Journal of genetic psychology*, *164*(4), 389–409. https://doi.org/10.1080/00221320309597886

Zimmermann, L. K. & Stansbury, K [Kathy] (2004). The influence of emotion regulation, level of shyness, and habituation on the neuroendocrine response of three-year-old children. *Psychoneuroendocrinology*, *29*(8), 973–982. https://doi.org/10.1016/j.psyneuen.2003.09.003
